# Leveraging electronic health record data to evidence collider stratification bias and inform clinical epidemiology: obesity, diabetes, and rotator cuff tears

**DOI:** 10.1093/aje/kwag117

**Published:** 2026-06-01

**Authors:** Simone D Ueland, Til B Basnet, Wenting Liu, Henry H Ong, Srushti Gangreddi, Wei-Qi Wei, Wanqing Wen, Katherine E Hartmann, Nitin B Jain, Ayush Giri

**Affiliations:** Vanderbilt University School of Medicine, Nashville, TN, United States; Division of Epidemiology, Department of Medicine, Vanderbilt Health, Nashville, TN, United States; Department of Orthopedic Surgery, Boston Medical Center, Boston, MA, United States; Division of Epidemiology, Department of Medicine, Vanderbilt Health, Nashville, TN, United States; Department of Biostatistics and Data Science, University of Texas Health Science Center at Houston, Houston, TX, United States; Department of Biomedical Informatics, Vanderbilt Health, Nashville, TN, United States; Department of Biomedical Informatics, Vanderbilt Health, Nashville, TN, United States; Department of Biomedical Informatics, Vanderbilt Health, Nashville, TN, United States; Division of Epidemiology, Department of Medicine, Vanderbilt Health, Nashville, TN, United States; Center for Clinical & Translational Science, University of Kentucky, Lexington, KY, United States; Department of Obstetrics and Gynecology, University of Kentucky, Lexington, KY, United States; Department of Physical Medicine and Rehabilitation, University of Michigan, Ann Arbor, MI, United States; Department of Orthopaedic Surgery, University of Michigan, Ann Arbor, MI, United States; Division of Epidemiology, Department of Medicine, Vanderbilt Health, Nashville, TN, United States; Division of Quantitative and Clinical Sciences, Department of Obstetrics and Gynecology, Vanderbilt Health, Nashville, TN, United States

**Keywords:** electronic health records, selection bias, rotator cuff, shoulder, sports injury

## Abstract

Electronic health record (EHR) data are increasingly used for case–control investigations. Using multiple control groups in de-identified EHR data, we evidence how conditioning on imaging (descendant of a collider: symptomatology) can perturb exposure estimations enough to reverse conclusions. Because imaging is often required for rotator cuff tear diagnosis, some argue imaging should be required for control selection. We constructed 2 control groups (with vs without imaging) to evaluate selection bias through collider stratification involving metabolic exposures—body mass index (BMI), type 1 diabetes (T1D), and type 2 diabetes (T2D)—and rotator cuff tears. Cases and controls were identified using validated algorithms. We compared baseline characteristics and performed multivariable logistic regression across designs. Cases were older and more likely to have arthritis (57%), ligamentous disease (9%), and prior shoulder injury (99%) than controls. Controls requiring imaging more closely resembled cases than controls not requiring imaging, with more arthritis (9% vs 1%), ligamentous disease (6% vs 2%), and prior shoulder injury (54% vs 7%). T1D prevalence was 3% in cases, 4% in controls with imaging, and 1% in controls without, compared to ~1% nationally. T1D was positively associated with tears using controls without imaging (aOR = 1.78; 95% CI, 1.64-1.92), but inversely using controls with imaging (aOR = 0.75; 0.57-0.97).

## Introduction

Electronic health records (EHRs) are increasingly prominent sources of data for clinical and observational studies. More recently, linkage of EHRs to large-scale biobanks, including the Million Veteran Program, Vanderbilt Health Biorepository, Michigan Genetics Initiative, and UK Biobank, among others, further enhances their value in asking questions related to molecular and genetic epidemiology previously not possible with traditional cohort or case–control studies. With the ever-growing sample sizes, manual chart review for every individual is impractical, necessitating reliance on algorithmic approaches to define exposures and outcomes. The strength of these algorithmic approaches lies in their ability to efficiently capture vast amounts of clinical data, but their validity hinges on accurate ascertainment of phenotypes.

Substantial focus in the literature regarding validity is directed toward assessing misclassification of outcome through predictive values, sensitivity, and specificity. Indeed, studies report a wide range of positive and negative predictive values (PPV and NPV) regarding accuracy with the International Classification of Diseases (ICD) and Current Procedural Terminology (CPT) codes ranging from 25% to 100% depending on the condition or procedure being studied.^1-8^ Manually curated algorithms that contextualize diagnosis and billing practices and incorporate elements of temporality or similar complexity show greater accuracy.^9^^,^^10^

Another source of validity often neglected is whether the algorithms undertake appropriate selection strategy for controls derived from EHR records. Efforts to reduce misclassification can conflict with the primary goal of control selection in case–control studies, which is to ensure that controls provide an exposure distribution representative of the population that produced cases. Algorithmic features in control selection strategy that over- or underestimate population exposure distribution can lead to biased results even in scenarios that may have close to 100% PPV and NPV.

The scenario: Diagnostic codes for orthopedic conditions such as rotator cuff tears are often entered into EHRs preemptively to facilitate imaging confirmation or referral to outpatient specialty clinics. Reliance on ICD codes alone for musculoskeletal conditions are notoriously imprecise for phenotyping.^4-8^ A suggested approach to reducing misclassification is to require imaging-based verification of rotator cuff tear to confirm case classification, which would increase algorithm PPV. That same logic may be extended to posit that since cases were required to have imaging confirmation, controls also should have the same requirement: individuals required to have imaging and confirmed not to have rotator cuff tears. We postulated requiring imaging for controls chosen from a population, whose indication for imaging is pain/shoulder symptomatology, would necessarily enrich other shoulder-related conditions in controls, and if these shoulder-related conditions are also affected by an exposure of interest, the observed association between exposure and rotator cuff tear would be biased. In most circumstances, the postulated hypothesis remains untested and perhaps relegated to a sentence on possible bias in the discussion.

Here, using data from the Vanderbilt University Medical Center (VUMC) de-identified EHR system, the synthetic derivative (SD), we explore this scenario to implement case and control phenotyping strategies for rotator cuff tear as a motivating case study example to (1) inform how specific control selection strategies from EHR data can induce collider stratification and may lead to bias; (2) explicate how fixation for higher predictive value, the idea of “pure/healthy/clean controls,” motivates the source of bias; (3) evidence how complete case analysis can further exaggerate bias; and (4) inform how EHR records can be used proactively to test multiple case–control sets to further improve study design and inference. We evaluate whether commonly studied exposures, such as obesity and preexisting type 1 diabetes (T1D) and type 2 diabetes (T2D), are associated with rotator cuff tears in an EHR-based case–control study and how sample selection choices can induce bias for some exposures under study. We hypothesized that requiring imaging for controls selected from EHR data would lead to selection bias due to collider stratification and depicted a putative mechanism for such bias a priori using directed acyclic graphs (Figure 1).

**Figure 1:**
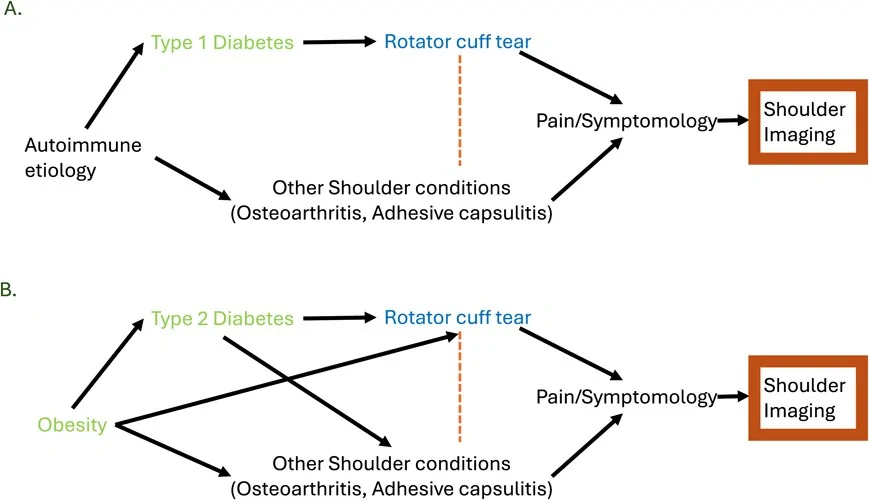
Directed acyclic graphs depicting collider stratification bias by conditioning on shoulder imaging. Individuals are indicated for shoulder imaging, such as MRI, by their provider once they present with pain or shoulder-related symptomatology. The reason for symptomatology could be due to either rotator cuff tear or other shoulder-related pathologies. Shoulder imaging is a descendant of a collider pain/symptomatology, and requiring shoulder imaging (conditioning on the descendant of the collider as depicted by the box around “Shoulder Imaging”) for control selection enriches the control population to have a larger proportion of other shoulder-related pathologies. Therefore, by restricting on imaging, an inverse correlation is created between rotator cuff tear and other shoulder pathologies (depicted by the orange dashed line). In such a study evaluating the relationship between an exposure and rotator cuff tears, if the shoulder-related pathologies are associated with exposure, type 1 diabetes (A) or type 2 diabetes, or obesity (B), then the association between the exposure and rotator cuff tear could be biased.

## Methods

We first developed 3 case and 2 control algorithms for rotator cuff tears using the VUMC SD, a unique de-identified clinical records database derived from VUMC medical records with over 2 million unique individuals.^11^ Algorithm development is detailed elsewhere.^9^ Briefly, we developed and validated 2 broad classes of algorithms, ones requiring imaging verification of cases and controls (imaging-based algorithms), and ones not requiring imaging-based verification of case and control status (no imaging verification). Imaging-based algorithms included a combination of ICD-9/10, and CPT codes to identify cases and controls and required MRI, CT, or ultrasound imaging to verify case and control status. Algorithms not requiring imaging verification used either diagnostic and CPT-based definitions or natural language processing (NLP)–based definitions for cases, and a combination of NLP and code-based methods for determining controls. The non–imaging-based NLP-derived control definition served as the control criteria for both code-based and NLP-based case algorithms.

### Case–control sets

Using these definitions, we constructed 3 case–control study sets in individuals over 35 years of age in the SD: (1) imaging-based cases and controls, (2) code-based no-imaging-required cases and NLP-based + code-based no-imaging-required controls, and (3) NLP-based no-imaging-required cases and NLP-based + code-based no-imaging-required controls. To maximize case sample size and maintain optimal power, all cases were included in the study and controls were randomly selected from the corresponding image-based or non–image-based control algorithm in a 1:4 case to control ratio. Due to lack of a “baseline” visit as in cohort studies, or a date of recruitment visit as in prospectively recruited case–control studies, cases were indexed at first diagnosis/mention of degenerative cuff tear (DCT) at or above 35 years of age in the EHR. Controls were indexed at a randomly selected visit occurring at or after 35 years of age in the EHR. We extracted information on age, race/ethnicity, and presence of additional medical comorbidities that were present on or prior to index date of case/control status. To maintain temporality of assessing body mass index (BMI) as a preceding risk factor for DCT, BMI reported at the most recent visit leading up to 2 years before DCT diagnosis date for cases and randomly chosen indexed visit for controls was extracted. Participants were noted to have T1D if they had any mention of one of the ICD codes for insulin-dependent diabetes noted in their record on or before the date of diagnosis for cases or the indexed visit date for controls. Similarly, participants were noted to have T2D if they had any mention of one of the ICD codes for non–insulin-dependent diabetes noted in their record on or before the date of diagnosis for cases or randomly indexed visit date for controls. Participants were categorized as having any diabetes if they had any ICD codes for T1D, T2D, or an unknown form of diabetes (gestational diabetes was not included in this category). Any participant who did not have any diagnostic code for diabetes was assumed not to have diabetes and was categorized as “no diabetes” and was the reference group for all categories of diabetes. All ICD codes are listed in Table S1.

### Statistical analyses

We used univariate and multivariable logistic regression models to investigate associations between obesity and diabetes with DCT. Obesity was modeled continuously and categorically as modified WHO standard BMI categories, where normal weight is <25 kg/m^2^, overweight is 25 to 29.9 kg/m^2^, and obese is ≥30 kg/m^2^. Diabetes was modeled as binary T1D (yes/no), T2D (yes/no), and any diabetes (yes/no). Multivariable models for BMI as the exposure included age, sex, race/ethnicity, and T1D. Models evaluating diabetes (T1D, T2D, or any diabetes) as the exposure included age, sex, and race/ethnicity and were assessed with and without adjustment for BMI (as restricted cubic splines), and in complete case vs multiple imputation scenarios. We used restricted cubic splines to evaluate evidence of nonlinearity for continuous variables and modeled them appropriately. Since measurements of BMI went back up to 2 years prior to the index visit, we conducted sensitivity analyses, further adjusting for length of time that elapsed between BMI evaluation and index case/control status.

To address missing BMI data across the NLP (61.2%), Code Image-Required (34.0%), and Code Non-Image (57.9%) cohorts, we employed Multiple Imputation by Chained Equations under the missing-at-random assumption. Independent imputation models were specified for each of the 3 datasets. The imputation models incorporated all analytical covariates and the primary outcome (DCT status) to preserve correlational structure of the data.^12^ Continuous variables were imputed using predictive mean matching. To robustly account for maximum missingness proportion and guarantee algorithmic convergence, we generated 65 imputed datasets over 10 iterations per cohort. Convergence and distributional plausibility were confirmed via diagnostic trace and density plots (File S1). Finally, logistic regression models evaluating associations of BMI and diabetes with DCT status were fitted across imputed datasets, with parameter estimates and standard errors pooled using Rubin’s rules.

### Assessment of bias

We evaluated 2 common sources of bias: collider stratification and selection due to complete case analysis or restriction based on data availability. The primary goal of controls in a case–control study is to obtain reliable estimates of exposure distribution for a population that gave rise to both cases and controls.^13-15^ When sampling of controls is done in such a manner that it is not independent of the exposure distribution that we are trying to estimate, it can lead to a bias. Such biases have been thoroughly described in epidemiologic literature as collider stratification bias, where Berkson’s bias is described as a specific case where sample selection is restricted to one stratum of the collider.^16-21^ To explicate the impact of collider stratification due to the requirement of imaging in controls in the EHR setting, we compare the proportion of controls with other musculoskeletal conditions in controls with and without imaging requirements. We then compare the proportion of exposure across control groups requiring imaging versus those not requiring imaging and compare these exposure distributions to population prevalence estimates in the United States. Finally, we compare odds ratios across case–control sets to evaluate the impact on effect estimates and inference. To evaluate selection induced by complete case analysis, we compare exposure prevalence and odds ratios in full vs reduced/effective sample sizes imposed by complete case analyses and further evaluate whether multiple imputation can rescue the effects of this type of selection.

## Results

### Participant characteristics across case–control sets

Algorithms identified 8482 code-based no-imaging-required cases with 33 928 controls sampled from the no-imaging-required control pool, 54 846 NLP-based no-imaging-required cases with 219 384 controls sampled from the no-imaging-required control pool, and 2632 imaging-required cases with 10 528 imaging-required controls sampled from a separate imaging-required controls pool (Table 1). Cases were generally older than controls across all designs, with mean ages ranging from approximately 61 to 63 years across case sets and 57 to 59 years across control sets. Imaging-required cases and controls tended to be slightly younger than no-imaging-required counterparts. Sex distributions were relatively balanced among cases, whereas controls were slightly more likely to be females, especially in non–imaging-based control groups (55%). Most participants were identified as non–Hispanic White, accounting for about 66% to 77% of cases and 53% to 68% of controls. No-imaging-required controls were less likely to be identified as non–Hispanic White on average than corresponding case groups, whereas imaging-required controls were more likely to be non–Hispanic White than no-imaging-required control groups (68% vs 53%) and were similar or more likely to be non–Hispanic White than other case groups. Individuals identifying as non–Hispanic Black accounted for approximately 6% to 9% of both cases and controls, while other racial and ethnic groups, including Hispanic and Asian participants, comprised smaller proportions (Table 1). Across all designs, data on BMI were more likely to be available in cases (66% in code-based cases, 51% in NLP-based cases, and 77% in code-based-imaging-required cases) than controls (36% in code-based + NLP-based controls across both samples and 63% in code-based-imaging-required controls) (Table 1).

**Table 1:** Demographics and characteristics of rotator cuff tear case–control sets with and without imaging.

		**Non–image required**						**Image required**		
		Cases (code)	Controls^a^ (code + NLP)	SMD	Cases (NLP)	Controls^a^ (code + NLP)	SMD	Cases (code)	Controls (code)	SMD
Total count (*N*)										
All SD^b^		8482	33 928		54 846	219 384		2632	10 528	
Participants with BMI data		5603	12 256		28 201	78 160		2045	640	
Sex (*N*)										
Male		4680 (55%)	15 341 (45%)	0.20	28 319 (52%)	99 144 (45%)	0.14	1384 (53%)	5277 (50%)	0.06
Female		3802 (45%)	18 568 (55%)	0.20	26 519 (48%)	120 175 (55%)	0.14	1248 (47%)	5251 (50%)	0.06
Unknown			18 (<0.1%)		8 (<0.1%)	65 (<0.1%)	0			
Age Mean (Std Dev)		60.52 (10.24)	59.22 (12.42)	0.11	62.44 (11.17)	59.27 (12.47)	0.27	61.43 (10.47)	56.83 (11.58)	0.42
Race/Eth (*N*)										
Non-Hispanic	White	5853 (63%)	17 917 (53%)	0.20	35 277 (64%)	116 685 (53%)	0.23	1751 (67%)	7117 (68%)	0.02
	Black	552 (7%)	2206 (7%)	0.00	3810 (7%)	13 746 (6%)	0.04	171 (6%)	965 (9%)	0.11
	Asian	40 (0%)	198 (1%)	0.10	221 (0%)	1424 (1%)	0.10	9 (0%)	82 (1%)	0.10
Hispanic		124 (2%)	157 (2%)	0.00	639 (1.9%)	904 (2%)	0.01	4023 (2%)	57 (2%)	0
Other		1311 (23%)	1880 (22%)	0.02	12 968 (38%)	14 634 (27%)	0.24	83 506 (38%)	644 (24%)	0.31
BMI continuous (kg/m^2^) Mean (Std Dev)		30.33 (6.43)	29.65 (7.24)	0.10	30.27 (6.81)	29.60 (8.08)	0.09	29.85 (6.20)	29.42 (7.01)	0.06
BMI categorical (*N*)										
Underweight <18.5 (kg/m^2^)		29 (1%)	222 (2%)	0.09	284 (1%)	1357 (2%)	0.08	14 (1%)	115 (2%)	0.08
Normal 18.5-24.9 (kg/m^2^)		1029 (18%)	3078 (25%)	0.17	5705 (20%)	19 774 (25%)	0.12	408 (20%)	1697 (26%)	0.14
Overweight 25-29.9 (kg/m^2^)		2036 (36%)	3996 (33%)	0.07	9573 (34%)	25 617 (33%)	0.02	755 (37%)	2219 (33%)	0.08
Obese > 30 (kg/m^2^)		2509 (45%)	4960 (40%)	0.10	12 639 (45%)	31 412 (40%)	0.10	868 (42%)	2609 (39%)	0.06
Diabetes (*N*)										
Type I		232 (3%)	393 (1%)	0.14	1387 (3%)	2465 (1%)	0.15	83 (3%)	382 (4%)	0.05
Type II		560 (7%)	1132 (3%)	0.17	3202 (6%)	7179 (3%)	0.14	246 (9%)	651 (6%)	0.11
Any		1197 (14%)	2546 (8%)	0.19	7477 (14%)	16 028 (7%)	0.22	476 (18%)	1589 (15%)	0.08
None		7285 (86%)	31 382 (92%)	0.19	47 369 (86%)	203 356 (93%)	0.22	2156 (82%)	8939 (86%)	0.08
Ligamentous disease (*N*)		595 (7%)	803 (2%)	0.24	3700 (7%)	5023 (2%)	0.23	234 (9%)	682 (6%)	0.11
Prior shoulder injury (*N*)		8260 (97%)	2450 (7%)	2.47	28 306 (52%)	15 586 (7%)	1.06	2594 (99%)	5710 (54%)	1.23
Arthritis (*N*)		3959 (47%)	351 (1%)	1.17	9590 (17%)	2173 (1%)	0.57	1499 (57%)	986 (9%)	1.09
VUMC utilization average days Mean (SD)		41.72 (65.64)	11.08 (25.28)	0.60	27.58 (53.39)	10.77 (24.86)	0.41	48.17 (72.29)	38.59 (66.03)	0.14

Abbreviations: N, number of participants; NLP, natural language processing; SMD, standardized mean deviation; SD, synthetic derivative; BMI, body mass index; Std Dev, standard deviation; VUMC, Vanderbilt University Medical Center.

a The non–image controls were sampled from the same, code + NLP non–image set, but randomly selected to match either code-based or NLP-based cases at a 4:1 ratio and thus were ultimately composed of different participants.

b This table includes information on all participants in the VUMC SD regardless of if BMI data were available.

### Assessment of collider stratification bias due to imaging requirement in controls

To test the hypothesized mechanism of conditioning on imaging, we compared prevalence of musculoskeletal comorbidities across all case–control sets (Table 1). Rotator cuff tear case sets were more likely to have musculoskeletal comorbidities than controls; however, the proportion of musculoskeletal comorbidities was substantially higher across imaging-required controls than no-imaging-required controls (Table 1).

We then compared prevalence of exposure distributions across case–control sets with and without imaging requirement (Table 1, Figure 2, and Table S2). Regardless of algorithm type, the proportion of rotator cuff tear cases with diabetes was similar across case sets for T1D (~3%), slightly elevated in imaging-required cases for T2D (9% in imaging-required cases and 6%-7% in no-imaging-required cases), and slightly elevated in imaging-required cases for any diabetes (18% in imaging-required cases and 14% in no-imaging-required cases) (Table 1, Figure 2, and Table S2). However, the proportion of controls with diabetes varied substantially between imaging-required and no-imaging-required control groups, with imaging-required controls (4% for T1D, 6% for T2D, and 15% for any diabetes) showing a 2 to 3 times higher proportion of diabetes than no-imaging-required control definitions (1% for T1D, 3% for T2D, and 7%-8% for any diabetes) (Table 1, Figure 2, and Table S2). For reference, in the United States, prevalence of T1D is <1%, and about 8.9% of the population has a diagnosis of diabetes (including T1D and T2D).^22^

**Figure 2:**
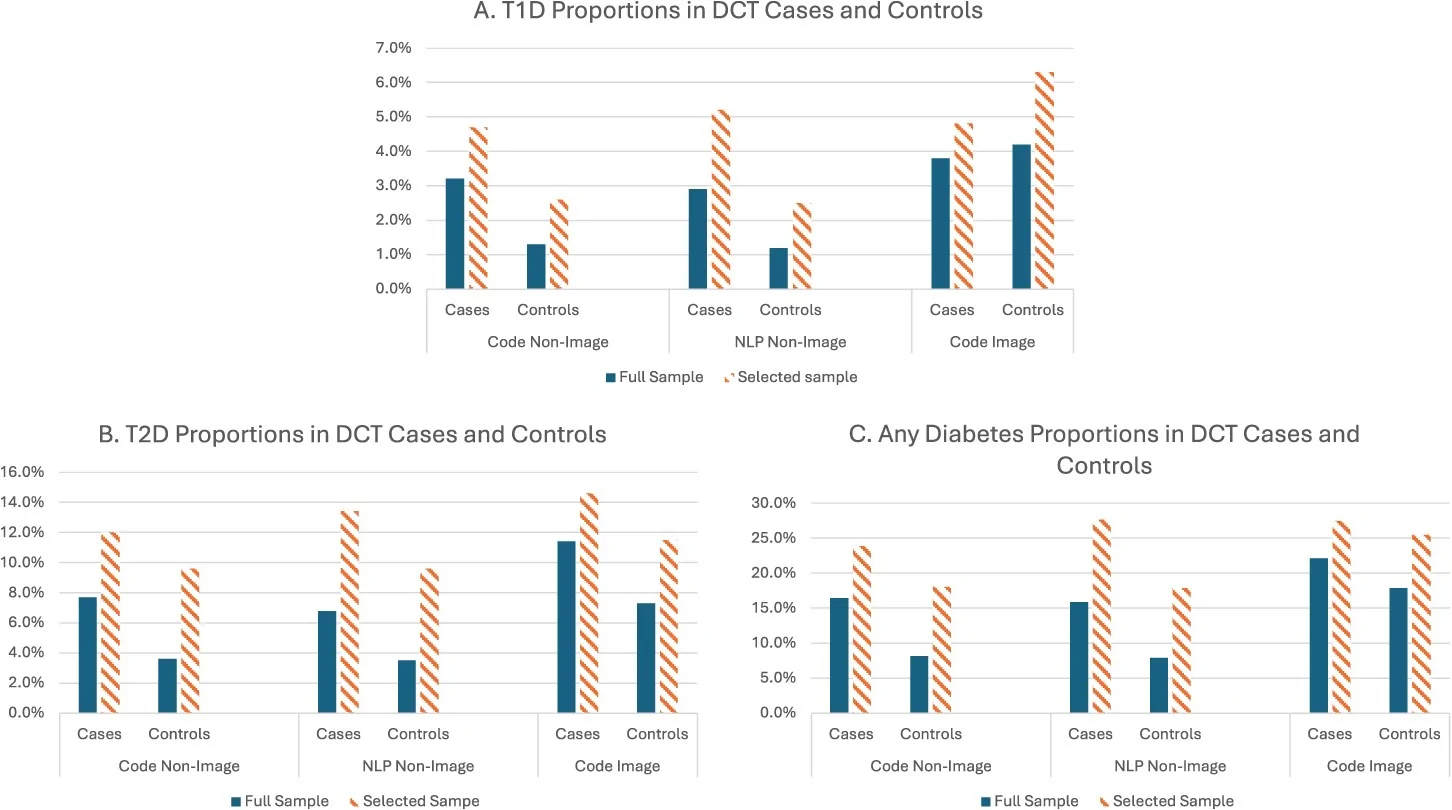
Comparing the proportion of exposure (type 1, type 2, and any diabetes) across case–control study sets for rotator cuff tears with and without imaging requirement. Exposure distribution across 3 case–control sets (code-based no-imaging-required cases and controls [Code Non-Image], natural language processing (NLP)-based no-imaging-required cases and controls [NLP Non-Image], and code-based imaging-required cases and controls [Code Image]) depicted for the following exposures: type 1 diabetes (A), type 2 diabetes (B), and any diabetes (C). Across all figures, solid bars represent exposure distribution in the full case–control study set originally sampled, whereas the striped bars represent exposure distribution in the sample selected by complete case analysis where individuals missing information on BMI are excluded from the analysis.

In regression models with or without multivariable adjustment for age, sex, and race/ethnicity, T2D and any diabetes were positively associated with rotator cuff tears and had the strongest magnitudes of association in no-imaging-required case–control sets (OR range, 1.77 to 2.13), while they were substantially attenuated in the imaging-required case–control set (1.12 to 1.57) (Figure 3 and Table S2). Similarly, odds ratios for T1D and rotator cuff tear were 2.19 (95% CI) in the code-based no-imaging-required set and 2.09 (95% CI) in the NLP-based no-imaging-required set, and these were completely reversed at 0.92 (95% CI) in the code-based imaging-required set (Figure 3 and Table S2).

**Figure 3:**
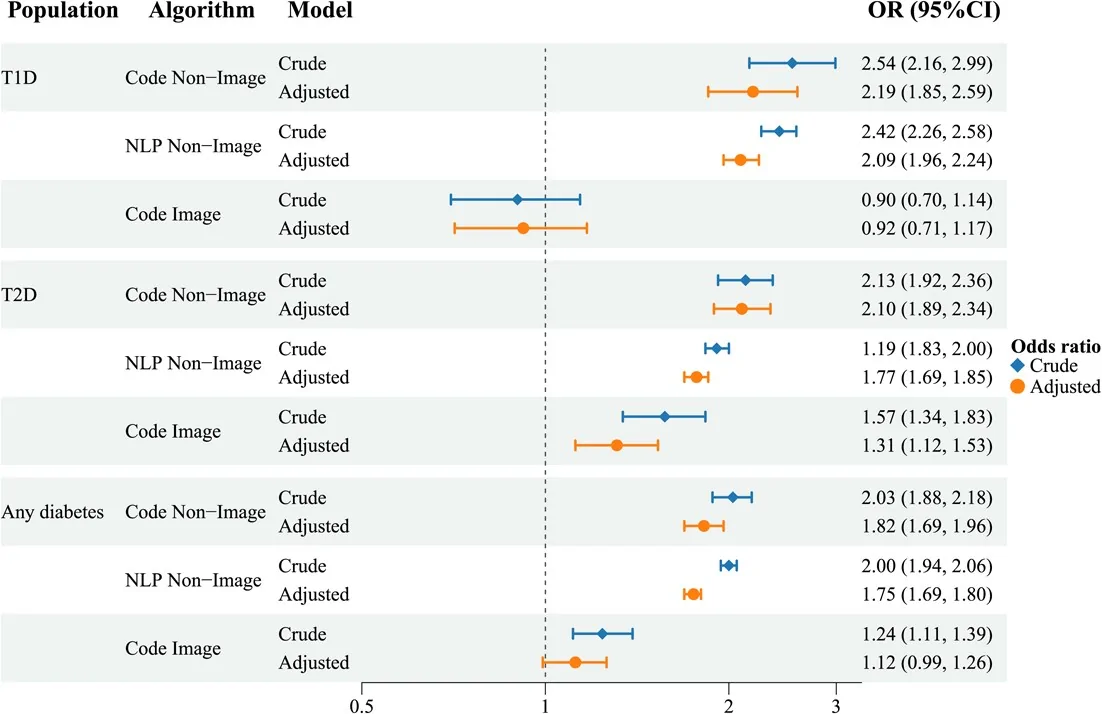
Forest plots showing association between diabetes exposures (type 1, type 2, and any diabetes) in relation to rotator cuff tears across study sets with and without requirement for imaging. Blue diamonds (crude) represent odds ratios and 95% confidence intervals from univariate models. Orange circles (adjusted) represent odds ratios and 95% confidence intervals from multivariable models adjusted for age, sex, and race/ethnicity. Odds ratios for all 3 exposures are attenuated for the case–control sets requiring imaging [code image], with or without adjustment for covariates.

Interestingly, the association between BMI and rotator cuff tear was not substantially different between no-imaging-required and imaging-required sets in unadjusted or multivariable regression models adjusted for age, sex, race/ethnicity, and T1D (Table 2). Prevalence of overweight (33%) and obesity (39%-40%) in controls was similarly distributed in imaging-required and no-imaging-required controls. For reference, in the United States, 30.7% are overweight and 42.4% have obesity.^23^ In multivariable-adjusted models, a 5-unit increase in BMI was associated with 10% increased odds of DCT for case–control study designs (OR = 1.10; CI, 1.08-1.13—Code Non-Image; CI, 1.09-1.11—NLP Non-Image, and CI: 1.06-1.15—Code Image-Required). Categorical analysis was similar across algorithm types and demonstrated graded effect, where risk of DCT increased with each increase in the category of BMI. Compared to individuals in the normal BMI group, individuals in the overweight group (OR = 1.26; 95% CI, 1.21-1.31) and obese group (OR = 1.45; 95% CI, 1.39-1.51) were more likely to have rotator cuff tears in the NLP-based cohort. Effect estimates were slightly larger in the code non–imaging-based and imaging-based designs (Table 2).

**Table 2:** Association between BMI and rotator cuff tears across case–control sets with and without imaging requirement.

	**Code non-image**				**NLP non-image**				**Code image-eequired**			
	**Cases** ***n* = 5603**	**Controls** ^**a**^ ***n* = 12 256**	**OR**	**95% CI**	**Cases** ***n* = 28 201**	**Controls** ^**a**^ ***n* = 78 160**	**OR**	**95% CI**	**Cases** ***n* = 2045**	**Controls** ***n* = 6640**	**OR**	**95% CI**
Obesity												
BMI continuous—unadjusted												
5-unit increase	5603	12 256	1.07	1.05-1.10	28 201	78 160	1.06	1.05-1.07	2045	6640	1.05	1.01-1.08
BMI continuous—adjusted^b^												
5-unit increase	4739	10 653	1.10	1.08-1.13	23 244	68 023	1.10	1.09-1.11	1682	5625	1.10	1.06-1.15
BMI categorical—unadjusted												
Underweight	29	222	0.39	0.26-0.57	284	1357	0.73	0.64-0.83	14	115	0.51	0.28-0.86
Normal	1029	3078	Ref	Ref	5705	19 774	Ref	Ref	408	1697	Ref	Ref
Overweight	2036	3996	1.52	1.40-1.67	9573	25 617	1.30	1.25-1.35	755	2219	1.38	1.21-1.58
Obese	2509	4960	1.51	1.39-1.65	12 639	31 412	1.39	1.35-1.45	868	2609	1.42	1.24-1.63
BMI categorical—adjusted^b^												
Underweight	27	211	0.38	0.24-0.56	258	1276	0.68	0.59-0.78	14	107	0.53	0.29-0.91
Normal	951	2852	Ref	Ref	5150	18 313	Ref	Ref	361	1540	Ref	Ref
Overweight	1788	3576	1.45	1.32-1.59	8159	22 814	1.26	1.21-1.31	652	1934	1.47	1.26-1.70
Obese	1973	4014	1.54	1.40-1.69	9677	25 620	1.45	1.39-1.51	655	2044	1.51	1.30-1.75
BMI categorical—imputed^c^ and adjusted^b^												
Underweight			0.57	0.42-0.79			0.82	0.73-0.92			0.69	0.40-1.18
Normal			Ref	Ref			Ref	Ref			Ref	Ref
Overweight			1.27	1.16-1.38			1.17	1.13-1.21			1.32	1.14-1.52
Obese			1.44	1.31-1.59			1.39	1.34-1.44			1.43	1.23-1.66

Abbreviations: N, number of participants; OR, odds ratio; 95% CI, 95% confidence interval; NLP, natural language processing; SD, synthetic derivative; BMI, body mass index; Ref, reference.

a The algorithm for control classification is identical for Code Non-Image and NLP, and represents all possible controls in the SD from which these 2 control groups were selected. The control groups represented in this table are different as they were randomly selected to meet a 1:4 case–control ratio.

b Adjusted for age, sex, race, and T1D.

c Results were generated after multiple imputation of BMI across datasets.

### Selection into the study

To explore selection induced by analytical choices, specifically complete case analysis in the context of data on missing covariates, we evaluated how study population characteristics and associations between diabetes exposures and rotator cuff tear are affected when restricting the analytic sample size to individuals for whom information on BMI is available (Figure 2, Tables S2 and S3) in univariable and multivariable analyses with and without adjustment for BMI (Figures 3 and 4). Starting with the scenario where obesity would be a confounder in the association between T2D and rotator cuff tear, we show limiting analytic sample size to individuals with information on BMI available attenuated unadjusted odds ratios from the code-only no-imaging-required set from ORUnadj-full sample: 2.13 (1.92-2.36) (Figure 3) to ORUnadj-comp-case: 1.26 (1.13-1.41) (Figure 4). Further adjustment for age, race/ethnicity, sex, and BMI only attenuated the OR slightly to 1.12 (1.00-1.26), suggesting that majority of the attenuation of signal was not due to adjustment of BMI but rather due to selection based on non–missing data for BMI (Figures 3 and 4). This phenomenon was observed across all diabetes exposures and all case–control sets (with or without imaging requirement). It was particularly consequential for T1D and rotator cuff tear in the imaging-required case–control set where the odds ratios went from 0.90 (0.70-1.14) in the unadjusted full-sample analytic set to 0.76 (0.59-0.98) in the unadjusted complete case analysis analytic set, and to 0.75 (0.58-0.97) in analyses adjusted for BMI.

**Figure 4:**
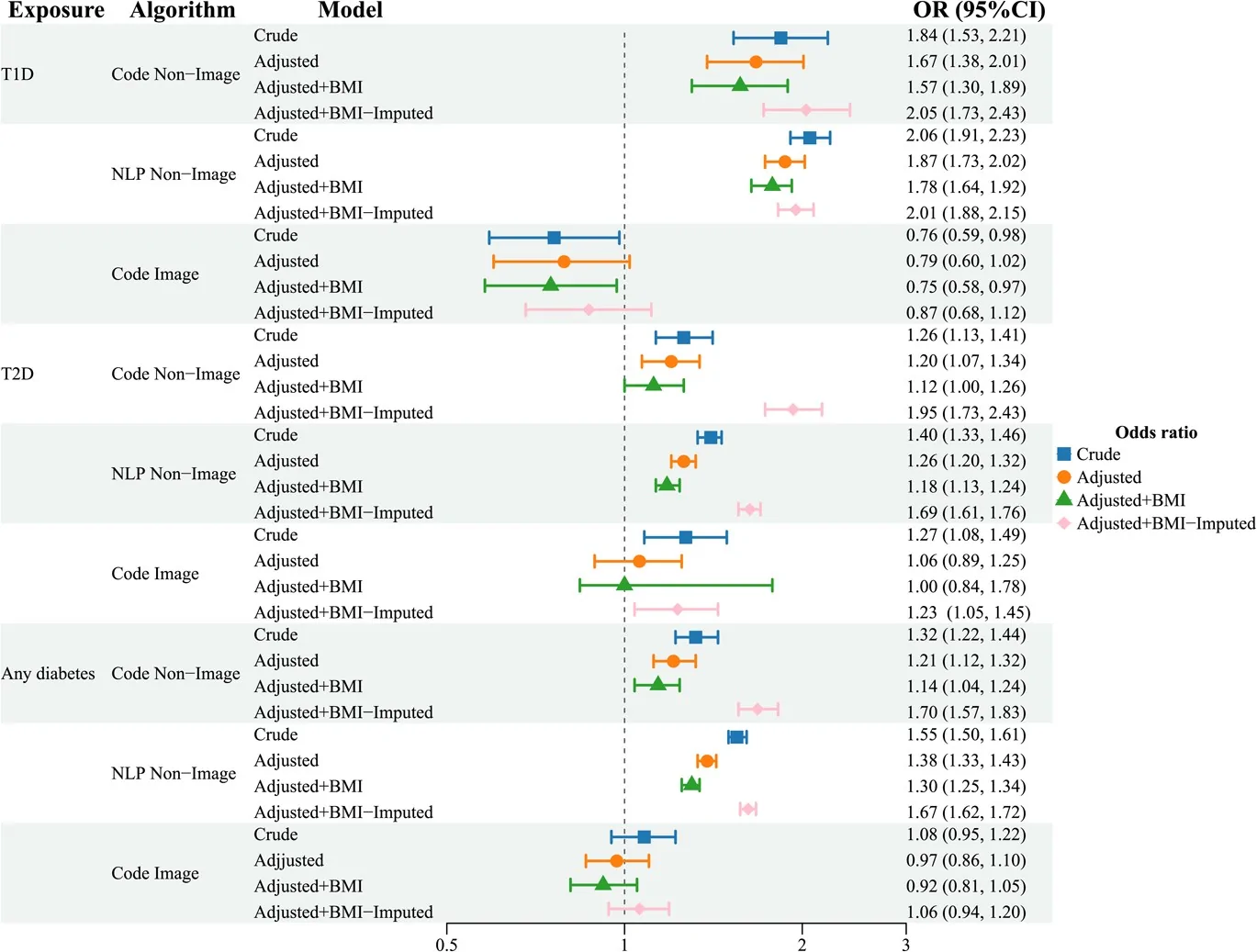
Forest plots showing association between diabetes exposures (type 1, type 2, and any diabetes) and rotator cuff tears in sample selected for nonmissing information on BMI [complete case] vs BMI-imputed full sample. Restricting analysis to case–control sets for whom data on BMI was missing led to a substantial attenuation of ORs for all exposures in unadjusted models, compared to unadjusted models from the full-sample case–control sets presented in Figure 3. Further adjustment for BMI in this complete case analysis set only attenuated effect estimates marginally. Imputing datasets for missing information on BMI and running analyses on the full sample adjusted for BMI reduced the attenuation. Blue boxes (crude) represent sample odds ratios from univariate models restricted to samples with BMI information available. Orange circles (adjusted) represent odds ratios from multivariable models adjusted for age, sex, and race/ethnicity restricted to samples with BMI information available. Green triangles (adjusted + BMI) represent odds ratios from multivariable models adjusted for age, sex, race/ethnicity, and BMI, restricted to samples with BMI information available. Pink diamonds (adjusted + BMI imputed) represent odds ratios from multivariable models adjusted for age, sex, race/ethnicity, and BMI in full sample following multiple imputations.

This idea is further supported by comparing the proportion of diabetes exposures as we increase selection stringency into the study population going from full analytic set to restriction based on BMI availability to further restriction based on availability of genetic data. We observe an increase in proportions of T1D from the original image-required case–control study (T1D proportion: 3.8% in cases; 4.2% in controls) to the sample that excludes individuals without BMI data (T1D proportion: 4.8% in cases; 6.3% in controls) (Table 1, Figure 2, Tables S2 and S3). Imposing additional restrictions to require availability of genetic data further strengthened the inverse association between T1D and rotator cuff tear (OR = 0.60; 95% CI, 0.35-0.99; T1D proportion: 6% in cases; and 11% in controls) (Tables S4 and S5). The prevalence of T1D also increased in the non–image-based control group as each selection criteria is imposed (T1D prevalence: 1% in originally selected controls) (Table 1, Figure 2, Table S2); 2% after excluding individuals without information on BMI (Figure 2, Tables S2 and S3); and 5% after selecting based on genetic data availability (Tables S4 and S5); however, the overall positive association between T1D and rotator cuff tear is maintained across the case–control sets where imaging was not required (Figures 3 and 4, Table S5).

We then evaluated whether some of this selection due to analytic choices could be ameliorated by multiple imputation of missing BMI values (Figure 4 and Table S2). Across all diabetes exposures, performing multiple imputation to include the full sample while adjusting for BMI led to reversal of selection bias observed due to complete case analysis, and resulting odds ratios were similar to those models that did not adjust for BMI but included the full sample while adjusting for age, sex, and race/ethnicity.

## Discussion

Using common metabolic exposures and rotator cuff tears as motivating examples, we showcase how electronic health care records can be leveraged along with a priori epidemiologic assumptions to identify and enumerate sources of selection bias in case–control designs that otherwise often remain hypothetical. Using multiple control groups constructed under the presence or absence of a putative collider stratification bias, we showed how the requirement of imaging for controls led to a substantial overestimation of exposure distribution (particularly T1D) in controls than is observed in the general population, which led to an inverse association between T1D and rotator cuff tears. We further showed that this bias may be exacerbated by applying selection pressures to study samples based on complete case analysis or by restricting to subsets of samples for whom specific biologic data may be available (eg, genetic data). Bias induced by analytic choices such as complete case analysis may be reversed with multiple imputation (assuming missingness at random); however, selection due to collider stratification or restriction due to sample availability are difficult to address once the choice is made.

We note that collider stratification bias induced by the requirement for imaging is exposure specific as it was observed for diabetes exposures with varying magnitudes but not for BMI. Diabetes exposures in imaging-required control groups showed the greatest deviation from population-based estimates. Our study suggests that the impact of the bias on inference may be more readily observed for exposures with low prevalence estimates in the general population such as T1D, where even a small absolute increase in exposure in controls (1% to 3%) leads to a substantially large increase in the relative scale (300%), yielding inverse associations. Diabetes is associated with several musculoskeletal disorders including adhesive capsulitis, Dupuytren’s contracture, carpal tunnel syndrome, osteoarthritis, and osteoarthropathy, among others.^24^ If controls received imaging for shoulder symptomatology and do not have rotator cuff tears, then it necessarily increases the prevalence of these other conditions into the control population and therefore increases prevalence of diabetes in this population as well, as we observe in our study. This also indicates that bias is not due to noncollapsibility of the odds ratio but due to an overestimation of the exposure distribution due to imaging requirement in controls. On the other hand, we find that prevalence of obesity based on WHO categories in controls was similar to estimates from the general population. It is possible that obesity is not as strongly associated or correlated with the conditions in the imaging-confirmed cases as diabetes.

In this study, while we provide evidence for selection bias due to restrictive strategies such as requirement of imaging for the choice of controls, this does not necessarily mean that the positive association between diabetes and rotator cuff tear observed in the no-imaging-required case–control sets is causal. Several variables, such as occupation, socio-economic status, and smoking history, were not reliably attainable from the VUMC SD without use of complex variable coding (ie, NLP) and could therefore not be adjusted for in these models. Our goal was primarily to explicate and provide evidence for putative mechanisms of bias.

Many examples of selection bias induced by restricting on a collider in extant literature are based on hypothetical scenarios^17-21^ or simulations (which are useful and necessary for demonstration), and although few empirical demonstrations from real data exist,^25-27^ to our knowledge, most depictions of selection bias in the context of modern EHRs are descriptive or theoretical (informative nonetheless).^28-30^ The work presented here not only theorizes a possible selection bias mechanism due to restriction on a descendant of a collider, but also shows empirical evidence of selection bias using data from a modern EHR system. The article further highlights the use of multiple control types^15^ and encourages its usage in EHR-based research, which, if juxtaposed along with knowledge of population distribution of exposure of interest, can be used to test theorized mechanisms regarding a putative selection bias to inform robust study design. The approach demonstrated here may be applicable across musculoskeletal, cardiovascular, reproductive, and any conditions where selection may be based on imaging or some other modality of testing, which is not universal, but indicated by symptomatology. These results further have substantial implications for molecular and genetic studies that are further selected via biobanks that may be linked to a larger EHR system. As shown in our study, the combination of collider stratification bias and further selection based on sample availability can substantially exacerbate bias, potentially also impacting associations between molecular and genetic signals associated with outcome of interest. On a more practical note, restrictions on imaging in this demonstration led to a biased control sample, did not provide substantial gains in predictive values,^9^ and also led to the smallest sample size for both cases and controls, which is often a strong consideration for genetic studies that are reliant on large sample sizes. The choice remains problematic even if restriction leads to improvement in predictive values but introduces collider bias. An alternate approach is to use imaging-required cases along with no-imaging-required controls as a potential strategy to improve PPV in cases while reducing chances of collider bias. Finally, image requirement in controls chosen from secondary data may not universally lead to bias, particularly for conditions screened for in routine medical care, such as vision^31^ and kidney^32^ tests for diabetics. However, here, care must be taken in evaluating differences between controls compliant in routine care compared to those who may not be.

Electronic health records and linked biobanks are an increasingly important part of today’s research infrastructure, and leveraging these to advance research comes with challenges that require processing large volumes of data and understanding of problems related to random and systematic patterns of data availability. Access to previously unprecedented volume of data also presents opportunities to apply approaches such as multiple controls with relative ease and cost efficiency to understand sources of biases (which previously remained theoretical), improve study designs and improve inferences borne from studies derived from these resources.

## Supplementary material

Supplementary material is available at the *American Journal of Epidemiology* online.

## Consent to participate

Retrospective and prospective data were collected for patients who agreed to participate and signed informed consent. There were no additional interventions or treatments offered to patients in this study.

## Consent to publish

The authors affirm that human research participants provided informed consent for publication of the works presented in this manuscript.

## Supplementary Material

Web_Material_kwag117

## Data Availability

All data pertaining to this publication is available upon request and approval.
