# Supplementary material for: Leveraging electronic health record data to evidence collider stratification bias and inform clinical epidemiology: obesity, diabetes, and rotator cuff tears
Source: Am J Epidemiol. 2026 Jun 1;195(10):2592–601. doi: 10.1093/aje/kwag117 (PMC13642364; doi:10.1093/aje/kwag117)
Supplement: Supplementary file 1 [file web_material_kwag117.zip › Supplemental File 1 Code.html]

RCT\_AG


Code 

- Show All Code
- Hide All Code

# RCT\_AG

#### Wenting Liu

#### 2026-03-19

# Import

```
# get all of variables that are used in the table 
all_vars <- c("rct_status", "sex", "age", "race_eth", "bmi", "bmi_cat", "t1d", "t2d", "dm_any", "hb_a1c_diag_numeric", "hpt_at_1st_diag_code", "hpt_at_1st_diag_meds", "ligamentous_disease", "prior_injury", "arthritis", "distinct_visit_cnt", "time_between_bmi_before")

# which ones are categorical and which are continuous
cat_vars <- c("rct_status", "sex", "race_eth", "bmi_cat", "t1d", "t2d", "dm_any", "hpt_at_1st_diag_code", "hpt_at_1st_diag_meds", "ligamentous_disease", "prior_injury", "arthritis")
cont_vars <- c("age", "bmi", "hb_a1c_diag_numeric", "distinct_visit_cnt", "time_between_bmi_before")
```

```
#setwd("/Users/simoneherzberg/Desktop/Simone/Graduate school/Rotator cuff /case control study/SD Data post R processing 1.11.24")
# setwd("/Users/ayushgiri/Library/CloudStorage/OneDrive-VUMC/BoxMigrationContent/Fac_life/Rotator_Cuff_Genetics/Simones_proposal_work/dissertation_datasets/dissertation_demographic/case_control_data")
tmp_nlp_df <- import("nlp.txt") %>%
    clean_names() %>%
    as_tibble()  %>% 
    dplyr::select(all_of(all_vars))
```

```
#setwd("/Users/simoneherzberg/Desktop/Simone/Graduate school/Rotator cuff /case control study/SD Data post R processing 1.11.24")
# setwd("/Users/ayushgiri/Library/CloudStorage/OneDrive-VUMC/BoxMigrationContent/Fac_life/Rotator_Cuff_Genetics/Simones_proposal_work/dissertation_datasets/dissertation_demographic/case_control_data")
tmp_code_image_df <- import("code_image.txt") %>% 
    clean_names() %>%
    as_tibble()  %>% 
    dplyr::select(all_of(all_vars))
```

```
#setwd("/Users/simoneherzberg/Desktop/Simone/Graduate school/Rotator cuff /case control study/SD Data post R processing 1.11.24")
# setwd("/Users/ayushgiri/Library/CloudStorage/OneDrive-VUMC/BoxMigrationContent/Fac_life/Rotator_Cuff_Genetics/Simones_proposal_work/dissertation_datasets/dissertation_demographic/case_control_data")
tmp_code_non_image_df <- import("code_non_image.txt") %>% 
    clean_names() %>%
    as_tibble()  %>% 
    dplyr::select(all_of(all_vars))
```

# Labeling fn

```
# make function to define variable labels
set_my_var_labels <- function(df) {
  labelled::var_label(df) <- list(
    rct_status = "RCT Status",
    age = "Age",
    sex = "Sex",
    race_eth = "Race/Ethn",
    bmi = "BMI Continuous (kg / m2)",
    bmi_cat = "BMI Categorical (kg / m2)",
    t1d = "Type 1 Diabetes",
    t2d = "Type 2 Diabetes",
    dm_any = "Any Diabetes",
    hb_a1c_diag_numeric = "HbA1c (mmol/mol)",
    hpt_at_1st_diag_code = "HTN Code",
    hpt_at_1st_diag_meds = "HTN Meds",      
    ligamentous_disease = "Ligamentous Disease", 
    prior_injury = "Prior Injury",
    arthritis = "Arthritis",
    distinct_visit_cnt = "VUMC Utilization (avg days)"
  )
  return(df)
}
```

# Wrangle

```
# make function to clean the tables

wrangle_my_df <- function(df, alg_type = c("NLP", "Code")){
  wrangled_df <- df %>%
    mutate(across(c(rct_status, sex, race_eth, bmi_cat, t1d, t2d, dm_any, hpt_at_1st_diag_code, hpt_at_1st_diag_meds, ligamentous_disease, prior_injury, arthritis),
                  as.factor)) %>%
    mutate(across(c(t1d, t2d),
                  ~fct_recode(as.character(.), 
                             "Yes" = "1", 
                             "No" = "0"))) %>%
    mutate(across(c(hpt_at_1st_diag_code, hpt_at_1st_diag_meds, ligamentous_disease, prior_injury, arthritis),
                  ~fct_recode(as.character(.), 
                              "Yes" = "1")))  %>% 
    mutate(across(c(hpt_at_1st_diag_code, hpt_at_1st_diag_meds, ligamentous_disease, prior_injury, arthritis),
                  ~fct_na_value_to_level(as.character(.), level = "No")))  %>% 
    mutate(dm_any = as.character(dm_any)) %>%
    mutate(dm_any = fct_recode(dm_any, 
                               "Any" = "1", 
                               "None" = "0"))  %>% 
    mutate(race_eth = fct_recode(race_eth, 
                                "White Non-Hispanic" = "W_NH",
                                "Black Non-Hispanic" = "B_NH",
                                "Asian Non-Hispanic" = "A_NH",
                                "Hispanic" = "H",
                                "Other" = "OTHER"))  %>%
    mutate(bmi_cat = fct_recode(bmi_cat, 
                               "Underweight (<18.5)" = "underweight",
                               "Normal (18.5-24.9)" = "normal",
                               "Overweight (25-29.9)" = "overweight",
                               "Obese (>30)" = "obese"))  %>% 
    mutate(sex = fct_recode(sex, 
                            "Female" = "F",
                            "Male" = "M"))  %>% 
    mutate(rct_status = fct_recode(as.character(rct_status), 
                                   "Case" = "1",
                                   "Control" = "0"))  %>% 
    mutate(rct_status = fct_relabel(rct_status, 
                                    ~paste0(.x, " (", alg_type, ")")
    ))  
  return(wrangled_df)
}
```

```
# Clean the tables
nlp_df <- wrangle_my_df(df = tmp_nlp_df, alg_type = "NLP")
```

```
# Clean the tables
code_image_df <- wrangle_my_df(df = tmp_code_image_df, alg_type = "Code Image")
```

```
# Clean the tables
code_non_image_df <- wrangle_my_df(df = tmp_code_non_image_df, alg_type = "Code Non Image")
```

# nlp\_df: Multiple Imputation for bmi

- outcome: rct\_status (included in MI), ref: control
- do not include: distinct\_visit\_cnt, bmi\_cat (derivative of bmi)
- set imputed dataset m to be at least the percentage of missing
  cases.

## abnormal bmi and bmi\_cat

```
library(tidyverse)

mismatched_bmi <- nlp_df %>%
  # create a new column with the CORRECT category based on the bmi value
  mutate(
    correct_bmi_cat = case_when(
      bmi < 18.5          ~ "Underweight (<18.5)",
      bmi >= 18.5 & bmi < 25 ~ "Normal (18.5-24.9)",
      bmi >= 25   & bmi < 30 ~ "Overweight (25-29.9)",
      bmi >= 30           ~ "Obese (>30)",
      TRUE                ~ NA_character_
    )
  ) %>%
  # filter for rows that meet any of error conditions
  filter(
    # Condition 1: The original category doesn't match the correct one
    (bmi_cat != correct_bmi_cat & !is.na(bmi)) |
    # Condition 2: The BMI value is biologically implausible
    (bmi > 150 | bmi < 10)
  ) %>%
  # Select relevant columns
  dplyr::select(rct_status, age, sex, bmi, bmi_cat, correct_bmi_cat)


print(mismatched_bmi)
```

```
## # A tibble: 157 � 6
##    rct_status      age sex      bmi bmi_cat              correct_bmi_cat     
##    <fct>         <dbl> <fct>  <dbl> <fct>                <chr>               
##  1 Control (NLP)  69.7 Male    30   Overweight (25-29.9) Obese (>30)         
##  2 Control (NLP)  77.0 Male    30   Overweight (25-29.9) Obese (>30)         
##  3 Control (NLP)  71.4 Female  25   Normal (18.5-24.9)   Overweight (25-29.9)
##  4 Case (NLP)     73.5 Female  25   Normal (18.5-24.9)   Overweight (25-29.9)
##  5 Control (NLP)  63.4 Female  18.5 Underweight (<18.5)  Normal (18.5-24.9)  
##  6 Case (NLP)     48.6 Male    25   Normal (18.5-24.9)   Overweight (25-29.9)
##  7 Control (NLP)  49.4 Male   197.  Normal (18.5-24.9)   Obese (>30)         
##  8 Case (NLP)     58.0 Male    25   Normal (18.5-24.9)   Overweight (25-29.9)
##  9 Case (NLP)     66.1 Male    25   Normal (18.5-24.9)   Overweight (25-29.9)
## 10 Case (NLP)     53.4 Female  30   Overweight (25-29.9) Obese (>30)         
## # ? 147 more rows
```

```
# Get a count of how many mismatches were found
cat(glue::glue("\nFound {nrow(mismatched_bmi)} rows with BMI mismatches or implausible values."))
```

```
## Found 157 rows with BMI mismatches or implausible values.
```

```
# Set implausible BMI values to NA
nlp_df <- nlp_df %>%
  mutate(
    bmi = if_else(bmi > 150 | bmi < 10, NA_real_, bmi)
  )
```

- Biologically implausible BMI values (bmi >150 or <10) were
  recoded as missing (NA) to be properly handled during the multiple
  imputation process.
- note: The BMI categories may need redefine to align with CDC/WHO
  guidelines. Specifically, boundary values are now included in the higher
  category; for example, a BMI of 30.0 is classified as �Obese (?30)�
  rather than �Overweight�; a BMI of 25.0 is classified as �Overweight
  (25-29.9)� rather than �Normal (18.5-24.9)�.

## check missingness in bmi

```
library(tidyverse)
library(naniar)

# str(nlp_df)
miss_var_summary(nlp_df)
```

```
## # A tibble: 17 � 3
##    variable                n_miss pct_miss
##    <chr>                    <int>    <num>
##  1 hb_a1c_diag_numeric     253840   92.6  
##  2 bmi                     167873   61.2  
##  3 bmi_cat                 167869   61.2  
##  4 time_between_bmi_before 157393   57.4  
##  5 t1d                      19653    7.17 
##  6 t2d                      13124    4.79 
##  7 distinct_visit_cnt         801    0.292
##  8 rct_status                   0    0    
##  9 sex                          0    0    
## 10 age                          0    0    
## 11 race_eth                     0    0    
## 12 dm_any                       0    0    
## 13 hpt_at_1st_diag_code         0    0    
## 14 hpt_at_1st_diag_meds         0    0    
## 15 ligamentous_disease          0    0    
## 16 prior_injury                 0    0    
## 17 arthritis                    0    0
```

```
# Set "Control (NLP)" as the reference level for the outcome variable
nlp_df <- nlp_df %>%
  mutate(rct_status = fct_relevel(rct_status, "Control (NLP)"))
```

## imputation

- �distinct\_visit\_cnt�, �bmi\_cat� are not used in MI.
- DO INCLUDE OUTCOME rct\_status in imputation, but ensured rct\_status
  is treated as a predictor but not imputed.

```
library(mice)

# Select the variables to use for imputation
vars_for_imputation <- setdiff(names(nlp_df), c("distinct_visit_cnt", "bmi_cat"))

# Keep only those variables in the data to be imputed
nlp_impute_data <- nlp_df %>%
  dplyr::select(all_of(vars_for_imputation))

# Configure mice to NOT impute the outcome
meth <- make.method(nlp_impute_data)

# Ensure rct_status is used as a predictor but has no imputation method assigned
meth["rct_status"] <- ""
pred <- make.predictorMatrix(nlp_impute_data)


# 3. Run the MICE algorithm
set.seed(123) 
nlp_imputed <- mice(nlp_impute_data, 
                    method = meth, 
                    predictorMatrix = pred,
                    m = 65, 
                    maxit = 10, # Increased maxit to 10 for better convergence
                    printFlag = TRUE)
```

```
## 
##  iter imp variable
##   1   1  bmi  hb_a1c_diag_numeric  time_between_bmi_before
##   1   2  bmi  hb_a1c_diag_numeric  time_between_bmi_before
##   1   3  bmi  hb_a1c_diag_numeric  time_between_bmi_before
##   1   4  bmi  hb_a1c_diag_numeric  time_between_bmi_before
##   1   5  bmi  hb_a1c_diag_numeric  time_between_bmi_before
##   1   6  bmi  hb_a1c_diag_numeric  time_between_bmi_before
##   1   7  bmi  hb_a1c_diag_numeric  time_between_bmi_before
##   1   8  bmi  hb_a1c_diag_numeric  time_between_bmi_before
##   1   9  bmi  hb_a1c_diag_numeric  time_between_bmi_before
##   1   10  bmi  hb_a1c_diag_numeric  time_between_bmi_before
##   1   11  bmi  hb_a1c_diag_numeric  time_between_bmi_before
##   1   12  bmi  hb_a1c_diag_numeric  time_between_bmi_before
##   1   13  bmi  hb_a1c_diag_numeric  time_between_bmi_before
##   1   14  bmi  hb_a1c_diag_numeric  time_between_bmi_before
##   1   15  bmi  hb_a1c_diag_numeric  time_between_bmi_before
##   1   16  bmi  hb_a1c_diag_numeric  time_between_bmi_before
##   1   17  bmi  hb_a1c_diag_numeric  time_between_bmi_before
##   1   18  bmi  hb_a1c_diag_numeric  time_between_bmi_before
##   1   19  bmi  hb_a1c_diag_numeric  time_between_bmi_before
##   1   20  bmi  hb_a1c_diag_numeric  time_between_bmi_before
##   1   21  bmi  hb_a1c_diag_numeric  time_between_bmi_before
##   1   22  bmi  hb_a1c_diag_numeric  time_between_bmi_before
##   1   23  bmi  hb_a1c_diag_numeric  time_between_bmi_before
##   1   24  bmi  hb_a1c_diag_numeric  time_between_bmi_before
##   1   25  bmi  hb_a1c_diag_numeric  time_between_bmi_before
##   1   26  bmi  hb_a1c_diag_numeric  time_between_bmi_before
##   1   27  bmi  hb_a1c_diag_numeric  time_between_bmi_before
##   1   28  bmi  hb_a1c_diag_numeric  time_between_bmi_before
##   1   29  bmi  hb_a1c_diag_numeric  time_between_bmi_before
##   1   30  bmi  hb_a1c_diag_numeric  time_between_bmi_before
##   1   31  bmi  hb_a1c_diag_numeric  time_between_bmi_before
##   1   32  bmi  hb_a1c_diag_numeric  time_between_bmi_before
##   1   33  bmi  hb_a1c_diag_numeric  time_between_bmi_before
##   1   34  bmi  hb_a1c_diag_numeric  time_between_bmi_before
##   1   35  bmi  hb_a1c_diag_numeric  time_between_bmi_before
##   1   36  bmi  hb_a1c_diag_numeric  time_between_bmi_before
##   1   37  bmi  hb_a1c_diag_numeric  time_between_bmi_before
##   1   38  bmi  hb_a1c_diag_numeric  time_between_bmi_before
##   1   39  bmi  hb_a1c_diag_numeric  time_between_bmi_before
##   1   40  bmi  hb_a1c_diag_numeric  time_between_bmi_before
##   1   41  bmi  hb_a1c_diag_numeric  time_between_bmi_before
##   1   42  bmi  hb_a1c_diag_numeric  time_between_bmi_before
##   1   43  bmi  hb_a1c_diag_numeric  time_between_bmi_before
##   1   44  bmi  hb_a1c_diag_numeric  time_between_bmi_before
##   1   45  bmi  hb_a1c_diag_numeric  time_between_bmi_before
##   1   46  bmi  hb_a1c_diag_numeric  time_between_bmi_before
##   1   47  bmi  hb_a1c_diag_numeric  time_between_bmi_before
##   1   48  bmi  hb_a1c_diag_numeric  time_between_bmi_before
##   1   49  bmi  hb_a1c_diag_numeric  time_between_bmi_before
##   1   50  bmi  hb_a1c_diag_numeric  time_between_bmi_before
##   1   51  bmi  hb_a1c_diag_numeric  time_between_bmi_before
##   1   52  bmi  hb_a1c_diag_numeric  time_between_bmi_before
##   1   53  bmi  hb_a1c_diag_numeric  time_between_bmi_before
##   1   54  bmi  hb_a1c_diag_numeric  time_between_bmi_before
##   1   55  bmi  hb_a1c_diag_numeric  time_between_bmi_before
##   1   56  bmi  hb_a1c_diag_numeric  time_between_bmi_before
##   1   57  bmi  hb_a1c_diag_numeric  time_between_bmi_before
##   1   58  bmi  hb_a1c_diag_numeric  time_between_bmi_before
##   1   59  bmi  hb_a1c_diag_numeric  time_between_bmi_before
##   1   60  bmi  hb_a1c_diag_numeric  time_between_bmi_before
##   1   61  bmi  hb_a1c_diag_numeric  time_between_bmi_before
##   1   62  bmi  hb_a1c_diag_numeric  time_between_bmi_before
##   1   63  bmi  hb_a1c_diag_numeric  time_between_bmi_before
##   1   64  bmi  hb_a1c_diag_numeric  time_between_bmi_before
##   1   65  bmi  hb_a1c_diag_numeric  time_between_bmi_before
##   2   1  bmi  hb_a1c_diag_numeric  time_between_bmi_before
##   2   2  bmi  hb_a1c_diag_numeric  time_between_bmi_before
##   2   3  bmi  hb_a1c_diag_numeric  time_between_bmi_before
##   2   4  bmi  hb_a1c_diag_numeric  time_between_bmi_before
##   2   5  bmi  hb_a1c_diag_numeric  time_between_bmi_before
##   2   6  bmi  hb_a1c_diag_numeric  time_between_bmi_before
##   2   7  bmi  hb_a1c_diag_numeric  time_between_bmi_before
##   2   8  bmi  hb_a1c_diag_numeric  time_between_bmi_before
##   2   9  bmi  hb_a1c_diag_numeric  time_between_bmi_before
##   2   10  bmi  hb_a1c_diag_numeric  time_between_bmi_before
##   2   11  bmi  hb_a1c_diag_numeric  time_between_bmi_before
##   2   12  bmi  hb_a1c_diag_numeric  time_between_bmi_before
##   2   13  bmi  hb_a1c_diag_numeric  time_between_bmi_before
##   2   14  bmi  hb_a1c_diag_numeric  time_between_bmi_before
##   2   15  bmi  hb_a1c_diag_numeric  time_between_bmi_before
##   2   16  bmi  hb_a1c_diag_numeric  time_between_bmi_before
##   2   17  bmi  hb_a1c_diag_numeric  time_between_bmi_before
##   2   18  bmi  hb_a1c_diag_numeric  time_between_bmi_before
##   2   19  bmi  hb_a1c_diag_numeric  time_between_bmi_before
##   2   20  bmi  hb_a1c_diag_numeric  time_between_bmi_before
##   2   21  bmi  hb_a1c_diag_numeric  time_between_bmi_before
##   2   22  bmi  hb_a1c_diag_numeric  time_between_bmi_before
##   2   23  bmi  hb_a1c_diag_numeric  time_between_bmi_before
##   2   24  bmi  hb_a1c_diag_numeric  time_between_bmi_before
##   2   25  bmi  hb_a1c_diag_numeric  time_between_bmi_before
##   2   26  bmi  hb_a1c_diag_numeric  time_between_bmi_before
##   2   27  bmi  hb_a1c_diag_numeric  time_between_bmi_before
##   2   28  bmi  hb_a1c_diag_numeric  time_between_bmi_before
##   2   29  bmi  hb_a1c_diag_numeric  time_between_bmi_before
##   2   30  bmi  hb_a1c_diag_numeric  time_between_bmi_before
##   2   31  bmi  hb_a1c_diag_numeric  time_between_bmi_before
##   2   32  bmi  hb_a1c_diag_numeric  time_between_bmi_before
##   2   33  bmi  hb_a1c_diag_numeric  time_between_bmi_before
##   2   34  bmi  hb_a1c_diag_numeric  time_between_bmi_before
##   2   35  bmi  hb_a1c_diag_numeric  time_between_bmi_before
##   2   36  bmi  hb_a1c_diag_numeric  time_between_bmi_before
##   2   37  bmi  hb_a1c_diag_numeric  time_between_bmi_before
##   2   38  bmi  hb_a1c_diag_numeric  time_between_bmi_before
##   2   39  bmi  hb_a1c_diag_numeric  time_between_bmi_before
##   2   40  bmi  hb_a1c_diag_numeric  time_between_bmi_before
##   2   41  bmi  hb_a1c_diag_numeric  time_between_bmi_before
##   2   42  bmi  hb_a1c_diag_numeric  time_between_bmi_before
##   2   43  bmi  hb_a1c_diag_numeric  time_between_bmi_before
##   2   44  bmi  hb_a1c_diag_numeric  time_between_bmi_before
##   2   45  bmi  hb_a1c_diag_numeric  time_between_bmi_before
##   2   46  bmi  hb_a1c_diag_numeric  time_between_bmi_before
##   2   47  bmi  hb_a1c_diag_numeric  time_between_bmi_before
##   2   48  bmi  hb_a1c_diag_numeric  time_between_bmi_before
##   2   49  bmi  hb_a1c_diag_numeric  time_between_bmi_before
##   2   50  bmi  hb_a1c_diag_numeric  time_between_bmi_before
##   2   51  bmi  hb_a1c_diag_numeric  time_between_bmi_before
##   2   52  bmi  hb_a1c_diag_numeric  time_between_bmi_before
##   2   53  bmi  hb_a1c_diag_numeric  time_between_bmi_before
##   2   54  bmi  hb_a1c_diag_numeric  time_between_bmi_before
##   2   55  bmi  hb_a1c_diag_numeric  time_between_bmi_before
##   2   56  bmi  hb_a1c_diag_numeric  time_between_bmi_before
##   2   57  bmi  hb_a1c_diag_numeric  time_between_bmi_before
##   2   58  bmi  hb_a1c_diag_numeric  time_between_bmi_before
##   2   59  bmi  hb_a1c_diag_numeric  time_between_bmi_before
##   2   60  bmi  hb_a1c_diag_numeric  time_between_bmi_before
##   2   61  bmi  hb_a1c_diag_numeric  time_between_bmi_before
##   2   62  bmi  hb_a1c_diag_numeric  time_between_bmi_before
##   2   63  bmi  hb_a1c_diag_numeric  time_between_bmi_before
##   2   64  bmi  hb_a1c_diag_numeric  time_between_bmi_before
##   2   65  bmi  hb_a1c_diag_numeric  time_between_bmi_before
##   3   1  bmi  hb_a1c_diag_numeric  time_between_bmi_before
##   3   2  bmi  hb_a1c_diag_numeric  time_between_bmi_before
##   3   3  bmi  hb_a1c_diag_numeric  time_between_bmi_before
##   3   4  bmi  hb_a1c_diag_numeric  time_between_bmi_before
##   3   5  bmi  hb_a1c_diag_numeric  time_between_bmi_before
##   3   6  bmi  hb_a1c_diag_numeric  time_between_bmi_before
##   3   7  bmi  hb_a1c_diag_numeric  time_between_bmi_before
##   3   8  bmi  hb_a1c_diag_numeric  time_between_bmi_before
##   3   9  bmi  hb_a1c_diag_numeric  time_between_bmi_before
##   3   10  bmi  hb_a1c_diag_numeric  time_between_bmi_before
##   3   11  bmi  hb_a1c_diag_numeric  time_between_bmi_before
##   3   12  bmi  hb_a1c_diag_numeric  time_between_bmi_before
##   3   13  bmi  hb_a1c_diag_numeric  time_between_bmi_before
##   3   14  bmi  hb_a1c_diag_numeric  time_between_bmi_before
##   3   15  bmi  hb_a1c_diag_numeric  time_between_bmi_before
##   3   16  bmi  hb_a1c_diag_numeric  time_between_bmi_before
##   3   17  bmi  hb_a1c_diag_numeric  time_between_bmi_before
##   3   18  bmi  hb_a1c_diag_numeric  time_between_bmi_before
##   3   19  bmi  hb_a1c_diag_numeric  time_between_bmi_before
##   3   20  bmi  hb_a1c_diag_numeric  time_between_bmi_before
##   3   21  bmi  hb_a1c_diag_numeric  time_between_bmi_before
##   3   22  bmi  hb_a1c_diag_numeric  time_between_bmi_before
##   3   23  bmi  hb_a1c_diag_numeric  time_between_bmi_before
##   3   24  bmi  hb_a1c_diag_numeric  time_between_bmi_before
##   3   25  bmi  hb_a1c_diag_numeric  time_between_bmi_before
##   3   26  bmi  hb_a1c_diag_numeric  time_between_bmi_before
##   3   27  bmi  hb_a1c_diag_numeric  time_between_bmi_before
##   3   28  bmi  hb_a1c_diag_numeric  time_between_bmi_before
##   3   29  bmi  hb_a1c_diag_numeric  time_between_bmi_before
##   3   30  bmi  hb_a1c_diag_numeric  time_between_bmi_before
##   3   31  bmi  hb_a1c_diag_numeric  time_between_bmi_before
##   3   32  bmi  hb_a1c_diag_numeric  time_between_bmi_before
##   3   33  bmi  hb_a1c_diag_numeric  time_between_bmi_before
##   3   34  bmi  hb_a1c_diag_numeric  time_between_bmi_before
##   3   35  bmi  hb_a1c_diag_numeric  time_between_bmi_before
##   3   36  bmi  hb_a1c_diag_numeric  time_between_bmi_before
##   3   37  bmi  hb_a1c_diag_numeric  time_between_bmi_before
##   3   38  bmi  hb_a1c_diag_numeric  time_between_bmi_before
##   3   39  bmi  hb_a1c_diag_numeric  time_between_bmi_before
##   3   40  bmi  hb_a1c_diag_numeric  time_between_bmi_before
##   3   41  bmi  hb_a1c_diag_numeric  time_between_bmi_before
##   3   42  bmi  hb_a1c_diag_numeric  time_between_bmi_before
##   3   43  bmi  hb_a1c_diag_numeric  time_between_bmi_before
##   3   44  bmi  hb_a1c_diag_numeric  time_between_bmi_before
##   3   45  bmi  hb_a1c_diag_numeric  time_between_bmi_before
##   3   46  bmi  hb_a1c_diag_numeric  time_between_bmi_before
##   3   47  bmi  hb_a1c_diag_numeric  time_between_bmi_before
##   3   48  bmi  hb_a1c_diag_numeric  time_between_bmi_before
##   3   49  bmi  hb_a1c_diag_numeric  time_between_bmi_before
##   3   50  bmi  hb_a1c_diag_numeric  time_between_bmi_before
##   3   51  bmi  hb_a1c_diag_numeric  time_between_bmi_before
##   3   52  bmi  hb_a1c_diag_numeric  time_between_bmi_before
##   3   53  bmi  hb_a1c_diag_numeric  time_between_bmi_before
##   3   54  bmi  hb_a1c_diag_numeric  time_between_bmi_before
##   3   55  bmi  hb_a1c_diag_numeric  time_between_bmi_before
##   3   56  bmi  hb_a1c_diag_numeric  time_between_bmi_before
##   3   57  bmi  hb_a1c_diag_numeric  time_between_bmi_before
##   3   58  bmi  hb_a1c_diag_numeric  time_between_bmi_before
##   3   59  bmi  hb_a1c_diag_numeric  time_between_bmi_before
##   3   60  bmi  hb_a1c_diag_numeric  time_between_bmi_before
##   3   61  bmi  hb_a1c_diag_numeric  time_between_bmi_before
##   3   62  bmi  hb_a1c_diag_numeric  time_between_bmi_before
##   3   63  bmi  hb_a1c_diag_numeric  time_between_bmi_before
##   3   64  bmi  hb_a1c_diag_numeric  time_between_bmi_before
##   3   65  bmi  hb_a1c_diag_numeric  time_between_bmi_before
##   4   1  bmi  hb_a1c_diag_numeric  time_between_bmi_before
##   4   2  bmi  hb_a1c_diag_numeric  time_between_bmi_before
##   4   3  bmi  hb_a1c_diag_numeric  time_between_bmi_before
##   4   4  bmi  hb_a1c_diag_numeric  time_between_bmi_before
##   4   5  bmi  hb_a1c_diag_numeric  time_between_bmi_before
##   4   6  bmi  hb_a1c_diag_numeric  time_between_bmi_before
##   4   7  bmi  hb_a1c_diag_numeric  time_between_bmi_before
##   4   8  bmi  hb_a1c_diag_numeric  time_between_bmi_before
##   4   9  bmi  hb_a1c_diag_numeric  time_between_bmi_before
##   4   10  bmi  hb_a1c_diag_numeric  time_between_bmi_before
##   4   11  bmi  hb_a1c_diag_numeric  time_between_bmi_before
##   4   12  bmi  hb_a1c_diag_numeric  time_between_bmi_before
##   4   13  bmi  hb_a1c_diag_numeric  time_between_bmi_before
##   4   14  bmi  hb_a1c_diag_numeric  time_between_bmi_before
##   4   15  bmi  hb_a1c_diag_numeric  time_between_bmi_before
##   4   16  bmi  hb_a1c_diag_numeric  time_between_bmi_before
##   4   17  bmi  hb_a1c_diag_numeric  time_between_bmi_before
##   4   18  bmi  hb_a1c_diag_numeric  time_between_bmi_before
##   4   19  bmi  hb_a1c_diag_numeric  time_between_bmi_before
##   4   20  bmi  hb_a1c_diag_numeric  time_between_bmi_before
##   4   21  bmi  hb_a1c_diag_numeric  time_between_bmi_before
##   4   22  bmi  hb_a1c_diag_numeric  time_between_bmi_before
##   4   23  bmi  hb_a1c_diag_numeric  time_between_bmi_before
##   4   24  bmi  hb_a1c_diag_numeric  time_between_bmi_before
##   4   25  bmi  hb_a1c_diag_numeric  time_between_bmi_before
##   4   26  bmi  hb_a1c_diag_numeric  time_between_bmi_before
##   4   27  bmi  hb_a1c_diag_numeric  time_between_bmi_before
##   4   28  bmi  hb_a1c_diag_numeric  time_between_bmi_before
##   4   29  bmi  hb_a1c_diag_numeric  time_between_bmi_before
##   4   30  bmi  hb_a1c_diag_numeric  time_between_bmi_before
##   4   31  bmi  hb_a1c_diag_numeric  time_between_bmi_before
##   4   32  bmi  hb_a1c_diag_numeric  time_between_bmi_before
##   4   33  bmi  hb_a1c_diag_numeric  time_between_bmi_before
##   4   34  bmi  hb_a1c_diag_numeric  time_between_bmi_before
##   4   35  bmi  hb_a1c_diag_numeric  time_between_bmi_before
##   4   36  bmi  hb_a1c_diag_numeric  time_between_bmi_before
##   4   37  bmi  hb_a1c_diag_numeric  time_between_bmi_before
##   4   38  bmi  hb_a1c_diag_numeric  time_between_bmi_before
##   4   39  bmi  hb_a1c_diag_numeric  time_between_bmi_before
##   4   40  bmi  hb_a1c_diag_numeric  time_between_bmi_before
##   4   41  bmi  hb_a1c_diag_numeric  time_between_bmi_before
##   4   42  bmi  hb_a1c_diag_numeric  time_between_bmi_before
##   4   43  bmi  hb_a1c_diag_numeric  time_between_bmi_before
##   4   44  bmi  hb_a1c_diag_numeric  time_between_bmi_before
##   4   45  bmi  hb_a1c_diag_numeric  time_between_bmi_before
##   4   46  bmi  hb_a1c_diag_numeric  time_between_bmi_before
##   4   47  bmi  hb_a1c_diag_numeric  time_between_bmi_before
##   4   48  bmi  hb_a1c_diag_numeric  time_between_bmi_before
##   4   49  bmi  hb_a1c_diag_numeric  time_between_bmi_before
##   4   50  bmi  hb_a1c_diag_numeric  time_between_bmi_before
##   4   51  bmi  hb_a1c_diag_numeric  time_between_bmi_before
##   4   52  bmi  hb_a1c_diag_numeric  time_between_bmi_before
##   4   53  bmi  hb_a1c_diag_numeric  time_between_bmi_before
##   4   54  bmi  hb_a1c_diag_numeric  time_between_bmi_before
##   4   55  bmi  hb_a1c_diag_numeric  time_between_bmi_before
##   4   56  bmi  hb_a1c_diag_numeric  time_between_bmi_before
##   4   57  bmi  hb_a1c_diag_numeric  time_between_bmi_before
##   4   58  bmi  hb_a1c_diag_numeric  time_between_bmi_before
##   4   59  bmi  hb_a1c_diag_numeric  time_between_bmi_before
##   4   60  bmi  hb_a1c_diag_numeric  time_between_bmi_before
##   4   61  bmi  hb_a1c_diag_numeric  time_between_bmi_before
##   4   62  bmi  hb_a1c_diag_numeric  time_between_bmi_before
##   4   63  bmi  hb_a1c_diag_numeric  time_between_bmi_before
##   4   64  bmi  hb_a1c_diag_numeric  time_between_bmi_before
##   4   65  bmi  hb_a1c_diag_numeric  time_between_bmi_before
##   5   1  bmi  hb_a1c_diag_numeric  time_between_bmi_before
##   5   2  bmi  hb_a1c_diag_numeric  time_between_bmi_before
##   5   3  bmi  hb_a1c_diag_numeric  time_between_bmi_before
##   5   4  bmi  hb_a1c_diag_numeric  time_between_bmi_before
##   5   5  bmi  hb_a1c_diag_numeric  time_between_bmi_before
##   5   6  bmi  hb_a1c_diag_numeric  time_between_bmi_before
##   5   7  bmi  hb_a1c_diag_numeric  time_between_bmi_before
##   5   8  bmi  hb_a1c_diag_numeric  time_between_bmi_before
##   5   9  bmi  hb_a1c_diag_numeric  time_between_bmi_before
##   5   10  bmi  hb_a1c_diag_numeric  time_between_bmi_before
##   5   11  bmi  hb_a1c_diag_numeric  time_between_bmi_before
##   5   12  bmi  hb_a1c_diag_numeric  time_between_bmi_before
##   5   13  bmi  hb_a1c_diag_numeric  time_between_bmi_before
##   5   14  bmi  hb_a1c_diag_numeric  time_between_bmi_before
##   5   15  bmi  hb_a1c_diag_numeric  time_between_bmi_before
##   5   16  bmi  hb_a1c_diag_numeric  time_between_bmi_before
##   5   17  bmi  hb_a1c_diag_numeric  time_between_bmi_before
##   5   18  bmi  hb_a1c_diag_numeric  time_between_bmi_before
##   5   19  bmi  hb_a1c_diag_numeric  time_between_bmi_before
##   5   20  bmi  hb_a1c_diag_numeric  time_between_bmi_before
##   5   21  bmi  hb_a1c_diag_numeric  time_between_bmi_before
##   5   22  bmi  hb_a1c_diag_numeric  time_between_bmi_before
##   5   23  bmi  hb_a1c_diag_numeric  time_between_bmi_before
##   5   24  bmi  hb_a1c_diag_numeric  time_between_bmi_before
##   5   25  bmi  hb_a1c_diag_numeric  time_between_bmi_before
##   5   26  bmi  hb_a1c_diag_numeric  time_between_bmi_before
##   5   27  bmi  hb_a1c_diag_numeric  time_between_bmi_before
##   5   28  bmi  hb_a1c_diag_numeric  time_between_bmi_before
##   5   29  bmi  hb_a1c_diag_numeric  time_between_bmi_before
##   5   30  bmi  hb_a1c_diag_numeric  time_between_bmi_before
##   5   31  bmi  hb_a1c_diag_numeric  time_between_bmi_before
##   5   32  bmi  hb_a1c_diag_numeric  time_between_bmi_before
##   5   33  bmi  hb_a1c_diag_numeric  time_between_bmi_before
##   5   34  bmi  hb_a1c_diag_numeric  time_between_bmi_before
##   5   35  bmi  hb_a1c_diag_numeric  time_between_bmi_before
##   5   36  bmi  hb_a1c_diag_numeric  time_between_bmi_before
##   5   37  bmi  hb_a1c_diag_numeric  time_between_bmi_before
##   5   38  bmi  hb_a1c_diag_numeric  time_between_bmi_before
##   5   39  bmi  hb_a1c_diag_numeric  time_between_bmi_before
##   5   40  bmi  hb_a1c_diag_numeric  time_between_bmi_before
##   5   41  bmi  hb_a1c_diag_numeric  time_between_bmi_before
##   5   42  bmi  hb_a1c_diag_numeric  time_between_bmi_before
##   5   43  bmi  hb_a1c_diag_numeric  time_between_bmi_before
##   5   44  bmi  hb_a1c_diag_numeric  time_between_bmi_before
##   5   45  bmi  hb_a1c_diag_numeric  time_between_bmi_before
##   5   46  bmi  hb_a1c_diag_numeric  time_between_bmi_before
##   5   47  bmi  hb_a1c_diag_numeric  time_between_bmi_before
##   5   48  bmi  hb_a1c_diag_numeric  time_between_bmi_before
##   5   49  bmi  hb_a1c_diag_numeric  time_between_bmi_before
##   5   50  bmi  hb_a1c_diag_numeric  time_between_bmi_before
##   5   51  bmi  hb_a1c_diag_numeric  time_between_bmi_before
##   5   52  bmi  hb_a1c_diag_numeric  time_between_bmi_before
##   5   53  bmi  hb_a1c_diag_numeric  time_between_bmi_before
##   5   54  bmi  hb_a1c_diag_numeric  time_between_bmi_before
##   5   55  bmi  hb_a1c_diag_numeric  time_between_bmi_before
##   5   56  bmi  hb_a1c_diag_numeric  time_between_bmi_before
##   5   57  bmi  hb_a1c_diag_numeric  time_between_bmi_before
##   5   58  bmi  hb_a1c_diag_numeric  time_between_bmi_before
##   5   59  bmi  hb_a1c_diag_numeric  time_between_bmi_before
##   5   60  bmi  hb_a1c_diag_numeric  time_between_bmi_before
##   5   61  bmi  hb_a1c_diag_numeric  time_between_bmi_before
##   5   62  bmi  hb_a1c_diag_numeric  time_between_bmi_before
##   5   63  bmi  hb_a1c_diag_numeric  time_between_bmi_before
##   5   64  bmi  hb_a1c_diag_numeric  time_between_bmi_before
##   5   65  bmi  hb_a1c_diag_numeric  time_between_bmi_before
##   6   1  bmi  hb_a1c_diag_numeric  time_between_bmi_before
##   6   2  bmi  hb_a1c_diag_numeric  time_between_bmi_before
##   6   3  bmi  hb_a1c_diag_numeric  time_between_bmi_before
##   6   4  bmi  hb_a1c_diag_numeric  time_between_bmi_before
##   6   5  bmi  hb_a1c_diag_numeric  time_between_bmi_before
##   6   6  bmi  hb_a1c_diag_numeric  time_between_bmi_before
##   6   7  bmi  hb_a1c_diag_numeric  time_between_bmi_before
##   6   8  bmi  hb_a1c_diag_numeric  time_between_bmi_before
##   6   9  bmi  hb_a1c_diag_numeric  time_between_bmi_before
##   6   10  bmi  hb_a1c_diag_numeric  time_between_bmi_before
##   6   11  bmi  hb_a1c_diag_numeric  time_between_bmi_before
##   6   12  bmi  hb_a1c_diag_numeric  time_between_bmi_before
##   6   13  bmi  hb_a1c_diag_numeric  time_between_bmi_before
##   6   14  bmi  hb_a1c_diag_numeric  time_between_bmi_before
##   6   15  bmi  hb_a1c_diag_numeric  time_between_bmi_before
##   6   16  bmi  hb_a1c_diag_numeric  time_between_bmi_before
##   6   17  bmi  hb_a1c_diag_numeric  time_between_bmi_before
##   6   18  bmi  hb_a1c_diag_numeric  time_between_bmi_before
##   6   19  bmi  hb_a1c_diag_numeric  time_between_bmi_before
##   6   20  bmi  hb_a1c_diag_numeric  time_between_bmi_before
##   6   21  bmi  hb_a1c_diag_numeric  time_between_bmi_before
##   6   22  bmi  hb_a1c_diag_numeric  time_between_bmi_before
##   6   23  bmi  hb_a1c_diag_numeric  time_between_bmi_before
##   6   24  bmi  hb_a1c_diag_numeric  time_between_bmi_before
##   6   25  bmi  hb_a1c_diag_numeric  time_between_bmi_before
##   6   26  bmi  hb_a1c_diag_numeric  time_between_bmi_before
##   6   27  bmi  hb_a1c_diag_numeric  time_between_bmi_before
##   6   28  bmi  hb_a1c_diag_numeric  time_between_bmi_before
##   6   29  bmi  hb_a1c_diag_numeric  time_between_bmi_before
##   6   30  bmi  hb_a1c_diag_numeric  time_between_bmi_before
##   6   31  bmi  hb_a1c_diag_numeric  time_between_bmi_before
##   6   32  bmi  hb_a1c_diag_numeric  time_between_bmi_before
##   6   33  bmi  hb_a1c_diag_numeric  time_between_bmi_before
##   6   34  bmi  hb_a1c_diag_numeric  time_between_bmi_before
##   6   35  bmi  hb_a1c_diag_numeric  time_between_bmi_before
##   6   36  bmi  hb_a1c_diag_numeric  time_between_bmi_before
##   6   37  bmi  hb_a1c_diag_numeric  time_between_bmi_before
##   6   38  bmi  hb_a1c_diag_numeric  time_between_bmi_before
##   6   39  bmi  hb_a1c_diag_numeric  time_between_bmi_before
##   6   40  bmi  hb_a1c_diag_numeric  time_between_bmi_before
##   6   41  bmi  hb_a1c_diag_numeric  time_between_bmi_before
##   6   42  bmi  hb_a1c_diag_numeric  time_between_bmi_before
##   6   43  bmi  hb_a1c_diag_numeric  time_between_bmi_before
##   6   44  bmi  hb_a1c_diag_numeric  time_between_bmi_before
##   6   45  bmi  hb_a1c_diag_numeric  time_between_bmi_before
##   6   46  bmi  hb_a1c_diag_numeric  time_between_bmi_before
##   6   47  bmi  hb_a1c_diag_numeric  time_between_bmi_before
##   6   48  bmi  hb_a1c_diag_numeric  time_between_bmi_before
##   6   49  bmi  hb_a1c_diag_numeric  time_between_bmi_before
##   6   50  bmi  hb_a1c_diag_numeric  time_between_bmi_before
##   6   51  bmi  hb_a1c_diag_numeric  time_between_bmi_before
##   6   52  bmi  hb_a1c_diag_numeric  time_between_bmi_before
##   6   53  bmi  hb_a1c_diag_numeric  time_between_bmi_before
##   6   54  bmi  hb_a1c_diag_numeric  time_between_bmi_before
##   6   55  bmi  hb_a1c_diag_numeric  time_between_bmi_before
##   6   56  bmi  hb_a1c_diag_numeric  time_between_bmi_before
##   6   57  bmi  hb_a1c_diag_numeric  time_between_bmi_before
##   6   58  bmi  hb_a1c_diag_numeric  time_between_bmi_before
##   6   59  bmi  hb_a1c_diag_numeric  time_between_bmi_before
##   6   60  bmi  hb_a1c_diag_numeric  time_between_bmi_before
##   6   61  bmi  hb_a1c_diag_numeric  time_between_bmi_before
##   6   62  bmi  hb_a1c_diag_numeric  time_between_bmi_before
##   6   63  bmi  hb_a1c_diag_numeric  time_between_bmi_before
##   6   64  bmi  hb_a1c_diag_numeric  time_between_bmi_before
##   6   65  bmi  hb_a1c_diag_numeric  time_between_bmi_before
##   7   1  bmi  hb_a1c_diag_numeric  time_between_bmi_before
##   7   2  bmi  hb_a1c_diag_numeric  time_between_bmi_before
##   7   3  bmi  hb_a1c_diag_numeric  time_between_bmi_before
##   7   4  bmi  hb_a1c_diag_numeric  time_between_bmi_before
##   7   5  bmi  hb_a1c_diag_numeric  time_between_bmi_before
##   7   6  bmi  hb_a1c_diag_numeric  time_between_bmi_before
##   7   7  bmi  hb_a1c_diag_numeric  time_between_bmi_before
##   7   8  bmi  hb_a1c_diag_numeric  time_between_bmi_before
##   7   9  bmi  hb_a1c_diag_numeric  time_between_bmi_before
##   7   10  bmi  hb_a1c_diag_numeric  time_between_bmi_before
##   7   11  bmi  hb_a1c_diag_numeric  time_between_bmi_before
##   7   12  bmi  hb_a1c_diag_numeric  time_between_bmi_before
##   7   13  bmi  hb_a1c_diag_numeric  time_between_bmi_before
##   7   14  bmi  hb_a1c_diag_numeric  time_between_bmi_before
##   7   15  bmi  hb_a1c_diag_numeric  time_between_bmi_before
##   7   16  bmi  hb_a1c_diag_numeric  time_between_bmi_before
##   7   17  bmi  hb_a1c_diag_numeric  time_between_bmi_before
##   7   18  bmi  hb_a1c_diag_numeric  time_between_bmi_before
##   7   19  bmi  hb_a1c_diag_numeric  time_between_bmi_before
##   7   20  bmi  hb_a1c_diag_numeric  time_between_bmi_before
##   7   21  bmi  hb_a1c_diag_numeric  time_between_bmi_before
##   7   22  bmi  hb_a1c_diag_numeric  time_between_bmi_before
##   7   23  bmi  hb_a1c_diag_numeric  time_between_bmi_before
##   7   24  bmi  hb_a1c_diag_numeric  time_between_bmi_before
##   7   25  bmi  hb_a1c_diag_numeric  time_between_bmi_before
##   7   26  bmi  hb_a1c_diag_numeric  time_between_bmi_before
##   7   27  bmi  hb_a1c_diag_numeric  time_between_bmi_before
##   7   28  bmi  hb_a1c_diag_numeric  time_between_bmi_before
##   7   29  bmi  hb_a1c_diag_numeric  time_between_bmi_before
##   7   30  bmi  hb_a1c_diag_numeric  time_between_bmi_before
##   7   31  bmi  hb_a1c_diag_numeric  time_between_bmi_before
##   7   32  bmi  hb_a1c_diag_numeric  time_between_bmi_before
##   7   33  bmi  hb_a1c_diag_numeric  time_between_bmi_before
##   7   34  bmi  hb_a1c_diag_numeric  time_between_bmi_before
##   7   35  bmi  hb_a1c_diag_numeric  time_between_bmi_before
##   7   36  bmi  hb_a1c_diag_numeric  time_between_bmi_before
##   7   37  bmi  hb_a1c_diag_numeric  time_between_bmi_before
##   7   38  bmi  hb_a1c_diag_numeric  time_between_bmi_before
##   7   39  bmi  hb_a1c_diag_numeric  time_between_bmi_before
##   7   40  bmi  hb_a1c_diag_numeric  time_between_bmi_before
##   7   41  bmi  hb_a1c_diag_numeric  time_between_bmi_before
##   7   42  bmi  hb_a1c_diag_numeric  time_between_bmi_before
##   7   43  bmi  hb_a1c_diag_numeric  time_between_bmi_before
##   7   44  bmi  hb_a1c_diag_numeric  time_between_bmi_before
##   7   45  bmi  hb_a1c_diag_numeric  time_between_bmi_before
##   7   46  bmi  hb_a1c_diag_numeric  time_between_bmi_before
##   7   47  bmi  hb_a1c_diag_numeric  time_between_bmi_before
##   7   48  bmi  hb_a1c_diag_numeric  time_between_bmi_before
##   7   49  bmi  hb_a1c_diag_numeric  time_between_bmi_before
##   7   50  bmi  hb_a1c_diag_numeric  time_between_bmi_before
##   7   51  bmi  hb_a1c_diag_numeric  time_between_bmi_before
##   7   52  bmi  hb_a1c_diag_numeric  time_between_bmi_before
##   7   53  bmi  hb_a1c_diag_numeric  time_between_bmi_before
##   7   54  bmi  hb_a1c_diag_numeric  time_between_bmi_before
##   7   55  bmi  hb_a1c_diag_numeric  time_between_bmi_before
##   7   56  bmi  hb_a1c_diag_numeric  time_between_bmi_before
##   7   57  bmi  hb_a1c_diag_numeric  time_between_bmi_before
##   7   58  bmi  hb_a1c_diag_numeric  time_between_bmi_before
##   7   59  bmi  hb_a1c_diag_numeric  time_between_bmi_before
##   7   60  bmi  hb_a1c_diag_numeric  time_between_bmi_before
##   7   61  bmi  hb_a1c_diag_numeric  time_between_bmi_before
##   7   62  bmi  hb_a1c_diag_numeric  time_between_bmi_before
##   7   63  bmi  hb_a1c_diag_numeric  time_between_bmi_before
##   7   64  bmi  hb_a1c_diag_numeric  time_between_bmi_before
##   7   65  bmi  hb_a1c_diag_numeric  time_between_bmi_before
##   8   1  bmi  hb_a1c_diag_numeric  time_between_bmi_before
##   8   2  bmi  hb_a1c_diag_numeric  time_between_bmi_before
##   8   3  bmi  hb_a1c_diag_numeric  time_between_bmi_before
##   8   4  bmi  hb_a1c_diag_numeric  time_between_bmi_before
##   8   5  bmi  hb_a1c_diag_numeric  time_between_bmi_before
##   8   6  bmi  hb_a1c_diag_numeric  time_between_bmi_before
##   8   7  bmi  hb_a1c_diag_numeric  time_between_bmi_before
##   8   8  bmi  hb_a1c_diag_numeric  time_between_bmi_before
##   8   9  bmi  hb_a1c_diag_numeric  time_between_bmi_before
##   8   10  bmi  hb_a1c_diag_numeric  time_between_bmi_before
##   8   11  bmi  hb_a1c_diag_numeric  time_between_bmi_before
##   8   12  bmi  hb_a1c_diag_numeric  time_between_bmi_before
##   8   13  bmi  hb_a1c_diag_numeric  time_between_bmi_before
##   8   14  bmi  hb_a1c_diag_numeric  time_between_bmi_before
##   8   15  bmi  hb_a1c_diag_numeric  time_between_bmi_before
##   8   16  bmi  hb_a1c_diag_numeric  time_between_bmi_before
##   8   17  bmi  hb_a1c_diag_numeric  time_between_bmi_before
##   8   18  bmi  hb_a1c_diag_numeric  time_between_bmi_before
##   8   19  bmi  hb_a1c_diag_numeric  time_between_bmi_before
##   8   20  bmi  hb_a1c_diag_numeric  time_between_bmi_before
##   8   21  bmi  hb_a1c_diag_numeric  time_between_bmi_before
##   8   22  bmi  hb_a1c_diag_numeric  time_between_bmi_before
##   8   23  bmi  hb_a1c_diag_numeric  time_between_bmi_before
##   8   24  bmi  hb_a1c_diag_numeric  time_between_bmi_before
##   8   25  bmi  hb_a1c_diag_numeric  time_between_bmi_before
##   8   26  bmi  hb_a1c_diag_numeric  time_between_bmi_before
##   8   27  bmi  hb_a1c_diag_numeric  time_between_bmi_before
##   8   28  bmi  hb_a1c_diag_numeric  time_between_bmi_before
##   8   29  bmi  hb_a1c_diag_numeric  time_between_bmi_before
##   8   30  bmi  hb_a1c_diag_numeric  time_between_bmi_before
##   8   31  bmi  hb_a1c_diag_numeric  time_between_bmi_before
##   8   32  bmi  hb_a1c_diag_numeric  time_between_bmi_before
##   8   33  bmi  hb_a1c_diag_numeric  time_between_bmi_before
##   8   34  bmi  hb_a1c_diag_numeric  time_between_bmi_before
##   8   35  bmi  hb_a1c_diag_numeric  time_between_bmi_before
##   8   36  bmi  hb_a1c_diag_numeric  time_between_bmi_before
##   8   37  bmi  hb_a1c_diag_numeric  time_between_bmi_before
##   8   38  bmi  hb_a1c_diag_numeric  time_between_bmi_before
##   8   39  bmi  hb_a1c_diag_numeric  time_between_bmi_before
##   8   40  bmi  hb_a1c_diag_numeric  time_between_bmi_before
##   8   41  bmi  hb_a1c_diag_numeric  time_between_bmi_before
##   8   42  bmi  hb_a1c_diag_numeric  time_between_bmi_before
##   8   43  bmi  hb_a1c_diag_numeric  time_between_bmi_before
##   8   44  bmi  hb_a1c_diag_numeric  time_between_bmi_before
##   8   45  bmi  hb_a1c_diag_numeric  time_between_bmi_before
##   8   46  bmi  hb_a1c_diag_numeric  time_between_bmi_before
##   8   47  bmi  hb_a1c_diag_numeric  time_between_bmi_before
##   8   48  bmi  hb_a1c_diag_numeric  time_between_bmi_before
##   8   49  bmi  hb_a1c_diag_numeric  time_between_bmi_before
##   8   50  bmi  hb_a1c_diag_numeric  time_between_bmi_before
##   8   51  bmi  hb_a1c_diag_numeric  time_between_bmi_before
##   8   52  bmi  hb_a1c_diag_numeric  time_between_bmi_before
##   8   53  bmi  hb_a1c_diag_numeric  time_between_bmi_before
##   8   54  bmi  hb_a1c_diag_numeric  time_between_bmi_before
##   8   55  bmi  hb_a1c_diag_numeric  time_between_bmi_before
##   8   56  bmi  hb_a1c_diag_numeric  time_between_bmi_before
##   8   57  bmi  hb_a1c_diag_numeric  time_between_bmi_before
##   8   58  bmi  hb_a1c_diag_numeric  time_between_bmi_before
##   8   59  bmi  hb_a1c_diag_numeric  time_between_bmi_before
##   8   60  bmi  hb_a1c_diag_numeric  time_between_bmi_before
##   8   61  bmi  hb_a1c_diag_numeric  time_between_bmi_before
##   8   62  bmi  hb_a1c_diag_numeric  time_between_bmi_before
##   8   63  bmi  hb_a1c_diag_numeric  time_between_bmi_before
##   8   64  bmi  hb_a1c_diag_numeric  time_between_bmi_before
##   8   65  bmi  hb_a1c_diag_numeric  time_between_bmi_before
##   9   1  bmi  hb_a1c_diag_numeric  time_between_bmi_before
##   9   2  bmi  hb_a1c_diag_numeric  time_between_bmi_before
##   9   3  bmi  hb_a1c_diag_numeric  time_between_bmi_before
##   9   4  bmi  hb_a1c_diag_numeric  time_between_bmi_before
##   9   5  bmi  hb_a1c_diag_numeric  time_between_bmi_before
##   9   6  bmi  hb_a1c_diag_numeric  time_between_bmi_before
##   9   7  bmi  hb_a1c_diag_numeric  time_between_bmi_before
##   9   8  bmi  hb_a1c_diag_numeric  time_between_bmi_before
##   9   9  bmi  hb_a1c_diag_numeric  time_between_bmi_before
##   9   10  bmi  hb_a1c_diag_numeric  time_between_bmi_before
##   9   11  bmi  hb_a1c_diag_numeric  time_between_bmi_before
##   9   12  bmi  hb_a1c_diag_numeric  time_between_bmi_before
##   9   13  bmi  hb_a1c_diag_numeric  time_between_bmi_before
##   9   14  bmi  hb_a1c_diag_numeric  time_between_bmi_before
##   9   15  bmi  hb_a1c_diag_numeric  time_between_bmi_before
##   9   16  bmi  hb_a1c_diag_numeric  time_between_bmi_before
##   9   17  bmi  hb_a1c_diag_numeric  time_between_bmi_before
##   9   18  bmi  hb_a1c_diag_numeric  time_between_bmi_before
##   9   19  bmi  hb_a1c_diag_numeric  time_between_bmi_before
##   9   20  bmi  hb_a1c_diag_numeric  time_between_bmi_before
##   9   21  bmi  hb_a1c_diag_numeric  time_between_bmi_before
##   9   22  bmi  hb_a1c_diag_numeric  time_between_bmi_before
##   9   23  bmi  hb_a1c_diag_numeric  time_between_bmi_before
##   9   24  bmi  hb_a1c_diag_numeric  time_between_bmi_before
##   9   25  bmi  hb_a1c_diag_numeric  time_between_bmi_before
##   9   26  bmi  hb_a1c_diag_numeric  time_between_bmi_before
##   9   27  bmi  hb_a1c_diag_numeric  time_between_bmi_before
##   9   28  bmi  hb_a1c_diag_numeric  time_between_bmi_before
##   9   29  bmi  hb_a1c_diag_numeric  time_between_bmi_before
##   9   30  bmi  hb_a1c_diag_numeric  time_between_bmi_before
##   9   31  bmi  hb_a1c_diag_numeric  time_between_bmi_before
##   9   32  bmi  hb_a1c_diag_numeric  time_between_bmi_before
##   9   33  bmi  hb_a1c_diag_numeric  time_between_bmi_before
##   9   34  bmi  hb_a1c_diag_numeric  time_between_bmi_before
##   9   35  bmi  hb_a1c_diag_numeric  time_between_bmi_before
##   9   36  bmi  hb_a1c_diag_numeric  time_between_bmi_before
##   9   37  bmi  hb_a1c_diag_numeric  time_between_bmi_before
##   9   38  bmi  hb_a1c_diag_numeric  time_between_bmi_before
##   9   39  bmi  hb_a1c_diag_numeric  time_between_bmi_before
##   9   40  bmi  hb_a1c_diag_numeric  time_between_bmi_before
##   9   41  bmi  hb_a1c_diag_numeric  time_between_bmi_before
##   9   42  bmi  hb_a1c_diag_numeric  time_between_bmi_before
##   9   43  bmi  hb_a1c_diag_numeric  time_between_bmi_before
##   9   44  bmi  hb_a1c_diag_numeric  time_between_bmi_before
##   9   45  bmi  hb_a1c_diag_numeric  time_between_bmi_before
##   9   46  bmi  hb_a1c_diag_numeric  time_between_bmi_before
##   9   47  bmi  hb_a1c_diag_numeric  time_between_bmi_before
##   9   48  bmi  hb_a1c_diag_numeric  time_between_bmi_before
##   9   49  bmi  hb_a1c_diag_numeric  time_between_bmi_before
##   9   50  bmi  hb_a1c_diag_numeric  time_between_bmi_before
##   9   51  bmi  hb_a1c_diag_numeric  time_between_bmi_before
##   9   52  bmi  hb_a1c_diag_numeric  time_between_bmi_before
##   9   53  bmi  hb_a1c_diag_numeric  time_between_bmi_before
##   9   54  bmi  hb_a1c_diag_numeric  time_between_bmi_before
##   9   55  bmi  hb_a1c_diag_numeric  time_between_bmi_before
##   9   56  bmi  hb_a1c_diag_numeric  time_between_bmi_before
##   9   57  bmi  hb_a1c_diag_numeric  time_between_bmi_before
##   9   58  bmi  hb_a1c_diag_numeric  time_between_bmi_before
##   9   59  bmi  hb_a1c_diag_numeric  time_between_bmi_before
##   9   60  bmi  hb_a1c_diag_numeric  time_between_bmi_before
##   9   61  bmi  hb_a1c_diag_numeric  time_between_bmi_before
##   9   62  bmi  hb_a1c_diag_numeric  time_between_bmi_before
##   9   63  bmi  hb_a1c_diag_numeric  time_between_bmi_before
##   9   64  bmi  hb_a1c_diag_numeric  time_between_bmi_before
##   9   65  bmi  hb_a1c_diag_numeric  time_between_bmi_before
##   10   1  bmi  hb_a1c_diag_numeric  time_between_bmi_before
##   10   2  bmi  hb_a1c_diag_numeric  time_between_bmi_before
##   10   3  bmi  hb_a1c_diag_numeric  time_between_bmi_before
##   10   4  bmi  hb_a1c_diag_numeric  time_between_bmi_before
##   10   5  bmi  hb_a1c_diag_numeric  time_between_bmi_before
##   10   6  bmi  hb_a1c_diag_numeric  time_between_bmi_before
##   10   7  bmi  hb_a1c_diag_numeric  time_between_bmi_before
##   10   8  bmi  hb_a1c_diag_numeric  time_between_bmi_before
##   10   9  bmi  hb_a1c_diag_numeric  time_between_bmi_before
##   10   10  bmi  hb_a1c_diag_numeric  time_between_bmi_before
##   10   11  bmi  hb_a1c_diag_numeric  time_between_bmi_before
##   10   12  bmi  hb_a1c_diag_numeric  time_between_bmi_before
##   10   13  bmi  hb_a1c_diag_numeric  time_between_bmi_before
##   10   14  bmi  hb_a1c_diag_numeric  time_between_bmi_before
##   10   15  bmi  hb_a1c_diag_numeric  time_between_bmi_before
##   10   16  bmi  hb_a1c_diag_numeric  time_between_bmi_before
##   10   17  bmi  hb_a1c_diag_numeric  time_between_bmi_before
##   10   18  bmi  hb_a1c_diag_numeric  time_between_bmi_before
##   10   19  bmi  hb_a1c_diag_numeric  time_between_bmi_before
##   10   20  bmi  hb_a1c_diag_numeric  time_between_bmi_before
##   10   21  bmi  hb_a1c_diag_numeric  time_between_bmi_before
##   10   22  bmi  hb_a1c_diag_numeric  time_between_bmi_before
##   10   23  bmi  hb_a1c_diag_numeric  time_between_bmi_before
##   10   24  bmi  hb_a1c_diag_numeric  time_between_bmi_before
##   10   25  bmi  hb_a1c_diag_numeric  time_between_bmi_before
##   10   26  bmi  hb_a1c_diag_numeric  time_between_bmi_before
##   10   27  bmi  hb_a1c_diag_numeric  time_between_bmi_before
##   10   28  bmi  hb_a1c_diag_numeric  time_between_bmi_before
##   10   29  bmi  hb_a1c_diag_numeric  time_between_bmi_before
##   10   30  bmi  hb_a1c_diag_numeric  time_between_bmi_before
##   10   31  bmi  hb_a1c_diag_numeric  time_between_bmi_before
##   10   32  bmi  hb_a1c_diag_numeric  time_between_bmi_before
##   10   33  bmi  hb_a1c_diag_numeric  time_between_bmi_before
##   10   34  bmi  hb_a1c_diag_numeric  time_between_bmi_before
##   10   35  bmi  hb_a1c_diag_numeric  time_between_bmi_before
##   10   36  bmi  hb_a1c_diag_numeric  time_between_bmi_before
##   10   37  bmi  hb_a1c_diag_numeric  time_between_bmi_before
##   10   38  bmi  hb_a1c_diag_numeric  time_between_bmi_before
##   10   39  bmi  hb_a1c_diag_numeric  time_between_bmi_before
##   10   40  bmi  hb_a1c_diag_numeric  time_between_bmi_before
##   10   41  bmi  hb_a1c_diag_numeric  time_between_bmi_before
##   10   42  bmi  hb_a1c_diag_numeric  time_between_bmi_before
##   10   43  bmi  hb_a1c_diag_numeric  time_between_bmi_before
##   10   44  bmi  hb_a1c_diag_numeric  time_between_bmi_before
##   10   45  bmi  hb_a1c_diag_numeric  time_between_bmi_before
##   10   46  bmi  hb_a1c_diag_numeric  time_between_bmi_before
##   10   47  bmi  hb_a1c_diag_numeric  time_between_bmi_before
##   10   48  bmi  hb_a1c_diag_numeric  time_between_bmi_before
##   10   49  bmi  hb_a1c_diag_numeric  time_between_bmi_before
##   10   50  bmi  hb_a1c_diag_numeric  time_between_bmi_before
##   10   51  bmi  hb_a1c_diag_numeric  time_between_bmi_before
##   10   52  bmi  hb_a1c_diag_numeric  time_between_bmi_before
##   10   53  bmi  hb_a1c_diag_numeric  time_between_bmi_before
##   10   54  bmi  hb_a1c_diag_numeric  time_between_bmi_before
##   10   55  bmi  hb_a1c_diag_numeric  time_between_bmi_before
##   10   56  bmi  hb_a1c_diag_numeric  time_between_bmi_before
##   10   57  bmi  hb_a1c_diag_numeric  time_between_bmi_before
##   10   58  bmi  hb_a1c_diag_numeric  time_between_bmi_before
##   10   59  bmi  hb_a1c_diag_numeric  time_between_bmi_before
##   10   60  bmi  hb_a1c_diag_numeric  time_between_bmi_before
##   10   61  bmi  hb_a1c_diag_numeric  time_between_bmi_before
##   10   62  bmi  hb_a1c_diag_numeric  time_between_bmi_before
##   10   63  bmi  hb_a1c_diag_numeric  time_between_bmi_before
##   10   64  bmi  hb_a1c_diag_numeric  time_between_bmi_before
##   10   65  bmi  hb_a1c_diag_numeric  time_between_bmi_before
```

### Diagnostic

```
# 1. Inspect methods
nlp_imputed$method
```

```
##              rct_status                     sex                     age                race_eth                     bmi                     t1d                     t2d                  dm_any     hb_a1c_diag_numeric    hpt_at_1st_diag_code 
##                      ""                      ""                      ""                      ""                   "pmm"                      ""                      ""                      ""                   "pmm"                      "" 
##    hpt_at_1st_diag_meds     ligamentous_disease            prior_injury               arthritis time_between_bmi_before 
##                      ""                      ""                      ""                      ""                   "pmm"
```

```
# 2. Trace plots (Convergence)
# title: MICE Algorithm Convergence Trace Plots for BMI and Clinical Covariates
plot(nlp_imputed)
```

```
# 3. Density plots (Observed vs Imputed)
# title: Comparison of Observed vs. Imputed BMI Distributions (Density Plots)
densityplot(nlp_imputed, ~bmi)
```

```
# 4. Extract FMI and Lambda from a fully adjusted model
# We use Model 5 as the representative diagnostic model
pooled_diag <- nlp_imputed %>%
  with(glm(rct_status ~ bmi + age + sex + race_eth, family = binomial)) %>%
  pool()

# Create a clean table for the supplement
mi_diagnostics <- pooled_diag$pooled %>%
  dplyr::select(term, riv, lambda, fmi) %>%
  mutate(across(where(is.numeric), ~round(., 4)))

# title: Multiple Imputation Diagnostic Statistics (FMI and Lambda) for the Fully Adjusted NLP Model
print(mi_diagnostics)
```

```
##                         term    riv lambda    fmi
## 1                (Intercept) 0.0605 0.0570 0.0571
## 2                        bmi 0.7553 0.4303 0.4336
## 3                        age 0.0299 0.0291 0.0291
## 4                    sexMale 0.0070 0.0069 0.0069
## 5                       sexU 0.0026 0.0025 0.0026
## 6 race_ethBlack Non-Hispanic 0.0055 0.0055 0.0055
## 7           race_ethHispanic 0.0029 0.0029 0.0029
## 8              race_ethOther 0.0034 0.0034 0.0034
## 9 race_ethWhite Non-Hispanic 0.0032 0.0032 0.0032
```

interpretation after imputation: - ppm: predictive mean matching, for
continuous variables. - An empty string �� means that the variable was
not imputed. Because: 1) no missing data; 2) skipped by algorithm, often
due to perfect collinearity.

Trace Plots:

- No Obvious Trends: For all three imputed variables (bmi,
  hb\_a1c\_diag\_numeric, and time\_between\_bmi\_before), the lines move
  horizontally across the plot without any clear upward or downward
  pattern.
- Good Mixing: The lines are tangled together in what is often called
  a �fuzzy caterpillar,� which shows that the imputation process is stable
  and the different imputed datasets are similar to each other.

Density Plot (Observed vs.�Imputed) - Blue Line: observed BMI data. -
Red Lines: distributions of 65 imputed datasets. - The imputed values
(red) follow the shape of the observed data (blue) very closely, but
they are slightly more �peaked� and shifted left. This indicates that
the MICE algorithm correctly identified that the �missing� population
tended to have slightly lower BMIs based on their other characteristics
(eg, younger age or control status), which supports the Missing at
Random (MAR) assumption.

Fraction of Missing Information (FMI) - For BMI, the FMI is 0.4336. -
This is lower than the raw percentage of missing data (61.2%). This
proves that by including the outcome (rct\_status) and covariates like
age, sex, race in the imputation model, we effectively recovered roughly
18% of the information that would have been lost in a simple
complete-case analysis.

## create bmi\_cat

```
# Create `bmi_cat` within Imputed Datasets

# Convert the imputed object to a long-format data frame
long_df <- mice::complete(nlp_imputed, action = "long", include = TRUE)

# Add the original `rct_status` back
long_df$rct_status <- rep(nlp_df$rct_status, nlp_imputed$m + 1)

# Create `bmi_cat` and set the reference level
long_df_wrangled <- long_df %>%
  mutate(
    bmi_cat = case_when(
      bmi < 18.5          ~ "Underweight (<18.5)",
      bmi >= 18.5 & bmi < 25 ~ "Normal (18.5-24.9)",
      bmi >= 25   & bmi < 30 ~ "Overweight (25-29.9)",
      bmi >= 30           ~ "Obese (>=30)"
    ),
    # Convert to factor and set the reference group
    bmi_cat = fct_relevel(bmi_cat, "Normal (18.5-24.9)")
  )

# Convert the modified long data frame back into a `Multiply imputed data set (mids)` object for analysis
nlp_final_mids <- as.mids(long_df_wrangled)
```

## pooled analysis

ref: Control (NLP)

```
# run each model on the imputed data and pool the results.
# `tbl_regression` automatically understands pooled results and formats them.
# The `variable` argument is used to select the predictors we are interested in.
library(gtsummary)

run_and_format <- function(mids_obj, formula, include_vars) {
  with(mids_obj, glm(as.formula(formula), family = binomial)) %>%
    tbl_regression(exponentiate = TRUE) %>%
    modify_table_body(~.x %>% filter(variable %in% include_vars)) %>%
    add_n(location = "level")
}

cat("### Model 1: RCT Status ~ Any Diabetes (Adjusted)")
```

```
## ### Model 1: RCT Status ~ Any Diabetes (Adjusted)
```

```
run_and_format(nlp_final_mids, "rct_status ~ dm_any + age + sex + race_eth + bmi", c("dm_any", "bmi"))
```

| **Characteristic** | **N** | **OR** | **95% CI** | **p-value** |
| --- | --- | --- | --- | --- |
| dm\_any |  |  |  |  |
| ����None | 250,725 | � | � |  |
| ����Any | 23,505 | 1.62 | 1.57, 1.67 | <0.001 |
| bmi | 274,230 | 1.02 | 1.02, 1.02 | <0.001 |
|  |  |  |  |  |
| --- | --- | --- | --- | --- |
| Abbreviations: CI = Confidence Interval, OR = Odds Ratio | | | | |

```
cat("\n### Model 2: RCT Status ~ T1 Diabetes (Adjusted)")
```

```
## 
## ### Model 2: RCT Status ~ T1 Diabetes (Adjusted)
```

```
run_and_format(nlp_final_mids, "rct_status ~ t1d + age + sex + race_eth + bmi", c("t1d", "bmi"))
```

| **Characteristic** | **N** | **OR** | **95% CI** | **p-value** |
| --- | --- | --- | --- | --- |
| t1d |  |  |  |  |
| ����No | 250,725 | � | � |  |
| ����Yes | 3,852 | 1.95 | 1.82, 2.09 | <0.001 |
| bmi | 254,577 | 1.02 | 1.02, 1.02 | <0.001 |
|  |  |  |  |  |
| --- | --- | --- | --- | --- |
| Abbreviations: CI = Confidence Interval, OR = Odds Ratio | | | | |

```
cat("\n### Model 3: RCT Status ~ T2 Diabetes (Adjusted)")
```

```
## 
## ### Model 3: RCT Status ~ T2 Diabetes (Adjusted)
```

```
run_and_format(nlp_final_mids, "rct_status ~ t2d + age + sex + race_eth + bmi", c("t2d", "bmi"))
```

| **Characteristic** | **N** | **OR** | **95% CI** | **p-value** |
| --- | --- | --- | --- | --- |
| t2d |  |  |  |  |
| ����No | 250,725 | � | � |  |
| ����Yes | 10,381 | 1.63 | 1.56, 1.70 | <0.001 |
| bmi | 261,106 | 1.02 | 1.02, 1.02 | <0.001 |
|  |  |  |  |  |
| --- | --- | --- | --- | --- |
| Abbreviations: CI = Confidence Interval, OR = Odds Ratio | | | | |

```
cat("\n### Model 4: RCT Status ~ BMI (Unadjusted)")
```

```
## 
## ### Model 4: RCT Status ~ BMI (Unadjusted)
```

```
run_and_format(nlp_final_mids, "rct_status ~ bmi", "bmi")
```

| **Characteristic** | **N** | **OR** | **95% CI** | **p-value** |
| --- | --- | --- | --- | --- |
| bmi | 274,230 | 1.02 | 1.01, 1.02 | <0.001 |
|  |  |  |  |  |
| --- | --- | --- | --- | --- |
| Abbreviations: CI = Confidence Interval, OR = Odds Ratio | | | | |

```
cat("\n### Model 5: RCT Status ~ BMI (Adjusted)")
```

```
## 
## ### Model 5: RCT Status ~ BMI (Adjusted)
```

```
run_and_format(nlp_final_mids, "rct_status ~ bmi + age + sex + race_eth", "bmi")
```

| **Characteristic** | **N** | **OR** | **95% CI** | **p-value** |
| --- | --- | --- | --- | --- |
| bmi | 274,230 | 1.02 | 1.02, 1.03 | <0.001 |
|  |  |  |  |  |
| --- | --- | --- | --- | --- |
| Abbreviations: CI = Confidence Interval, OR = Odds Ratio | | | | |

```
cat("\n### Model 6: RCT Status ~ BMI Category (Unadjusted)")
```

```
## 
## ### Model 6: RCT Status ~ BMI Category (Unadjusted)
```

```
run_and_format(nlp_final_mids, "rct_status ~ bmi_cat", "bmi_cat")
```

| **Characteristic** | **N** | **OR** | **95% CI** | **p-value** |
| --- | --- | --- | --- | --- |
| bmi\_cat |  |  |  |  |
| ����Normal (18.5-24.9) | 72,980 | � | � |  |
| ����Obese (>=30) | 102,431 | 1.33 | 1.28, 1.37 | <0.001 |
| ����Overweight (25-29.9) | 94,008 | 1.17 | 1.13, 1.21 | <0.001 |
| ����Underweight (<18.5) | 4,811 | 0.85 | 0.77, 0.95 | 0.005 |
|  |  |  |  |  |
| --- | --- | --- | --- | --- |
| Abbreviations: CI = Confidence Interval, OR = Odds Ratio | | | | |

```
cat("\n### Model 7: RCT Status ~ BMI Category (Adjusted)")
```

```
## 
## ### Model 7: RCT Status ~ BMI Category (Adjusted)
```

```
run_and_format(nlp_final_mids, "rct_status ~ bmi_cat + age + sex + race_eth", "bmi_cat")
```

| **Characteristic** | **N** | **OR** | **95% CI** | **p-value** |
| --- | --- | --- | --- | --- |
| bmi\_cat |  |  |  |  |
| ����Normal (18.5-24.9) | 72,980 | � | � |  |
| ����Obese (>=30) | 102,431 | 1.47 | 1.42, 1.52 | <0.001 |
| ����Overweight (25-29.9) | 94,008 | 1.20 | 1.16, 1.24 | <0.001 |
| ����Underweight (<18.5) | 4,811 | 0.82 | 0.73, 0.91 | <0.001 |
|  |  |  |  |  |
| --- | --- | --- | --- | --- |
| Abbreviations: CI = Confidence Interval, OR = Odds Ratio | | | | |

```
cat("\n### Model 8: RCT Status ~ BMI Category (Adjusted + T1D)")
```

```
## 
## ### Model 8: RCT Status ~ BMI Category (Adjusted + T1D)
```

```
run_and_format(nlp_final_mids, "rct_status ~ bmi_cat + age + sex + race_eth + t1d", c("t1d", "bmi_cat"))
```

| **Characteristic** | **N** | **OR** | **95% CI** | **p-value** |
| --- | --- | --- | --- | --- |
| bmi\_cat |  |  |  |  |
| ����Normal (18.5-24.9) | 70,199 | � | � |  |
| ����Obese (>=30) | 91,197 | 1.39 | 1.34, 1.44 | <0.001 |
| ����Overweight (25-29.9) | 88,516 | 1.17 | 1.13, 1.21 | <0.001 |
| ����Underweight (<18.5) | 4,665 | 0.82 | 0.73, 0.92 | <0.001 |
| t1d |  |  |  |  |
| ����No | 250,725 | � | � |  |
| ����Yes | 3,852 | 1.98 | 1.85, 2.12 | <0.001 |
|  |  |  |  |  |
| --- | --- | --- | --- | --- |
| Abbreviations: CI = Confidence Interval, OR = Odds Ratio | | | | |

# code\_image\_df: Multiple Imputation for bmi

- outcome: rct\_status
- do not include: distinct\_visit\_cnt, bmi\_cat (derivative of bmi)
- set imputed dataset m to be at least the percentage of missing
  cases.

## abnormal bmi and bmi\_cat

```
library(tidyverse)

mismatched_bmi <- code_image_df %>%
  # create a new column with the CORRECT category based on the bmi value
  mutate(
    correct_bmi_cat = case_when(
      bmi < 18.5          ~ "Underweight (<18.5)",
      bmi >= 18.5 & bmi < 25 ~ "Normal (18.5-24.9)",
      bmi >= 25   & bmi < 30 ~ "Overweight (25-29.9)",
      bmi >= 30           ~ "Obese (>30)",
      TRUE                ~ NA_character_
    )
  ) %>%
  # filter for rows that meet any of error conditions
  filter(
    # Condition 1: The original category doesn't match the correct one
    (bmi_cat != correct_bmi_cat & !is.na(bmi)) |
    # Condition 2: The BMI value is biologically implausible
    (bmi > 150 | bmi < 10)
  ) %>%
  # Select relevant columns
  dplyr::select(rct_status, age, sex, bmi, bmi_cat, correct_bmi_cat)


print(mismatched_bmi)
```

```
## # A tibble: 12 � 6
##    rct_status             age sex      bmi bmi_cat              correct_bmi_cat     
##    <fct>                <dbl> <fct>  <dbl> <fct>                <chr>               
##  1 Control (Code Image)  48.1 Male      30 Overweight (25-29.9) Obese (>30)         
##  2 Control (Code Image)  44.6 Male      30 Overweight (25-29.9) Obese (>30)         
##  3 Control (Code Image)  40.5 Male      30 Overweight (25-29.9) Obese (>30)         
##  4 Control (Code Image)  83.7 Female    30 Overweight (25-29.9) Obese (>30)         
##  5 Control (Code Image)  48.2 Male      25 Normal (18.5-24.9)   Overweight (25-29.9)
##  6 Control (Code Image)  49.3 Male      30 Overweight (25-29.9) Obese (>30)         
##  7 Control (Code Image)  42.1 Female    25 Normal (18.5-24.9)   Overweight (25-29.9)
##  8 Control (Code Image)  57.9 Male      30 Overweight (25-29.9) Obese (>30)         
##  9 Control (Code Image)  42.5 Male      25 Normal (18.5-24.9)   Overweight (25-29.9)
## 10 Control (Code Image)  51.9 Male      30 Overweight (25-29.9) Obese (>30)         
## 11 Case (Code Image)     42.8 Male      25 Normal (18.5-24.9)   Overweight (25-29.9)
## 12 Control (Code Image)  56.8 Male      30 Overweight (25-29.9) Obese (>30)
```

```
# Get a count of how many mismatches were found
cat(glue::glue("\nFound {nrow(mismatched_bmi)} rows with BMI mismatches or implausible values."))
```

```
## Found 12 rows with BMI mismatches or implausible values.
```

```
# Set implausible BMI values to NA
code_image_df <- code_image_df %>%
  mutate(
    bmi = if_else(bmi > 150 | bmi < 10, NA_real_, bmi)
  )
```

- Biologically implausible BMI values (bmi >150 or <10) were
  recoded as missing (NA) to be properly handled during the multiple
  imputation process.
- note: The BMI categories may need redefine to align with CDC/WHO
  guidelines. Specifically, boundary values are now included in the higher
  category; for example, a BMI of 30.0 is classified as �Obese (?30)�
  rather than �Overweight�; a BMI of 25.0 is classified as �Overweight
  (25-29.9)� rather than �Normal (18.5-24.9)�.

## check missingness in bmi

```
library(tidyverse)
library(naniar)

miss_var_summary(code_image_df)
```

```
## # A tibble: 17 � 3
##    variable                n_miss pct_miss
##    <chr>                    <int>    <num>
##  1 hb_a1c_diag_numeric      10228  77.7   
##  2 bmi                       4475  34.0   
##  3 bmi_cat                   4475  34.0   
##  4 time_between_bmi_before   3882  29.5   
##  5 t1d                       1600  12.2   
##  6 t2d                       1168   8.88  
##  7 distinct_visit_cnt           2   0.0152
##  8 rct_status                   0   0     
##  9 sex                          0   0     
## 10 age                          0   0     
## 11 race_eth                     0   0     
## 12 dm_any                       0   0     
## 13 hpt_at_1st_diag_code         0   0     
## 14 hpt_at_1st_diag_meds         0   0     
## 15 ligamentous_disease          0   0     
## 16 prior_injury                 0   0     
## 17 arthritis                    0   0
```

```
# Set "Control (Code Image)" as the reference level for the outcome variable
code_image_df <- code_image_df %>%
  mutate(rct_status = fct_relevel(rct_status, "Control (Code Image)"))
```

## imputation

```
# Prepare Imputation Data (Outcome rct_status is INCLUDED)
vars_for_imputation <- setdiff(names(code_image_df), c("distinct_visit_cnt", "bmi_cat"))

code_image_impute_data <- code_image_df %>%
  dplyr::select(all_of(vars_for_imputation))

# Configure MICE (Outcome used as predictor but not imputed)
meth <- make.method(code_image_impute_data)
meth["rct_status"] <- "" 
pred <- make.predictorMatrix(code_image_impute_data)

# Run MICE algorithm (Using m=40 and increased iterations)
set.seed(123)
code_image_imputed <- mice(code_image_impute_data, 
                           method = meth, 
                           predictorMatrix = pred, 
                           m = 65, 
                           maxit = 10, 
                           printFlag = TRUE)
```

```
## 
##  iter imp variable
##   1   1  bmi  hb_a1c_diag_numeric  time_between_bmi_before
##   1   2  bmi  hb_a1c_diag_numeric  time_between_bmi_before
##   1   3  bmi  hb_a1c_diag_numeric  time_between_bmi_before
##   1   4  bmi  hb_a1c_diag_numeric  time_between_bmi_before
##   1   5  bmi  hb_a1c_diag_numeric  time_between_bmi_before
##   1   6  bmi  hb_a1c_diag_numeric  time_between_bmi_before
##   1   7  bmi  hb_a1c_diag_numeric  time_between_bmi_before
##   1   8  bmi  hb_a1c_diag_numeric  time_between_bmi_before
##   1   9  bmi  hb_a1c_diag_numeric  time_between_bmi_before
##   1   10  bmi  hb_a1c_diag_numeric  time_between_bmi_before
##   1   11  bmi  hb_a1c_diag_numeric  time_between_bmi_before
##   1   12  bmi  hb_a1c_diag_numeric  time_between_bmi_before
##   1   13  bmi  hb_a1c_diag_numeric  time_between_bmi_before
##   1   14  bmi  hb_a1c_diag_numeric  time_between_bmi_before
##   1   15  bmi  hb_a1c_diag_numeric  time_between_bmi_before
##   1   16  bmi  hb_a1c_diag_numeric  time_between_bmi_before
##   1   17  bmi  hb_a1c_diag_numeric  time_between_bmi_before
##   1   18  bmi  hb_a1c_diag_numeric  time_between_bmi_before
##   1   19  bmi  hb_a1c_diag_numeric  time_between_bmi_before
##   1   20  bmi  hb_a1c_diag_numeric  time_between_bmi_before
##   1   21  bmi  hb_a1c_diag_numeric  time_between_bmi_before
##   1   22  bmi  hb_a1c_diag_numeric  time_between_bmi_before
##   1   23  bmi  hb_a1c_diag_numeric  time_between_bmi_before
##   1   24  bmi  hb_a1c_diag_numeric  time_between_bmi_before
##   1   25  bmi  hb_a1c_diag_numeric  time_between_bmi_before
##   1   26  bmi  hb_a1c_diag_numeric  time_between_bmi_before
##   1   27  bmi  hb_a1c_diag_numeric  time_between_bmi_before
##   1   28  bmi  hb_a1c_diag_numeric  time_between_bmi_before
##   1   29  bmi  hb_a1c_diag_numeric  time_between_bmi_before
##   1   30  bmi  hb_a1c_diag_numeric  time_between_bmi_before
##   1   31  bmi  hb_a1c_diag_numeric  time_between_bmi_before
##   1   32  bmi  hb_a1c_diag_numeric  time_between_bmi_before
##   1   33  bmi  hb_a1c_diag_numeric  time_between_bmi_before
##   1   34  bmi  hb_a1c_diag_numeric  time_between_bmi_before
##   1   35  bmi  hb_a1c_diag_numeric  time_between_bmi_before
##   1   36  bmi  hb_a1c_diag_numeric  time_between_bmi_before
##   1   37  bmi  hb_a1c_diag_numeric  time_between_bmi_before
##   1   38  bmi  hb_a1c_diag_numeric  time_between_bmi_before
##   1   39  bmi  hb_a1c_diag_numeric  time_between_bmi_before
##   1   40  bmi  hb_a1c_diag_numeric  time_between_bmi_before
##   1   41  bmi  hb_a1c_diag_numeric  time_between_bmi_before
##   1   42  bmi  hb_a1c_diag_numeric  time_between_bmi_before
##   1   43  bmi  hb_a1c_diag_numeric  time_between_bmi_before
##   1   44  bmi  hb_a1c_diag_numeric  time_between_bmi_before
##   1   45  bmi  hb_a1c_diag_numeric  time_between_bmi_before
##   1   46  bmi  hb_a1c_diag_numeric  time_between_bmi_before
##   1   47  bmi  hb_a1c_diag_numeric  time_between_bmi_before
##   1   48  bmi  hb_a1c_diag_numeric  time_between_bmi_before
##   1   49  bmi  hb_a1c_diag_numeric  time_between_bmi_before
##   1   50  bmi  hb_a1c_diag_numeric  time_between_bmi_before
##   1   51  bmi  hb_a1c_diag_numeric  time_between_bmi_before
##   1   52  bmi  hb_a1c_diag_numeric  time_between_bmi_before
##   1   53  bmi  hb_a1c_diag_numeric  time_between_bmi_before
##   1   54  bmi  hb_a1c_diag_numeric  time_between_bmi_before
##   1   55  bmi  hb_a1c_diag_numeric  time_between_bmi_before
##   1   56  bmi  hb_a1c_diag_numeric  time_between_bmi_before
##   1   57  bmi  hb_a1c_diag_numeric  time_between_bmi_before
##   1   58  bmi  hb_a1c_diag_numeric  time_between_bmi_before
##   1   59  bmi  hb_a1c_diag_numeric  time_between_bmi_before
##   1   60  bmi  hb_a1c_diag_numeric  time_between_bmi_before
##   1   61  bmi  hb_a1c_diag_numeric  time_between_bmi_before
##   1   62  bmi  hb_a1c_diag_numeric  time_between_bmi_before
##   1   63  bmi  hb_a1c_diag_numeric  time_between_bmi_before
##   1   64  bmi  hb_a1c_diag_numeric  time_between_bmi_before
##   1   65  bmi  hb_a1c_diag_numeric  time_between_bmi_before
##   2   1  bmi  hb_a1c_diag_numeric  time_between_bmi_before
##   2   2  bmi  hb_a1c_diag_numeric  time_between_bmi_before
##   2   3  bmi  hb_a1c_diag_numeric  time_between_bmi_before
##   2   4  bmi  hb_a1c_diag_numeric  time_between_bmi_before
##   2   5  bmi  hb_a1c_diag_numeric  time_between_bmi_before
##   2   6  bmi  hb_a1c_diag_numeric  time_between_bmi_before
##   2   7  bmi  hb_a1c_diag_numeric  time_between_bmi_before
##   2   8  bmi  hb_a1c_diag_numeric  time_between_bmi_before
##   2   9  bmi  hb_a1c_diag_numeric  time_between_bmi_before
##   2   10  bmi  hb_a1c_diag_numeric  time_between_bmi_before
##   2   11  bmi  hb_a1c_diag_numeric  time_between_bmi_before
##   2   12  bmi  hb_a1c_diag_numeric  time_between_bmi_before
##   2   13  bmi  hb_a1c_diag_numeric  time_between_bmi_before
##   2   14  bmi  hb_a1c_diag_numeric  time_between_bmi_before
##   2   15  bmi  hb_a1c_diag_numeric  time_between_bmi_before
##   2   16  bmi  hb_a1c_diag_numeric  time_between_bmi_before
##   2   17  bmi  hb_a1c_diag_numeric  time_between_bmi_before
##   2   18  bmi  hb_a1c_diag_numeric  time_between_bmi_before
##   2   19  bmi  hb_a1c_diag_numeric  time_between_bmi_before
##   2   20  bmi  hb_a1c_diag_numeric  time_between_bmi_before
##   2   21  bmi  hb_a1c_diag_numeric  time_between_bmi_before
##   2   22  bmi  hb_a1c_diag_numeric  time_between_bmi_before
##   2   23  bmi  hb_a1c_diag_numeric  time_between_bmi_before
##   2   24  bmi  hb_a1c_diag_numeric  time_between_bmi_before
##   2   25  bmi  hb_a1c_diag_numeric  time_between_bmi_before
##   2   26  bmi  hb_a1c_diag_numeric  time_between_bmi_before
##   2   27  bmi  hb_a1c_diag_numeric  time_between_bmi_before
##   2   28  bmi  hb_a1c_diag_numeric  time_between_bmi_before
##   2   29  bmi  hb_a1c_diag_numeric  time_between_bmi_before
##   2   30  bmi  hb_a1c_diag_numeric  time_between_bmi_before
##   2   31  bmi  hb_a1c_diag_numeric  time_between_bmi_before
##   2   32  bmi  hb_a1c_diag_numeric  time_between_bmi_before
##   2   33  bmi  hb_a1c_diag_numeric  time_between_bmi_before
##   2   34  bmi  hb_a1c_diag_numeric  time_between_bmi_before
##   2   35  bmi  hb_a1c_diag_numeric  time_between_bmi_before
##   2   36  bmi  hb_a1c_diag_numeric  time_between_bmi_before
##   2   37  bmi  hb_a1c_diag_numeric  time_between_bmi_before
##   2   38  bmi  hb_a1c_diag_numeric  time_between_bmi_before
##   2   39  bmi  hb_a1c_diag_numeric  time_between_bmi_before
##   2   40  bmi  hb_a1c_diag_numeric  time_between_bmi_before
##   2   41  bmi  hb_a1c_diag_numeric  time_between_bmi_before
##   2   42  bmi  hb_a1c_diag_numeric  time_between_bmi_before
##   2   43  bmi  hb_a1c_diag_numeric  time_between_bmi_before
##   2   44  bmi  hb_a1c_diag_numeric  time_between_bmi_before
##   2   45  bmi  hb_a1c_diag_numeric  time_between_bmi_before
##   2   46  bmi  hb_a1c_diag_numeric  time_between_bmi_before
##   2   47  bmi  hb_a1c_diag_numeric  time_between_bmi_before
##   2   48  bmi  hb_a1c_diag_numeric  time_between_bmi_before
##   2   49  bmi  hb_a1c_diag_numeric  time_between_bmi_before
##   2   50  bmi  hb_a1c_diag_numeric  time_between_bmi_before
##   2   51  bmi  hb_a1c_diag_numeric  time_between_bmi_before
##   2   52  bmi  hb_a1c_diag_numeric  time_between_bmi_before
##   2   53  bmi  hb_a1c_diag_numeric  time_between_bmi_before
##   2   54  bmi  hb_a1c_diag_numeric  time_between_bmi_before
##   2   55  bmi  hb_a1c_diag_numeric  time_between_bmi_before
##   2   56  bmi  hb_a1c_diag_numeric  time_between_bmi_before
##   2   57  bmi  hb_a1c_diag_numeric  time_between_bmi_before
##   2   58  bmi  hb_a1c_diag_numeric  time_between_bmi_before
##   2   59  bmi  hb_a1c_diag_numeric  time_between_bmi_before
##   2   60  bmi  hb_a1c_diag_numeric  time_between_bmi_before
##   2   61  bmi  hb_a1c_diag_numeric  time_between_bmi_before
##   2   62  bmi  hb_a1c_diag_numeric  time_between_bmi_before
##   2   63  bmi  hb_a1c_diag_numeric  time_between_bmi_before
##   2   64  bmi  hb_a1c_diag_numeric  time_between_bmi_before
##   2   65  bmi  hb_a1c_diag_numeric  time_between_bmi_before
##   3   1  bmi  hb_a1c_diag_numeric  time_between_bmi_before
##   3   2  bmi  hb_a1c_diag_numeric  time_between_bmi_before
##   3   3  bmi  hb_a1c_diag_numeric  time_between_bmi_before
##   3   4  bmi  hb_a1c_diag_numeric  time_between_bmi_before
##   3   5  bmi  hb_a1c_diag_numeric  time_between_bmi_before
##   3   6  bmi  hb_a1c_diag_numeric  time_between_bmi_before
##   3   7  bmi  hb_a1c_diag_numeric  time_between_bmi_before
##   3   8  bmi  hb_a1c_diag_numeric  time_between_bmi_before
##   3   9  bmi  hb_a1c_diag_numeric  time_between_bmi_before
##   3   10  bmi  hb_a1c_diag_numeric  time_between_bmi_before
##   3   11  bmi  hb_a1c_diag_numeric  time_between_bmi_before
##   3   12  bmi  hb_a1c_diag_numeric  time_between_bmi_before
##   3   13  bmi  hb_a1c_diag_numeric  time_between_bmi_before
##   3   14  bmi  hb_a1c_diag_numeric  time_between_bmi_before
##   3   15  bmi  hb_a1c_diag_numeric  time_between_bmi_before
##   3   16  bmi  hb_a1c_diag_numeric  time_between_bmi_before
##   3   17  bmi  hb_a1c_diag_numeric  time_between_bmi_before
##   3   18  bmi  hb_a1c_diag_numeric  time_between_bmi_before
##   3   19  bmi  hb_a1c_diag_numeric  time_between_bmi_before
##   3   20  bmi  hb_a1c_diag_numeric  time_between_bmi_before
##   3   21  bmi  hb_a1c_diag_numeric  time_between_bmi_before
##   3   22  bmi  hb_a1c_diag_numeric  time_between_bmi_before
##   3   23  bmi  hb_a1c_diag_numeric  time_between_bmi_before
##   3   24  bmi  hb_a1c_diag_numeric  time_between_bmi_before
##   3   25  bmi  hb_a1c_diag_numeric  time_between_bmi_before
##   3   26  bmi  hb_a1c_diag_numeric  time_between_bmi_before
##   3   27  bmi  hb_a1c_diag_numeric  time_between_bmi_before
##   3   28  bmi  hb_a1c_diag_numeric  time_between_bmi_before
##   3   29  bmi  hb_a1c_diag_numeric  time_between_bmi_before
##   3   30  bmi  hb_a1c_diag_numeric  time_between_bmi_before
##   3   31  bmi  hb_a1c_diag_numeric  time_between_bmi_before
##   3   32  bmi  hb_a1c_diag_numeric  time_between_bmi_before
##   3   33  bmi  hb_a1c_diag_numeric  time_between_bmi_before
##   3   34  bmi  hb_a1c_diag_numeric  time_between_bmi_before
##   3   35  bmi  hb_a1c_diag_numeric  time_between_bmi_before
##   3   36  bmi  hb_a1c_diag_numeric  time_between_bmi_before
##   3   37  bmi  hb_a1c_diag_numeric  time_between_bmi_before
##   3   38  bmi  hb_a1c_diag_numeric  time_between_bmi_before
##   3   39  bmi  hb_a1c_diag_numeric  time_between_bmi_before
##   3   40  bmi  hb_a1c_diag_numeric  time_between_bmi_before
##   3   41  bmi  hb_a1c_diag_numeric  time_between_bmi_before
##   3   42  bmi  hb_a1c_diag_numeric  time_between_bmi_before
##   3   43  bmi  hb_a1c_diag_numeric  time_between_bmi_before
##   3   44  bmi  hb_a1c_diag_numeric  time_between_bmi_before
##   3   45  bmi  hb_a1c_diag_numeric  time_between_bmi_before
##   3   46  bmi  hb_a1c_diag_numeric  time_between_bmi_before
##   3   47  bmi  hb_a1c_diag_numeric  time_between_bmi_before
##   3   48  bmi  hb_a1c_diag_numeric  time_between_bmi_before
##   3   49  bmi  hb_a1c_diag_numeric  time_between_bmi_before
##   3   50  bmi  hb_a1c_diag_numeric  time_between_bmi_before
##   3   51  bmi  hb_a1c_diag_numeric  time_between_bmi_before
##   3   52  bmi  hb_a1c_diag_numeric  time_between_bmi_before
##   3   53  bmi  hb_a1c_diag_numeric  time_between_bmi_before
##   3   54  bmi  hb_a1c_diag_numeric  time_between_bmi_before
##   3   55  bmi  hb_a1c_diag_numeric  time_between_bmi_before
##   3   56  bmi  hb_a1c_diag_numeric  time_between_bmi_before
##   3   57  bmi  hb_a1c_diag_numeric  time_between_bmi_before
##   3   58  bmi  hb_a1c_diag_numeric  time_between_bmi_before
##   3   59  bmi  hb_a1c_diag_numeric  time_between_bmi_before
##   3   60  bmi  hb_a1c_diag_numeric  time_between_bmi_before
##   3   61  bmi  hb_a1c_diag_numeric  time_between_bmi_before
##   3   62  bmi  hb_a1c_diag_numeric  time_between_bmi_before
##   3   63  bmi  hb_a1c_diag_numeric  time_between_bmi_before
##   3   64  bmi  hb_a1c_diag_numeric  time_between_bmi_before
##   3   65  bmi  hb_a1c_diag_numeric  time_between_bmi_before
##   4   1  bmi  hb_a1c_diag_numeric  time_between_bmi_before
##   4   2  bmi  hb_a1c_diag_numeric  time_between_bmi_before
##   4   3  bmi  hb_a1c_diag_numeric  time_between_bmi_before
##   4   4  bmi  hb_a1c_diag_numeric  time_between_bmi_before
##   4   5  bmi  hb_a1c_diag_numeric  time_between_bmi_before
##   4   6  bmi  hb_a1c_diag_numeric  time_between_bmi_before
##   4   7  bmi  hb_a1c_diag_numeric  time_between_bmi_before
##   4   8  bmi  hb_a1c_diag_numeric  time_between_bmi_before
##   4   9  bmi  hb_a1c_diag_numeric  time_between_bmi_before
##   4   10  bmi  hb_a1c_diag_numeric  time_between_bmi_before
##   4   11  bmi  hb_a1c_diag_numeric  time_between_bmi_before
##   4   12  bmi  hb_a1c_diag_numeric  time_between_bmi_before
##   4   13  bmi  hb_a1c_diag_numeric  time_between_bmi_before
##   4   14  bmi  hb_a1c_diag_numeric  time_between_bmi_before
##   4   15  bmi  hb_a1c_diag_numeric  time_between_bmi_before
##   4   16  bmi  hb_a1c_diag_numeric  time_between_bmi_before
##   4   17  bmi  hb_a1c_diag_numeric  time_between_bmi_before
##   4   18  bmi  hb_a1c_diag_numeric  time_between_bmi_before
##   4   19  bmi  hb_a1c_diag_numeric  time_between_bmi_before
##   4   20  bmi  hb_a1c_diag_numeric  time_between_bmi_before
##   4   21  bmi  hb_a1c_diag_numeric  time_between_bmi_before
##   4   22  bmi  hb_a1c_diag_numeric  time_between_bmi_before
##   4   23  bmi  hb_a1c_diag_numeric  time_between_bmi_before
##   4   24  bmi  hb_a1c_diag_numeric  time_between_bmi_before
##   4   25  bmi  hb_a1c_diag_numeric  time_between_bmi_before
##   4   26  bmi  hb_a1c_diag_numeric  time_between_bmi_before
##   4   27  bmi  hb_a1c_diag_numeric  time_between_bmi_before
##   4   28  bmi  hb_a1c_diag_numeric  time_between_bmi_before
##   4   29  bmi  hb_a1c_diag_numeric  time_between_bmi_before
##   4   30  bmi  hb_a1c_diag_numeric  time_between_bmi_before
##   4   31  bmi  hb_a1c_diag_numeric  time_between_bmi_before
##   4   32  bmi  hb_a1c_diag_numeric  time_between_bmi_before
##   4   33  bmi  hb_a1c_diag_numeric  time_between_bmi_before
##   4   34  bmi  hb_a1c_diag_numeric  time_between_bmi_before
##   4   35  bmi  hb_a1c_diag_numeric  time_between_bmi_before
##   4   36  bmi  hb_a1c_diag_numeric  time_between_bmi_before
##   4   37  bmi  hb_a1c_diag_numeric  time_between_bmi_before
##   4   38  bmi  hb_a1c_diag_numeric  time_between_bmi_before
##   4   39  bmi  hb_a1c_diag_numeric  time_between_bmi_before
##   4   40  bmi  hb_a1c_diag_numeric  time_between_bmi_before
##   4   41  bmi  hb_a1c_diag_numeric  time_between_bmi_before
##   4   42  bmi  hb_a1c_diag_numeric  time_between_bmi_before
##   4   43  bmi  hb_a1c_diag_numeric  time_between_bmi_before
##   4   44  bmi  hb_a1c_diag_numeric  time_between_bmi_before
##   4   45  bmi  hb_a1c_diag_numeric  time_between_bmi_before
##   4   46  bmi  hb_a1c_diag_numeric  time_between_bmi_before
##   4   47  bmi  hb_a1c_diag_numeric  time_between_bmi_before
##   4   48  bmi  hb_a1c_diag_numeric  time_between_bmi_before
##   4   49  bmi  hb_a1c_diag_numeric  time_between_bmi_before
##   4   50  bmi  hb_a1c_diag_numeric  time_between_bmi_before
##   4   51  bmi  hb_a1c_diag_numeric  time_between_bmi_before
##   4   52  bmi  hb_a1c_diag_numeric  time_between_bmi_before
##   4   53  bmi  hb_a1c_diag_numeric  time_between_bmi_before
##   4   54  bmi  hb_a1c_diag_numeric  time_between_bmi_before
##   4   55  bmi  hb_a1c_diag_numeric  time_between_bmi_before
##   4   56  bmi  hb_a1c_diag_numeric  time_between_bmi_before
##   4   57  bmi  hb_a1c_diag_numeric  time_between_bmi_before
##   4   58  bmi  hb_a1c_diag_numeric  time_between_bmi_before
##   4   59  bmi  hb_a1c_diag_numeric  time_between_bmi_before
##   4   60  bmi  hb_a1c_diag_numeric  time_between_bmi_before
##   4   61  bmi  hb_a1c_diag_numeric  time_between_bmi_before
##   4   62  bmi  hb_a1c_diag_numeric  time_between_bmi_before
##   4   63  bmi  hb_a1c_diag_numeric  time_between_bmi_before
##   4   64  bmi  hb_a1c_diag_numeric  time_between_bmi_before
##   4   65  bmi  hb_a1c_diag_numeric  time_between_bmi_before
##   5   1  bmi  hb_a1c_diag_numeric  time_between_bmi_before
##   5   2  bmi  hb_a1c_diag_numeric  time_between_bmi_before
##   5   3  bmi  hb_a1c_diag_numeric  time_between_bmi_before
##   5   4  bmi  hb_a1c_diag_numeric  time_between_bmi_before
##   5   5  bmi  hb_a1c_diag_numeric  time_between_bmi_before
##   5   6  bmi  hb_a1c_diag_numeric  time_between_bmi_before
##   5   7  bmi  hb_a1c_diag_numeric  time_between_bmi_before
##   5   8  bmi  hb_a1c_diag_numeric  time_between_bmi_before
##   5   9  bmi  hb_a1c_diag_numeric  time_between_bmi_before
##   5   10  bmi  hb_a1c_diag_numeric  time_between_bmi_before
##   5   11  bmi  hb_a1c_diag_numeric  time_between_bmi_before
##   5   12  bmi  hb_a1c_diag_numeric  time_between_bmi_before
##   5   13  bmi  hb_a1c_diag_numeric  time_between_bmi_before
##   5   14  bmi  hb_a1c_diag_numeric  time_between_bmi_before
##   5   15  bmi  hb_a1c_diag_numeric  time_between_bmi_before
##   5   16  bmi  hb_a1c_diag_numeric  time_between_bmi_before
##   5   17  bmi  hb_a1c_diag_numeric  time_between_bmi_before
##   5   18  bmi  hb_a1c_diag_numeric  time_between_bmi_before
##   5   19  bmi  hb_a1c_diag_numeric  time_between_bmi_before
##   5   20  bmi  hb_a1c_diag_numeric  time_between_bmi_before
##   5   21  bmi  hb_a1c_diag_numeric  time_between_bmi_before
##   5   22  bmi  hb_a1c_diag_numeric  time_between_bmi_before
##   5   23  bmi  hb_a1c_diag_numeric  time_between_bmi_before
##   5   24  bmi  hb_a1c_diag_numeric  time_between_bmi_before
##   5   25  bmi  hb_a1c_diag_numeric  time_between_bmi_before
##   5   26  bmi  hb_a1c_diag_numeric  time_between_bmi_before
##   5   27  bmi  hb_a1c_diag_numeric  time_between_bmi_before
##   5   28  bmi  hb_a1c_diag_numeric  time_between_bmi_before
##   5   29  bmi  hb_a1c_diag_numeric  time_between_bmi_before
##   5   30  bmi  hb_a1c_diag_numeric  time_between_bmi_before
##   5   31  bmi  hb_a1c_diag_numeric  time_between_bmi_before
##   5   32  bmi  hb_a1c_diag_numeric  time_between_bmi_before
##   5   33  bmi  hb_a1c_diag_numeric  time_between_bmi_before
##   5   34  bmi  hb_a1c_diag_numeric  time_between_bmi_before
##   5   35  bmi  hb_a1c_diag_numeric  time_between_bmi_before
##   5   36  bmi  hb_a1c_diag_numeric  time_between_bmi_before
##   5   37  bmi  hb_a1c_diag_numeric  time_between_bmi_before
##   5   38  bmi  hb_a1c_diag_numeric  time_between_bmi_before
##   5   39  bmi  hb_a1c_diag_numeric  time_between_bmi_before
##   5   40  bmi  hb_a1c_diag_numeric  time_between_bmi_before
##   5   41  bmi  hb_a1c_diag_numeric  time_between_bmi_before
##   5   42  bmi  hb_a1c_diag_numeric  time_between_bmi_before
##   5   43  bmi  hb_a1c_diag_numeric  time_between_bmi_before
##   5   44  bmi  hb_a1c_diag_numeric  time_between_bmi_before
##   5   45  bmi  hb_a1c_diag_numeric  time_between_bmi_before
##   5   46  bmi  hb_a1c_diag_numeric  time_between_bmi_before
##   5   47  bmi  hb_a1c_diag_numeric  time_between_bmi_before
##   5   48  bmi  hb_a1c_diag_numeric  time_between_bmi_before
##   5   49  bmi  hb_a1c_diag_numeric  time_between_bmi_before
##   5   50  bmi  hb_a1c_diag_numeric  time_between_bmi_before
##   5   51  bmi  hb_a1c_diag_numeric  time_between_bmi_before
##   5   52  bmi  hb_a1c_diag_numeric  time_between_bmi_before
##   5   53  bmi  hb_a1c_diag_numeric  time_between_bmi_before
##   5   54  bmi  hb_a1c_diag_numeric  time_between_bmi_before
##   5   55  bmi  hb_a1c_diag_numeric  time_between_bmi_before
##   5   56  bmi  hb_a1c_diag_numeric  time_between_bmi_before
##   5   57  bmi  hb_a1c_diag_numeric  time_between_bmi_before
##   5   58  bmi  hb_a1c_diag_numeric  time_between_bmi_before
##   5   59  bmi  hb_a1c_diag_numeric  time_between_bmi_before
##   5   60  bmi  hb_a1c_diag_numeric  time_between_bmi_before
##   5   61  bmi  hb_a1c_diag_numeric  time_between_bmi_before
##   5   62  bmi  hb_a1c_diag_numeric  time_between_bmi_before
##   5   63  bmi  hb_a1c_diag_numeric  time_between_bmi_before
##   5   64  bmi  hb_a1c_diag_numeric  time_between_bmi_before
##   5   65  bmi  hb_a1c_diag_numeric  time_between_bmi_before
##   6   1  bmi  hb_a1c_diag_numeric  time_between_bmi_before
##   6   2  bmi  hb_a1c_diag_numeric  time_between_bmi_before
##   6   3  bmi  hb_a1c_diag_numeric  time_between_bmi_before
##   6   4  bmi  hb_a1c_diag_numeric  time_between_bmi_before
##   6   5  bmi  hb_a1c_diag_numeric  time_between_bmi_before
##   6   6  bmi  hb_a1c_diag_numeric  time_between_bmi_before
##   6   7  bmi  hb_a1c_diag_numeric  time_between_bmi_before
##   6   8  bmi  hb_a1c_diag_numeric  time_between_bmi_before
##   6   9  bmi  hb_a1c_diag_numeric  time_between_bmi_before
##   6   10  bmi  hb_a1c_diag_numeric  time_between_bmi_before
##   6   11  bmi  hb_a1c_diag_numeric  time_between_bmi_before
##   6   12  bmi  hb_a1c_diag_numeric  time_between_bmi_before
##   6   13  bmi  hb_a1c_diag_numeric  time_between_bmi_before
##   6   14  bmi  hb_a1c_diag_numeric  time_between_bmi_before
##   6   15  bmi  hb_a1c_diag_numeric  time_between_bmi_before
##   6   16  bmi  hb_a1c_diag_numeric  time_between_bmi_before
##   6   17  bmi  hb_a1c_diag_numeric  time_between_bmi_before
##   6   18  bmi  hb_a1c_diag_numeric  time_between_bmi_before
##   6   19  bmi  hb_a1c_diag_numeric  time_between_bmi_before
##   6   20  bmi  hb_a1c_diag_numeric  time_between_bmi_before
##   6   21  bmi  hb_a1c_diag_numeric  time_between_bmi_before
##   6   22  bmi  hb_a1c_diag_numeric  time_between_bmi_before
##   6   23  bmi  hb_a1c_diag_numeric  time_between_bmi_before
##   6   24  bmi  hb_a1c_diag_numeric  time_between_bmi_before
##   6   25  bmi  hb_a1c_diag_numeric  time_between_bmi_before
##   6   26  bmi  hb_a1c_diag_numeric  time_between_bmi_before
##   6   27  bmi  hb_a1c_diag_numeric  time_between_bmi_before
##   6   28  bmi  hb_a1c_diag_numeric  time_between_bmi_before
##   6   29  bmi  hb_a1c_diag_numeric  time_between_bmi_before
##   6   30  bmi  hb_a1c_diag_numeric  time_between_bmi_before
##   6   31  bmi  hb_a1c_diag_numeric  time_between_bmi_before
##   6   32  bmi  hb_a1c_diag_numeric  time_between_bmi_before
##   6   33  bmi  hb_a1c_diag_numeric  time_between_bmi_before
##   6   34  bmi  hb_a1c_diag_numeric  time_between_bmi_before
##   6   35  bmi  hb_a1c_diag_numeric  time_between_bmi_before
##   6   36  bmi  hb_a1c_diag_numeric  time_between_bmi_before
##   6   37  bmi  hb_a1c_diag_numeric  time_between_bmi_before
##   6   38  bmi  hb_a1c_diag_numeric  time_between_bmi_before
##   6   39  bmi  hb_a1c_diag_numeric  time_between_bmi_before
##   6   40  bmi  hb_a1c_diag_numeric  time_between_bmi_before
##   6   41  bmi  hb_a1c_diag_numeric  time_between_bmi_before
##   6   42  bmi  hb_a1c_diag_numeric  time_between_bmi_before
##   6   43  bmi  hb_a1c_diag_numeric  time_between_bmi_before
##   6   44  bmi  hb_a1c_diag_numeric  time_between_bmi_before
##   6   45  bmi  hb_a1c_diag_numeric  time_between_bmi_before
##   6   46  bmi  hb_a1c_diag_numeric  time_between_bmi_before
##   6   47  bmi  hb_a1c_diag_numeric  time_between_bmi_before
##   6   48  bmi  hb_a1c_diag_numeric  time_between_bmi_before
##   6   49  bmi  hb_a1c_diag_numeric  time_between_bmi_before
##   6   50  bmi  hb_a1c_diag_numeric  time_between_bmi_before
##   6   51  bmi  hb_a1c_diag_numeric  time_between_bmi_before
##   6   52  bmi  hb_a1c_diag_numeric  time_between_bmi_before
##   6   53  bmi  hb_a1c_diag_numeric  time_between_bmi_before
##   6   54  bmi  hb_a1c_diag_numeric  time_between_bmi_before
##   6   55  bmi  hb_a1c_diag_numeric  time_between_bmi_before
##   6   56  bmi  hb_a1c_diag_numeric  time_between_bmi_before
##   6   57  bmi  hb_a1c_diag_numeric  time_between_bmi_before
##   6   58  bmi  hb_a1c_diag_numeric  time_between_bmi_before
##   6   59  bmi  hb_a1c_diag_numeric  time_between_bmi_before
##   6   60  bmi  hb_a1c_diag_numeric  time_between_bmi_before
##   6   61  bmi  hb_a1c_diag_numeric  time_between_bmi_before
##   6   62  bmi  hb_a1c_diag_numeric  time_between_bmi_before
##   6   63  bmi  hb_a1c_diag_numeric  time_between_bmi_before
##   6   64  bmi  hb_a1c_diag_numeric  time_between_bmi_before
##   6   65  bmi  hb_a1c_diag_numeric  time_between_bmi_before
##   7   1  bmi  hb_a1c_diag_numeric  time_between_bmi_before
##   7   2  bmi  hb_a1c_diag_numeric  time_between_bmi_before
##   7   3  bmi  hb_a1c_diag_numeric  time_between_bmi_before
##   7   4  bmi  hb_a1c_diag_numeric  time_between_bmi_before
##   7   5  bmi  hb_a1c_diag_numeric  time_between_bmi_before
##   7   6  bmi  hb_a1c_diag_numeric  time_between_bmi_before
##   7   7  bmi  hb_a1c_diag_numeric  time_between_bmi_before
##   7   8  bmi  hb_a1c_diag_numeric  time_between_bmi_before
##   7   9  bmi  hb_a1c_diag_numeric  time_between_bmi_before
##   7   10  bmi  hb_a1c_diag_numeric  time_between_bmi_before
##   7   11  bmi  hb_a1c_diag_numeric  time_between_bmi_before
##   7   12  bmi  hb_a1c_diag_numeric  time_between_bmi_before
##   7   13  bmi  hb_a1c_diag_numeric  time_between_bmi_before
##   7   14  bmi  hb_a1c_diag_numeric  time_between_bmi_before
##   7   15  bmi  hb_a1c_diag_numeric  time_between_bmi_before
##   7   16  bmi  hb_a1c_diag_numeric  time_between_bmi_before
##   7   17  bmi  hb_a1c_diag_numeric  time_between_bmi_before
##   7   18  bmi  hb_a1c_diag_numeric  time_between_bmi_before
##   7   19  bmi  hb_a1c_diag_numeric  time_between_bmi_before
##   7   20  bmi  hb_a1c_diag_numeric  time_between_bmi_before
##   7   21  bmi  hb_a1c_diag_numeric  time_between_bmi_before
##   7   22  bmi  hb_a1c_diag_numeric  time_between_bmi_before
##   7   23  bmi  hb_a1c_diag_numeric  time_between_bmi_before
##   7   24  bmi  hb_a1c_diag_numeric  time_between_bmi_before
##   7   25  bmi  hb_a1c_diag_numeric  time_between_bmi_before
##   7   26  bmi  hb_a1c_diag_numeric  time_between_bmi_before
##   7   27  bmi  hb_a1c_diag_numeric  time_between_bmi_before
##   7   28  bmi  hb_a1c_diag_numeric  time_between_bmi_before
##   7   29  bmi  hb_a1c_diag_numeric  time_between_bmi_before
##   7   30  bmi  hb_a1c_diag_numeric  time_between_bmi_before
##   7   31  bmi  hb_a1c_diag_numeric  time_between_bmi_before
##   7   32  bmi  hb_a1c_diag_numeric  time_between_bmi_before
##   7   33  bmi  hb_a1c_diag_numeric  time_between_bmi_before
##   7   34  bmi  hb_a1c_diag_numeric  time_between_bmi_before
##   7   35  bmi  hb_a1c_diag_numeric  time_between_bmi_before
##   7   36  bmi  hb_a1c_diag_numeric  time_between_bmi_before
##   7   37  bmi  hb_a1c_diag_numeric  time_between_bmi_before
##   7   38  bmi  hb_a1c_diag_numeric  time_between_bmi_before
##   7   39  bmi  hb_a1c_diag_numeric  time_between_bmi_before
##   7   40  bmi  hb_a1c_diag_numeric  time_between_bmi_before
##   7   41  bmi  hb_a1c_diag_numeric  time_between_bmi_before
##   7   42  bmi  hb_a1c_diag_numeric  time_between_bmi_before
##   7   43  bmi  hb_a1c_diag_numeric  time_between_bmi_before
##   7   44  bmi  hb_a1c_diag_numeric  time_between_bmi_before
##   7   45  bmi  hb_a1c_diag_numeric  time_between_bmi_before
##   7   46  bmi  hb_a1c_diag_numeric  time_between_bmi_before
##   7   47  bmi  hb_a1c_diag_numeric  time_between_bmi_before
##   7   48  bmi  hb_a1c_diag_numeric  time_between_bmi_before
##   7   49  bmi  hb_a1c_diag_numeric  time_between_bmi_before
##   7   50  bmi  hb_a1c_diag_numeric  time_between_bmi_before
##   7   51  bmi  hb_a1c_diag_numeric  time_between_bmi_before
##   7   52  bmi  hb_a1c_diag_numeric  time_between_bmi_before
##   7   53  bmi  hb_a1c_diag_numeric  time_between_bmi_before
##   7   54  bmi  hb_a1c_diag_numeric  time_between_bmi_before
##   7   55  bmi  hb_a1c_diag_numeric  time_between_bmi_before
##   7   56  bmi  hb_a1c_diag_numeric  time_between_bmi_before
##   7   57  bmi  hb_a1c_diag_numeric  time_between_bmi_before
##   7   58  bmi  hb_a1c_diag_numeric  time_between_bmi_before
##   7   59  bmi  hb_a1c_diag_numeric  time_between_bmi_before
##   7   60  bmi  hb_a1c_diag_numeric  time_between_bmi_before
##   7   61  bmi  hb_a1c_diag_numeric  time_between_bmi_before
##   7   62  bmi  hb_a1c_diag_numeric  time_between_bmi_before
##   7   63  bmi  hb_a1c_diag_numeric  time_between_bmi_before
##   7   64  bmi  hb_a1c_diag_numeric  time_between_bmi_before
##   7   65  bmi  hb_a1c_diag_numeric  time_between_bmi_before
##   8   1  bmi  hb_a1c_diag_numeric  time_between_bmi_before
##   8   2  bmi  hb_a1c_diag_numeric  time_between_bmi_before
##   8   3  bmi  hb_a1c_diag_numeric  time_between_bmi_before
##   8   4  bmi  hb_a1c_diag_numeric  time_between_bmi_before
##   8   5  bmi  hb_a1c_diag_numeric  time_between_bmi_before
##   8   6  bmi  hb_a1c_diag_numeric  time_between_bmi_before
##   8   7  bmi  hb_a1c_diag_numeric  time_between_bmi_before
##   8   8  bmi  hb_a1c_diag_numeric  time_between_bmi_before
##   8   9  bmi  hb_a1c_diag_numeric  time_between_bmi_before
##   8   10  bmi  hb_a1c_diag_numeric  time_between_bmi_before
##   8   11  bmi  hb_a1c_diag_numeric  time_between_bmi_before
##   8   12  bmi  hb_a1c_diag_numeric  time_between_bmi_before
##   8   13  bmi  hb_a1c_diag_numeric  time_between_bmi_before
##   8   14  bmi  hb_a1c_diag_numeric  time_between_bmi_before
##   8   15  bmi  hb_a1c_diag_numeric  time_between_bmi_before
##   8   16  bmi  hb_a1c_diag_numeric  time_between_bmi_before
##   8   17  bmi  hb_a1c_diag_numeric  time_between_bmi_before
##   8   18  bmi  hb_a1c_diag_numeric  time_between_bmi_before
##   8   19  bmi  hb_a1c_diag_numeric  time_between_bmi_before
##   8   20  bmi  hb_a1c_diag_numeric  time_between_bmi_before
##   8   21  bmi  hb_a1c_diag_numeric  time_between_bmi_before
##   8   22  bmi  hb_a1c_diag_numeric  time_between_bmi_before
##   8   23  bmi  hb_a1c_diag_numeric  time_between_bmi_before
##   8   24  bmi  hb_a1c_diag_numeric  time_between_bmi_before
##   8   25  bmi  hb_a1c_diag_numeric  time_between_bmi_before
##   8   26  bmi  hb_a1c_diag_numeric  time_between_bmi_before
##   8   27  bmi  hb_a1c_diag_numeric  time_between_bmi_before
##   8   28  bmi  hb_a1c_diag_numeric  time_between_bmi_before
##   8   29  bmi  hb_a1c_diag_numeric  time_between_bmi_before
##   8   30  bmi  hb_a1c_diag_numeric  time_between_bmi_before
##   8   31  bmi  hb_a1c_diag_numeric  time_between_bmi_before
##   8   32  bmi  hb_a1c_diag_numeric  time_between_bmi_before
##   8   33  bmi  hb_a1c_diag_numeric  time_between_bmi_before
##   8   34  bmi  hb_a1c_diag_numeric  time_between_bmi_before
##   8   35  bmi  hb_a1c_diag_numeric  time_between_bmi_before
##   8   36  bmi  hb_a1c_diag_numeric  time_between_bmi_before
##   8   37  bmi  hb_a1c_diag_numeric  time_between_bmi_before
##   8   38  bmi  hb_a1c_diag_numeric  time_between_bmi_before
##   8   39  bmi  hb_a1c_diag_numeric  time_between_bmi_before
##   8   40  bmi  hb_a1c_diag_numeric  time_between_bmi_before
##   8   41  bmi  hb_a1c_diag_numeric  time_between_bmi_before
##   8   42  bmi  hb_a1c_diag_numeric  time_between_bmi_before
##   8   43  bmi  hb_a1c_diag_numeric  time_between_bmi_before
##   8   44  bmi  hb_a1c_diag_numeric  time_between_bmi_before
##   8   45  bmi  hb_a1c_diag_numeric  time_between_bmi_before
##   8   46  bmi  hb_a1c_diag_numeric  time_between_bmi_before
##   8   47  bmi  hb_a1c_diag_numeric  time_between_bmi_before
##   8   48  bmi  hb_a1c_diag_numeric  time_between_bmi_before
##   8   49  bmi  hb_a1c_diag_numeric  time_between_bmi_before
##   8   50  bmi  hb_a1c_diag_numeric  time_between_bmi_before
##   8   51  bmi  hb_a1c_diag_numeric  time_between_bmi_before
##   8   52  bmi  hb_a1c_diag_numeric  time_between_bmi_before
##   8   53  bmi  hb_a1c_diag_numeric  time_between_bmi_before
##   8   54  bmi  hb_a1c_diag_numeric  time_between_bmi_before
##   8   55  bmi  hb_a1c_diag_numeric  time_between_bmi_before
##   8   56  bmi  hb_a1c_diag_numeric  time_between_bmi_before
##   8   57  bmi  hb_a1c_diag_numeric  time_between_bmi_before
##   8   58  bmi  hb_a1c_diag_numeric  time_between_bmi_before
##   8   59  bmi  hb_a1c_diag_numeric  time_between_bmi_before
##   8   60  bmi  hb_a1c_diag_numeric  time_between_bmi_before
##   8   61  bmi  hb_a1c_diag_numeric  time_between_bmi_before
##   8   62  bmi  hb_a1c_diag_numeric  time_between_bmi_before
##   8   63  bmi  hb_a1c_diag_numeric  time_between_bmi_before
##   8   64  bmi  hb_a1c_diag_numeric  time_between_bmi_before
##   8   65  bmi  hb_a1c_diag_numeric  time_between_bmi_before
##   9   1  bmi  hb_a1c_diag_numeric  time_between_bmi_before
##   9   2  bmi  hb_a1c_diag_numeric  time_between_bmi_before
##   9   3  bmi  hb_a1c_diag_numeric  time_between_bmi_before
##   9   4  bmi  hb_a1c_diag_numeric  time_between_bmi_before
##   9   5  bmi  hb_a1c_diag_numeric  time_between_bmi_before
##   9   6  bmi  hb_a1c_diag_numeric  time_between_bmi_before
##   9   7  bmi  hb_a1c_diag_numeric  time_between_bmi_before
##   9   8  bmi  hb_a1c_diag_numeric  time_between_bmi_before
##   9   9  bmi  hb_a1c_diag_numeric  time_between_bmi_before
##   9   10  bmi  hb_a1c_diag_numeric  time_between_bmi_before
##   9   11  bmi  hb_a1c_diag_numeric  time_between_bmi_before
##   9   12  bmi  hb_a1c_diag_numeric  time_between_bmi_before
##   9   13  bmi  hb_a1c_diag_numeric  time_between_bmi_before
##   9   14  bmi  hb_a1c_diag_numeric  time_between_bmi_before
##   9   15  bmi  hb_a1c_diag_numeric  time_between_bmi_before
##   9   16  bmi  hb_a1c_diag_numeric  time_between_bmi_before
##   9   17  bmi  hb_a1c_diag_numeric  time_between_bmi_before
##   9   18  bmi  hb_a1c_diag_numeric  time_between_bmi_before
##   9   19  bmi  hb_a1c_diag_numeric  time_between_bmi_before
##   9   20  bmi  hb_a1c_diag_numeric  time_between_bmi_before
##   9   21  bmi  hb_a1c_diag_numeric  time_between_bmi_before
##   9   22  bmi  hb_a1c_diag_numeric  time_between_bmi_before
##   9   23  bmi  hb_a1c_diag_numeric  time_between_bmi_before
##   9   24  bmi  hb_a1c_diag_numeric  time_between_bmi_before
##   9   25  bmi  hb_a1c_diag_numeric  time_between_bmi_before
##   9   26  bmi  hb_a1c_diag_numeric  time_between_bmi_before
##   9   27  bmi  hb_a1c_diag_numeric  time_between_bmi_before
##   9   28  bmi  hb_a1c_diag_numeric  time_between_bmi_before
##   9   29  bmi  hb_a1c_diag_numeric  time_between_bmi_before
##   9   30  bmi  hb_a1c_diag_numeric  time_between_bmi_before
##   9   31  bmi  hb_a1c_diag_numeric  time_between_bmi_before
##   9   32  bmi  hb_a1c_diag_numeric  time_between_bmi_before
##   9   33  bmi  hb_a1c_diag_numeric  time_between_bmi_before
##   9   34  bmi  hb_a1c_diag_numeric  time_between_bmi_before
##   9   35  bmi  hb_a1c_diag_numeric  time_between_bmi_before
##   9   36  bmi  hb_a1c_diag_numeric  time_between_bmi_before
##   9   37  bmi  hb_a1c_diag_numeric  time_between_bmi_before
##   9   38  bmi  hb_a1c_diag_numeric  time_between_bmi_before
##   9   39  bmi  hb_a1c_diag_numeric  time_between_bmi_before
##   9   40  bmi  hb_a1c_diag_numeric  time_between_bmi_before
##   9   41  bmi  hb_a1c_diag_numeric  time_between_bmi_before
##   9   42  bmi  hb_a1c_diag_numeric  time_between_bmi_before
##   9   43  bmi  hb_a1c_diag_numeric  time_between_bmi_before
##   9   44  bmi  hb_a1c_diag_numeric  time_between_bmi_before
##   9   45  bmi  hb_a1c_diag_numeric  time_between_bmi_before
##   9   46  bmi  hb_a1c_diag_numeric  time_between_bmi_before
##   9   47  bmi  hb_a1c_diag_numeric  time_between_bmi_before
##   9   48  bmi  hb_a1c_diag_numeric  time_between_bmi_before
##   9   49  bmi  hb_a1c_diag_numeric  time_between_bmi_before
##   9   50  bmi  hb_a1c_diag_numeric  time_between_bmi_before
##   9   51  bmi  hb_a1c_diag_numeric  time_between_bmi_before
##   9   52  bmi  hb_a1c_diag_numeric  time_between_bmi_before
##   9   53  bmi  hb_a1c_diag_numeric  time_between_bmi_before
##   9   54  bmi  hb_a1c_diag_numeric  time_between_bmi_before
##   9   55  bmi  hb_a1c_diag_numeric  time_between_bmi_before
##   9   56  bmi  hb_a1c_diag_numeric  time_between_bmi_before
##   9   57  bmi  hb_a1c_diag_numeric  time_between_bmi_before
##   9   58  bmi  hb_a1c_diag_numeric  time_between_bmi_before
##   9   59  bmi  hb_a1c_diag_numeric  time_between_bmi_before
##   9   60  bmi  hb_a1c_diag_numeric  time_between_bmi_before
##   9   61  bmi  hb_a1c_diag_numeric  time_between_bmi_before
##   9   62  bmi  hb_a1c_diag_numeric  time_between_bmi_before
##   9   63  bmi  hb_a1c_diag_numeric  time_between_bmi_before
##   9   64  bmi  hb_a1c_diag_numeric  time_between_bmi_before
##   9   65  bmi  hb_a1c_diag_numeric  time_between_bmi_before
##   10   1  bmi  hb_a1c_diag_numeric  time_between_bmi_before
##   10   2  bmi  hb_a1c_diag_numeric  time_between_bmi_before
##   10   3  bmi  hb_a1c_diag_numeric  time_between_bmi_before
##   10   4  bmi  hb_a1c_diag_numeric  time_between_bmi_before
##   10   5  bmi  hb_a1c_diag_numeric  time_between_bmi_before
##   10   6  bmi  hb_a1c_diag_numeric  time_between_bmi_before
##   10   7  bmi  hb_a1c_diag_numeric  time_between_bmi_before
##   10   8  bmi  hb_a1c_diag_numeric  time_between_bmi_before
##   10   9  bmi  hb_a1c_diag_numeric  time_between_bmi_before
##   10   10  bmi  hb_a1c_diag_numeric  time_between_bmi_before
##   10   11  bmi  hb_a1c_diag_numeric  time_between_bmi_before
##   10   12  bmi  hb_a1c_diag_numeric  time_between_bmi_before
##   10   13  bmi  hb_a1c_diag_numeric  time_between_bmi_before
##   10   14  bmi  hb_a1c_diag_numeric  time_between_bmi_before
##   10   15  bmi  hb_a1c_diag_numeric  time_between_bmi_before
##   10   16  bmi  hb_a1c_diag_numeric  time_between_bmi_before
##   10   17  bmi  hb_a1c_diag_numeric  time_between_bmi_before
##   10   18  bmi  hb_a1c_diag_numeric  time_between_bmi_before
##   10   19  bmi  hb_a1c_diag_numeric  time_between_bmi_before
##   10   20  bmi  hb_a1c_diag_numeric  time_between_bmi_before
##   10   21  bmi  hb_a1c_diag_numeric  time_between_bmi_before
##   10   22  bmi  hb_a1c_diag_numeric  time_between_bmi_before
##   10   23  bmi  hb_a1c_diag_numeric  time_between_bmi_before
##   10   24  bmi  hb_a1c_diag_numeric  time_between_bmi_before
##   10   25  bmi  hb_a1c_diag_numeric  time_between_bmi_before
##   10   26  bmi  hb_a1c_diag_numeric  time_between_bmi_before
##   10   27  bmi  hb_a1c_diag_numeric  time_between_bmi_before
##   10   28  bmi  hb_a1c_diag_numeric  time_between_bmi_before
##   10   29  bmi  hb_a1c_diag_numeric  time_between_bmi_before
##   10   30  bmi  hb_a1c_diag_numeric  time_between_bmi_before
##   10   31  bmi  hb_a1c_diag_numeric  time_between_bmi_before
##   10   32  bmi  hb_a1c_diag_numeric  time_between_bmi_before
##   10   33  bmi  hb_a1c_diag_numeric  time_between_bmi_before
##   10   34  bmi  hb_a1c_diag_numeric  time_between_bmi_before
##   10   35  bmi  hb_a1c_diag_numeric  time_between_bmi_before
##   10   36  bmi  hb_a1c_diag_numeric  time_between_bmi_before
##   10   37  bmi  hb_a1c_diag_numeric  time_between_bmi_before
##   10   38  bmi  hb_a1c_diag_numeric  time_between_bmi_before
##   10   39  bmi  hb_a1c_diag_numeric  time_between_bmi_before
##   10   40  bmi  hb_a1c_diag_numeric  time_between_bmi_before
##   10   41  bmi  hb_a1c_diag_numeric  time_between_bmi_before
##   10   42  bmi  hb_a1c_diag_numeric  time_between_bmi_before
##   10   43  bmi  hb_a1c_diag_numeric  time_between_bmi_before
##   10   44  bmi  hb_a1c_diag_numeric  time_between_bmi_before
##   10   45  bmi  hb_a1c_diag_numeric  time_between_bmi_before
##   10   46  bmi  hb_a1c_diag_numeric  time_between_bmi_before
##   10   47  bmi  hb_a1c_diag_numeric  time_between_bmi_before
##   10   48  bmi  hb_a1c_diag_numeric  time_between_bmi_before
##   10   49  bmi  hb_a1c_diag_numeric  time_between_bmi_before
##   10   50  bmi  hb_a1c_diag_numeric  time_between_bmi_before
##   10   51  bmi  hb_a1c_diag_numeric  time_between_bmi_before
##   10   52  bmi  hb_a1c_diag_numeric  time_between_bmi_before
##   10   53  bmi  hb_a1c_diag_numeric  time_between_bmi_before
##   10   54  bmi  hb_a1c_diag_numeric  time_between_bmi_before
##   10   55  bmi  hb_a1c_diag_numeric  time_between_bmi_before
##   10   56  bmi  hb_a1c_diag_numeric  time_between_bmi_before
##   10   57  bmi  hb_a1c_diag_numeric  time_between_bmi_before
##   10   58  bmi  hb_a1c_diag_numeric  time_between_bmi_before
##   10   59  bmi  hb_a1c_diag_numeric  time_between_bmi_before
##   10   60  bmi  hb_a1c_diag_numeric  time_between_bmi_before
##   10   61  bmi  hb_a1c_diag_numeric  time_between_bmi_before
##   10   62  bmi  hb_a1c_diag_numeric  time_between_bmi_before
##   10   63  bmi  hb_a1c_diag_numeric  time_between_bmi_before
##   10   64  bmi  hb_a1c_diag_numeric  time_between_bmi_before
##   10   65  bmi  hb_a1c_diag_numeric  time_between_bmi_before
```

### Diagnostics

```
# Trace plots
plot(code_image_imputed)
```

```
# Density plots
densityplot(code_image_imputed, ~bmi)
```

```
# Extract FMI/Lambda
pooled_diag_image <- code_image_imputed %>%
  with(glm(rct_status ~ bmi + age + sex + race_eth, family = binomial)) %>%
  pool()

mi_diagnostics_image <- pooled_diag_image$pooled %>%
  dplyr::select(term, riv, lambda, fmi) %>%
  mutate(across(where(is.numeric), ~round(., 4)))

print(mi_diagnostics_image)
```

```
##                         term    riv lambda    fmi
## 1                (Intercept) 0.0311 0.0301 0.0303
## 2                        bmi 0.4324 0.3018 0.3040
## 3                        age 0.0078 0.0077 0.0079
## 4                    sexMale 0.0016 0.0016 0.0017
## 5 race_ethBlack Non-Hispanic 0.0007 0.0007 0.0009
## 6           race_ethHispanic 0.0004 0.0004 0.0006
## 7              race_ethOther 0.0005 0.0005 0.0006
## 8 race_ethWhite Non-Hispanic 0.0004 0.0004 0.0005
```

interpretation after imputation: - ppm: predictive mean matching, for
continuous variables. - An empty string �� means that the variable was
not imputed. Because: 1) no missing data; 2) skipped by algorithm, often
due to perfect collinearity.

Trace Plot: - No Obvious Trends: For all three imputed variables
(bmi, hb\_a1c\_diag\_numeric, and time\_between\_bmi\_before), the lines move
horizontally across the plot without any clear upward or downward
pattern. - Good Mixing: The lines are tangled together in what is often
called a �fuzzy caterpillar,� which shows that the imputation process is
stable and the different imputed datasets are similar to each other.

Density Plot: - Unlike the NLP dataset, the imputed values here don�t
show as dramatic a shift. This suggests that in this cohort, the missing
BMI data is very similar in distribution to the observed data. - It
demonstrates that the MAR assumption is very well-supported here; the
model isn�t inventing a new distribution, but rather robustly filling in
the gaps using the existing clinical data structure.

FMI: - The FMI (Fraction of Missing Information) for BMI is 0.3040
(30.4%), and the lambda (\(\lambda\),
the proportion of total variance due to missingness) is 0.3018. - raw
BMI missingness was 34.0%, the imputation model(including covariates and
the outcome) successfully recovered information and outperformed a
simple complete-case analysis.

## create bmi\_cat

```
# Create `bmi_cat` within Imputed Datasets

# Convert the imputed object to a long-format data frame
long_df <- mice::complete(code_image_imputed, action = "long", include = TRUE)

# Add the original `rct_status` back
long_df$rct_status <- rep(code_image_df$rct_status, code_image_imputed$m + 1)

# Create `bmi_cat` and set the reference level
long_df_wrangled <- long_df %>%
  mutate(
    bmi_cat = case_when(
      bmi < 18.5          ~ "Underweight (<18.5)",
      bmi >= 18.5 & bmi < 25 ~ "Normal (18.5-24.9)",
      bmi >= 25   & bmi < 30 ~ "Overweight (25-29.9)",
      bmi >= 30           ~ "Obese (>=30)"
    ),
    # Convert to factor and set the reference group
    bmi_cat = fct_relevel(bmi_cat, "Normal (18.5-24.9)")
  )

# Convert the modified long data frame back into a `Multiply imputed data set (mids)` object for analysis
code_image_final_mids <- as.mids(long_df_wrangled)
```

## pooled analysis

ref: Control (Code Image)

```
# run each model on the imputed data and pool the results.
# `tbl_regression` automatically understands pooled results and formats them.
# The `variable` argument is used to select the predictors we are interested in.
library(gtsummary)

run_and_format_image <- function(mids_obj, formula, include_vars) {
  with(mids_obj, glm(as.formula(formula), family = binomial)) %>%
    tbl_regression(exponentiate = TRUE) %>%
    modify_table_body(~.x %>% filter(variable %in% include_vars)) %>%
    add_n(location = "level")
}

cat("### Model 1: RCT Status ~ Any Diabetes (Adjusted)")
```

```
## ### Model 1: RCT Status ~ Any Diabetes (Adjusted)
```

```
run_and_format_image(code_image_final_mids, "rct_status ~ dm_any + age + sex + race_eth + bmi", c("dm_any", "bmi"))
```

| **Characteristic** | **N** | **OR** | **95% CI** | **p-value** |
| --- | --- | --- | --- | --- |
| dm\_any |  |  |  |  |
| ����None | 11,095 | � | � |  |
| ����Any | 2,065 | 1.06 | 0.94, 1.19 | 0.4 |
| bmi | 13,160 | 1.02 | 1.01, 1.03 | <0.001 |
|  |  |  |  |  |
| --- | --- | --- | --- | --- |
| Abbreviations: CI = Confidence Interval, OR = Odds Ratio | | | | |

```
cat("\n### Model 2: RCT Status ~ T1 Diabetes (Adjusted)")
```

```
## 
## ### Model 2: RCT Status ~ T1 Diabetes (Adjusted)
```

```
run_and_format_image(code_image_final_mids, "rct_status ~ t1d + age + sex + race_eth + bmi", c("t1d", "bmi"))
```

| **Characteristic** | **N** | **OR** | **95% CI** | **p-value** |
| --- | --- | --- | --- | --- |
| t1d |  |  |  |  |
| ����No | 11,095 | � | � |  |
| ����Yes | 465 | 0.87 | 0.68, 1.11 | 0.3 |
| bmi | 11,560 | 1.02 | 1.01, 1.03 | <0.001 |
|  |  |  |  |  |
| --- | --- | --- | --- | --- |
| Abbreviations: CI = Confidence Interval, OR = Odds Ratio | | | | |

```
cat("\n### Model 3: RCT Status ~ T2 Diabetes (Adjusted)")
```

```
## 
## ### Model 3: RCT Status ~ T2 Diabetes (Adjusted)
```

```
run_and_format_image(code_image_final_mids, "rct_status ~ t2d + age + sex + race_eth + bmi", c("t2d", "bmi"))
```

| **Characteristic** | **N** | **OR** | **95% CI** | **p-value** |
| --- | --- | --- | --- | --- |
| t2d |  |  |  |  |
| ����No | 11,095 | � | � |  |
| ����Yes | 897 | 1.23 | 1.04, 1.44 | 0.014 |
| bmi | 11,992 | 1.02 | 1.01, 1.03 | <0.001 |
|  |  |  |  |  |
| --- | --- | --- | --- | --- |
| Abbreviations: CI = Confidence Interval, OR = Odds Ratio | | | | |

```
cat("\n### Model 4: RCT Status ~ BMI (Unadjusted)")
```

```
## 
## ### Model 4: RCT Status ~ BMI (Unadjusted)
```

```
run_and_format_image(code_image_final_mids, "rct_status ~ bmi", "bmi")
```

| **Characteristic** | **N** | **OR** | **95% CI** | **p-value** |
| --- | --- | --- | --- | --- |
| bmi | 13,160 | 1.01 | 1.00, 1.02 | 0.011 |
|  |  |  |  |  |
| --- | --- | --- | --- | --- |
| Abbreviations: CI = Confidence Interval, OR = Odds Ratio | | | | |

```
cat("\n### Model 5: RCT Status ~ BMI (Adjusted)")
```

```
## 
## ### Model 5: RCT Status ~ BMI (Adjusted)
```

```
run_and_format_image(code_image_final_mids, "rct_status ~ bmi + age + sex + race_eth", "bmi")
```

| **Characteristic** | **N** | **OR** | **95% CI** | **p-value** |
| --- | --- | --- | --- | --- |
| bmi | 13,160 | 1.02 | 1.01, 1.03 | <0.001 |
|  |  |  |  |  |
| --- | --- | --- | --- | --- |
| Abbreviations: CI = Confidence Interval, OR = Odds Ratio | | | | |

```
cat("\n### Model 6: RCT Status ~ BMI Category (Unadjusted)")
```

```
## 
## ### Model 6: RCT Status ~ BMI Category (Unadjusted)
```

```
run_and_format_image(code_image_final_mids, "rct_status ~ bmi_cat", "bmi_cat")
```

| **Characteristic** | **N** | **OR** | **95% CI** | **p-value** |
| --- | --- | --- | --- | --- |
| bmi\_cat |  |  |  |  |
| ����Normal (18.5-24.9) | 3,336 | � | � |  |
| ����Obese (>=30) | 4,983 | 1.32 | 1.15, 1.51 | <0.001 |
| ����Overweight (25-29.9) | 4,623 | 1.30 | 1.14, 1.49 | <0.001 |
| ����Underweight (<18.5) | 218 | 0.66 | 0.39, 1.11 | 0.12 |
|  |  |  |  |  |
| --- | --- | --- | --- | --- |
| Abbreviations: CI = Confidence Interval, OR = Odds Ratio | | | | |

```
cat("\n### Model 7: RCT Status ~ BMI Category (Adjusted)")
```

```
## 
## ### Model 7: RCT Status ~ BMI Category (Adjusted)
```

```
run_and_format_image(code_image_final_mids, "rct_status ~ bmi_cat + age + sex + race_eth", "bmi_cat")
```

| **Characteristic** | **N** | **OR** | **95% CI** | **p-value** |
| --- | --- | --- | --- | --- |
| bmi\_cat |  |  |  |  |
| ����Normal (18.5-24.9) | 3,336 | � | � |  |
| ����Obese (>=30) | 4,983 | 1.44 | 1.25, 1.65 | <0.001 |
| ����Overweight (25-29.9) | 4,623 | 1.31 | 1.14, 1.50 | <0.001 |
| ����Underweight (<18.5) | 218 | 0.65 | 0.38, 1.12 | 0.12 |
|  |  |  |  |  |
| --- | --- | --- | --- | --- |
| Abbreviations: CI = Confidence Interval, OR = Odds Ratio | | | | |

```
cat("\n### Model 8: RCT Status ~ BMI Category (Adjusted + T1D)")
```

```
## 
## ### Model 8: RCT Status ~ BMI Category (Adjusted + T1D)
```

```
run_and_format_image(code_image_final_mids, "rct_status ~ bmi_cat + age + sex + race_eth + t1d", c("t1d", "bmi_cat"))
```

| **Characteristic** | **N** | **OR** | **95% CI** | **p-value** |
| --- | --- | --- | --- | --- |
| bmi\_cat |  |  |  |  |
| ����Normal (18.5-24.9) | 3,096 | � | � |  |
| ����Obese (>=30) | 4,078 | 1.43 | 1.23, 1.66 | <0.001 |
| ����Overweight (25-29.9) | 4,178 | 1.32 | 1.14, 1.52 | <0.001 |
| ����Underweight (<18.5) | 208 | 0.69 | 0.40, 1.18 | 0.2 |
| t1d |  |  |  |  |
| ����No | 11,095 | � | � |  |
| ����Yes | 465 | 0.88 | 0.69, 1.13 | 0.3 |
|  |  |  |  |  |
| --- | --- | --- | --- | --- |
| Abbreviations: CI = Confidence Interval, OR = Odds Ratio | | | | |

# code\_non\_image\_df: Multiple Imputation for bmi

- outcome: rct\_status
- do not include: distinct\_visit\_cnt, bmi\_cat (derivative of bmi)
- set imputed dataset m to be at least the percentage of missing
  cases.

## abnormal bmi and bmi\_cat

```
library(tidyverse)

mismatched_bmi <- code_non_image_df %>%
  # create a new column with the CORRECT category based on the bmi value
  mutate(
    correct_bmi_cat = case_when(
      bmi < 18.5          ~ "Underweight (<18.5)",
      bmi >= 18.5 & bmi < 25 ~ "Normal (18.5-24.9)",
      bmi >= 25   & bmi < 30 ~ "Overweight (25-29.9)",
      bmi >= 30           ~ "Obese (>30)",
      TRUE                ~ NA_character_
    )
  ) %>%
  # filter for rows that meet any of error conditions
  filter(
    # Condition 1: The original category doesn't match the correct one
    (bmi_cat != correct_bmi_cat & !is.na(bmi)) |
    # Condition 2: The BMI value is biologically implausible
    (bmi > 150 | bmi < 10)
  ) %>%
  # Select relevant columns
  dplyr::select(rct_status, age, sex, bmi, bmi_cat, correct_bmi_cat)


print(mismatched_bmi)
```

```
## # A tibble: 36 � 6
##    rct_status                 age sex      bmi bmi_cat              correct_bmi_cat     
##    <fct>                    <dbl> <fct>  <dbl> <fct>                <chr>               
##  1 Case (Code Non Image)     73.4 Female    25 Normal (18.5-24.9)   Overweight (25-29.9)
##  2 Case (Code Non Image)     48.9 Male      25 Normal (18.5-24.9)   Overweight (25-29.9)
##  3 Case (Code Non Image)     53.1 Female    30 Overweight (25-29.9) Obese (>30)         
##  4 Control (Code Non Image)  41.8 Male      30 Overweight (25-29.9) Obese (>30)         
##  5 Control (Code Non Image)  71.8 Female    30 Overweight (25-29.9) Obese (>30)         
##  6 Control (Code Non Image)  71.8 Female    25 Normal (18.5-24.9)   Overweight (25-29.9)
##  7 Case (Code Non Image)     66.1 Female    25 Normal (18.5-24.9)   Overweight (25-29.9)
##  8 Control (Code Non Image)  41.9 Female    30 Overweight (25-29.9) Obese (>30)         
##  9 Case (Code Non Image)     64.6 Male      25 Normal (18.5-24.9)   Overweight (25-29.9)
## 10 Case (Code Non Image)     61.5 Female    30 Overweight (25-29.9) Obese (>30)         
## # ? 26 more rows
```

```
# Get a count of how many mismatches were found
cat(glue::glue("\nFound {nrow(mismatched_bmi)} rows with BMI mismatches or implausible values."))
```

```
## Found 36 rows with BMI mismatches or implausible values.
```

```
# Set implausible BMI values to NA
code_non_image_df <- code_non_image_df %>%
  mutate(
    bmi = if_else(bmi > 150 | bmi < 10, NA_real_, bmi)
  )
```

- Biologically implausible BMI values (bmi >150 or <10) were
  recoded as missing (NA) to be properly handled during the multiple
  imputation process.
- note: The BMI categories may need redefine to align with CDC/WHO
  guidelines. Specifically, boundary values are now included in the higher
  category; for example, a BMI of 30.0 is classified as �Obese (?30)�
  rather than �Overweight�; a BMI of 25.0 is classified as �Overweight
  (25-29.9)� rather than �Normal (18.5-24.9)�.

## check missingness in bmi

```
library(tidyverse)
library(naniar)

str(code_non_image_df)
```

```
## tibble [42,410 � 17] (S3: tbl_df/tbl/data.frame)
##  $ rct_status             : Factor w/ 2 levels "Control (Code Non Image)",..: 1 2 2 1 1 1 1 1 2 1 ...
##  $ sex                    : Factor w/ 3 levels "Female","Male",..: 2 2 2 2 2 2 2 1 2 2 ...
##  $ age                    : num [1:42410] 51.5 79.6 63 73 54.2 ...
##  $ race_eth               : Factor w/ 5 levels "Asian Non-Hispanic",..: 5 5 4 4 2 5 5 5 4 5 ...
##  $ bmi                    : num [1:42410] 29.8 NA 24.6 NA NA ...
##  $ bmi_cat                : Factor w/ 4 levels "Normal (18.5-24.9)",..: 3 NA 1 NA NA NA 3 NA 3 NA ...
##  $ t1d                    : Factor w/ 2 levels "No","Yes": 1 1 1 1 1 1 1 1 1 1 ...
##  $ t2d                    : Factor w/ 2 levels "No","Yes": 1 1 1 1 1 1 1 1 1 1 ...
##  $ dm_any                 : Factor w/ 2 levels "None","Any": 1 1 1 1 1 1 1 1 1 1 ...
##  $ hb_a1c_diag_numeric    : num [1:42410] NA NA NA NA NA NA NA NA NA NA ...
##  $ hpt_at_1st_diag_code   : Factor w/ 2 levels "Yes","No": 2 2 2 2 2 2 1 2 1 2 ...
##  $ hpt_at_1st_diag_meds   : Factor w/ 2 levels "Yes","No": 2 1 2 2 2 2 1 2 1 2 ...
##  $ ligamentous_disease    : Factor w/ 2 levels "Yes","No": 2 2 2 2 2 2 2 2 1 2 ...
##  $ prior_injury           : Factor w/ 2 levels "Yes","No": 2 1 1 2 2 2 2 2 1 2 ...
##  $ arthritis              : Factor w/ 2 levels "Yes","No": 2 1 1 2 2 2 2 2 1 2 ...
##  $ distinct_visit_cnt     : int [1:42410] 4 18 3 1 9 1 16 1 42 2 ...
##  $ time_between_bmi_before: int [1:42410] -8 -1311 -7 NA NA NA -26 NA -77 NA ...
```

```
miss_var_summary(code_non_image_df)
```

```
## # A tibble: 17 � 3
##    variable                n_miss pct_miss
##    <chr>                    <int>    <num>
##  1 hb_a1c_diag_numeric      38835  91.6   
##  2 bmi                      24551  57.9   
##  3 bmi_cat                  24551  57.9   
##  4 time_between_bmi_before  23012  54.3   
##  5 t1d                       3118   7.35  
##  6 t2d                       2051   4.84  
##  7 distinct_visit_cnt          12   0.0283
##  8 rct_status                   0   0     
##  9 sex                          0   0     
## 10 age                          0   0     
## 11 race_eth                     0   0     
## 12 dm_any                       0   0     
## 13 hpt_at_1st_diag_code         0   0     
## 14 hpt_at_1st_diag_meds         0   0     
## 15 ligamentous_disease          0   0     
## 16 prior_injury                 0   0     
## 17 arthritis                    0   0
```

```
# Set "Control (Code Non Image)" as the reference level for the outcome variable
code_non_image_df <- code_non_image_df %>%
  mutate(rct_status = fct_relevel(rct_status, "Control (Code Non Image)"))
```

## imputation

```
# Prepare Imputation Data (rct_status is KEPT in the data)
vars_for_imputation <- setdiff(names(code_non_image_df), c("distinct_visit_cnt", "bmi_cat"))

code_non_image_impute_data <- code_non_image_df %>%
  dplyr::select(all_of(vars_for_imputation))

# Configure MICE
# Ensure the length of method matches the columns exactly
meth <- make.method(code_non_image_impute_data)
meth["rct_status"] <- "" # Do not impute the outcome
pred <- make.predictorMatrix(code_non_image_impute_data)

# Run MICE algorithm (Using m=60 per your missingness check)
set.seed(123)
code_non_image_imputed <- mice(code_non_image_impute_data, 
                               method = meth, 
                               predictorMatrix = pred, 
                               m = 65, 
                               maxit = 10, 
                               printFlag = TRUE)
```

```
## 
##  iter imp variable
##   1   1  bmi  hb_a1c_diag_numeric  time_between_bmi_before
##   1   2  bmi  hb_a1c_diag_numeric  time_between_bmi_before
##   1   3  bmi  hb_a1c_diag_numeric  time_between_bmi_before
##   1   4  bmi  hb_a1c_diag_numeric  time_between_bmi_before
##   1   5  bmi  hb_a1c_diag_numeric  time_between_bmi_before
##   1   6  bmi  hb_a1c_diag_numeric  time_between_bmi_before
##   1   7  bmi  hb_a1c_diag_numeric  time_between_bmi_before
##   1   8  bmi  hb_a1c_diag_numeric  time_between_bmi_before
##   1   9  bmi  hb_a1c_diag_numeric  time_between_bmi_before
##   1   10  bmi  hb_a1c_diag_numeric  time_between_bmi_before
##   1   11  bmi  hb_a1c_diag_numeric  time_between_bmi_before
##   1   12  bmi  hb_a1c_diag_numeric  time_between_bmi_before
##   1   13  bmi  hb_a1c_diag_numeric  time_between_bmi_before
##   1   14  bmi  hb_a1c_diag_numeric  time_between_bmi_before
##   1   15  bmi  hb_a1c_diag_numeric  time_between_bmi_before
##   1   16  bmi  hb_a1c_diag_numeric  time_between_bmi_before
##   1   17  bmi  hb_a1c_diag_numeric  time_between_bmi_before
##   1   18  bmi  hb_a1c_diag_numeric  time_between_bmi_before
##   1   19  bmi  hb_a1c_diag_numeric  time_between_bmi_before
##   1   20  bmi  hb_a1c_diag_numeric  time_between_bmi_before
##   1   21  bmi  hb_a1c_diag_numeric  time_between_bmi_before
##   1   22  bmi  hb_a1c_diag_numeric  time_between_bmi_before
##   1   23  bmi  hb_a1c_diag_numeric  time_between_bmi_before
##   1   24  bmi  hb_a1c_diag_numeric  time_between_bmi_before
##   1   25  bmi  hb_a1c_diag_numeric  time_between_bmi_before
##   1   26  bmi  hb_a1c_diag_numeric  time_between_bmi_before
##   1   27  bmi  hb_a1c_diag_numeric  time_between_bmi_before
##   1   28  bmi  hb_a1c_diag_numeric  time_between_bmi_before
##   1   29  bmi  hb_a1c_diag_numeric  time_between_bmi_before
##   1   30  bmi  hb_a1c_diag_numeric  time_between_bmi_before
##   1   31  bmi  hb_a1c_diag_numeric  time_between_bmi_before
##   1   32  bmi  hb_a1c_diag_numeric  time_between_bmi_before
##   1   33  bmi  hb_a1c_diag_numeric  time_between_bmi_before
##   1   34  bmi  hb_a1c_diag_numeric  time_between_bmi_before
##   1   35  bmi  hb_a1c_diag_numeric  time_between_bmi_before
##   1   36  bmi  hb_a1c_diag_numeric  time_between_bmi_before
##   1   37  bmi  hb_a1c_diag_numeric  time_between_bmi_before
##   1   38  bmi  hb_a1c_diag_numeric  time_between_bmi_before
##   1   39  bmi  hb_a1c_diag_numeric  time_between_bmi_before
##   1   40  bmi  hb_a1c_diag_numeric  time_between_bmi_before
##   1   41  bmi  hb_a1c_diag_numeric  time_between_bmi_before
##   1   42  bmi  hb_a1c_diag_numeric  time_between_bmi_before
##   1   43  bmi  hb_a1c_diag_numeric  time_between_bmi_before
##   1   44  bmi  hb_a1c_diag_numeric  time_between_bmi_before
##   1   45  bmi  hb_a1c_diag_numeric  time_between_bmi_before
##   1   46  bmi  hb_a1c_diag_numeric  time_between_bmi_before
##   1   47  bmi  hb_a1c_diag_numeric  time_between_bmi_before
##   1   48  bmi  hb_a1c_diag_numeric  time_between_bmi_before
##   1   49  bmi  hb_a1c_diag_numeric  time_between_bmi_before
##   1   50  bmi  hb_a1c_diag_numeric  time_between_bmi_before
##   1   51  bmi  hb_a1c_diag_numeric  time_between_bmi_before
##   1   52  bmi  hb_a1c_diag_numeric  time_between_bmi_before
##   1   53  bmi  hb_a1c_diag_numeric  time_between_bmi_before
##   1   54  bmi  hb_a1c_diag_numeric  time_between_bmi_before
##   1   55  bmi  hb_a1c_diag_numeric  time_between_bmi_before
##   1   56  bmi  hb_a1c_diag_numeric  time_between_bmi_before
##   1   57  bmi  hb_a1c_diag_numeric  time_between_bmi_before
##   1   58  bmi  hb_a1c_diag_numeric  time_between_bmi_before
##   1   59  bmi  hb_a1c_diag_numeric  time_between_bmi_before
##   1   60  bmi  hb_a1c_diag_numeric  time_between_bmi_before
##   1   61  bmi  hb_a1c_diag_numeric  time_between_bmi_before
##   1   62  bmi  hb_a1c_diag_numeric  time_between_bmi_before
##   1   63  bmi  hb_a1c_diag_numeric  time_between_bmi_before
##   1   64  bmi  hb_a1c_diag_numeric  time_between_bmi_before
##   1   65  bmi  hb_a1c_diag_numeric  time_between_bmi_before
##   2   1  bmi  hb_a1c_diag_numeric  time_between_bmi_before
##   2   2  bmi  hb_a1c_diag_numeric  time_between_bmi_before
##   2   3  bmi  hb_a1c_diag_numeric  time_between_bmi_before
##   2   4  bmi  hb_a1c_diag_numeric  time_between_bmi_before
##   2   5  bmi  hb_a1c_diag_numeric  time_between_bmi_before
##   2   6  bmi  hb_a1c_diag_numeric  time_between_bmi_before
##   2   7  bmi  hb_a1c_diag_numeric  time_between_bmi_before
##   2   8  bmi  hb_a1c_diag_numeric  time_between_bmi_before
##   2   9  bmi  hb_a1c_diag_numeric  time_between_bmi_before
##   2   10  bmi  hb_a1c_diag_numeric  time_between_bmi_before
##   2   11  bmi  hb_a1c_diag_numeric  time_between_bmi_before
##   2   12  bmi  hb_a1c_diag_numeric  time_between_bmi_before
##   2   13  bmi  hb_a1c_diag_numeric  time_between_bmi_before
##   2   14  bmi  hb_a1c_diag_numeric  time_between_bmi_before
##   2   15  bmi  hb_a1c_diag_numeric  time_between_bmi_before
##   2   16  bmi  hb_a1c_diag_numeric  time_between_bmi_before
##   2   17  bmi  hb_a1c_diag_numeric  time_between_bmi_before
##   2   18  bmi  hb_a1c_diag_numeric  time_between_bmi_before
##   2   19  bmi  hb_a1c_diag_numeric  time_between_bmi_before
##   2   20  bmi  hb_a1c_diag_numeric  time_between_bmi_before
##   2   21  bmi  hb_a1c_diag_numeric  time_between_bmi_before
##   2   22  bmi  hb_a1c_diag_numeric  time_between_bmi_before
##   2   23  bmi  hb_a1c_diag_numeric  time_between_bmi_before
##   2   24  bmi  hb_a1c_diag_numeric  time_between_bmi_before
##   2   25  bmi  hb_a1c_diag_numeric  time_between_bmi_before
##   2   26  bmi  hb_a1c_diag_numeric  time_between_bmi_before
##   2   27  bmi  hb_a1c_diag_numeric  time_between_bmi_before
##   2   28  bmi  hb_a1c_diag_numeric  time_between_bmi_before
##   2   29  bmi  hb_a1c_diag_numeric  time_between_bmi_before
##   2   30  bmi  hb_a1c_diag_numeric  time_between_bmi_before
##   2   31  bmi  hb_a1c_diag_numeric  time_between_bmi_before
##   2   32  bmi  hb_a1c_diag_numeric  time_between_bmi_before
##   2   33  bmi  hb_a1c_diag_numeric  time_between_bmi_before
##   2   34  bmi  hb_a1c_diag_numeric  time_between_bmi_before
##   2   35  bmi  hb_a1c_diag_numeric  time_between_bmi_before
##   2   36  bmi  hb_a1c_diag_numeric  time_between_bmi_before
##   2   37  bmi  hb_a1c_diag_numeric  time_between_bmi_before
##   2   38  bmi  hb_a1c_diag_numeric  time_between_bmi_before
##   2   39  bmi  hb_a1c_diag_numeric  time_between_bmi_before
##   2   40  bmi  hb_a1c_diag_numeric  time_between_bmi_before
##   2   41  bmi  hb_a1c_diag_numeric  time_between_bmi_before
##   2   42  bmi  hb_a1c_diag_numeric  time_between_bmi_before
##   2   43  bmi  hb_a1c_diag_numeric  time_between_bmi_before
##   2   44  bmi  hb_a1c_diag_numeric  time_between_bmi_before
##   2   45  bmi  hb_a1c_diag_numeric  time_between_bmi_before
##   2   46  bmi  hb_a1c_diag_numeric  time_between_bmi_before
##   2   47  bmi  hb_a1c_diag_numeric  time_between_bmi_before
##   2   48  bmi  hb_a1c_diag_numeric  time_between_bmi_before
##   2   49  bmi  hb_a1c_diag_numeric  time_between_bmi_before
##   2   50  bmi  hb_a1c_diag_numeric  time_between_bmi_before
##   2   51  bmi  hb_a1c_diag_numeric  time_between_bmi_before
##   2   52  bmi  hb_a1c_diag_numeric  time_between_bmi_before
##   2   53  bmi  hb_a1c_diag_numeric  time_between_bmi_before
##   2   54  bmi  hb_a1c_diag_numeric  time_between_bmi_before
##   2   55  bmi  hb_a1c_diag_numeric  time_between_bmi_before
##   2   56  bmi  hb_a1c_diag_numeric  time_between_bmi_before
##   2   57  bmi  hb_a1c_diag_numeric  time_between_bmi_before
##   2   58  bmi  hb_a1c_diag_numeric  time_between_bmi_before
##   2   59  bmi  hb_a1c_diag_numeric  time_between_bmi_before
##   2   60  bmi  hb_a1c_diag_numeric  time_between_bmi_before
##   2   61  bmi  hb_a1c_diag_numeric  time_between_bmi_before
##   2   62  bmi  hb_a1c_diag_numeric  time_between_bmi_before
##   2   63  bmi  hb_a1c_diag_numeric  time_between_bmi_before
##   2   64  bmi  hb_a1c_diag_numeric  time_between_bmi_before
##   2   65  bmi  hb_a1c_diag_numeric  time_between_bmi_before
##   3   1  bmi  hb_a1c_diag_numeric  time_between_bmi_before
##   3   2  bmi  hb_a1c_diag_numeric  time_between_bmi_before
##   3   3  bmi  hb_a1c_diag_numeric  time_between_bmi_before
##   3   4  bmi  hb_a1c_diag_numeric  time_between_bmi_before
##   3   5  bmi  hb_a1c_diag_numeric  time_between_bmi_before
##   3   6  bmi  hb_a1c_diag_numeric  time_between_bmi_before
##   3   7  bmi  hb_a1c_diag_numeric  time_between_bmi_before
##   3   8  bmi  hb_a1c_diag_numeric  time_between_bmi_before
##   3   9  bmi  hb_a1c_diag_numeric  time_between_bmi_before
##   3   10  bmi  hb_a1c_diag_numeric  time_between_bmi_before
##   3   11  bmi  hb_a1c_diag_numeric  time_between_bmi_before
##   3   12  bmi  hb_a1c_diag_numeric  time_between_bmi_before
##   3   13  bmi  hb_a1c_diag_numeric  time_between_bmi_before
##   3   14  bmi  hb_a1c_diag_numeric  time_between_bmi_before
##   3   15  bmi  hb_a1c_diag_numeric  time_between_bmi_before
##   3   16  bmi  hb_a1c_diag_numeric  time_between_bmi_before
##   3   17  bmi  hb_a1c_diag_numeric  time_between_bmi_before
##   3   18  bmi  hb_a1c_diag_numeric  time_between_bmi_before
##   3   19  bmi  hb_a1c_diag_numeric  time_between_bmi_before
##   3   20  bmi  hb_a1c_diag_numeric  time_between_bmi_before
##   3   21  bmi  hb_a1c_diag_numeric  time_between_bmi_before
##   3   22  bmi  hb_a1c_diag_numeric  time_between_bmi_before
##   3   23  bmi  hb_a1c_diag_numeric  time_between_bmi_before
##   3   24  bmi  hb_a1c_diag_numeric  time_between_bmi_before
##   3   25  bmi  hb_a1c_diag_numeric  time_between_bmi_before
##   3   26  bmi  hb_a1c_diag_numeric  time_between_bmi_before
##   3   27  bmi  hb_a1c_diag_numeric  time_between_bmi_before
##   3   28  bmi  hb_a1c_diag_numeric  time_between_bmi_before
##   3   29  bmi  hb_a1c_diag_numeric  time_between_bmi_before
##   3   30  bmi  hb_a1c_diag_numeric  time_between_bmi_before
##   3   31  bmi  hb_a1c_diag_numeric  time_between_bmi_before
##   3   32  bmi  hb_a1c_diag_numeric  time_between_bmi_before
##   3   33  bmi  hb_a1c_diag_numeric  time_between_bmi_before
##   3   34  bmi  hb_a1c_diag_numeric  time_between_bmi_before
##   3   35  bmi  hb_a1c_diag_numeric  time_between_bmi_before
##   3   36  bmi  hb_a1c_diag_numeric  time_between_bmi_before
##   3   37  bmi  hb_a1c_diag_numeric  time_between_bmi_before
##   3   38  bmi  hb_a1c_diag_numeric  time_between_bmi_before
##   3   39  bmi  hb_a1c_diag_numeric  time_between_bmi_before
##   3   40  bmi  hb_a1c_diag_numeric  time_between_bmi_before
##   3   41  bmi  hb_a1c_diag_numeric  time_between_bmi_before
##   3   42  bmi  hb_a1c_diag_numeric  time_between_bmi_before
##   3   43  bmi  hb_a1c_diag_numeric  time_between_bmi_before
##   3   44  bmi  hb_a1c_diag_numeric  time_between_bmi_before
##   3   45  bmi  hb_a1c_diag_numeric  time_between_bmi_before
##   3   46  bmi  hb_a1c_diag_numeric  time_between_bmi_before
##   3   47  bmi  hb_a1c_diag_numeric  time_between_bmi_before
##   3   48  bmi  hb_a1c_diag_numeric  time_between_bmi_before
##   3   49  bmi  hb_a1c_diag_numeric  time_between_bmi_before
##   3   50  bmi  hb_a1c_diag_numeric  time_between_bmi_before
##   3   51  bmi  hb_a1c_diag_numeric  time_between_bmi_before
##   3   52  bmi  hb_a1c_diag_numeric  time_between_bmi_before
##   3   53  bmi  hb_a1c_diag_numeric  time_between_bmi_before
##   3   54  bmi  hb_a1c_diag_numeric  time_between_bmi_before
##   3   55  bmi  hb_a1c_diag_numeric  time_between_bmi_before
##   3   56  bmi  hb_a1c_diag_numeric  time_between_bmi_before
##   3   57  bmi  hb_a1c_diag_numeric  time_between_bmi_before
##   3   58  bmi  hb_a1c_diag_numeric  time_between_bmi_before
##   3   59  bmi  hb_a1c_diag_numeric  time_between_bmi_before
##   3   60  bmi  hb_a1c_diag_numeric  time_between_bmi_before
##   3   61  bmi  hb_a1c_diag_numeric  time_between_bmi_before
##   3   62  bmi  hb_a1c_diag_numeric  time_between_bmi_before
##   3   63  bmi  hb_a1c_diag_numeric  time_between_bmi_before
##   3   64  bmi  hb_a1c_diag_numeric  time_between_bmi_before
##   3   65  bmi  hb_a1c_diag_numeric  time_between_bmi_before
##   4   1  bmi  hb_a1c_diag_numeric  time_between_bmi_before
##   4   2  bmi  hb_a1c_diag_numeric  time_between_bmi_before
##   4   3  bmi  hb_a1c_diag_numeric  time_between_bmi_before
##   4   4  bmi  hb_a1c_diag_numeric  time_between_bmi_before
##   4   5  bmi  hb_a1c_diag_numeric  time_between_bmi_before
##   4   6  bmi  hb_a1c_diag_numeric  time_between_bmi_before
##   4   7  bmi  hb_a1c_diag_numeric  time_between_bmi_before
##   4   8  bmi  hb_a1c_diag_numeric  time_between_bmi_before
##   4   9  bmi  hb_a1c_diag_numeric  time_between_bmi_before
##   4   10  bmi  hb_a1c_diag_numeric  time_between_bmi_before
##   4   11  bmi  hb_a1c_diag_numeric  time_between_bmi_before
##   4   12  bmi  hb_a1c_diag_numeric  time_between_bmi_before
##   4   13  bmi  hb_a1c_diag_numeric  time_between_bmi_before
##   4   14  bmi  hb_a1c_diag_numeric  time_between_bmi_before
##   4   15  bmi  hb_a1c_diag_numeric  time_between_bmi_before
##   4   16  bmi  hb_a1c_diag_numeric  time_between_bmi_before
##   4   17  bmi  hb_a1c_diag_numeric  time_between_bmi_before
##   4   18  bmi  hb_a1c_diag_numeric  time_between_bmi_before
##   4   19  bmi  hb_a1c_diag_numeric  time_between_bmi_before
##   4   20  bmi  hb_a1c_diag_numeric  time_between_bmi_before
##   4   21  bmi  hb_a1c_diag_numeric  time_between_bmi_before
##   4   22  bmi  hb_a1c_diag_numeric  time_between_bmi_before
##   4   23  bmi  hb_a1c_diag_numeric  time_between_bmi_before
##   4   24  bmi  hb_a1c_diag_numeric  time_between_bmi_before
##   4   25  bmi  hb_a1c_diag_numeric  time_between_bmi_before
##   4   26  bmi  hb_a1c_diag_numeric  time_between_bmi_before
##   4   27  bmi  hb_a1c_diag_numeric  time_between_bmi_before
##   4   28  bmi  hb_a1c_diag_numeric  time_between_bmi_before
##   4   29  bmi  hb_a1c_diag_numeric  time_between_bmi_before
##   4   30  bmi  hb_a1c_diag_numeric  time_between_bmi_before
##   4   31  bmi  hb_a1c_diag_numeric  time_between_bmi_before
##   4   32  bmi  hb_a1c_diag_numeric  time_between_bmi_before
##   4   33  bmi  hb_a1c_diag_numeric  time_between_bmi_before
##   4   34  bmi  hb_a1c_diag_numeric  time_between_bmi_before
##   4   35  bmi  hb_a1c_diag_numeric  time_between_bmi_before
##   4   36  bmi  hb_a1c_diag_numeric  time_between_bmi_before
##   4   37  bmi  hb_a1c_diag_numeric  time_between_bmi_before
##   4   38  bmi  hb_a1c_diag_numeric  time_between_bmi_before
##   4   39  bmi  hb_a1c_diag_numeric  time_between_bmi_before
##   4   40  bmi  hb_a1c_diag_numeric  time_between_bmi_before
##   4   41  bmi  hb_a1c_diag_numeric  time_between_bmi_before
##   4   42  bmi  hb_a1c_diag_numeric  time_between_bmi_before
##   4   43  bmi  hb_a1c_diag_numeric  time_between_bmi_before
##   4   44  bmi  hb_a1c_diag_numeric  time_between_bmi_before
##   4   45  bmi  hb_a1c_diag_numeric  time_between_bmi_before
##   4   46  bmi  hb_a1c_diag_numeric  time_between_bmi_before
##   4   47  bmi  hb_a1c_diag_numeric  time_between_bmi_before
##   4   48  bmi  hb_a1c_diag_numeric  time_between_bmi_before
##   4   49  bmi  hb_a1c_diag_numeric  time_between_bmi_before
##   4   50  bmi  hb_a1c_diag_numeric  time_between_bmi_before
##   4   51  bmi  hb_a1c_diag_numeric  time_between_bmi_before
##   4   52  bmi  hb_a1c_diag_numeric  time_between_bmi_before
##   4   53  bmi  hb_a1c_diag_numeric  time_between_bmi_before
##   4   54  bmi  hb_a1c_diag_numeric  time_between_bmi_before
##   4   55  bmi  hb_a1c_diag_numeric  time_between_bmi_before
##   4   56  bmi  hb_a1c_diag_numeric  time_between_bmi_before
##   4   57  bmi  hb_a1c_diag_numeric  time_between_bmi_before
##   4   58  bmi  hb_a1c_diag_numeric  time_between_bmi_before
##   4   59  bmi  hb_a1c_diag_numeric  time_between_bmi_before
##   4   60  bmi  hb_a1c_diag_numeric  time_between_bmi_before
##   4   61  bmi  hb_a1c_diag_numeric  time_between_bmi_before
##   4   62  bmi  hb_a1c_diag_numeric  time_between_bmi_before
##   4   63  bmi  hb_a1c_diag_numeric  time_between_bmi_before
##   4   64  bmi  hb_a1c_diag_numeric  time_between_bmi_before
##   4   65  bmi  hb_a1c_diag_numeric  time_between_bmi_before
##   5   1  bmi  hb_a1c_diag_numeric  time_between_bmi_before
##   5   2  bmi  hb_a1c_diag_numeric  time_between_bmi_before
##   5   3  bmi  hb_a1c_diag_numeric  time_between_bmi_before
##   5   4  bmi  hb_a1c_diag_numeric  time_between_bmi_before
##   5   5  bmi  hb_a1c_diag_numeric  time_between_bmi_before
##   5   6  bmi  hb_a1c_diag_numeric  time_between_bmi_before
##   5   7  bmi  hb_a1c_diag_numeric  time_between_bmi_before
##   5   8  bmi  hb_a1c_diag_numeric  time_between_bmi_before
##   5   9  bmi  hb_a1c_diag_numeric  time_between_bmi_before
##   5   10  bmi  hb_a1c_diag_numeric  time_between_bmi_before
##   5   11  bmi  hb_a1c_diag_numeric  time_between_bmi_before
##   5   12  bmi  hb_a1c_diag_numeric  time_between_bmi_before
##   5   13  bmi  hb_a1c_diag_numeric  time_between_bmi_before
##   5   14  bmi  hb_a1c_diag_numeric  time_between_bmi_before
##   5   15  bmi  hb_a1c_diag_numeric  time_between_bmi_before
##   5   16  bmi  hb_a1c_diag_numeric  time_between_bmi_before
##   5   17  bmi  hb_a1c_diag_numeric  time_between_bmi_before
##   5   18  bmi  hb_a1c_diag_numeric  time_between_bmi_before
##   5   19  bmi  hb_a1c_diag_numeric  time_between_bmi_before
##   5   20  bmi  hb_a1c_diag_numeric  time_between_bmi_before
##   5   21  bmi  hb_a1c_diag_numeric  time_between_bmi_before
##   5   22  bmi  hb_a1c_diag_numeric  time_between_bmi_before
##   5   23  bmi  hb_a1c_diag_numeric  time_between_bmi_before
##   5   24  bmi  hb_a1c_diag_numeric  time_between_bmi_before
##   5   25  bmi  hb_a1c_diag_numeric  time_between_bmi_before
##   5   26  bmi  hb_a1c_diag_numeric  time_between_bmi_before
##   5   27  bmi  hb_a1c_diag_numeric  time_between_bmi_before
##   5   28  bmi  hb_a1c_diag_numeric  time_between_bmi_before
##   5   29  bmi  hb_a1c_diag_numeric  time_between_bmi_before
##   5   30  bmi  hb_a1c_diag_numeric  time_between_bmi_before
##   5   31  bmi  hb_a1c_diag_numeric  time_between_bmi_before
##   5   32  bmi  hb_a1c_diag_numeric  time_between_bmi_before
##   5   33  bmi  hb_a1c_diag_numeric  time_between_bmi_before
##   5   34  bmi  hb_a1c_diag_numeric  time_between_bmi_before
##   5   35  bmi  hb_a1c_diag_numeric  time_between_bmi_before
##   5   36  bmi  hb_a1c_diag_numeric  time_between_bmi_before
##   5   37  bmi  hb_a1c_diag_numeric  time_between_bmi_before
##   5   38  bmi  hb_a1c_diag_numeric  time_between_bmi_before
##   5   39  bmi  hb_a1c_diag_numeric  time_between_bmi_before
##   5   40  bmi  hb_a1c_diag_numeric  time_between_bmi_before
##   5   41  bmi  hb_a1c_diag_numeric  time_between_bmi_before
##   5   42  bmi  hb_a1c_diag_numeric  time_between_bmi_before
##   5   43  bmi  hb_a1c_diag_numeric  time_between_bmi_before
##   5   44  bmi  hb_a1c_diag_numeric  time_between_bmi_before
##   5   45  bmi  hb_a1c_diag_numeric  time_between_bmi_before
##   5   46  bmi  hb_a1c_diag_numeric  time_between_bmi_before
##   5   47  bmi  hb_a1c_diag_numeric  time_between_bmi_before
##   5   48  bmi  hb_a1c_diag_numeric  time_between_bmi_before
##   5   49  bmi  hb_a1c_diag_numeric  time_between_bmi_before
##   5   50  bmi  hb_a1c_diag_numeric  time_between_bmi_before
##   5   51  bmi  hb_a1c_diag_numeric  time_between_bmi_before
##   5   52  bmi  hb_a1c_diag_numeric  time_between_bmi_before
##   5   53  bmi  hb_a1c_diag_numeric  time_between_bmi_before
##   5   54  bmi  hb_a1c_diag_numeric  time_between_bmi_before
##   5   55  bmi  hb_a1c_diag_numeric  time_between_bmi_before
##   5   56  bmi  hb_a1c_diag_numeric  time_between_bmi_before
##   5   57  bmi  hb_a1c_diag_numeric  time_between_bmi_before
##   5   58  bmi  hb_a1c_diag_numeric  time_between_bmi_before
##   5   59  bmi  hb_a1c_diag_numeric  time_between_bmi_before
##   5   60  bmi  hb_a1c_diag_numeric  time_between_bmi_before
##   5   61  bmi  hb_a1c_diag_numeric  time_between_bmi_before
##   5   62  bmi  hb_a1c_diag_numeric  time_between_bmi_before
##   5   63  bmi  hb_a1c_diag_numeric  time_between_bmi_before
##   5   64  bmi  hb_a1c_diag_numeric  time_between_bmi_before
##   5   65  bmi  hb_a1c_diag_numeric  time_between_bmi_before
##   6   1  bmi  hb_a1c_diag_numeric  time_between_bmi_before
##   6   2  bmi  hb_a1c_diag_numeric  time_between_bmi_before
##   6   3  bmi  hb_a1c_diag_numeric  time_between_bmi_before
##   6   4  bmi  hb_a1c_diag_numeric  time_between_bmi_before
##   6   5  bmi  hb_a1c_diag_numeric  time_between_bmi_before
##   6   6  bmi  hb_a1c_diag_numeric  time_between_bmi_before
##   6   7  bmi  hb_a1c_diag_numeric  time_between_bmi_before
##   6   8  bmi  hb_a1c_diag_numeric  time_between_bmi_before
##   6   9  bmi  hb_a1c_diag_numeric  time_between_bmi_before
##   6   10  bmi  hb_a1c_diag_numeric  time_between_bmi_before
##   6   11  bmi  hb_a1c_diag_numeric  time_between_bmi_before
##   6   12  bmi  hb_a1c_diag_numeric  time_between_bmi_before
##   6   13  bmi  hb_a1c_diag_numeric  time_between_bmi_before
##   6   14  bmi  hb_a1c_diag_numeric  time_between_bmi_before
##   6   15  bmi  hb_a1c_diag_numeric  time_between_bmi_before
##   6   16  bmi  hb_a1c_diag_numeric  time_between_bmi_before
##   6   17  bmi  hb_a1c_diag_numeric  time_between_bmi_before
##   6   18  bmi  hb_a1c_diag_numeric  time_between_bmi_before
##   6   19  bmi  hb_a1c_diag_numeric  time_between_bmi_before
##   6   20  bmi  hb_a1c_diag_numeric  time_between_bmi_before
##   6   21  bmi  hb_a1c_diag_numeric  time_between_bmi_before
##   6   22  bmi  hb_a1c_diag_numeric  time_between_bmi_before
##   6   23  bmi  hb_a1c_diag_numeric  time_between_bmi_before
##   6   24  bmi  hb_a1c_diag_numeric  time_between_bmi_before
##   6   25  bmi  hb_a1c_diag_numeric  time_between_bmi_before
##   6   26  bmi  hb_a1c_diag_numeric  time_between_bmi_before
##   6   27  bmi  hb_a1c_diag_numeric  time_between_bmi_before
##   6   28  bmi  hb_a1c_diag_numeric  time_between_bmi_before
##   6   29  bmi  hb_a1c_diag_numeric  time_between_bmi_before
##   6   30  bmi  hb_a1c_diag_numeric  time_between_bmi_before
##   6   31  bmi  hb_a1c_diag_numeric  time_between_bmi_before
##   6   32  bmi  hb_a1c_diag_numeric  time_between_bmi_before
##   6   33  bmi  hb_a1c_diag_numeric  time_between_bmi_before
##   6   34  bmi  hb_a1c_diag_numeric  time_between_bmi_before
##   6   35  bmi  hb_a1c_diag_numeric  time_between_bmi_before
##   6   36  bmi  hb_a1c_diag_numeric  time_between_bmi_before
##   6   37  bmi  hb_a1c_diag_numeric  time_between_bmi_before
##   6   38  bmi  hb_a1c_diag_numeric  time_between_bmi_before
##   6   39  bmi  hb_a1c_diag_numeric  time_between_bmi_before
##   6   40  bmi  hb_a1c_diag_numeric  time_between_bmi_before
##   6   41  bmi  hb_a1c_diag_numeric  time_between_bmi_before
##   6   42  bmi  hb_a1c_diag_numeric  time_between_bmi_before
##   6   43  bmi  hb_a1c_diag_numeric  time_between_bmi_before
##   6   44  bmi  hb_a1c_diag_numeric  time_between_bmi_before
##   6   45  bmi  hb_a1c_diag_numeric  time_between_bmi_before
##   6   46  bmi  hb_a1c_diag_numeric  time_between_bmi_before
##   6   47  bmi  hb_a1c_diag_numeric  time_between_bmi_before
##   6   48  bmi  hb_a1c_diag_numeric  time_between_bmi_before
##   6   49  bmi  hb_a1c_diag_numeric  time_between_bmi_before
##   6   50  bmi  hb_a1c_diag_numeric  time_between_bmi_before
##   6   51  bmi  hb_a1c_diag_numeric  time_between_bmi_before
##   6   52  bmi  hb_a1c_diag_numeric  time_between_bmi_before
##   6   53  bmi  hb_a1c_diag_numeric  time_between_bmi_before
##   6   54  bmi  hb_a1c_diag_numeric  time_between_bmi_before
##   6   55  bmi  hb_a1c_diag_numeric  time_between_bmi_before
##   6   56  bmi  hb_a1c_diag_numeric  time_between_bmi_before
##   6   57  bmi  hb_a1c_diag_numeric  time_between_bmi_before
##   6   58  bmi  hb_a1c_diag_numeric  time_between_bmi_before
##   6   59  bmi  hb_a1c_diag_numeric  time_between_bmi_before
##   6   60  bmi  hb_a1c_diag_numeric  time_between_bmi_before
##   6   61  bmi  hb_a1c_diag_numeric  time_between_bmi_before
##   6   62  bmi  hb_a1c_diag_numeric  time_between_bmi_before
##   6   63  bmi  hb_a1c_diag_numeric  time_between_bmi_before
##   6   64  bmi  hb_a1c_diag_numeric  time_between_bmi_before
##   6   65  bmi  hb_a1c_diag_numeric  time_between_bmi_before
##   7   1  bmi  hb_a1c_diag_numeric  time_between_bmi_before
##   7   2  bmi  hb_a1c_diag_numeric  time_between_bmi_before
##   7   3  bmi  hb_a1c_diag_numeric  time_between_bmi_before
##   7   4  bmi  hb_a1c_diag_numeric  time_between_bmi_before
##   7   5  bmi  hb_a1c_diag_numeric  time_between_bmi_before
##   7   6  bmi  hb_a1c_diag_numeric  time_between_bmi_before
##   7   7  bmi  hb_a1c_diag_numeric  time_between_bmi_before
##   7   8  bmi  hb_a1c_diag_numeric  time_between_bmi_before
##   7   9  bmi  hb_a1c_diag_numeric  time_between_bmi_before
##   7   10  bmi  hb_a1c_diag_numeric  time_between_bmi_before
##   7   11  bmi  hb_a1c_diag_numeric  time_between_bmi_before
##   7   12  bmi  hb_a1c_diag_numeric  time_between_bmi_before
##   7   13  bmi  hb_a1c_diag_numeric  time_between_bmi_before
##   7   14  bmi  hb_a1c_diag_numeric  time_between_bmi_before
##   7   15  bmi  hb_a1c_diag_numeric  time_between_bmi_before
##   7   16  bmi  hb_a1c_diag_numeric  time_between_bmi_before
##   7   17  bmi  hb_a1c_diag_numeric  time_between_bmi_before
##   7   18  bmi  hb_a1c_diag_numeric  time_between_bmi_before
##   7   19  bmi  hb_a1c_diag_numeric  time_between_bmi_before
##   7   20  bmi  hb_a1c_diag_numeric  time_between_bmi_before
##   7   21  bmi  hb_a1c_diag_numeric  time_between_bmi_before
##   7   22  bmi  hb_a1c_diag_numeric  time_between_bmi_before
##   7   23  bmi  hb_a1c_diag_numeric  time_between_bmi_before
##   7   24  bmi  hb_a1c_diag_numeric  time_between_bmi_before
##   7   25  bmi  hb_a1c_diag_numeric  time_between_bmi_before
##   7   26  bmi  hb_a1c_diag_numeric  time_between_bmi_before
##   7   27  bmi  hb_a1c_diag_numeric  time_between_bmi_before
##   7   28  bmi  hb_a1c_diag_numeric  time_between_bmi_before
##   7   29  bmi  hb_a1c_diag_numeric  time_between_bmi_before
##   7   30  bmi  hb_a1c_diag_numeric  time_between_bmi_before
##   7   31  bmi  hb_a1c_diag_numeric  time_between_bmi_before
##   7   32  bmi  hb_a1c_diag_numeric  time_between_bmi_before
##   7   33  bmi  hb_a1c_diag_numeric  time_between_bmi_before
##   7   34  bmi  hb_a1c_diag_numeric  time_between_bmi_before
##   7   35  bmi  hb_a1c_diag_numeric  time_between_bmi_before
##   7   36  bmi  hb_a1c_diag_numeric  time_between_bmi_before
##   7   37  bmi  hb_a1c_diag_numeric  time_between_bmi_before
##   7   38  bmi  hb_a1c_diag_numeric  time_between_bmi_before
##   7   39  bmi  hb_a1c_diag_numeric  time_between_bmi_before
##   7   40  bmi  hb_a1c_diag_numeric  time_between_bmi_before
##   7   41  bmi  hb_a1c_diag_numeric  time_between_bmi_before
##   7   42  bmi  hb_a1c_diag_numeric  time_between_bmi_before
##   7   43  bmi  hb_a1c_diag_numeric  time_between_bmi_before
##   7   44  bmi  hb_a1c_diag_numeric  time_between_bmi_before
##   7   45  bmi  hb_a1c_diag_numeric  time_between_bmi_before
##   7   46  bmi  hb_a1c_diag_numeric  time_between_bmi_before
##   7   47  bmi  hb_a1c_diag_numeric  time_between_bmi_before
##   7   48  bmi  hb_a1c_diag_numeric  time_between_bmi_before
##   7   49  bmi  hb_a1c_diag_numeric  time_between_bmi_before
##   7   50  bmi  hb_a1c_diag_numeric  time_between_bmi_before
##   7   51  bmi  hb_a1c_diag_numeric  time_between_bmi_before
##   7   52  bmi  hb_a1c_diag_numeric  time_between_bmi_before
##   7   53  bmi  hb_a1c_diag_numeric  time_between_bmi_before
##   7   54  bmi  hb_a1c_diag_numeric  time_between_bmi_before
##   7   55  bmi  hb_a1c_diag_numeric  time_between_bmi_before
##   7   56  bmi  hb_a1c_diag_numeric  time_between_bmi_before
##   7   57  bmi  hb_a1c_diag_numeric  time_between_bmi_before
##   7   58  bmi  hb_a1c_diag_numeric  time_between_bmi_before
##   7   59  bmi  hb_a1c_diag_numeric  time_between_bmi_before
##   7   60  bmi  hb_a1c_diag_numeric  time_between_bmi_before
##   7   61  bmi  hb_a1c_diag_numeric  time_between_bmi_before
##   7   62  bmi  hb_a1c_diag_numeric  time_between_bmi_before
##   7   63  bmi  hb_a1c_diag_numeric  time_between_bmi_before
##   7   64  bmi  hb_a1c_diag_numeric  time_between_bmi_before
##   7   65  bmi  hb_a1c_diag_numeric  time_between_bmi_before
##   8   1  bmi  hb_a1c_diag_numeric  time_between_bmi_before
##   8   2  bmi  hb_a1c_diag_numeric  time_between_bmi_before
##   8   3  bmi  hb_a1c_diag_numeric  time_between_bmi_before
##   8   4  bmi  hb_a1c_diag_numeric  time_between_bmi_before
##   8   5  bmi  hb_a1c_diag_numeric  time_between_bmi_before
##   8   6  bmi  hb_a1c_diag_numeric  time_between_bmi_before
##   8   7  bmi  hb_a1c_diag_numeric  time_between_bmi_before
##   8   8  bmi  hb_a1c_diag_numeric  time_between_bmi_before
##   8   9  bmi  hb_a1c_diag_numeric  time_between_bmi_before
##   8   10  bmi  hb_a1c_diag_numeric  time_between_bmi_before
##   8   11  bmi  hb_a1c_diag_numeric  time_between_bmi_before
##   8   12  bmi  hb_a1c_diag_numeric  time_between_bmi_before
##   8   13  bmi  hb_a1c_diag_numeric  time_between_bmi_before
##   8   14  bmi  hb_a1c_diag_numeric  time_between_bmi_before
##   8   15  bmi  hb_a1c_diag_numeric  time_between_bmi_before
##   8   16  bmi  hb_a1c_diag_numeric  time_between_bmi_before
##   8   17  bmi  hb_a1c_diag_numeric  time_between_bmi_before
##   8   18  bmi  hb_a1c_diag_numeric  time_between_bmi_before
##   8   19  bmi  hb_a1c_diag_numeric  time_between_bmi_before
##   8   20  bmi  hb_a1c_diag_numeric  time_between_bmi_before
##   8   21  bmi  hb_a1c_diag_numeric  time_between_bmi_before
##   8   22  bmi  hb_a1c_diag_numeric  time_between_bmi_before
##   8   23  bmi  hb_a1c_diag_numeric  time_between_bmi_before
##   8   24  bmi  hb_a1c_diag_numeric  time_between_bmi_before
##   8   25  bmi  hb_a1c_diag_numeric  time_between_bmi_before
##   8   26  bmi  hb_a1c_diag_numeric  time_between_bmi_before
##   8   27  bmi  hb_a1c_diag_numeric  time_between_bmi_before
##   8   28  bmi  hb_a1c_diag_numeric  time_between_bmi_before
##   8   29  bmi  hb_a1c_diag_numeric  time_between_bmi_before
##   8   30  bmi  hb_a1c_diag_numeric  time_between_bmi_before
##   8   31  bmi  hb_a1c_diag_numeric  time_between_bmi_before
##   8   32  bmi  hb_a1c_diag_numeric  time_between_bmi_before
##   8   33  bmi  hb_a1c_diag_numeric  time_between_bmi_before
##   8   34  bmi  hb_a1c_diag_numeric  time_between_bmi_before
##   8   35  bmi  hb_a1c_diag_numeric  time_between_bmi_before
##   8   36  bmi  hb_a1c_diag_numeric  time_between_bmi_before
##   8   37  bmi  hb_a1c_diag_numeric  time_between_bmi_before
##   8   38  bmi  hb_a1c_diag_numeric  time_between_bmi_before
##   8   39  bmi  hb_a1c_diag_numeric  time_between_bmi_before
##   8   40  bmi  hb_a1c_diag_numeric  time_between_bmi_before
##   8   41  bmi  hb_a1c_diag_numeric  time_between_bmi_before
##   8   42  bmi  hb_a1c_diag_numeric  time_between_bmi_before
##   8   43  bmi  hb_a1c_diag_numeric  time_between_bmi_before
##   8   44  bmi  hb_a1c_diag_numeric  time_between_bmi_before
##   8   45  bmi  hb_a1c_diag_numeric  time_between_bmi_before
##   8   46  bmi  hb_a1c_diag_numeric  time_between_bmi_before
##   8   47  bmi  hb_a1c_diag_numeric  time_between_bmi_before
##   8   48  bmi  hb_a1c_diag_numeric  time_between_bmi_before
##   8   49  bmi  hb_a1c_diag_numeric  time_between_bmi_before
##   8   50  bmi  hb_a1c_diag_numeric  time_between_bmi_before
##   8   51  bmi  hb_a1c_diag_numeric  time_between_bmi_before
##   8   52  bmi  hb_a1c_diag_numeric  time_between_bmi_before
##   8   53  bmi  hb_a1c_diag_numeric  time_between_bmi_before
##   8   54  bmi  hb_a1c_diag_numeric  time_between_bmi_before
##   8   55  bmi  hb_a1c_diag_numeric  time_between_bmi_before
##   8   56  bmi  hb_a1c_diag_numeric  time_between_bmi_before
##   8   57  bmi  hb_a1c_diag_numeric  time_between_bmi_before
##   8   58  bmi  hb_a1c_diag_numeric  time_between_bmi_before
##   8   59  bmi  hb_a1c_diag_numeric  time_between_bmi_before
##   8   60  bmi  hb_a1c_diag_numeric  time_between_bmi_before
##   8   61  bmi  hb_a1c_diag_numeric  time_between_bmi_before
##   8   62  bmi  hb_a1c_diag_numeric  time_between_bmi_before
##   8   63  bmi  hb_a1c_diag_numeric  time_between_bmi_before
##   8   64  bmi  hb_a1c_diag_numeric  time_between_bmi_before
##   8   65  bmi  hb_a1c_diag_numeric  time_between_bmi_before
##   9   1  bmi  hb_a1c_diag_numeric  time_between_bmi_before
##   9   2  bmi  hb_a1c_diag_numeric  time_between_bmi_before
##   9   3  bmi  hb_a1c_diag_numeric  time_between_bmi_before
##   9   4  bmi  hb_a1c_diag_numeric  time_between_bmi_before
##   9   5  bmi  hb_a1c_diag_numeric  time_between_bmi_before
##   9   6  bmi  hb_a1c_diag_numeric  time_between_bmi_before
##   9   7  bmi  hb_a1c_diag_numeric  time_between_bmi_before
##   9   8  bmi  hb_a1c_diag_numeric  time_between_bmi_before
##   9   9  bmi  hb_a1c_diag_numeric  time_between_bmi_before
##   9   10  bmi  hb_a1c_diag_numeric  time_between_bmi_before
##   9   11  bmi  hb_a1c_diag_numeric  time_between_bmi_before
##   9   12  bmi  hb_a1c_diag_numeric  time_between_bmi_before
##   9   13  bmi  hb_a1c_diag_numeric  time_between_bmi_before
##   9   14  bmi  hb_a1c_diag_numeric  time_between_bmi_before
##   9   15  bmi  hb_a1c_diag_numeric  time_between_bmi_before
##   9   16  bmi  hb_a1c_diag_numeric  time_between_bmi_before
##   9   17  bmi  hb_a1c_diag_numeric  time_between_bmi_before
##   9   18  bmi  hb_a1c_diag_numeric  time_between_bmi_before
##   9   19  bmi  hb_a1c_diag_numeric  time_between_bmi_before
##   9   20  bmi  hb_a1c_diag_numeric  time_between_bmi_before
##   9   21  bmi  hb_a1c_diag_numeric  time_between_bmi_before
##   9   22  bmi  hb_a1c_diag_numeric  time_between_bmi_before
##   9   23  bmi  hb_a1c_diag_numeric  time_between_bmi_before
##   9   24  bmi  hb_a1c_diag_numeric  time_between_bmi_before
##   9   25  bmi  hb_a1c_diag_numeric  time_between_bmi_before
##   9   26  bmi  hb_a1c_diag_numeric  time_between_bmi_before
##   9   27  bmi  hb_a1c_diag_numeric  time_between_bmi_before
##   9   28  bmi  hb_a1c_diag_numeric  time_between_bmi_before
##   9   29  bmi  hb_a1c_diag_numeric  time_between_bmi_before
##   9   30  bmi  hb_a1c_diag_numeric  time_between_bmi_before
##   9   31  bmi  hb_a1c_diag_numeric  time_between_bmi_before
##   9   32  bmi  hb_a1c_diag_numeric  time_between_bmi_before
##   9   33  bmi  hb_a1c_diag_numeric  time_between_bmi_before
##   9   34  bmi  hb_a1c_diag_numeric  time_between_bmi_before
##   9   35  bmi  hb_a1c_diag_numeric  time_between_bmi_before
##   9   36  bmi  hb_a1c_diag_numeric  time_between_bmi_before
##   9   37  bmi  hb_a1c_diag_numeric  time_between_bmi_before
##   9   38  bmi  hb_a1c_diag_numeric  time_between_bmi_before
##   9   39  bmi  hb_a1c_diag_numeric  time_between_bmi_before
##   9   40  bmi  hb_a1c_diag_numeric  time_between_bmi_before
##   9   41  bmi  hb_a1c_diag_numeric  time_between_bmi_before
##   9   42  bmi  hb_a1c_diag_numeric  time_between_bmi_before
##   9   43  bmi  hb_a1c_diag_numeric  time_between_bmi_before
##   9   44  bmi  hb_a1c_diag_numeric  time_between_bmi_before
##   9   45  bmi  hb_a1c_diag_numeric  time_between_bmi_before
##   9   46  bmi  hb_a1c_diag_numeric  time_between_bmi_before
##   9   47  bmi  hb_a1c_diag_numeric  time_between_bmi_before
##   9   48  bmi  hb_a1c_diag_numeric  time_between_bmi_before
##   9   49  bmi  hb_a1c_diag_numeric  time_between_bmi_before
##   9   50  bmi  hb_a1c_diag_numeric  time_between_bmi_before
##   9   51  bmi  hb_a1c_diag_numeric  time_between_bmi_before
##   9   52  bmi  hb_a1c_diag_numeric  time_between_bmi_before
##   9   53  bmi  hb_a1c_diag_numeric  time_between_bmi_before
##   9   54  bmi  hb_a1c_diag_numeric  time_between_bmi_before
##   9   55  bmi  hb_a1c_diag_numeric  time_between_bmi_before
##   9   56  bmi  hb_a1c_diag_numeric  time_between_bmi_before
##   9   57  bmi  hb_a1c_diag_numeric  time_between_bmi_before
##   9   58  bmi  hb_a1c_diag_numeric  time_between_bmi_before
##   9   59  bmi  hb_a1c_diag_numeric  time_between_bmi_before
##   9   60  bmi  hb_a1c_diag_numeric  time_between_bmi_before
##   9   61  bmi  hb_a1c_diag_numeric  time_between_bmi_before
##   9   62  bmi  hb_a1c_diag_numeric  time_between_bmi_before
##   9   63  bmi  hb_a1c_diag_numeric  time_between_bmi_before
##   9   64  bmi  hb_a1c_diag_numeric  time_between_bmi_before
##   9   65  bmi  hb_a1c_diag_numeric  time_between_bmi_before
##   10   1  bmi  hb_a1c_diag_numeric  time_between_bmi_before
##   10   2  bmi  hb_a1c_diag_numeric  time_between_bmi_before
##   10   3  bmi  hb_a1c_diag_numeric  time_between_bmi_before
##   10   4  bmi  hb_a1c_diag_numeric  time_between_bmi_before
##   10   5  bmi  hb_a1c_diag_numeric  time_between_bmi_before
##   10   6  bmi  hb_a1c_diag_numeric  time_between_bmi_before
##   10   7  bmi  hb_a1c_diag_numeric  time_between_bmi_before
##   10   8  bmi  hb_a1c_diag_numeric  time_between_bmi_before
##   10   9  bmi  hb_a1c_diag_numeric  time_between_bmi_before
##   10   10  bmi  hb_a1c_diag_numeric  time_between_bmi_before
##   10   11  bmi  hb_a1c_diag_numeric  time_between_bmi_before
##   10   12  bmi  hb_a1c_diag_numeric  time_between_bmi_before
##   10   13  bmi  hb_a1c_diag_numeric  time_between_bmi_before
##   10   14  bmi  hb_a1c_diag_numeric  time_between_bmi_before
##   10   15  bmi  hb_a1c_diag_numeric  time_between_bmi_before
##   10   16  bmi  hb_a1c_diag_numeric  time_between_bmi_before
##   10   17  bmi  hb_a1c_diag_numeric  time_between_bmi_before
##   10   18  bmi  hb_a1c_diag_numeric  time_between_bmi_before
##   10   19  bmi  hb_a1c_diag_numeric  time_between_bmi_before
##   10   20  bmi  hb_a1c_diag_numeric  time_between_bmi_before
##   10   21  bmi  hb_a1c_diag_numeric  time_between_bmi_before
##   10   22  bmi  hb_a1c_diag_numeric  time_between_bmi_before
##   10   23  bmi  hb_a1c_diag_numeric  time_between_bmi_before
##   10   24  bmi  hb_a1c_diag_numeric  time_between_bmi_before
##   10   25  bmi  hb_a1c_diag_numeric  time_between_bmi_before
##   10   26  bmi  hb_a1c_diag_numeric  time_between_bmi_before
##   10   27  bmi  hb_a1c_diag_numeric  time_between_bmi_before
##   10   28  bmi  hb_a1c_diag_numeric  time_between_bmi_before
##   10   29  bmi  hb_a1c_diag_numeric  time_between_bmi_before
##   10   30  bmi  hb_a1c_diag_numeric  time_between_bmi_before
##   10   31  bmi  hb_a1c_diag_numeric  time_between_bmi_before
##   10   32  bmi  hb_a1c_diag_numeric  time_between_bmi_before
##   10   33  bmi  hb_a1c_diag_numeric  time_between_bmi_before
##   10   34  bmi  hb_a1c_diag_numeric  time_between_bmi_before
##   10   35  bmi  hb_a1c_diag_numeric  time_between_bmi_before
##   10   36  bmi  hb_a1c_diag_numeric  time_between_bmi_before
##   10   37  bmi  hb_a1c_diag_numeric  time_between_bmi_before
##   10   38  bmi  hb_a1c_diag_numeric  time_between_bmi_before
##   10   39  bmi  hb_a1c_diag_numeric  time_between_bmi_before
##   10   40  bmi  hb_a1c_diag_numeric  time_between_bmi_before
##   10   41  bmi  hb_a1c_diag_numeric  time_between_bmi_before
##   10   42  bmi  hb_a1c_diag_numeric  time_between_bmi_before
##   10   43  bmi  hb_a1c_diag_numeric  time_between_bmi_before
##   10   44  bmi  hb_a1c_diag_numeric  time_between_bmi_before
##   10   45  bmi  hb_a1c_diag_numeric  time_between_bmi_before
##   10   46  bmi  hb_a1c_diag_numeric  time_between_bmi_before
##   10   47  bmi  hb_a1c_diag_numeric  time_between_bmi_before
##   10   48  bmi  hb_a1c_diag_numeric  time_between_bmi_before
##   10   49  bmi  hb_a1c_diag_numeric  time_between_bmi_before
##   10   50  bmi  hb_a1c_diag_numeric  time_between_bmi_before
##   10   51  bmi  hb_a1c_diag_numeric  time_between_bmi_before
##   10   52  bmi  hb_a1c_diag_numeric  time_between_bmi_before
##   10   53  bmi  hb_a1c_diag_numeric  time_between_bmi_before
##   10   54  bmi  hb_a1c_diag_numeric  time_between_bmi_before
##   10   55  bmi  hb_a1c_diag_numeric  time_between_bmi_before
##   10   56  bmi  hb_a1c_diag_numeric  time_between_bmi_before
##   10   57  bmi  hb_a1c_diag_numeric  time_between_bmi_before
##   10   58  bmi  hb_a1c_diag_numeric  time_between_bmi_before
##   10   59  bmi  hb_a1c_diag_numeric  time_between_bmi_before
##   10   60  bmi  hb_a1c_diag_numeric  time_between_bmi_before
##   10   61  bmi  hb_a1c_diag_numeric  time_between_bmi_before
##   10   62  bmi  hb_a1c_diag_numeric  time_between_bmi_before
##   10   63  bmi  hb_a1c_diag_numeric  time_between_bmi_before
##   10   64  bmi  hb_a1c_diag_numeric  time_between_bmi_before
##   10   65  bmi  hb_a1c_diag_numeric  time_between_bmi_before
```

### Diagnostics

```
# Trace plots
plot(code_non_image_imputed)
```

```
# Density plots
densityplot(code_non_image_imputed, ~bmi)
```

```
# FMI/Lambda Table
pooled_diag_non_image <- code_non_image_imputed %>%
  with(glm(rct_status ~ bmi + age + sex + race_eth, family = binomial)) %>%
  pool()

mi_diagnostics_non_image <- pooled_diag_non_image$pooled %>%
  dplyr::select(term, riv, lambda, fmi) %>%
  mutate(across(where(is.numeric), ~round(., 4)))

print(mi_diagnostics_non_image)
```

```
##                         term    riv lambda    fmi
## 1                (Intercept) 0.1200 0.1071 0.1075
## 2                        bmi 1.2067 0.5468 0.5511
## 3                        age 0.0517 0.0491 0.0492
## 4                    sexMale 0.0082 0.0081 0.0082
## 5                       sexU 0.0000 0.0000 0.0000
## 6 race_ethBlack Non-Hispanic 0.0035 0.0035 0.0035
## 7           race_ethHispanic 0.0026 0.0026 0.0026
## 8              race_ethOther 0.0024 0.0024 0.0025
## 9 race_ethWhite Non-Hispanic 0.0019 0.0019 0.0020
```

interpretation after imputation: - ppm: predictive mean matching, for
continuous variables. - An empty string �� means that the variable was
not imputed. Because: 1) no missing data; 2) skipped by algorithm, often
due to perfect collinearity.

Trace plot: - No Obvious Trends: For all three imputed variables
(bmi, hb\_a1c\_diag\_numeric, and time\_between\_bmi\_before), the lines move
horizontally across the plot without any clear upward or downward
pattern. - Good Mixing: The lines are tangled together in what is often
called a �fuzzy caterpillar,� which shows that the imputation process is
stable and the different imputed datasets are similar to each other.

Density Plot: - Similar to the NLP dataset, we see the red lines
(imputed data) are slightly shifted to the left and more �peaked�
compared to the blue line (observed data). - The model is detecting that
the patients in the �Code Non-Image� cohort who are missing their BMI
likely belong to a demographic with historically lower BMIs. The
imputation is correctly capturing this underlying clinical reality
rather than just pulling the average.

FMI: - The covariates in the �Code Non-Image� cohort did not provide
strong predictive power to �recover� the missing BMI. The missingness
added almost as much uncertainty as if the data were just deleted.

## create bmi\_cat

```
# Create `bmi_cat` within Imputed Datasets

# Convert the imputed object to a long-format data frame
long_df <- mice::complete(code_non_image_imputed, action = "long", include = TRUE)

# Add the original `rct_status` back
long_df$rct_status <- rep(code_non_image_df$rct_status, code_non_image_imputed$m + 1)

# Create `bmi_cat` and set the reference level
long_df_wrangled <- long_df %>%
  mutate(
    bmi_cat = case_when(
      bmi < 18.5          ~ "Underweight (<18.5)",
      bmi >= 18.5 & bmi < 25 ~ "Normal (18.5-24.9)",
      bmi >= 25   & bmi < 30 ~ "Overweight (25-29.9)",
      bmi >= 30           ~ "Obese (>=30)"
    ),
    # Convert to factor and set the reference group
    bmi_cat = fct_relevel(bmi_cat, "Normal (18.5-24.9)")
  )

# Convert the modified long data frame back into a `Multiply imputed data set (mids)` object for analysis
code_non_image_final_mids <- as.mids(long_df_wrangled)
```

## pooled analysis

ref: Control (Code Non Image)

```
# run each model on the imputed data and pool the results.
# `tbl_regression` automatically understands pooled results and formats them.
# The `variable` argument is used to select the predictors we are interested in.

run_and_format_non_image <- function(mids_obj, formula, include_vars) {
  with(mids_obj, glm(as.formula(formula), family = binomial)) %>%
    tbl_regression(exponentiate = TRUE) %>%
    modify_table_body(~.x %>% filter(variable %in% include_vars)) %>%
    add_n(location = "level")
}

cat("### Model 1: RCT Status ~ Any Diabetes (Adjusted)")
```

```
## ### Model 1: RCT Status ~ Any Diabetes (Adjusted)
```

```
run_and_format_non_image(code_non_image_final_mids, "rct_status ~ dm_any + age + sex + race_eth + bmi", c("dm_any", "bmi"))
```

| **Characteristic** | **N** | **OR** | **95% CI** | **p-value** |
| --- | --- | --- | --- | --- |
| dm\_any |  |  |  |  |
| ����None | 38,667 | � | � |  |
| ����Any | 3,743 | 1.68 | 1.56, 1.82 | <0.001 |
| bmi | 42,410 | 1.02 | 1.02, 1.03 | <0.001 |
|  |  |  |  |  |
| --- | --- | --- | --- | --- |
| Abbreviations: CI = Confidence Interval, OR = Odds Ratio | | | | |

```
cat("\n### Model 2: RCT Status ~ T1 Diabetes (Adjusted)")
```

```
## 
## ### Model 2: RCT Status ~ T1 Diabetes (Adjusted)
```

```
run_and_format_non_image(code_non_image_final_mids, "rct_status ~ t1d + age + sex + race_eth + bmi", c("t1d", "bmi"))
```

| **Characteristic** | **N** | **OR** | **95% CI** | **p-value** |
| --- | --- | --- | --- | --- |
| t1d |  |  |  |  |
| ����No | 38,667 | � | � |  |
| ����Yes | 625 | 2.03 | 1.72, 2.41 | <0.001 |
| bmi | 39,292 | 1.02 | 1.02, 1.03 | <0.001 |
|  |  |  |  |  |
| --- | --- | --- | --- | --- |
| Abbreviations: CI = Confidence Interval, OR = Odds Ratio | | | | |

```
cat("\n### Model 3: RCT Status ~ T2 Diabetes (Adjusted)")
```

```
## 
## ### Model 3: RCT Status ~ T2 Diabetes (Adjusted)
```

```
run_and_format_non_image(code_non_image_final_mids, "rct_status ~ t2d + age + sex + race_eth + bmi", c("t2d", "bmi"))
```

| **Characteristic** | **N** | **OR** | **95% CI** | **p-value** |
| --- | --- | --- | --- | --- |
| t2d |  |  |  |  |
| ����No | 38,667 | � | � |  |
| ����Yes | 1,692 | 1.93 | 1.73, 2.16 | <0.001 |
| bmi | 40,359 | 1.02 | 1.02, 1.03 | <0.001 |
|  |  |  |  |  |
| --- | --- | --- | --- | --- |
| Abbreviations: CI = Confidence Interval, OR = Odds Ratio | | | | |

```
cat("\n### Model 4: RCT Status ~ BMI (Unadjusted)")
```

```
## 
## ### Model 4: RCT Status ~ BMI (Unadjusted)
```

```
run_and_format_non_image(code_non_image_final_mids, "rct_status ~ bmi", "bmi")
```

| **Characteristic** | **N** | **OR** | **95% CI** | **p-value** |
| --- | --- | --- | --- | --- |
| bmi | 42,410 | 1.02 | 1.01, 1.02 | <0.001 |
|  |  |  |  |  |
| --- | --- | --- | --- | --- |
| Abbreviations: CI = Confidence Interval, OR = Odds Ratio | | | | |

```
cat("\n### Model 5: RCT Status ~ BMI (Adjusted)")
```

```
## 
## ### Model 5: RCT Status ~ BMI (Adjusted)
```

```
run_and_format_non_image(code_non_image_final_mids, "rct_status ~ bmi + age + sex + race_eth", "bmi")
```

| **Characteristic** | **N** | **OR** | **95% CI** | **p-value** |
| --- | --- | --- | --- | --- |
| bmi | 42,410 | 1.03 | 1.02, 1.03 | <0.001 |
|  |  |  |  |  |
| --- | --- | --- | --- | --- |
| Abbreviations: CI = Confidence Interval, OR = Odds Ratio | | | | |

```
cat("\n### Model 6: RCT Status ~ BMI Category (Unadjusted)")
```

```
## 
## ### Model 6: RCT Status ~ BMI Category (Unadjusted)
```

```
run_and_format_non_image(code_non_image_final_mids, "rct_status ~ bmi_cat", "bmi_cat")
```

| **Characteristic** | **N** | **OR** | **95% CI** | **p-value** |
| --- | --- | --- | --- | --- |
| bmi\_cat |  |  |  |  |
| ����Normal (18.5-24.9) | 10,728 | � | � |  |
| ����Obese (>=30) | 16,181 | 1.46 | 1.34, 1.59 | <0.001 |
| ����Overweight (25-29.9) | 14,829 | 1.31 | 1.21, 1.42 | <0.001 |
| ����Underweight (<18.5) | 672 | 0.60 | 0.44, 0.82 | 0.001 |
|  |  |  |  |  |
| --- | --- | --- | --- | --- |
| Abbreviations: CI = Confidence Interval, OR = Odds Ratio | | | | |

```
cat("\n### Model 7: RCT Status ~ BMI Category (Adjusted)")
```

```
## 
## ### Model 7: RCT Status ~ BMI Category (Adjusted)
```

```
run_and_format_non_image(code_non_image_final_mids, "rct_status ~ bmi_cat + age + sex + race_eth", "bmi_cat")
```

| **Characteristic** | **N** | **OR** | **95% CI** | **p-value** |
| --- | --- | --- | --- | --- |
| bmi\_cat |  |  |  |  |
| ����Normal (18.5-24.9) | 10,728 | � | � |  |
| ����Obese (>=30) | 16,181 | 1.54 | 1.41, 1.69 | <0.001 |
| ����Overweight (25-29.9) | 14,829 | 1.31 | 1.20, 1.42 | <0.001 |
| ����Underweight (<18.5) | 672 | 0.58 | 0.42, 0.79 | <0.001 |
|  |  |  |  |  |
| --- | --- | --- | --- | --- |
| Abbreviations: CI = Confidence Interval, OR = Odds Ratio | | | | |

```
cat("\n### Model 8: RCT Status ~ BMI Category (Adjusted + T1D)")
```

```
## 
## ### Model 8: RCT Status ~ BMI Category (Adjusted + T1D)
```

```
run_and_format_non_image(code_non_image_final_mids, "rct_status ~ bmi_cat + age + sex + race_eth + t1d", c("t1d", "bmi_cat"))
```

| **Characteristic** | **N** | **OR** | **95% CI** | **p-value** |
| --- | --- | --- | --- | --- |
| bmi\_cat |  |  |  |  |
| ����Normal (18.5-24.9) | 10,326 | � | � |  |
| ����Obese (>=30) | 14,349 | 1.44 | 1.31, 1.59 | <0.001 |
| ����Overweight (25-29.9) | 13,960 | 1.27 | 1.16, 1.38 | <0.001 |
| ����Underweight (<18.5) | 657 | 0.57 | 0.42, 0.79 | <0.001 |
| t1d |  |  |  |  |
| ����No | 38,667 | � | � |  |
| ����Yes | 625 | 2.07 | 1.75, 2.45 | <0.001 |
|  |  |  |  |  |
| --- | --- | --- | --- | --- |
| Abbreviations: CI = Confidence Interval, OR = Odds Ratio | | | | |
