# Supplementary material for: Leveraging electronic health record data to evidence collider stratification bias and inform clinical epidemiology: obesity, diabetes, and rotator cuff tears
Source: Am J Epidemiol. 2026 Jun 1;195(10):2592–601. doi: 10.1093/aje/kwag117 (PMC13642364; doi:10.1093/aje/kwag117)
Supplement: Supplementary file 1 [file web_material_kwag117.zip › Supplemental materials final .docx]

**Leveraging Electronic Health Record Data to Evidence Collider Stratification Bias and Inform Clinical Epidemiology: Obesity, Diabetes and Rotator Cuff Tears.**

Ueland, Simone D., MD, PhD^1,2,3^; Basnet, Til, PhD^2^; Liu, Wenting, MPH^4^; Ong, Henry H., PhD^5^; Gangreddi, Srushti, MS^5^; Wei, Wei-Qi, MD, PhD^5^; Wen, Wanqing, MD, MPH^2^; Hartmann, Katherine E., MD, PhD^6^; Jain, Nitin B., MD, MSPH^7^; Giri, Ayush, PhD^2,8^.

^1^ Vanderbilt University School of Medicine, Nashville, TN, USA

^2^ Division of Epidemiology, Department of Medicine, Vanderbilt Health, Nashville, TN, USA

^3^ Boston Medical Center, Department of Orthopedic Surgery, Boston, MA, USA.

^4^ Department of Biostatistics and Data Science, University of Texas Health Science Center at Houston, Houston, Texas, USA

^5^ Department of Biomedical Informatics, Vanderbilt Health, Nashville, TN, USA

^6^ University of Kentucky Center for Clinical and Translational Science, Lexington, KY, USA

^7^ Department of Physical Medicine and Rehabilitation, University of Michigan, Ann Arbor, MI, USA

^8^ Division of Quantitative and Clinical Sciences, Department of Obstetrics and Gynecology, Vanderbilt Health, Nashville, Tennessee, USA

**List of Included Supplemental Material:**

**Table S1:** Full List of ICD and CPT Codes

**Table S2:** Associations between diabetes exposures and rotator cuff tears across case-control study designs (with and without imaging requirement), with and without adjustment for BMI in full-sample and complete-case analysis scenarios, and with multiple imputation.

**Table S3:** Demographics and characteristics of rotator cuff tear cases and controls across sets with and without imaging restricted to individuals for whom BMI is not missing across the entire VUMC Synthetic Derivative.

**Table S4:** Demographics and characteristics of rotator cuff tear cases and controls across sets with and without imaging restricted to individuals for whom genetic data is available.

**Table S5:** Associations between diabetes exposures and rotator cuff tears across case-control study designs (with and without imaging requirement) with adjustment for BMI in complete-case analysis scenarios in individuals for whom genetic data is available.

**File S1.** Imputation and Pooled Analyses Code and Diagnostics (included in attached HTML file).

**Corresponding Author:**

Ayush Giri, PhD

Division of Epidemiology

Vanderbilt University

2525 West End Ave,

Nashville, TN, 37203

Email 1: [ayush.giri@vumc.org](mailto:ayush.giri@vumc.org)

Email 2: [girilabepidemiology@gmail.com](mailto:girilabepidemiology@gmail.com)

Supplemental Material:

| Table S1. Full List of ICD and CPT Codes | | | |
| --- | --- | --- | --- |
| Variable Name | Code Type | Code | Code Explanation |
| Codes for Rotator Cuff Tear | | | |
| rct_cpt_surg_spec_include | CPT | 23412 | repair of ruptured musculotendinous cuff (eg. Rotator cuff) open; chronic |
| rct_cpt_surg_spec_include | CPT | 23420 | reconstruction of complete shoulder (rotator) cuff avulsion; chronic |
| rct_cpt_surg_nonspec_include | CPT | 23397 | Under Repair, Revision, and/or Reconstruction Procedures on the Shoulder |
| rct_cpt_surg_nonspec_include | CPT | 29826 | Arthroscopy, shoulder, surgical; decompression of subacromial space with partial |
| rct_cpt_surg_nonspec_include | CPT | 29901 | Under Endoscopy/Arthroscopy Procedures on the Musculoskeletal System |
| rct_cpt_surg_nonspec_include | CPT | 29827 | Arthroscopy shoulder, surgical; with rotator cuff repair |
| rct_cpt_surg_nonspec_include | CPT | 29828 | Arthroscopy shoulder, surgical; with rotator cuff repair |
| rct_cpt_surg_nonspec_include | CPT | 29822 | Arthroscopy, shoulder, surgical; debridement, limited includes debridement of soft or hard tissue |
| RCT_icd9_diag_include | ICD9CM | 727.61 | Complete rupture of rotator cuff |
| RCT_icd9_diag_include | ICD9CM | 726.13 | Partial tear of rotator cuff |
| RCT_icd9_diag_include | ICD9CM | 83.63 | Rotator cuff repair |
| RCT_icd10_diag_include | ICD10CM | M75.120 | Complete rotator cuff tear or rupture of unspecified shoulder, not specified as traumatic |
| RCT_icd10_diag_include | ICD10CM | M75.121 | Complete rotator cuff tear or rupture of right shoulder, not specified as traumatic |
| RCT_icd10_diag_include | ICD10CM | M75.122 | Complete rotator cuff tear or rupture of left shoulder, not specified as traumatic |
| RCT_icd10_diag_include | ICD10CM | M75.110 | Incomplete rotator cuff tear or rupture of unpsecified shoulder, not specified as traumatic |
| RCT_icd10_diag_include | ICD10CM | M75.111 | Incomplete rotator cuff tear or rupture of right shoulder, not specified as traumatic |
| RCT_icd10_diag_include | ICD10CM | M75.112 | Incomplete rotator cuff tear or rupture of left shoulder, not specified as traumatic |
| RCT_icd10_diag_include | ICD10CM | M75.100 | Unspecified rotator cuff tear or rupture of unspecified shoulder, not specified as traumatic |
| RCT_icd10_diag_include | ICD10CM | M75.101 | Unspecified rotator cuff tear or rupture of right shoulder, not specified as traumatic |
| RCT_icd10_diag_include | ICD10CM | M75.102 | Unspecified rotator cuff tear or rupture of left shoulder, not specified as traumatic |
| Codes for Rotator Cuff Exclusions (Traumatic Tear) | | | |
| rct_cpt_surg_exclude | CPT | 23410 | repair of ruptured musculotendinous cuff (eg. Rotator cuff) open; acute |
| rct_cpt_surg_exclude | CPT | 24341 | repair, tendon or muscle, upper arm or elbow, each tendon or muscle, primary or secondary (excludes Rotator cuff) |
| RCT_icd9_exclusions | ICD9CM | 840.3 | Infraspinatus (muscle) (tendon) sprain |
| RCT_icd9_exclusions | ICD9CM | 840.4 | Rotator cuff (capsule) sprain |
| RCT_icd9_exclusions | ICD9CM | 840.5 | Subscapularis (muscle) sprain |
| RCT_icd9_exclusions | ICD9CM | 840.6 | Supraspinatus (muscle) (tendon) sprain |
| rct_icd10_exclude | ICD10CM | S46.011A | Strain of muscle(s) and tendon(s) of the rotator cuff of right shoulder, initial encounter |
| rct_icd10_exclude | ICD10CM | S46.011D | Strain of muscle(s) and tendon(s) of the rotator cuff of right shoulder, subsequent encounter |
| rct_icd10_exclude | ICD10CM | S46.011S | Strain of muscle(s) and tendon(s) of the rotator cuff of right shoulder, sequela |
| rct_icd10_exclude | ICD10CM | S46.012A | Strain of muscle(s) and tendon(s) of the rotator cuff of left shoulder, initial encounter |
| rct_icd10_exclude | ICD10CM | S46.012D | Strain of muscle(s) and tendon(s) of the rotator cuff of left shoulder, sequential encounter |
| rct_icd10_exclude | ICD10CM | S46.012S | Strain of muscle(s) and tendon(s) of the rotator cuff of left shoulder, sequela |
| rct_icd10_exclude | ICD10CM | S46.011A | Strain of muscle(s) and tendon(s) of the rotator cuff of unspecified shoulder, initial encounter |
| rct_icd10_exclude | ICD10CM | S46.011D | Strain of muscle(s) and tendon(s) of the rotator cuff of unspecified shoulder, subsequent encounter |
| rct_icd10_exclude | ICD10CM | S46.011S | Strain of muscle(s) and tendon(s) of the rotator cuff of unspecified shoulder, sequela |
| rct_icd10_exclude | ICD10CM | S46.021A | Laceration of muscle(s) and tendon(s) of the rotator cuff of the right shoulder, initial encounter |
| rct_icd10_exclude | ICD10CM | S46.021D | Laceration of muscle(s) and tendon(s) of the rotator cuff of the right shoulder, Sequential encounter |
| rct_icd10_exclude | ICD10CM | S46.021S | Laceration of muscle(s) and tendon(s) of the rotator cuff of the right shoulder, sequela |
| rct_icd10_exclude | ICD10CM | S46.022A | Laceration of muscle(s) and tendon(s) of the rotator cuff of the left shoulder, initial encounter |
| rct_icd10_exclude | ICD10CM | S46.022D | Laceration of muscle(s) and tendon(s) of the rotator cuff of the left shoulder, Sequential encounter |
| rct_icd10_exclude | ICD10CM | S46.022S | Laceration of muscle(s) and tendon(s) of the rotator cuff of the left shoulder, sequela |
| rct_icd10_exclude | ICD10CM | S46.029A | Laceration of muscle(s) and tendon(s) of the rotator cuff of unspecified shoulder, initial encounter |
| rct_icd10_exclude | ICD10CM | S46.029D | Laceration of muscle(s) and tendon(s) of the rotator cuff of unspecified shoulder, Sequential encounter |
| rct_icd10_exclude | ICD10CM | S46.029S | Laceration of muscle(s) and tendon(s) of the rotator cuff of unspecified shoulder, sequela |
| rct_icd10_exclude | ICD10CM | S43.421A | Sprain of muscle(s) and tendon(s) of the rotator cuff of right shoulder, initial encounter |
| rct_icd10_exclude | ICD10CM | S43.421D | Sprain of muscle(s) and tendon(s) of the rotator cuff of right shoulder, sequential encounter |
| rct_icd10_exclude | ICD10CM | S43.421S | Sprain of muscle(s) and tendon(s) of the rotator cuff of right shoulder, sequela |
| rct_icd10_exclude | ICD10CM | S43.422A | Sprain of muscle(s) and tendon(s) of the rotator cuff of left shoulder, initial encounter |
| rct_icd10_exclude | ICD10CM | S43.422D | Sprain of muscle(s) and tendon(s) of the rotator cuff of left shoulder, sequential encounter |
| rct_icd10_exclude | ICD10CM | S43.422S | Sprain of muscle(s) and tendon(s) of the rotator cuff of left shoulder, sequela |
| rct_icd10_exclude | ICD10CM | S43.429A | Sprain of muscle(s) and tendon(s) of the rotator cuff of unspecified shoulder, initial encounter |
| rct_icd10_exclude | ICD10CM | S43.429D | Sprain of muscle(s) and tendon(s) of the rotator cuff of unspecified shoulder, sequential encounter |
| rct_icd10_exclude | ICD10CM | S43.429S | Sprain of muscle(s) and tendon(s) of the rotator cuff of unspecified shoulder, sequela |
| rct_icd10_exclude | ICD10CM | M12.511 | Traumatic Arthropathy, right shoulder |
| rct_icd10_exclude | ICD10CM | M12.512 | Traumatic Arthropathy, left shoulder |
| rct_icd10_exclude | ICD10CM | M12.519 | Traumatic Arthropathy, unspecified shoulder |
| rct_icd10_exclude | ICD10CM | M75.30 | Calcific Tendinitis of unpsecified shoulder |
| rct_icd10_exclude | ICD10CM | M75.31 | Calcific Tendinitis of right shoulder |
| rct_icd10_exclude | ICD10CM | M75.32 | Calcific Tendinitis of left shoulder |
| CPT Codes for Shoulder Imaging | | | |
| rct_cpt_image_include | CPT | 76880 | [Expired] Ultrasound, extremity, nonvascular, real time with image documentation |
| rct_cpt_image_include | CPT | 78661 | Ultrasound, complete joint (ie. Joint space and peri-articular soft tissue structures) real time with image documentation |
| rct_cpt_image_include | CPT | 78662 | Ultrasound, limited, joint or other non-vascular extremity structure (i.e. joint space, peri-articular tendon[s], muscle[s], nerve[s], other soft tissue structure[s], or soft tissue mass[es]) real time with image documentation |
| RCT_icd9_image_include | ICD9CM | 88.94 | Magnetic Resonance Imaging of Musculoskeletal |
| RCT_icd9_image_include | ICD9CM | 88.32 | Contrast arthrogram |
| RCT_icd9_image_include | ICD9CM | 88.7 | Diagnostic Ultrasound |
| RCT_icd10_image_include | ICD10CM | BP3EZZZ | MRI upper extremity left |
| RCT_icd10_image_include | ICD10CM | BP3FYZZ | MRI upper extremity left, with contrast |
| RCT_icd10_image_include | ICD10CM | BP3FY0Z | MRI upper extremity left, enhanced or unenhanced |
| RCT_icd10_image_include | ICD10CM | BP38ZZZ | MRI upper extremity right |
| RCT_icd10_image_include | ICD10CM | BP38YZZ | MRI upper extremity right, with contrast |
| RCT_icd10_image_include | ICD10CM | BP38Y0Z | MRI upper extremity right, enhanced or unenhanced |
| RCT_icd10_image_include | ICD10CM | BL33YZZZ | MRI upper extremity tendon |
| RCT_icd10_image_include | ICD10CM | BL33YZZ | MRI upper extremity tendon with contrast |
| RCT_icd10_image_include | ICD10CM | BL33Y0Z | MRI upper extremity tendon with contrast, enhanced or unenhanced |
| RCT_icd10_image_include | ICD10CM | BL30ZZZ | MRI upper extremity Connective tissue |
| RCT_icd10_image_include | ICD10CM | BL30YZZ | MRI upper extremity Connective tissue with contrast |
| RCT_icd10_image_include | ICD10CM | BL30Y0Z | MRI upper extremity Connective Tissue with contrast, enhanced or unenhanced |
| RCT_icd10_image_include | ICD10CM | B53NZZZ | MRI upper extremity vein |
| RCT_icd10_image_include | ICD10CM | B53NYZZ | MRI upper extremity vein with contrast |
| RCT_icd10_image_include | ICD10CM | B53NY0Z | MRI upper extremity vein with contrast enhanced or unenhanced |
| RCT_icd10_image_include | ICD10CM | B33KZZZ | MRI upper extremity artery |
| RCT_icd10_image_include | ICD10CM | B33KYZZ | MRI upper extremity artery with contrast |
| RCT_icd10_image_include | ICD10CM | B33KY0Z | MRI upper extremity artery with contrast enhanced or unenhanced |
| RCT_icd10_image_include | ICD10CM | B33JZZZ | MRI Left upper extremity artery |
| RCT_icd10_image_include | ICD10CM | B33JYZZ | MRI Left upper extremity artery with contrast |
| RCT_icd10_image_include | ICD10CM | B33JY0Z | MRI Left upper extremity artery with contrast enhanced or unenhanced |
| RCT_icd10_image_include | ICD10CM | B33HZZZ | MRI Right upper extremity artery |
| RCT_icd10_image_include | ICD10CM | B33HYZZ | MRI Right upper extremity artery with contrast |
| RCT_icd10_image_include | ICD10CM | B33HY0Z | MRI Right upper extremity artery with contrast enhanced or unenhanced |
| ICD 9 Codes for Non-Traumatic Tendon Injury | | | |
| RCT_icd9_nontraum | ICD9CM | 727.6 | Rupture of tendon nontraumatic |
| RCT_icd9_nontraum | ICD9CM | 727.60 | Nontraumatic rupture of unspecified tendon |
| CPT codes for Physical Therapy | | | |
| rct_cpt_PT_include | CPT | 90071 | [Expired] Physical therapy evaluation |
| rct_cpt_PT_include | CPT | 90072 | [Expired] Physical therapy re-evaluation |
| rct_cpt_PT_include | CPT | 97161 | Physical therapy, low complexity, typical time with patient and/or/family 10 mins |
| rct_cpt_PT_include | CPT | 97162 | Physical therapy, moderate complexity, typical time with patient and/or/family 30 mins |
| rct_cpt_PT_include | CPT | 97163 | Physical therapy, high complexity, typical time with patient and/or/family 45 mins |
| rct_cpt_PT_include | CPT | 97164 | Reevaluation of physical therapy established plan of care, requiring these components: An examination including a review of history and use of standardized tests and measures is required; and revised plan of care using a standardized patient assessment instrument or measurable assessment of functional outcome Typically 20 mins are spent with the patient or family. |
| RCT_icd9_pt_include | ICD9CM | 93.0 | physical therapy, respiratory therapy, rehabilitation, and related procedures |
| RCT_icd9_pt_include | ICD9CM | 93.00 | diagnostic physical therapy |
| RCT_icd9_pt_include | ICD9CM | 93.09 | other diagnostic physical therapy procedure |
| RCT_icd9_pt_include | ICD9CM | 93.1 | physical therapy procedure |
| RCT_icd9_pt_include | ICD9CM | 93.2 | other physical therapy musculoskeletal procedures |
| RCT_icd9_pt_include | ICD9CM | 93.38 | combined physical therapy without mention of components |
| RCT_icd9_pt_include | ICD9CM | 93.39 | other physical therapy |
| RCT_icd9_pt_include | ICD9CM | V57.1 | care involving other physical therapy |
| RCT_icd10_image_include | ICD10CM | BP3EZZZ | MRI upper extremity left |
| RCT_icd10_image_include | ICD10CM | BP3FYZZ | MRI upper extremity left, with contrast |
| RCT_icd10_image_include | ICD10CM | BP3FY0Z | MRI upper extremity left, enhanced or unenhanced |
| RCT_icd10_image_include | ICD10CM | BP38ZZZ | MRI upper extremity right |
| RCT_icd10_image_include | ICD10CM | BP38YZZ | MRI upper extremity right, with contrast |
| RCT_icd10_image_include | ICD10CM | BP38Y0Z | MRI upper extremity right, enhanced or unenhanced |
| RCT_icd10_image_include | ICD10CM | BL33YZZZ | MRI upper extremity tendon |
| RCT_icd10_image_include | ICD10CM | BL33YZZ | MRI upper extremity tendon with contrast |
| RCT_icd10_image_include | ICD10CM | BL33Y0Z | MRI upper extremity tendon with contrast, enhanced or unenhanced |
| RCT_icd10_image_include | ICD10CM | BL30ZZZ | MRI upper extremity Connective tissue |
| RCT_icd10_image_include | ICD10CM | BL30YZZ | MRI upper extremity Connective tissue with contrast |
| RCT_icd10_image_include | ICD10CM | BL30Y0Z | MRI upper extremity Connective Tissue with contrast, enhanced or unenhanced |
| RCT_icd10_image_include | ICD10CM | B53NZZZ | MRI upper extremity vein |
| RCT_icd10_image_include | ICD10CM | B53NYZZ | MRI upper extremity vein with contrast |
| RCT_icd10_image_include | ICD10CM | B53NY0Z | MRI upper extremity vein with contrast enhanced or unenhanced |
| RCT_icd10_image_include | ICD10CM | B33KZZZ | MRI upper extremity artery |
| RCT_icd10_image_include | ICD10CM | B33KYZZ | MRI upper extremity artery with contrast |
| RCT_icd10_image_include | ICD10CM | B33KY0Z | MRI upper extremity artery with contrast enhanced or unenhanced |
| RCT_icd10_image_include | ICD10CM | B33JZZZ | MRI Left upper extremity artery |
| RCT_icd10_image_include | ICD10CM | B33JYZZ | MRI Left upper extremity artery with contrast |
| RCT_icd10_image_include | ICD10CM | B33JY0Z | MRI Left upper extremity artery with contrast enhanced or unenhanced |
| RCT_icd10_image_include | ICD10CM | B33HZZZ | MRI Right upper extremity artery |
| RCT_icd10_image_include | ICD10CM | B33HYZZ | MRI Right upper extremity artery with contrast |
| RCT_icd10_image_include | ICD10CM | B33HY0Z | MRI Right upper extremity artery with contrast enhanced or unenhanced |
| Codes for Diabetes (Type I, Type II and Unspecified) | | | |
| t1_dm | ICD10CM | E10 | Type I diabetes |
| t1_dm | ICD10CM | E10.1 | Type I diabetes mellitus with ketoacidosis |
| t1_dm | ICD10CM | E10.10 | Type I diabetes mellitus with ketoacidosis without coma |
| t1_dm | ICD10CM | E10.11 | Type I diabetes mellitus with ketoacidosis with coma |
| t1_dm | ICD10CM | E10.2 | Type I diabetes mellitus with kidney complications |
| t1_dm | ICD10CM | E10.21 | Type I diabetes mellitus with diabetic nephropathy |
| t1_dm | ICD10CM | E10.22 | Type I diabetes mellitus with diabetic chronic kidney disease |
| t1_dm | ICD10CM | E10.29 | Type I diabetes mellitus with other diabetic kidney complication |
| t1_dm | ICD10CM | E10.3 | Type I diabetes mellitus with ophthalmic complications |
| t1_dm | ICD10CM | E10.31 | Type I diabetes mellitus with unspecified diabetic retinopathy |
| t1_dm | ICD10CM | E10.311 | Type I diabetes mellitus with unspecified diabetic retinopathy with macular edema |
| t1_dm | ICD10CM | E10.319 | Type I diabetes mellitus with unspecified diabetic retinopathy without macular edema |
| t1_dm | ICD10CM | E10.32 | Type I diabetes mellitus with mild nonproliferative diabetic retinopathy |
| t1_dm | ICD10CM | E10.321 | Type I diabetes mellitus with mild nonproliferative diabetic retinopathy with macular edema |
| t1_dm | ICD10CM | E10.3211 | Type I diabetes mellitus with mild nonproliferative diabetic retinopathy with macular edema right eye |
| t1_dm | ICD10CM | E10.3212 | Type I diabetes mellitus with mild nonproliferative diabetic retinopathy with macular edema left eye |
| t1_dm | ICD10CM | E10.3213 | Type I diabetes mellitus with mild nonproliferative diabetic retinopathy with macular edema bilateral |
| t1_dm | ICD10CM | E10.3219 | Type I diabetes mellitus with mild nonproliferative diabetic retinopathy with macular edema unspecified eye |
| t1_dm | ICD10CM | E10.329 | Type I diabetes mellitus with mild nonproliferative diabetic retinopathy without macular edema |
| t1_dm | ICD10CM | E10.3291 | Type I diabetes mellitus with mild nonproliferative diabetic retinopathy without macular edema right eye |
| t1_dm | ICD10CM | E10.3292 | Type I diabetes mellitus with mild nonproliferative diabetic retinopathy without macular edema left eye |
| t1_dm | ICD10CM | E10.3293 | Type I diabetes mellitus with mild nonproliferative diabetic retinopathy without macular edema bilateral |
| t1_dm | ICD10CM | E10.3299 | Type I diabetes mellitus with mild nonproliferative diabetic retinopathy without macular edema unspecified eye |
| t1_dm | ICD10CM | E10.33 | Type I diabetes mellitus with moderate nonproliferative diabetic retinopathy |
| t1_dm | ICD10CM | E10.331 | Type I diabetes mellitus with moderate nonproliferative diabetic retinopathy with macular edema |
| t1_dm | ICD10CM | E10.3311 | Type I diabetes mellitus with moderate nonproliferative diabetic retinopathy with macular edema right eye |
| t1_dm | ICD10CM | E10.3312 | Type I diabetes mellitus with moderate nonproliferative diabetic retinopathy with macular edema left eye |
| t1_dm | ICD10CM | E10.3313 | Type I diabetes mellitus with moderate nonproliferative diabetic retinopathy with macular edema bilateral |
| t1_dm | ICD10CM | E10.3319 | Type I diabetes mellitus with moderate nonproliferative diabetic retinopathy with macular edema unspecified eye |
| t1_dm | ICD10CM | E10.339 | Type I diabetes mellitus with moderate nonproliferative diabetic retinopathy without macular edema |
| t1_dm | ICD10CM | E10.3391 | Type I diabetes mellitus with moderate nonproliferative diabetic retinopathy without macular edema right eye |
| t1_dm | ICD10CM | E10.3392 | Type I diabetes mellitus with moderate nonproliferative diabetic retinopathy without macular edema left eye |
| t1_dm | ICD10CM | E10.3393 | Type I diabetes mellitus with moderate nonproliferative diabetic retinopathy without macular edema bilateral |
| t1_dm | ICD10CM | E10.3399 | Type I diabetes mellitus with moderate nonproliferative diabetic retinopathy without macular edema unspecified eye |
| t1_dm | ICD10CM | E10.34 | Type I diabetes mellitus with severe nonproliferative diabetic retinopathy |
| t1_dm | ICD10CM | E10.341 | Type I diabetes mellitus with severe nonproliferative diabetic retinopathy with macular edema |
| t1_dm | ICD10CM | E10.3411 | Type I diabetes mellitus with severe nonproliferative diabetic retinopathy with macular edema right eye |
| t1_dm | ICD10CM | E10.3412 | Type I diabetes mellitus with severe nonproliferative diabetic retinopathy with macular edema left eye |
| t1_dm | ICD10CM | E10.3413 | Type I diabetes mellitus with severe nonproliferative diabetic retinopathy with macular edema bilateral |
| t1_dm | ICD10CM | E10.3419 | Type I diabetes mellitus with severe nonproliferative diabetic retinopathy with macular edema unspecified eye |
| t1_dm | ICD10CM | E10.349 | Type I diabetes mellitus with severe nonproliferative diabetic retinopathy without macular edema |
| t1_dm | ICD10CM | E10.3491 | Type I diabetes mellitus with severe nonproliferative diabetic retinopathy without macular edema right eye |
| t1_dm | ICD10CM | E10.3492 | Type I diabetes mellitus with severe nonproliferative diabetic retinopathy without macular edema left eye |
| t1_dm | ICD10CM | E10.3493 | Type I diabetes mellitus with severe nonproliferative diabetic retinopathy without macular edema bilateral |
| t1_dm | ICD10CM | E10.3499 | Type I diabetes mellitus with severe nonproliferative diabetic retinopathy without macular edema unspecified eye |
| t1_dm | ICD10CM | E10.35 | Type I diabetes mellitus with proliferative diabetic retinopathy |
| t1_dm | ICD10CM | E10.351 | Type I diabetes mellitus with proliferative diabetic retinopathy with macular edema |
| t1_dm | ICD10CM | E10.3511 | Type I diabetes mellitus with proliferative diabetic retinopathy with macular edema right eye |
| t1_dm | ICD10CM | E10.3512 | Type I diabetes mellitus with proliferative diabetic retinopathy with macular edema left eye |
| t1_dm | ICD10CM | E10.3513 | Type I diabetes mellitus with proliferative diabetic retinopathy with macular edema bilateral |
| t1_dm | ICD10CM | E10.3519 | Type I diabetes mellitus with proliferative diabetic retinopathy with macular edema unspecified eye |
| t1_dm | ICD10CM | E10.352 | Type I diabetes mellitus with proliferative diabetic retinopathy with traction retinal detachment involving the macula |
| t1_dm | ICD10CM | E10.3521 | Type I diabetes mellitus with proliferative diabetic retinopathy with traction retinal detachment involving the macula right eye |
| t1_dm | ICD10CM | E10.3522 | Type I diabetes mellitus with proliferative diabetic retinopathy with traction retinal detachment involving the macula left eye |
| t1_dm | ICD10CM | E10.3523 | Type I diabetes mellitus with proliferative diabetic retinopathy with traction retinal detachment involving the macula bilateral |
| t1_dm | ICD10CM | E10.3529 | Type I diabetes mellitus with proliferative diabetic retinopathy with traction retinal detachment involving the macula unspecified eye |
| t1_dm | ICD10CM | E10.353 | Type I diabetes mellitus with proliferative diabetic retinopathy with traction retinal detachment not involving the macula |
| t1_dm | ICD10CM | E10.3531 | Type I diabetes mellitus with proliferative diabetic retinopathy with traction retinal detachment not involving the macula right eye |
| t1_dm | ICD10CM | E10.3532 | Type I diabetes mellitus with proliferative diabetic retinopathy with traction retinal detachment not involving the macula left eye |
| t1_dm | ICD10CM | E10.3533 | Type I diabetes mellitus with proliferative diabetic retinopathy with traction retinal detachment not involving the macula bilateral |
| t1_dm | ICD10CM | E10.3539 | Type I diabetes mellitus with proliferative diabetic retinopathy with traction retinal detachment not involving the macula unspecified eye |
| t1_dm | ICD10CM | E10.354 | Type I diabetes mellitus with proliferative diabetic retinopathy with combined traction retinal detachment and rhegmatogenous retinal detachment |
| t1_dm | ICD10CM | E10.3541 | Type I diabetes mellitus with proliferative diabetic retinopathy with combined traction retinal detachment and rhegmatogenous retinal detachment right eye |
| t1_dm | ICD10CM | E10.3542 | Type I diabetes mellitus with proliferative diabetic retinopathy with combined traction retinal detachment and rhegmatogenous retinal detachment left eye |
| t1_dm | ICD10CM | E10.3543 | Type I diabetes mellitus with proliferative diabetic retinopathy with combined traction retinal detachment and rhegmatogenous retinal detachment bilateral |
| t1_dm | ICD10CM | E10.3549 | Type I diabetes mellitus with proliferative diabetic retinopathy with combined traction retinal detachment and rhegmatogenous retinal detachment unspecified eye |
| t1_dm | ICD10CM | E10.355 | Type I diabetes mellitus with stable proliferative diabetic retinopathy |
| t1_dm | ICD10CM | E10.3551 | Type I diabetes mellitus with stable proliferative diabetic retinopathy right eye |
| t1_dm | ICD10CM | E10.3552 | Type I diabetes mellitus with stable proliferative diabetic retinopathy left eye |
| t1_dm | ICD10CM | E10.3553 | Type I diabetes mellitus with stable proliferative diabetic retinopathy bilateral |
| t1_dm | ICD10CM | E10.3559 | Type I diabetes mellitus with stable proliferative diabetic retinopathy unspecified eye |
| t1_dm | ICD10CM | E10.359 | Type I diabetes mellitus with proliferative diabetic retinopathy without macular edema |
| t1_dm | ICD10CM | E10.3591 | Type I diabetes mellitus with proliferative diabetic retinopathy without macular edema right eye |
| t1_dm | ICD10CM | E10.3592 | Type I diabetes mellitus with proliferative diabetic retinopathy without macular edema left eye |
| t1_dm | ICD10CM | E10.3593 | Type I diabetes mellitus with proliferative diabetic retinopathy without macular edema bilateral |
| t1_dm | ICD10CM | E10.3599 | Type I diabetes mellitus with proliferative diabetic retinopathy without macular edema unspecified eye |
| t1_dm | ICD10CM | E10.36 | Type I diabetes mellitus with diabetic cataract |
| t1_dm | ICD10CM | E10.37 | Type I diabetes mellitus with diabetic macular edema, resolved following treatment |
| t1_dm | ICD10CM | E10.37X1 | Type I diabetes mellitus with diabetic macular edema, resolved following treatment right eye |
| t1_dm | ICD10CM | E10.37X2 | Type I diabetes mellitus with diabetic macular edema, resolved following treatment left eye |
| t1_dm | ICD10CM | E10.37X3 | Type I diabetes mellitus with diabetic macular edema, resolved following treatment bilateral |
| t1_dm | ICD10CM | E10.37X9 | Type I diabetes mellitus with diabetic macular edema, resolved following treatment unspecified eye |
| t1_dm | ICD10CM | E10.39 | Type I diabetes mellitus with other diabetic ophthalmic complication |
| t1_dm | ICD10CM | E10.4 | Type I diabetes mellitus with neurological complications |
| t1_dm | ICD10CM | E10.40 | Type I diabetes mellitus with diabetic neuropathy, unspecified |
| t1_dm | ICD10CM | E10.41 | Type I diabetes mellitus with diabetic mononeuropathy |
| t1_dm | ICD10CM | E10.42 | Type I diabetes mellitus with diabetic polyneuropathy |
| t1_dm | ICD10CM | E10.43 | Type I diabetes mellitus with diabetic autonomic (poly)neuropathy |
| t1_dm | ICD10CM | E10.44 | Type I diabetes mellitus with diabetic amyotrophy |
| t1_dm | ICD10CM | E10.49 | Type I diabetes mellitus with other diabetic neurological complication |
| t1_dm | ICD10CM | E10.5 | Type I diabetes mellitus with circulatory complications |
| t1_dm | ICD10CM | E10.51 | Type I diabetes mellitus with diabetic peripheral angiopathy without gangrene |
| t1_dm | ICD10CM | E10.52 | Type I diabetes mellitus with diabetic peripheral angiopathy with gangrene |
| t1_dm | ICD10CM | E10.59 | Type I diabetes mellitus with other circulatory complications |
| t1_dm | ICD10CM | E10.6 | Type I diabetes mellitus with other specified complications |
| t1_dm | ICD10CM | E10.61 | Type I diabetes mellitus with diabetic arthropathy |
| t1_dm | ICD10CM | E10.610 | Type I diabetes mellitus with diabetic neuropathic arthropathy |
| t1_dm | ICD10CM | E10.618 | Type I diabetes mellitus with other diabetic arthropathy |
| t1_dm | ICD10CM | E10.62 | Type I diabetes mellitus with skin complications |
| t1_dm | ICD10CM | E10.620 | Type I diabetes mellitus with diabetic dermatitis |
| t1_dm | ICD10CM | E10.621 | Type I diabetes mellitus with foot ulcer |
| t1_dm | ICD10CM | E10.622 | Type I diabetes mellitus with other skin ulcer |
| t1_dm | ICD10CM | E10.628 | Type I diabetes mellitus with other skin complications |
| t1_dm | ICD10CM | E10.63 | Type I diabetes mellitus with oral complications |
| t1_dm | ICD10CM | E10.630 | Type I diabetes mellitus with periodontal disease |
| t1_dm | ICD10CM | E10.638 | Type I diabetes mellitus with other oral complications |
| t1_dm | ICD10CM | E10.64 | Type I diabetes mellitus with hypoglycemia |
| t1_dm | ICD10CM | E10.641 | Type I diabetes mellitus with hypoglycemiawith coma |
| t1_dm | ICD10CM | E10.649 | Type I diabetes mellitus with hypoglycemia without coma |
| t1_dm | ICD10CM | E10.65 | Type I diabetes mellitus with hyperglycemia |
| t1_dm | ICD10CM | E10.69 | Type I diabetes mellitus with other specified complication |
| t1_dm | ICD10CM | E10.8 | Type I diabetes mellitus with unspecified complications |
| t1_dm | ICD10CM | E10.9 | Type I diabetes mellitus without complications |
| t1_dm | ICD9CM | 250.01 | Diabetes mellitus without mention of complication, type I [juvenile type], not stated as uncontrolled |
| t1_dm | ICD9CM | 250.03 | Diabetes mellitus without mention of complication, type I [juvenile type], uncontrolled |
| t1_dm | ICD9CM | 250.21 | Diabetes with hyperosmolarity, type I [juvenile type], not stated as uncontrolled |
| t1_dm | ICD9CM | 250.23 | Diabetes with hyperosmolarity, type I [juvenile type], uncontrolled |
| t1_dm | ICD9CM | 250.31 | Diabetes with other coma, type I [juvenile type], not stated as uncontrolled |
| t1_dm | ICD9CM | 250.33 | Diabetes with other coma, type I [juvenile type], uncontrolled |
| t1_dm | ICD9CM | 250.41 | Diabetes with renal manifestations, type I [juvenile type], not stated as uncontrolled |
| t1_dm | ICD9CM | 250.43 | Diabetes with renal manifestations, type I [juvenile type], uncontrolled |
| t1_dm | ICD9CM | 250.51 | Diabetes with ophthalmic manifestations, type I [juvenile type], not stated as uncontrolled |
| t1_dm | ICD9CM | 250.53 | Diabetes with ophthalmic manifestations, type I [juvenile type], uncontrolled |
| t1_dm | ICD9CM | 250.61 | Diabetes with neurological manifestations, type I [juvenile type], not stated as uncontrolled |
| t1_dm | ICD9CM | 250.63 | Diabetes with neurological manifestations, type I [juvenile type], uncontrolled |
| t1_dm | ICD9CM | 250.71 | Diabetes with peripheral circulatory disorders, type I [juvenile type], not stated as uncontrolled |
| t1_dm | ICD9CM | 250.73 | Diabetes with peripheral circulatory disorders, type I [juvenile type], uncontrolled |
| t1_dm | ICD9CM | 250.81 | Diabetes with other specified manifestations, type I [juvenile type], not stated as uncontrolled |
| t1_dm | ICD9CM | 250.83 | Diabetes with other specified manifestations, type I [juvenile type], uncontrolled |
| t1_dm | ICD9CM | 250.91 | Diabetes with unspecified complication, type I [juvenile type], not stated as uncontrolled |
| t1_dm | ICD9CM | 250.93 | Diabetes with unspecified complication, type I [juvenile type], uncontrolled |
| t1_dm | ICD9CM | 250.11 | Diabetes with ketoacidosis, type I [juvenile type], not stated as uncontrolled |
| t1_dm | ICD9CM | 250.13 | Diabetes with ketoacidosis, type I [juvenile type], uncontrolled |
| t2_dm | ICD10CM | E11 | Type II diabetes mellitus |
| t2_dm | ICD10CM | E11.0 | Type II diabetes mellitus with hyperosmolarity |
| t2_dm | ICD10CM | E11.00 | Type II diabetes mellitus with hyperosmolarity without nonketotic hyperglycemic-hyperosmolar coma (NKHHC) |
| t2_dm | ICD10CM | E11.01 | Type II diabetes mellitus with hyperosmolarity with coma |
| t2_dm | ICD10CM | E11.1 | Type II diabetes mellitus with ketoacidosis |
| t2_dm | ICD10CM | E11.10 | Type II diabetes mellitus with ketoacidosis without coma |
| t2_dm | ICD10CM | E11.11 | Type II diabetes mellitus with ketoacidosis with coma |
| t2_dm | ICD10CM | E11.2 | Type II diabetes mellitus with kidney complications |
| t2_dm | ICD10CM | E11.21 | Type II diabetes mellitus with diabetic nephropathy |
| t2_dm | ICD10CM | E11.22 | Type II diabetes mellitus with diabetic chronic kidney disease |
| t2_dm | ICD10CM | E11.29 | Type II diabetes mellitus with other diabetic kidney complication |
| t2_dm | ICD10CM | E11.3 | Type II diabetes mellitus with ophthalmic complications |
| t2_dm | ICD10CM | E11.31 | Type II diabetes mellitus with unspecified diabetic retinopathy |
| t2_dm | ICD10CM | E11.311 | Type II diabetes mellitus with unspecified diabetic retinopathy with macular edema |
| t2_dm | ICD10CM | E11.319 | Type II diabetes mellitus with unspecified diabetic retinopathy without macular edema |
| t2_dm | ICD10CM | E11.32 | Type II diabetes mellitus with mild nonproliferative diabetic retinopathy |
| t2_dm | ICD10CM | E11.321 | Type II diabetes mellitus with mild nonproliferative diabetic retinopathy with macular edema |
| t2_dm | ICD10CM | E11.3211 | Type II diabetes mellitus with mild nonproliferative diabetic retinopathy with macular edema, right eye |
| t2_dm | ICD10CM | E11.3212 | Type II diabetes mellitus with mild nonproliferative diabetic retinopathy with macular edema, left eye |
| t2_dm | ICD10CM | E11.3213 | Type II diabetes mellitus with mild nonproliferative diabetic retinopathy with macular edema, bilateral |
| t2_dm | ICD10CM | E11.3219 | Type II diabetes mellitus with mild nonproliferative diabetic retinopathy with macular edema, unspecified eye |
| t2_dm | ICD10CM | E11.329 | Type II diabetes mellitus with mild nonproliferative diabetic retinopathy without macular edema |
| t2_dm | ICD10CM | E11.3291 | Type II diabetes mellitus with mild nonproliferative diabetic retinopathy without macular edema, right eye |
| t2_dm | ICD10CM | E11.3292 | Type II diabetes mellitus with mild nonproliferative diabetic retinopathy without macular edema, left eye |
| t2_dm | ICD10CM | E11.3293 | Type II diabetes mellitus with mild nonproliferative diabetic retinopathy without macular edema, bilateral |
| t2_dm | ICD10CM | E11.3299 | Type II diabetes mellitus with mild nonproliferative diabetic retinopathy without macular edema, unspecified eye |
| t2_dm | ICD10CM | E11.33 | Type II diabetes mellitus with moderate nonproliferative diabetic retinopathy |
| t2_dm | ICD10CM | E11.331 | Type II diabetes mellitus with moderate nonproliferative diabetic retinopathy with macular edema |
| t2_dm | ICD10CM | E11.3311 | Type II diabetes mellitus with moderate nonproliferative diabetic retinopathy with macular edema, right eye |
| t2_dm | ICD10CM | E11.3312 | Type II diabetes mellitus with moderate nonproliferative diabetic retinopathy with macular edema, left eye |
| t2_dm | ICD10CM | E11.3313 | Type II diabetes mellitus with moderate nonproliferative diabetic retinopathy with macular edema, bilateral |
| t2_dm | ICD10CM | E11.3319 | Type II diabetes mellitus with moderate nonproliferative diabetic retinopathy with macular edema, unspecified eye |
| t2_dm | ICD10CM | E11.339 | Type II diabetes mellitus with moderate nonproliferative diabetic retinopathy without macular edema |
| t2_dm | ICD10CM | E11.3391 | Type II diabetes mellitus with moderate nonproliferative diabetic retinopathy without macular edema, right eye |
| t2_dm | ICD10CM | E11.3392 | Type II diabetes mellitus with moderate nonproliferative diabetic retinopathy without macular edema, left eye |
| t2_dm | ICD10CM | E11.3393 | Type II diabetes mellitus with moderate nonproliferative diabetic retinopathy without macular edema, bilateral |
| t2_dm | ICD10CM | E11.3399 | Type II diabetes mellitus with moderate nonproliferative diabetic retinopathy without macular edema, unspecified eye |
| t2_dm | ICD10CM | E11.34 | Type II diabetes mellitus with severe nonproliferative diabetic retinopathy |
| t2_dm | ICD10CM | E11.341 | Type II diabetes mellitus with severe nonproliferative diabetic retinopathy with macular edema |
| t2_dm | ICD10CM | E11.3411 | Type II diabetes mellitus with severe nonproliferative diabetic retinopathy with macular edema, right eye |
| t2_dm | ICD10CM | E11.3412 | Type II diabetes mellitus with severe nonproliferative diabetic retinopathy with macular edema, left eye |
| t2_dm | ICD10CM | E11.3413 | Type II diabetes mellitus with severe nonproliferative diabetic retinopathy with macular edema, bilateral |
| t2_dm | ICD10CM | E11.3419 | Type II diabetes mellitus with severe nonproliferative diabetic retinopathy with macular edema, unspecified eye |
| t2_dm | ICD10CM | E11.349 | Type II diabetes mellitus with severe nonproliferative diabetic retinopathy without macular edema |
| t2_dm | ICD10CM | E11.3491 | Type II diabetes mellitus with severe nonproliferative diabetic retinopathy without macular edema, right eye |
| t2_dm | ICD10CM | E11.3492 | Type II diabetes mellitus with severe nonproliferative diabetic retinopathy without macular edema, left eye |
| t2_dm | ICD10CM | E11.3493 | Type II diabetes mellitus with severe nonproliferative diabetic retinopathy without macular edema, bilateral |
| t2_dm | ICD10CM | E11.3499 | Type II diabetes mellitus with severe nonproliferative diabetic retinopathy without macular edema, unspecified eye |
| t2_dm | ICD10CM | E11.35 | Type II diabetes mellitus with proliferative diabetic retinopathy |
| t2_dm | ICD10CM | E11.351 | Type II diabetes mellitus with proliferative diabetic retinopathy with macular edema |
| t2_dm | ICD10CM | E11.3511 | Type II diabetes mellitus with proliferative diabetic retinopathy with macular edema, right eye |
| t2_dm | ICD10CM | E11.3512 | Type II diabetes mellitus with proliferative diabetic retinopathy with macular edema, left eye |
| t2_dm | ICD10CM | E11.3513 | Type II diabetes mellitus with proliferative diabetic retinopathy with macular edema, bilateral |
| t2_dm | ICD10CM | E11.3519 | Type II diabetes mellitus with proliferative diabetic retinopathy with macular edema, unspecified eye |
| t2_dm | ICD10CM | E11.352 | Type II diabetes mellitus with proliferative diabetic retinopathy with traction retinal detachment involving the macula |
| t2_dm | ICD10CM | E11.3521 | Type II diabetes mellitus with proliferative diabetic retinopathy with traction retinal detachment involving the macula, right eye |
| t2_dm | ICD10CM | E11.3522 | Type II diabetes mellitus with proliferative diabetic retinopathy with traction retinal detachment involving the macula, left eye |
| t2_dm | ICD10CM | E11.3523 | Type II diabetes mellitus with proliferative diabetic retinopathy with traction retinal detachment involving the macula, bilateral |
| t2_dm | ICD10CM | E11.3529 | Type II diabetes mellitus with proliferative diabetic retinopathy with traction retinal detachment involving the macula, unspecified eye |
| t2_dm | ICD10CM | E11.353 | Type II diabetes mellitus with proliferative diabetic retinopathy with traction retinal detachment not involving the macula |
| t2_dm | ICD10CM | E11.3531 | Type II diabetes mellitus with proliferative diabetic retinopathy with traction retinal detachment not involving the macula, right eye |
| t2_dm | ICD10CM | E11.3532 | Type II diabetes mellitus with proliferative diabetic retinopathy with traction retinal detachment not involving the macula, left eye |
| t2_dm | ICD10CM | E11.3533 | Type II diabetes mellitus with proliferative diabetic retinopathy with traction retinal detachment not involving the macula, bilateral |
| t2_dm | ICD10CM | E11.3539 | Type II diabetes mellitus with proliferative diabetic retinopathy with traction retinal detachment not involving the macula, unspecified eye |
| t2_dm | ICD10CM | E11.354 | Type II diabetes mellitus with proliferative diabetic retinopathy with combined traction retinal detachment and rhegmatogenous retinal detachment |
| t2_dm | ICD10CM | E11.3541 | Type II diabetes mellitus with proliferative diabetic retinopathy with combined traction retinal detachment and rhegmatogenous retinal detachment, right eye |
| t2_dm | ICD10CM | E11.3542 | Type II diabetes mellitus with proliferative diabetic retinopathy with combined traction retinal detachment and rhegmatogenous retinal detachment, left eye |
| t2_dm | ICD10CM | E11.3543 | Type II diabetes mellitus with proliferative diabetic retinopathy with combined traction retinal detachment and rhegmatogenous retinal detachment, bilateral |
| t2_dm | ICD10CM | E11.3549 | Type II diabetes mellitus with proliferative diabetic retinopathy with combined traction retinal detachment and rhegmatogenous retinal detachment, unspecified eye |
| t2_dm | ICD10CM | E11.355 | Type II diabetes mellitus with stable proliferative diabetic retinopathy |
| t2_dm | ICD10CM | E11.3551 | Type II diabetes mellitus with stable proliferative diabetic retinopathy, right eye |
| t2_dm | ICD10CM | E11.3552 | Type II diabetes mellitus with stable proliferative diabetic retinopathy, left eye |
| t2_dm | ICD10CM | E11.3553 | Type II diabetes mellitus with stable proliferative diabetic retinopathy, bilateral |
| t2_dm | ICD10CM | E11.3559 | Type II diabetes mellitus with stable proliferative diabetic retinopathy, unspecified eye |
| t2_dm | ICD10CM | E11.359 | Type II diabetes mellitus with proliferative diabetic retinopathy without macular edema |
| t2_dm | ICD10CM | E11.3591 | Type II diabetes mellitus with proliferative diabetic retinopathy without macular edema, right eye |
| t2_dm | ICD10CM | E11.3592 | Type II diabetes mellitus with proliferative diabetic retinopathy without macular edema, left eye |
| t2_dm | ICD10CM | E11.3593 | Type II diabetes mellitus with proliferative diabetic retinopathy without macular edema, bilateral |
| t2_dm | ICD10CM | E11.3599 | Type II diabetes mellitus with proliferative diabetic retinopathy without macular edema, unspecified eye |
| t2_dm | ICD10CM | E11.36 | Type II diabetes mellitus with diabetic cataract |
| t2_dm | ICD10CM | E11.37 | Type II diabetes mellitus with diabetic macular edema, resolved following treatment |
| t2_dm | ICD10CM | E11.37X1 | Type II diabetes mellitus with diabetic macular edema, resolved following treatment, right eye |
| t2_dm | ICD10CM | E11.37X2 | Type II diabetes mellitus with diabetic macular edema, resolved following treatment, left eye |
| t2_dm | ICD10CM | E11.37X3 | Type II diabetes mellitus with diabetic macular edema, resolved following treatment, bilateral |
| t2_dm | ICD10CM | E11.37X9 | Type II diabetes mellitus with diabetic macular edema, resolved following treatment, unspecified eye |
| t2_dm | ICD10CM | E11.39 | Type II diabetes mellitus with other diabetic ophthalmic complication |
| t2_dm | ICD10CM | E11.4 | Type II diabetes mellitus with neurological complications |
| t2_dm | ICD10CM | E11.40 | Type II diabetes mellitus with diabetic neuropathy, unspecified |
| t2_dm | ICD10CM | E11.41 | Type II diabetes mellitus with diabetic mononeuropathy |
| t2_dm | ICD10CM | E11.42 | Type II diabetes mellitus with diabetic polyneuropathy |
| t2_dm | ICD10CM | E11.43 | Type II diabetes mellitus with diabetic autonomic (poly)neuropathy |
| t2_dm | ICD10CM | E11.44 | Type II diabetes mellitus with diabetic amyotrophy |
| t2_dm | ICD10CM | E11.49 | Type II diabetes mellitus with other diabetic neurological complication |
| t2_dm | ICD10CM | E11.5 | Type II diabetes mellitus with circulatory complications |
| t2_dm | ICD10CM | E11.51 | Type II diabetes mellitus with diabetic peripheral angiopathy without gangrene |
| t2_dm | ICD10CM | E11.52 | Type II diabetes mellitus with diabetic peripheral angiopathy with gangrene |
| t2_dm | ICD10CM | E11.59 | Type II diabetes mellitus with other circulatory complications |
| t2_dm | ICD10CM | E11.6 | Type II diabetes mellitus with other specified complications |
| t2_dm | ICD10CM | E11.61 | Type II diabetes mellitus with diabetic arthropathy |
| t2_dm | ICD10CM | E11.610 | Type II diabetes mellitus with diabetic neuropathic arthropathy |
| t2_dm | ICD10CM | E11.618 | Type II diabetes mellitus with other diabetic arthropathy |
| t2_dm | ICD10CM | E11.62 | Type II diabetes mellitus with skin complications |
| t2_dm | ICD10CM | E11.620 | Type II diabetes mellitus with diabetic dermatitis |
| t2_dm | ICD10CM | E11.621 | Type II diabetes mellitus with foot ulcer |
| t2_dm | ICD10CM | E11.622 | Type II diabetes mellitus with other skin ulcer |
| t2_dm | ICD10CM | E11.628 | Type II diabetes mellitus with other skin complications |
| t2_dm | ICD10CM | E11.63 | Type II diabetes mellitus with oral complications |
| t2_dm | ICD10CM | E11.630 | Type II diabetes mellitus with periodontal disease |
| t2_dm | ICD10CM | E11.638 | Type II diabetes mellitus with other oral complications |
| t2_dm | ICD10CM | E11.64 | Type II diabetes mellitus with hypoglycemia |
| t2_dm | ICD10CM | E11.640 | Type II diabetes mellitus with hypoglycemia without coma |
| t2_dm | ICD10CM | E11.641 | Type II diabetes mellitus with hypoglycemia with coma |
| t2_dm | ICD10CM | E11.649 | Type II diabetes mellitus with hypoglycemia without coma |
| t2_dm | ICD10CM | E11.65 | Type II diabetes mellitus with hyperglycemia |
| t2_dm | ICD10CM | E11.69 | Type II diabetes mellitus with other specified complication |
| t2_dm | ICD10CM | E11.8 | Type II diabetes mellitus with unspecified complications |
| t2_dm | ICD10CM | E11.9 | Type II diabetes mellitus without complications |
| dm_unspec | ICD9CM | 362.07 | Diabetic macular edema |
| dm_unspec | CPT4 | 67228 | Treatment of extensive or progressive retinopathy (eg, diabetic retinopathy), photocoagulation |
| dm_unspec | ICD9CM | 362.04 | Mild nonproliferative diabetic retinopathy |
| dm_unspec | ICD9CM | 362.05 | Moderate nonproliferative diabetic retinopathy |
| dm_unspec | ICD9CM | 357.2 | Polyneuropathy in diabetes |
| dm_unspec | ICD9CM | 362.01 | Background diabetic retinopathy |
| dm_unspec | ICD9CM | 362.03 | Nonproliferative diabetic retinopathy NOS |
| dm_unspec | ICD9CM | 362.02 | Proliferative diabetic retinopathy |
| dm_unspec | ICD9CM | 362.06 | Severe nonproliferative diabetic retinopathy |
| dm_unspec | ICD9CM | 250.00 | Diabetes mellitus without mention of complication, type II or unspecified type, not stated as uncontrolled |
| dm_unspec | ICD9CM | 250.02 | Diabetes mellitus without mention of complication, type II or unspecified type, uncontrolled |
| dm_unspec | ICD9CM | 250.20 | Diabetes with hyperosmolarity, type II or unspecified type, not stated as uncontrolled |
| dm_unspec | ICD9CM | 250.22 | Diabetes with hyperosmolarity, type II or unspecified type, uncontrolled |
| dm_unspec | ICD9CM | 250.30 | Diabetes with other coma, type II or unspecified type, not stated as uncontrolled |
| dm_unspec | ICD9CM | 250.32 | Diabetes with other coma, type II or unspecified type, uncontrolled |
| dm_unspec | ICD9CM | 250.40 | Diabetes with renal manifestations, type II or unspecified type, not stated as uncontrolled |
| dm_unspec | ICD9CM | 250.42 | Diabetes with renal manifestations, type II or unspecified type, uncontrolled |
| dm_unspec | ICD9CM | 250.50 | Diabetes with ophthalmic manifestations, type II or unspecified type, not stated as uncontrolled |
| dm_unspec | ICD9CM | 250.52 | Diabetes with ophthalmic manifestations, type II or unspecified type, uncontrolled |
| dm_unspec | ICD9CM | 250.60 | Diabetes with neurological manifestations, type II or unspecified type, not stated as uncontrolled |
| dm_unspec | ICD9CM | 250.62 | Diabetes with neurological manifestations, type II or unspecified type, uncontrolled |
| dm_unspec | ICD9CM | 250.7 | Diabetes with peripheral circulatory disorders, type II or unspecified type, not stated as uncontrolled |
| dm_unspec | ICD9CM | 250.72 | Diabetes with peripheral circulatory disorders, type II or unspecified type, uncontrolled |
| dm_unspec | ICD9CM | 250.80 | Diabetes with other specified manifestations, type II or unspecified type, not stated as uncontrolled |
| dm_unspec | ICD9CM | 250.82 | Diabetes with other specified manifestations, type II or unspecified type, uncontrolled |
| dm_unspec | ICD9CM | 250.90 | Diabetes with unspecified complication, type II or unspecified type, not stated as uncontrolled |
| dm_unspec | ICD9CM | 250.92 | Diabetes with unspecified complication, type II or unspecified type, uncontrolled |
| dm_unspec | ICD9CM | 250.10 | Diabetes with ketoacidosis, type II or unspecified type, not stated as uncontrolled |
| dm_unspec | ICD9CM | 250.12 | Diabetes with ketoacidosis, type II or unspecified type, uncontrolled |
| dm_unspec | ICD9CM | 250.40 | Diabetes with renal manifestations, type II or unspecified type, not stated as uncontrolled |
| dm_unspec | ICD9CM | 250 | Diabetes mellitus without mention of complication |
| dm_unspec | ICD9CM | 250.2 | Diabetes mellitus with hyperosmolarity |
| dm_unspec | ICD9CM | 250.3 | Diabetes with other coma |
| dm_unspec | ICD9CM | 250.4 | Diabetes with renal manifestations |
| dm_unspec | ICD9CM | 362.07 | Diabetic macular edema |
| dm_unspec | ICD10CM | E09 | Drug or chemical induced diabetes mellitus |
| dm_unspec | ICD10CM | E09.0 | Drug or chemical induced diabetes mellitus with hyperosmolarity |
| dm_unspec | ICD10CM | E09.00 | Drug or chemical induced diabetes mellitus with hyperosmolarity without nonketotic hyperglycemic-hyperosmolar coma (NKHHC) |
| dm_unspec | ICD10CM | E09.01 | Drug or chemical induced diabetes mellitus with hyperosmolarity with coma |
| dm_unspec | ICD10CM | E09.1 | Drug or chemical induced diabetes mellitus with ketoacidosis |
| dm_unspec | ICD10CM | E09.10 | Drug or chemical induced diabetes mellitus with ketoacidosis without coma |
| dm_unspec | ICD10CM | E09.11 | Drug or chemical induced diabetes mellitus with ketoacidosis with coma |
| dm_unspec | ICD10CM | E09.2 | Drug or chemical induced diabetes mellitus with kidney complications |
| dm_unspec | ICD10CM | E09.21 | Drug or chemical induced diabetes mellitus with diabetic nephropathy |
| dm_unspec | ICD10CM | E09.22 | Drug or chemical induced diabetes mellitus with diabetic chronic kidney disease |
| dm_unspec | ICD10CM | E09.29 | Drug or chemical induced diabetes mellitus with other diabetic kidney complication |
| dm_unspec | ICD10CM | E09.3 | Drug or chemical induced diabetes mellitus with ophthalmic complications |
| dm_unspec | ICD10CM | E09.31 | Drug or chemical induced diabetes mellitus with unspecified diabetic retinopathy |
| dm_unspec | ICD10CM | E09.311 | Drug or chemical induced diabetes mellitus with unspecified diabetic retinopathy with macular edema |
| dm_unspec | ICD10CM | E09.319 | Drug or chemical induced diabetes mellitus with unspecified diabetic retinopathy without macular edema |
| dm_unspec | ICD10CM | E09.32 | Drug or chemical induced diabetes mellitus with mild nonproliferative diabetic retinopathy |
| dm_unspec | ICD10CM | E09.321 | Drug or chemical induced diabetes mellitus with mild nonproliferative diabetic retinopathy with macular edema |
| dm_unspec | ICD10CM | E09.3211 | Drug or chemical induced diabetes mellitus with mild nonproliferative diabetic retinopathy with macular edema, right eye |
| dm_unspec | ICD10CM | E09.3212 | Drug or chemical induced diabetes mellitus with mild nonproliferative diabetic retinopathy with macular edema, left eye |
| dm_unspec | ICD10CM | E09.3213 | Drug or chemical induced diabetes mellitus with mild nonproliferative diabetic retinopathy with macular edema, bilateral |
| dm_unspec | ICD10CM | E09.3219 | Drug or chemical induced diabetes mellitus with mild nonproliferative diabetic retinopathy with macular edema, unspecified eye |
| dm_unspec | ICD10CM | E09.329 | Drug or chemical induced diabetes mellitus with mild nonproliferative diabetic retinopathy without macular edema |
| dm_unspec | ICD10CM | E09.3291 | Drug or chemical induced diabetes mellitus with mild nonproliferative diabetic retinopathy without macular edema, right eye |
| dm_unspec | ICD10CM | E09.3292 | Drug or chemical induced diabetes mellitus with mild nonproliferative diabetic retinopathy without macular edema, left eye |
| dm_unspec | ICD10CM | E09.3293 | Drug or chemical induced diabetes mellitus with mild nonproliferative diabetic retinopathy without macular edema, bilateral |
| dm_unspec | ICD10CM | E09.3299 | Drug or chemical induced diabetes mellitus with mild nonproliferative diabetic retinopathy without macular edema, unspecified eye |
| dm_unspec | ICD10CM | E09.33 | Drug or chemical induced diabetes mellitus with moderate nonproliferative diabetic retinopathy |
| dm_unspec | ICD10CM | E09.331 | Drug or chemical induced diabetes mellitus with moderate nonproliferative diabetic retinopathy with macular edema |
| dm_unspec | ICD10CM | E09.3311 | Drug or chemical induced diabetes mellitus with moderate nonproliferative diabetic retinopathy with macular edema, right eye |
| dm_unspec | ICD10CM | E09.3312 | Drug or chemical induced diabetes mellitus with moderate nonproliferative diabetic retinopathy with macular edema, left eye |
| dm_unspec | ICD10CM | E09.3313 | Drug or chemical induced diabetes mellitus with moderate nonproliferative diabetic retinopathy with macular edema, bilateral |
| dm_unspec | ICD10CM | E09.3319 | Drug or chemical induced diabetes mellitus with moderate nonproliferative diabetic retinopathy with macular edema, unspecified eye |
| dm_unspec | ICD10CM | E09.339 | Drug or chemical induced diabetes mellitus with moderate nonproliferative diabetic retinopathy without macular edema |
| dm_unspec | ICD10CM | E09.3391 | Drug or chemical induced diabetes mellitus with moderate nonproliferative diabetic retinopathy without macular edema, right eye |
| dm_unspec | ICD10CM | E09.3392 | Drug or chemical induced diabetes mellitus with moderate nonproliferative diabetic retinopathy without macular edema, left eye |
| dm_unspec | ICD10CM | E09.3393 | Drug or chemical induced diabetes mellitus with moderate nonproliferative diabetic retinopathy without macular edema, bilateral |
| dm_unspec | ICD10CM | E09.3399 | Drug or chemical induced diabetes mellitus with moderate nonproliferative diabetic retinopathy without macular edema, unspecified eye |
| dm_unspec | ICD10CM | E09.34 | Drug or chemical induced diabetes mellitus with severe nonproliferative diabetic retinopathy |
| dm_unspec | ICD10CM | E09.341 | Drug or chemical induced diabetes mellitus with severe nonproliferative diabetic retinopathy with macular edema |
| dm_unspec | ICD10CM | E09.3411 | Drug or chemical induced diabetes mellitus with severe nonproliferative diabetic retinopathy with macular edema, right eye |
| dm_unspec | ICD10CM | E09.3412 | Drug or chemical induced diabetes mellitus with severe nonproliferative diabetic retinopathy with macular edema, left eye |
| dm_unspec | ICD10CM | E09.3413 | Drug or chemical induced diabetes mellitus with severe nonproliferative diabetic retinopathy with macular edema, bilateral |
| dm_unspec | ICD10CM | E09.3419 | Drug or chemical induced diabetes mellitus with severe nonproliferative diabetic retinopathy with macular edema, unspecified eye |
| dm_unspec | ICD10CM | E09.349 | Drug or chemical induced diabetes mellitus with severe nonproliferative diabetic retinopathy without macular edema |
| dm_unspec | ICD10CM | E09.3491 | Drug or chemical induced diabetes mellitus with severe nonproliferative diabetic retinopathy without macular edema, right eye |
| dm_unspec | ICD10CM | E09.3492 | Drug or chemical induced diabetes mellitus with severe nonproliferative diabetic retinopathy without macular edema, left eye |
| dm_unspec | ICD10CM | E09.3493 | Drug or chemical induced diabetes mellitus with severe nonproliferative diabetic retinopathy without macular edema, bilateral |
| dm_unspec | ICD10CM | E09.3499 | Drug or chemical induced diabetes mellitus with severe nonproliferative diabetic retinopathy without macular edema, unspecified eye |
| dm_unspec | ICD10CM | E09.35 | Drug or chemical induced diabetes mellitus with proliferative diabetic retinopathy |
| dm_unspec | ICD10CM | E09.351 | Drug or chemical induced diabetes mellitus with proliferative diabetic retinopathy with macular edema |
| dm_unspec | ICD10CM | E09.3511 | Drug or chemical induced diabetes mellitus with proliferative diabetic retinopathy with macular edema, right eye |
| dm_unspec | ICD10CM | E09.3512 | Drug or chemical induced diabetes mellitus with proliferative diabetic retinopathy with macular edema, left eye |
| dm_unspec | ICD10CM | E09.3513 | Drug or chemical induced diabetes mellitus with proliferative diabetic retinopathy with macular edema, bilateral |
| dm_unspec | ICD10CM | E09.3519 | Drug or chemical induced diabetes mellitus with proliferative diabetic retinopathy with macular edema, unspecified eye |
| dm_unspec | ICD10CM | E09.352 | Drug or chemical induced diabetes mellitus with proliferative diabetic retinopathy with traction retinal detachment involving the macula |
| dm_unspec | ICD10CM | E09.3521 | Drug or chemical induced diabetes mellitus with proliferative diabetic retinopathy with traction retinal detachment involving the macula, right eye |
| dm_unspec | ICD10CM | E09.3522 | Drug or chemical induced diabetes mellitus with proliferative diabetic retinopathy with traction retinal detachment involving the macula, left eye |
| dm_unspec | ICD10CM | E09.3523 | Drug or chemical induced diabetes mellitus with proliferative diabetic retinopathy with traction retinal detachment involving the macula, bilateral |
| dm_unspec | ICD10CM | E09.3529 | Drug or chemical induced diabetes mellitus with proliferative diabetic retinopathy with traction retinal detachment involving the macula, unspecified eye |
| dm_unspec | ICD10CM | E09.353 | Drug or chemical induced diabetes mellitus with proliferative diabetic retinopathy with traction retinal detachment not involving the macula |
| dm_unspec | ICD10CM | E09.3531 | Drug or chemical induced diabetes mellitus with proliferative diabetic retinopathy with traction retinal detachment not involving the macula, right eye |
| dm_unspec | ICD10CM | E09.3532 | Drug or chemical induced diabetes mellitus with proliferative diabetic retinopathy with traction retinal detachment not involving the macula, left eye |
| dm_unspec | ICD10CM | E09.3533 | Drug or chemical induced diabetes mellitus with proliferative diabetic retinopathy with traction retinal detachment not involving the macula, bilateral |
| dm_unspec | ICD10CM | E09.3539 | Drug or chemical induced diabetes mellitus with proliferative diabetic retinopathy with traction retinal detachment not involving the macula, unspecified eye |
| dm_unspec | ICD10CM | E09.354 | Drug or chemical induced diabetes mellitus with proliferative diabetic retinopathy with combined traction retinal detachment and rhegmatogenous retinal detachment |
| dm_unspec | ICD10CM | E09.3541 | Drug or chemical induced diabetes mellitus with proliferative diabetic retinopathy with combined traction retinal detachment and rhegmatogenous retinal detachment, right eye |
| dm_unspec | ICD10CM | E09.3542 | Drug or chemical induced diabetes mellitus with proliferative diabetic retinopathy with combined traction retinal detachment and rhegmatogenous retinal detachment, left eye |
| dm_unspec | ICD10CM | E09.3543 | Drug or chemical induced diabetes mellitus with proliferative diabetic retinopathy with combined traction retinal detachment and rhegmatogenous retinal detachment, bilateral |
| dm_unspec | ICD10CM | E09.3549 | Drug or chemical induced diabetes mellitus with proliferative diabetic retinopathy with combined traction retinal detachment and rhegmatogenous retinal detachment, unspecified eye |
| dm_unspec | ICD10CM | E09.355 | Drug or chemical induced diabetes mellitus with stable proliferative diabetic retinopathy |
| dm_unspec | ICD10CM | E09.3551 | Drug or chemical induced diabetes mellitus with stable proliferative diabetic retinopathy, right eye |
| dm_unspec | ICD10CM | E09.3552 | Drug or chemical induced diabetes mellitus with stable proliferative diabetic retinopathy, left eye |
| dm_unspec | ICD10CM | E09.3553 | Drug or chemical induced diabetes mellitus with stable proliferative diabetic retinopathy, bilateral |
| dm_unspec | ICD10CM | E09.3559 | Drug or chemical induced diabetes mellitus with stable proliferative diabetic retinopathy, unspecified eye |
| dm_unspec | ICD10CM | E09.359 | Drug or chemical induced diabetes mellitus with proliferative diabetic retinopathy without macular edema |
| dm_unspec | ICD10CM | E09.3591 | Drug or chemical induced diabetes mellitus with proliferative diabetic retinopathy without macular edema, right eye |
| dm_unspec | ICD10CM | E09.3592 | Drug or chemical induced diabetes mellitus with proliferative diabetic retinopathy without macular edema, left eye |
| dm_unspec | ICD10CM | E09.3593 | Drug or chemical induced diabetes mellitus with proliferative diabetic retinopathy without macular edema, bilateral |
| dm_unspec | ICD10CM | E09.3599 | Drug or chemical induced diabetes mellitus with proliferative diabetic retinopathy without macular edema, unspecified eye |
| dm_unspec | ICD10CM | E09.36 | Drug or chemical induced diabetes mellitus with diabetic cataract |
| dm_unspec | ICD10CM | E09.37 | Drug or chemical induced diabetes mellitus with diabetic macular edema, resolved following treatment |
| dm_unspec | ICD10CM | E09.37X1 | Drug or chemical induced diabetes mellitus with diabetic macular edema, resolved following treatment, right eye |
| dm_unspec | ICD10CM | E09.37X2 | Drug or chemical induced diabetes mellitus with diabetic macular edema, resolved following treatment, left eye |
| dm_unspec | ICD10CM | E09.37X3 | Drug or chemical induced diabetes mellitus with diabetic macular edema, resolved following treatment, bilateral |
| dm_unspec | ICD10CM | E09.37X9 | Drug or chemical induced diabetes mellitus with diabetic macular edema, resolved following treatment, unspecified eye |
| dm_unspec | ICD10CM | E09.39 | Drug or chemical induced diabetes mellitus with other diabetic ophthalmic complication |
| dm_unspec | ICD10CM | E09.4 | Drug or chemical induced diabetes mellitus with neurological complications |
| dm_unspec | ICD10CM | E09.40 | Drug or chemical induced diabetes mellitus with neurological complications with diabetic neuropathy, unspecified |
| dm_unspec | ICD10CM | E09.41 | Drug or chemical induced diabetes mellitus with neurological complications with diabetic mononeuropathy |
| dm_unspec | ICD10CM | E09.42 | Drug or chemical induced diabetes mellitus with neurological complications with diabetic polyneuropathy |
| dm_unspec | ICD10CM | E09.43 | Drug or chemical induced diabetes mellitus with neurological complications with diabetic autonomic (poly)neuropathy |
| dm_unspec | ICD10CM | E09.44 | Drug or chemical induced diabetes mellitus with neurological complications with diabetic amyotrophy |
| dm_unspec | ICD10CM | E09.49 | Drug or chemical induced diabetes mellitus with neurological complications with other diabetic neurological complication |
| dm_unspec | ICD10CM | E09.5 | Drug or chemical induced diabetes mellitus with circulatory complications |
| dm_unspec | ICD10CM | E09.51 | Drug or chemical induced diabetes mellitus with diabetic peripheral angiopathy without gangrene |
| dm_unspec | ICD10CM | E09.52 | Drug or chemical induced diabetes mellitus with diabetic peripheral angiopathy with gangrene |
| dm_unspec | ICD10CM | E09.59 | Drug or chemical induced diabetes mellitus with other circulatory complications |
| dm_unspec | ICD10CM | E09.6 | Drug or chemical induced diabetes mellitus with other specified complications |
| dm_unspec | ICD10CM | E09.61 | Drug or chemical induced diabetes mellitus with diabetic arthropathy |
| dm_unspec | ICD10CM | E09.610 | Drug or chemical induced diabetes mellitus with diabetic neuropathic arthropathy |
| dm_unspec | ICD10CM | E09.618 | Drug or chemical induced diabetes mellitus with other diabetic arthropathy |
| dm_unspec | ICD10CM | E09.62 | Drug or chemical induced diabetes mellitus with skin complications |
| dm_unspec | ICD10CM | E09.620 | Drug or chemical induced diabetes mellitus with diabetic dermatitis |
| dm_unspec | ICD10CM | E09.621 | Drug or chemical induced diabetes mellitus with foot ulcer |
| dm_unspec | ICD10CM | E09.622 | Drug or chemical induced diabetes mellitus with other skin ulcer |
| dm_unspec | ICD10CM | E09.628 | Drug or chemical induced diabetes mellitus with other skin complications |
| dm_unspec | ICD10CM | E09.63 | Drug or chemical induced diabetes mellitus with oral complications |
| dm_unspec | ICD10CM | E09.630 | Drug or chemical induced diabetes mellitus with periodontal disease |
| dm_unspec | ICD10CM | E09.638 | Drug or chemical induced diabetes mellitus with other oral complications |
| dm_unspec | ICD10CM | E09.64 | Drug or chemical induced diabetes mellitus with hypoglycemia |
| dm_unspec | ICD10CM | E09.640 | Drug or chemical induced diabetes mellitus with hypoglycemia without coma |
| dm_unspec | ICD10CM | E09.641 | Drug or chemical induced diabetes mellitus with hypoglycemia with coma |
| dm_unspec | ICD10CM | E09.649 | Drug or chemical induced diabetes mellitus with hypoglycemia without coma |
| dm_unspec | ICD10CM | E09.65 | Drug or chemical induced diabetes mellitus with hyperglycemia |
| dm_unspec | ICD10CM | E09.69 | Drug or chemical induced diabetes mellitus with other specified complication |
| dm_unspec | ICD10CM | E09.8 | Drug or chemical induced diabetes mellitus with unspecified complications |
| dm_unspec | ICD10CM | E09.9 | Drug or chemical induced diabetes mellitus without complications |
| dm_unspec | ICD10CM | E13 | Other specified diabetes mellitus |
| dm_unspec | ICD10CM | E13.0 | Other specified diabetes mellitus with hyperosmolarity |
| dm_unspec | ICD10CM | E13.00 | Other specified diabetes mellitus with hyperosmolarity without nonketotic hyperglycemic-hyperosmolar coma (NKHHC) |
| dm_unspec | ICD10CM | E13.01 | Other specified diabetes mellitus with hyperosmolarity with coma |
| dm_unspec | ICD10CM | E13.1 | Other specified diabetes mellitus with ketoacidosis |
| dm_unspec | ICD10CM | E13.10 | Other specified diabetes mellitus with ketoacidosis without coma |
| dm_unspec | ICD10CM | E13.11 | Other specified diabetes mellitus with ketoacidosis with coma |
| dm_unspec | ICD10CM | E13.2 | Other specified diabetes mellitus with kidney complications |
| dm_unspec | ICD10CM | E13.21 | Other specified diabetes mellitus with diabetic nephropathy |
| dm_unspec | ICD10CM | E13.22 | Other specified diabetes mellitus with diabetic chronic kidney disease |
| dm_unspec | ICD10CM | E13.29 | Other specified diabetes mellitus with other diabetic kidney complication |
| dm_unspec | ICD10CM | E13.3 | Other specified diabetes mellitus with ophthalmic complications |
| dm_unspec | ICD10CM | E13.31 | Other specified diabetes mellitus with unspecified diabetic retinopathy |
| dm_unspec | ICD10CM | E13.311 | Other specified diabetes mellitus with unspecified diabetic retinopathy with macular edema |
| dm_unspec | ICD10CM | E13.319 | Other specified diabetes mellitus with unspecified diabetic retinopathy without macular edema |
| dm_unspec | ICD10CM | E13.32 | Other specified diabetes mellitus with mild nonproliferative diabetic retinopathy |
| dm_unspec | ICD10CM | E13.321 | Other specified diabetes mellitus with mild nonproliferative diabetic retinopathy with macular edema |
| dm_unspec | ICD10CM | E13.3211 | Other specified diabetes mellitus with mild nonproliferative diabetic retinopathy with macular edema, right eye |
| dm_unspec | ICD10CM | E13.3212 | Other specified diabetes mellitus with mild nonproliferative diabetic retinopathy with macular edema, left eye |
| dm_unspec | ICD10CM | E13.3213 | Other specified diabetes mellitus with mild nonproliferative diabetic retinopathy with macular edema, bilateral |
| dm_unspec | ICD10CM | E13.3219 | Other specified diabetes mellitus with mild nonproliferative diabetic retinopathy with macular edema, unspecified eye |
| dm_unspec | ICD10CM | E13.329 | Other specified diabetes mellitus with mild nonproliferative diabetic retinopathy without macular edema |
| dm_unspec | ICD10CM | E13.3291 | Other specified diabetes mellitus with mild nonproliferative diabetic retinopathy without macular edema, right eye |
| dm_unspec | ICD10CM | E13.3292 | Other specified diabetes mellitus with mild nonproliferative diabetic retinopathy without macular edema, left eye |
| dm_unspec | ICD10CM | E13.3293 | Other specified diabetes mellitus with mild nonproliferative diabetic retinopathy without macular edema, bilateral |
| dm_unspec | ICD10CM | E13.3299 | Other specified diabetes mellitus with mild nonproliferative diabetic retinopathy without macular edema, unspecified eye |
| dm_unspec | ICD10CM | E13.33 | Other specified diabetes mellitus with moderate nonproliferative diabetic retinopathy |
| dm_unspec | ICD10CM | E13.331 | Other specified diabetes mellitus with moderate nonproliferative diabetic retinopathy with macular edema |
| dm_unspec | ICD10CM | E13.3311 | Other specified diabetes mellitus with moderate nonproliferative diabetic retinopathy with macular edema, right eye |
| dm_unspec | ICD10CM | E13.3312 | Other specified diabetes mellitus with moderate nonproliferative diabetic retinopathy with macular edema, left eye |
| dm_unspec | ICD10CM | E13.3313 | Other specified diabetes mellitus with moderate nonproliferative diabetic retinopathy with macular edema, bilateral |
| dm_unspec | ICD10CM | E13.3319 | Other specified diabetes mellitus with moderate nonproliferative diabetic retinopathy with macular edema, unspecified eye |
| dm_unspec | ICD10CM | E13.339 | Other specified diabetes mellitus with moderate nonproliferative diabetic retinopathy without macular edema |
| dm_unspec | ICD10CM | E13.3391 | Other specified diabetes mellitus with moderate nonproliferative diabetic retinopathy without macular edema, right eye |
| dm_unspec | ICD10CM | E13.3392 | Other specified diabetes mellitus with moderate nonproliferative diabetic retinopathy without macular edema, left eye |
| dm_unspec | ICD10CM | E13.3393 | Other specified diabetes mellitus with moderate nonproliferative diabetic retinopathy without macular edema, bilateral |
| dm_unspec | ICD10CM | E13.3399 | Other specified diabetes mellitus with moderate nonproliferative diabetic retinopathy without macular edema, unspecified eye |
| dm_unspec | ICD10CM | E13.34 | Other specified diabetes mellitus with severe nonproliferative diabetic retinopathy |
| dm_unspec | ICD10CM | E13.341 | Other specified diabetes mellitus with severe nonproliferative diabetic retinopathy with macular edema |
| dm_unspec | ICD10CM | E13.3411 | Other specified diabetes mellitus with severe nonproliferative diabetic retinopathy with macular edema, right eye |
| dm_unspec | ICD10CM | E13.3412 | Other specified diabetes mellitus with severe nonproliferative diabetic retinopathy with macular edema, left eye |
| dm_unspec | ICD10CM | E13.3413 | Other specified diabetes mellitus with severe nonproliferative diabetic retinopathy with macular edema, bilateral |
| dm_unspec | ICD10CM | E13.3419 | Other specified diabetes mellitus with severe nonproliferative diabetic retinopathy with macular edema, unspecified eye |
| dm_unspec | ICD10CM | E13.349 | Other specified diabetes mellitus with severe nonproliferative diabetic retinopathy without macular edema |
| dm_unspec | ICD10CM | E13.3491 | Other specified diabetes mellitus with severe nonproliferative diabetic retinopathy without macular edema, right eye |
| dm_unspec | ICD10CM | E13.3492 | Other specified diabetes mellitus with severe nonproliferative diabetic retinopathy without macular edema, left eye |
| dm_unspec | ICD10CM | E13.3493 | Other specified diabetes mellitus with severe nonproliferative diabetic retinopathy without macular edema, bilateral |
| dm_unspec | ICD10CM | E13.3499 | Other specified diabetes mellitus with severe nonproliferative diabetic retinopathy without macular edema, unspecified eye |
| dm_unspec | ICD10CM | E13.35 | Other specified diabetes mellitus with proliferative diabetic retinopathy |
| dm_unspec | ICD10CM | E13.351 | Other specified diabetes mellitus with proliferative diabetic retinopathy with macular edema |
| dm_unspec | ICD10CM | E13.3511 | Other specified diabetes mellitus with proliferative diabetic retinopathy with macular edema, right eye |
| dm_unspec | ICD10CM | E13.3512 | Other specified diabetes mellitus with proliferative diabetic retinopathy with macular edema, left eye |
| dm_unspec | ICD10CM | E13.3513 | Other specified diabetes mellitus with proliferative diabetic retinopathy with macular edema, bilateral |
| dm_unspec | ICD10CM | E13.3519 | Other specified diabetes mellitus with proliferative diabetic retinopathy with macular edema, unspecified eye |
| dm_unspec | ICD10CM | E13.352 | Other specified diabetes mellitus with proliferative diabetic retinopathy with traction retinal detachment involving the macula |
| dm_unspec | ICD10CM | E13.3521 | Other specified diabetes mellitus with proliferative diabetic retinopathy with traction retinal detachment involving the macula, right eye |
| dm_unspec | ICD10CM | E13.3522 | Other specified diabetes mellitus with proliferative diabetic retinopathy with traction retinal detachment involving the macula, left eye |
| dm_unspec | ICD10CM | E13.3523 | Other specified diabetes mellitus with proliferative diabetic retinopathy with traction retinal detachment involving the macula, bilateral |
| dm_unspec | ICD10CM | E13.3529 | Other specified diabetes mellitus with proliferative diabetic retinopathy with traction retinal detachment involving the macula, unspecified eye |
| dm_unspec | ICD10CM | E13.353 | Other specified diabetes mellitus with proliferative diabetic retinopathy with traction retinal detachment not involving the macula |
| dm_unspec | ICD10CM | E13.3531 | Other specified diabetes mellitus with proliferative diabetic retinopathy with traction retinal detachment not involving the macula, right eye |
| dm_unspec | ICD10CM | E13.3532 | Other specified diabetes mellitus with proliferative diabetic retinopathy with traction retinal detachment not involving the macula, left eye |
| dm_unspec | ICD10CM | E13.3533 | Other specified diabetes mellitus with proliferative diabetic retinopathy with traction retinal detachment not involving the macula, bilateral |
| dm_unspec | ICD10CM | E13.3539 | Other specified diabetes mellitus with proliferative diabetic retinopathy with traction retinal detachment not involving the macula, unspecified eye |
| dm_unspec | ICD10CM | E13.354 | Other specified diabetes mellitus with proliferative diabetic retinopathy with combined traction retinal detachment and rhegmatogenous retinal detachment |
| dm_unspec | ICD10CM | E13.3541 | Other specified diabetes mellitus with proliferative diabetic retinopathy with combined traction retinal detachment and rhegmatogenous retinal detachment, right eye |
| dm_unspec | ICD10CM | E13.3542 | Other specified diabetes mellitus with proliferative diabetic retinopathy with combined traction retinal detachment and rhegmatogenous retinal detachment, left eye |
| dm_unspec | ICD10CM | E13.3543 | Other specified diabetes mellitus with proliferative diabetic retinopathy with combined traction retinal detachment and rhegmatogenous retinal detachment, bilateral |
| dm_unspec | ICD10CM | E13.3549 | Other specified diabetes mellitus with proliferative diabetic retinopathy with combined traction retinal detachment and rhegmatogenous retinal detachment, unspecified eye |
| dm_unspec | ICD10CM | E13.355 | Other specified diabetes mellitus with stable proliferative diabetic retinopathy |
| dm_unspec | ICD10CM | E13.3551 | Other specified diabetes mellitus with stable proliferative diabetic retinopathy, right eye |
| dm_unspec | ICD10CM | E13.3552 | Other specified diabetes mellitus with stable proliferative diabetic retinopathy, left eye |
| dm_unspec | ICD10CM | E13.3553 | Other specified diabetes mellitus with stable proliferative diabetic retinopathy, bilateral |
| dm_unspec | ICD10CM | E13.3559 | Other specified diabetes mellitus with stable proliferative diabetic retinopathy, unspecified eye |
| dm_unspec | ICD10CM | E13.359 | Other specified diabetes mellitus with proliferative diabetic retinopathy without macular edema |
| dm_unspec | ICD10CM | E13.3591 | Other specified diabetes mellitus with proliferative diabetic retinopathy without macular edema, right eye |
| dm_unspec | ICD10CM | E13.3592 | Other specified diabetes mellitus with proliferative diabetic retinopathy without macular edema, left eye |
| dm_unspec | ICD10CM | E13.3593 | Other specified diabetes mellitus with proliferative diabetic retinopathy without macular edema, bilateral |
| dm_unspec | ICD10CM | E13.3599 | Other specified diabetes mellitus with proliferative diabetic retinopathy without macular edema, unspecified eye |
| dm_unspec | ICD10CM | E13.36 | Other specified diabetes mellitus with diabetic cataract |
| dm_unspec | ICD10CM | E13.37 | Other specified diabetes mellitus with diabetic macular edema, resolved following treatment |
| dm_unspec | ICD10CM | E13.37X1 | Other specified diabetes mellitus with diabetic macular edema, resolved following treatment, right eye |
| dm_unspec | ICD10CM | E13.37X2 | Other specified diabetes mellitus with diabetic macular edema, resolved following treatment, left eye |
| dm_unspec | ICD10CM | E13.37X3 | Other specified diabetes mellitus with diabetic macular edema, resolved following treatment, bilateral |
| dm_unspec | ICD10CM | E13.37X9 | Other specified diabetes mellitus with diabetic macular edema, resolved following treatment, unspecified eye |
| dm_unspec | ICD10CM | E13.39 | Other specified diabetes mellitus with other diabetic ophthalmic complication |
| dm_unspec | ICD10CM | E13.4 | Other specified diabetes mellitus with neurological complications |
| dm_unspec | ICD10CM | E13.40 | Other specified diabetes mellitus with diabetic neuropathy, unspecified |
| dm_unspec | ICD10CM | E13.41 | Other specified diabetes mellitus with diabetic mononeuropathy |
| dm_unspec | ICD10CM | E13.42 | Other specified diabetes mellitus with diabetic polyneuropathy |
| dm_unspec | ICD10CM | E13.43 | Other specified diabetes mellitus with diabetic autonomic (poly)neuropathy |
| dm_unspec | ICD10CM | E13.44 | Other specified diabetes mellitus with diabetic amyotrophy |
| dm_unspec | ICD10CM | E13.49 | Other specified diabetes mellitus with other diabetic neurological complication |
| dm_unspec | ICD10CM | E13.5 | Other specified diabetes mellitus with circulatory complications |
| dm_unspec | ICD10CM | E13.51 | Other specified diabetes mellitus with diabetic peripheral angiopathy without gangrene |
| dm_unspec | ICD10CM | E13.52 | Other specified diabetes mellitus with diabetic peripheral angiopathy with gangrene |
| dm_unspec | ICD10CM | E13.59 | Other specified diabetes mellitus with other circulatory complications |
| dm_unspec | ICD10CM | E13.6 | Other specified diabetes mellitus with other specified complications |
| dm_unspec | ICD10CM | E13.61 | Other specified diabetes mellitus with diabetic arthropathy |
| dm_unspec | ICD10CM | E13.610 | Other specified diabetes mellitus with diabetic neuropathic arthropathy |
| dm_unspec | ICD10CM | E13.618 | Other specified diabetes mellitus with other diabetic arthropathy |
| dm_unspec | ICD10CM | E13.62 | Other specified diabetes mellitus with skin complications |
| dm_unspec | ICD10CM | E13.620 | Other specified diabetes mellitus with diabetic dermatitis |
| dm_unspec | ICD10CM | E13.621 | Other specified diabetes mellitus with foot ulcer |
| dm_unspec | ICD10CM | E13.622 | Other specified diabetes mellitus with other skin ulcer |
| dm_unspec | ICD10CM | E13.628 | Other specified diabetes mellitus with other skin complications |
| dm_unspec | ICD10CM | E13.63 | Other specified diabetes mellitus with oral complications |
| dm_unspec | ICD10CM | E13.630 | Other specified diabetes mellitus with periodontal disease |
| dm_unspec | ICD10CM | E13.638 | Other specified diabetes mellitus with other oral complications |
| dm_unspec | ICD10CM | E13.64 | Other specified diabetes mellitus with hypoglycemia |
| dm_unspec | ICD10CM | E13.640 | Other specified diabetes mellitus with hypoglycemia without coma |
| dm_unspec | ICD10CM | E13.641 | Other specified diabetes mellitus with hypoglycemia with coma |
| dm_unspec | ICD10CM | E13.649 | Other specified diabetes mellitus with hypoglycemia without coma |
| dm_unspec | ICD10CM | E13.65 | Other specified diabetes mellitus with hyperglycemia |
| dm_unspec | ICD10CM | E13.69 | Other specified diabetes mellitus with other specified complication |
| dm_unspec | ICD10CM | E13.8 | Other specified diabetes mellitus with unspecified complications |
| dm_unspec | ICD10CM | E13.9 | Other specified diabetes mellitus without complications |
| dm_unspec | ICD10CM | E08 | Diabetes mellitus due to underlying condition |
| dm_unspec | ICD10CM | E08.0 | Diabetes mellitus due to underlying condition with hyperosmolarity |
| dm_unspec | ICD10CM | E08.00 | Diabetes mellitus due to underlying condition with hyperosmolarity without nonketotic hyperglycemic-hyperosmolar coma (NKHHC) |
| dm_unspec | ICD10CM | E08.01 | Diabetes mellitus due to underlying condition with hyperosmolarity with coma |
| dm_unspec | ICD10CM | E08.1 | Diabetes mellitus due to underlying condition with ketoacidosis |
| dm_unspec | ICD10CM | E08.10 | Diabetes mellitus due to underlying condition with ketoacidosis without coma |
| dm_unspec | ICD10CM | E08.11 | Diabetes mellitus due to underlying condition with ketoacidosis with coma |
| dm_unspec | ICD10CM | E08.2 | Diabetes mellitus due to underlying condition with kidney complications |
| dm_unspec | ICD10CM | E08.21 | Diabetes mellitus due to underlying condition with diabetic nephropathy |
| dm_unspec | ICD10CM | E08.22 | Diabetes mellitus due to underlying condition with diabetic chronic kidney disease |
| dm_unspec | ICD10CM | E08.29 | Diabetes mellitus due to underlying condition with other diabetic kidney complication |
| dm_unspec | ICD10CM | E08.3 | Diabetes mellitus due to underlying condition with ophthalmic complications |
| dm_unspec | ICD10CM | E08.31 | Diabetes mellitus due to underlying condition with unspecified diabetic retinopathy |
| dm_unspec | ICD10CM | E08.311 | Diabetes mellitus due to underlying condition with unspecified diabetic retinopathy with macular edema |
| dm_unspec | ICD10CM | E08.319 | Diabetes mellitus due to underlying condition with unspecified diabetic retinopathy without macular edema |
| dm_unspec | ICD10CM | E08.32 | Diabetes mellitus due to underlying condition with mild nonproliferative diabetic retinopathy |
| dm_unspec | ICD10CM | E08.321 | Diabetes mellitus due to underlying condition with mild nonproliferative diabetic retinopathy with macular edema |
| dm_unspec | ICD10CM | E08.3211 | Diabetes mellitus due to underlying condition with mild nonproliferative diabetic retinopathy with macular edema, right eye |
| dm_unspec | ICD10CM | E08.3212 | Diabetes mellitus due to underlying condition with mild nonproliferative diabetic retinopathy with macular edema, left eye |
| dm_unspec | ICD10CM | E08.3213 | Diabetes mellitus due to underlying condition with mild nonproliferative diabetic retinopathy with macular edema, bilateral |
| dm_unspec | ICD10CM | E08.3219 | Diabetes mellitus due to underlying condition with mild nonproliferative diabetic retinopathy with macular edema, unspecified eye |
| dm_unspec | ICD10CM | E08.329 | Diabetes mellitus due to underlying condition with mild nonproliferative diabetic retinopathy without macular edema |
| dm_unspec | ICD10CM | E08.3291 | Diabetes mellitus due to underlying condition with mild nonproliferative diabetic retinopathy without macular edema, right eye |
| dm_unspec | ICD10CM | E08.3292 | Diabetes mellitus due to underlying condition with mild nonproliferative diabetic retinopathy without macular edema, left eye |
| dm_unspec | ICD10CM | E08.3293 | Diabetes mellitus due to underlying condition with mild nonproliferative diabetic retinopathy without macular edema, bilateral |
| dm_unspec | ICD10CM | E08.3299 | Diabetes mellitus due to underlying condition with mild nonproliferative diabetic retinopathy without macular edema, unspecified eye |
| dm_unspec | ICD10CM | E08.33 | Diabetes mellitus due to underlying condition with moderate nonproliferative diabetic retinopathy |
| dm_unspec | ICD10CM | E08.331 | Diabetes mellitus due to underlying condition with moderate nonproliferative diabetic retinopathy with macular edema |
| dm_unspec | ICD10CM | E08.3311 | Diabetes mellitus due to underlying condition with moderate nonproliferative diabetic retinopathy with macular edema, right eye |
| dm_unspec | ICD10CM | E08.3312 | Diabetes mellitus due to underlying condition with moderate nonproliferative diabetic retinopathy with macular edema, left eye |
| dm_unspec | ICD10CM | E08.3313 | Diabetes mellitus due to underlying condition with moderate nonproliferative diabetic retinopathy with macular edema, bilateral |
| dm_unspec | ICD10CM | E08.3319 | Diabetes mellitus due to underlying condition with moderate nonproliferative diabetic retinopathy with macular edema, unspecified eye |
| dm_unspec | ICD10CM | E08.339 | Diabetes mellitus due to underlying condition with moderate nonproliferative diabetic retinopathy without macular edema |
| dm_unspec | ICD10CM | E08.3391 | Diabetes mellitus due to underlying condition with moderate nonproliferative diabetic retinopathy without macular edema, right eye |
| dm_unspec | ICD10CM | E08.3392 | Diabetes mellitus due to underlying condition with moderate nonproliferative diabetic retinopathy without macular edema, left eye |
| dm_unspec | ICD10CM | E08.3393 | Diabetes mellitus due to underlying condition with moderate nonproliferative diabetic retinopathy without macular edema, bilateral |
| dm_unspec | ICD10CM | E08.3399 | Diabetes mellitus due to underlying condition with moderate nonproliferative diabetic retinopathy without macular edema, unspecified eye |
| dm_unspec | ICD10CM | E08.34 | Diabetes mellitus due to underlying condition with severe nonproliferative diabetic retinopathy |
| dm_unspec | ICD10CM | E08.341 | Diabetes mellitus due to underlying condition with severe nonproliferative diabetic retinopathy with macular edema |
| dm_unspec | ICD10CM | E08.3411 | Diabetes mellitus due to underlying condition with severe nonproliferative diabetic retinopathy with macular edema, right eye |
| dm_unspec | ICD10CM | E08.3412 | Diabetes mellitus due to underlying condition with severe nonproliferative diabetic retinopathy with macular edema, left eye |
| dm_unspec | ICD10CM | E08.3413 | Diabetes mellitus due to underlying condition with severe nonproliferative diabetic retinopathy with macular edema, bilateral |
| dm_unspec | ICD10CM | E08.3419 | Diabetes mellitus due to underlying condition with severe nonproliferative diabetic retinopathy with macular edema, unspecified eye |
| dm_unspec | ICD10CM | E08.349 | Diabetes mellitus due to underlying condition with severe nonproliferative diabetic retinopathy without macular edema |
| dm_unspec | ICD10CM | E08.3491 | Diabetes mellitus due to underlying condition with severe nonproliferative diabetic retinopathy without macular edema, right eye |
| dm_unspec | ICD10CM | E08.3492 | Diabetes mellitus due to underlying condition with severe nonproliferative diabetic retinopathy without macular edema, left eye |
| dm_unspec | ICD10CM | E08.3493 | Diabetes mellitus due to underlying condition with severe nonproliferative diabetic retinopathy without macular edema, bilateral |
| dm_unspec | ICD10CM | E08.3499 | Diabetes mellitus due to underlying condition with severe nonproliferative diabetic retinopathy without macular edema, unspecified eye |
| dm_unspec | ICD10CM | E08.35 | Diabetes mellitus due to underlying condition with proliferative diabetic retinopathy |
| dm_unspec | ICD10CM | E08.351 | Diabetes mellitus due to underlying condition with proliferative diabetic retinopathy with macular edema |
| dm_unspec | ICD10CM | E08.3511 | Diabetes mellitus due to underlying condition with proliferative diabetic retinopathy with macular edema, right eye |
| dm_unspec | ICD10CM | E08.3512 | Diabetes mellitus due to underlying condition with proliferative diabetic retinopathy with macular edema, left eye |
| dm_unspec | ICD10CM | E08.3513 | Diabetes mellitus due to underlying condition with proliferative diabetic retinopathy with macular edema, bilateral |
| dm_unspec | ICD10CM | E08.3519 | Diabetes mellitus due to underlying condition with proliferative diabetic retinopathy with macular edema, unspecified eye |
| dm_unspec | ICD10CM | E08.352 | Diabetes mellitus due to underlying condition with proliferative diabetic retinopathy with traction retinal detachment involving the macula |
| dm_unspec | ICD10CM | E08.3521 | Diabetes mellitus due to underlying condition with proliferative diabetic retinopathy with traction retinal detachment involving the macula, right eye |
| dm_unspec | ICD10CM | E08.3522 | Diabetes mellitus due to underlying condition with proliferative diabetic retinopathy with traction retinal detachment involving the macula, left eye |
| dm_unspec | ICD10CM | E08.3523 | Diabetes mellitus due to underlying condition with proliferative diabetic retinopathy with traction retinal detachment involving the macula, bilateral |
| dm_unspec | ICD10CM | E08.3529 | Diabetes mellitus due to underlying condition with proliferative diabetic retinopathy with traction retinal detachment involving the macula, unspecified eye |
| dm_unspec | ICD10CM | E08.353 | Diabetes mellitus due to underlying condition with proliferative diabetic retinopathy with traction retinal detachment not involving the macula |
| dm_unspec | ICD10CM | E08.3531 | Diabetes mellitus due to underlying condition with proliferative diabetic retinopathy with traction retinal detachment not involving the macula, right eye |
| dm_unspec | ICD10CM | E08.3532 | Diabetes mellitus due to underlying condition with proliferative diabetic retinopathy with traction retinal detachment not involving the macula, left eye |
| dm_unspec | ICD10CM | E08.3533 | Diabetes mellitus due to underlying condition with proliferative diabetic retinopathy with traction retinal detachment not involving the macula, bilateral |
| dm_unspec | ICD10CM | E08.3539 | Diabetes mellitus due to underlying condition with proliferative diabetic retinopathy with traction retinal detachment not involving the macula, unspecified eye |
| dm_unspec | ICD10CM | E08.354 | Diabetes mellitus due to underlying condition with proliferative diabetic retinopathy with combined traction retinal detachment and rhegmatogenous retinal detachment |
| dm_unspec | ICD10CM | E08.3541 | Diabetes mellitus due to underlying condition with proliferative diabetic retinopathy with combined traction retinal detachment and rhegmatogenous retinal detachment, right eye |
| dm_unspec | ICD10CM | E08.3542 | Diabetes mellitus due to underlying condition with proliferative diabetic retinopathy with combined traction retinal detachment and rhegmatogenous retinal detachment, left eye |
| dm_unspec | ICD10CM | E08.3543 | Diabetes mellitus due to underlying condition with proliferative diabetic retinopathy with combined traction retinal detachment and rhegmatogenous retinal detachment, bilateral |
| dm_unspec | ICD10CM | E08.3549 | Diabetes mellitus due to underlying condition with proliferative diabetic retinopathy with combined traction retinal detachment and rhegmatogenous retinal detachment, unspecified eye |
| dm_unspec | ICD10CM | E08.355 | Diabetes mellitus due to underlying condition with stable proliferative diabetic retinopathy |
| dm_unspec | ICD10CM | E08.3551 | Diabetes mellitus due to underlying condition with stable proliferative diabetic retinopathy, right eye |
| dm_unspec | ICD10CM | E08.3552 | Diabetes mellitus due to underlying condition with stable proliferative diabetic retinopathy, left eye |
| dm_unspec | ICD10CM | E08.3553 | Diabetes mellitus due to underlying condition with stable proliferative diabetic retinopathy, bilateral |
| dm_unspec | ICD10CM | E08.3559 | Diabetes mellitus due to underlying condition with stable proliferative diabetic retinopathy, unspecified eye |
| dm_unspec | ICD10CM | E08.359 | Diabetes mellitus due to underlying condition with proliferative diabetic retinopathy without macular edema |
| dm_unspec | ICD10CM | E08.3591 | Diabetes mellitus due to underlying condition with proliferative diabetic retinopathy without macular edema, right eye |
| dm_unspec | ICD10CM | E08.3592 | Diabetes mellitus due to underlying condition with proliferative diabetic retinopathy without macular edema, left eye |
| dm_unspec | ICD10CM | E08.3593 | Diabetes mellitus due to underlying condition with proliferative diabetic retinopathy without macular edema, bilateral |
| dm_unspec | ICD10CM | E08.3599 | Diabetes mellitus due to underlying condition with proliferative diabetic retinopathy without macular edema, unspecified eye |
| dm_unspec | ICD10CM | E08.36 | Diabetes mellitus due to underlying condition with diabetic cataract |
| dm_unspec | ICD10CM | E08.37 | Diabetes mellitus due to underlying condition with diabetic macular edema, resolved following treatment |
| dm_unspec | ICD10CM | E08.37X1 | Diabetes mellitus due to underlying condition with diabetic macular edema, resolved following treatment, right eye |
| dm_unspec | ICD10CM | E08.37X2 | Diabetes mellitus due to underlying condition with diabetic macular edema, resolved following treatment, left eye |
| dm_unspec | ICD10CM | E08.37X3 | Diabetes mellitus due to underlying condition with diabetic macular edema, resolved following treatment, bilateral |
| dm_unspec | ICD10CM | E08.37X9 | Diabetes mellitus due to underlying condition with diabetic macular edema, resolved following treatment, unspecified eye |
| dm_unspec | ICD10CM | E08.39 | Diabetes mellitus due to underlying condition with other diabetic ophthalmic complication |
| dm_unspec | ICD10CM | E08.4 | Diabetes mellitus due to underlying condition with neurological complications |
| dm_unspec | ICD10CM | E08.40 | Diabetes mellitus due to underlying condition with diabetic neuropathy, unspecified |
| dm_unspec | ICD10CM | E08.41 | Diabetes mellitus due to underlying condition with diabetic mononeuropathy |
| dm_unspec | ICD10CM | E08.42 | Diabetes mellitus due to underlying condition with diabetic polyneuropathy |
| dm_unspec | ICD10CM | E08.43 | Diabetes mellitus due to underlying condition with diabetic autonomic (poly)neuropathy |
| dm_unspec | ICD10CM | E08.44 | Diabetes mellitus due to underlying condition with diabetic amyotrophy |
| dm_unspec | ICD10CM | E08.49 | Diabetes mellitus due to underlying condition with other diabetic neurological complication |
| dm_unspec | ICD10CM | E08.5 | Diabetes mellitus due to underlying condition with circulatory complications |
| dm_unspec | ICD10CM | E08.51 | Diabetes mellitus due to underlying condition with diabetic peripheral angiopathy without gangrene |
| dm_unspec | ICD10CM | E08.52 | Diabetes mellitus due to underlying condition with diabetic peripheral angiopathy with gangrene |
| dm_unspec | ICD10CM | E08.59 | Diabetes mellitus due to underlying condition with other circulatory complications |
| dm_unspec | ICD10CM | E08.6 | Diabetes mellitus due to underlying condition with other specified complications |
| dm_unspec | ICD10CM | E08.61 | Diabetes mellitus due to underlying condition with diabetic arthropathy |
| dm_unspec | ICD10CM | E08.610 | Diabetes mellitus due to underlying condition with diabetic neuropathic arthropathy |
| dm_unspec | ICD10CM | E08.618 | Diabetes mellitus due to underlying condition with other diabetic arthropathy |
| dm_unspec | ICD10CM | E08.62 | Diabetes mellitus due to underlying condition with skin complications |
| dm_unspec | ICD10CM | E08.620 | Diabetes mellitus due to underlying condition with diabetic dermatitis |
| dm_unspec | ICD10CM | E08.621 | Diabetes mellitus due to underlying condition with foot ulcer |
| dm_unspec | ICD10CM | E08.622 | Diabetes mellitus due to underlying condition with other skin ulcer |
| dm_unspec | ICD10CM | E08.628 | Diabetes mellitus due to underlying condition with other skin complications |
| dm_unspec | ICD10CM | E08.63 | Diabetes mellitus due to underlying condition with oral complications |
| dm_unspec | ICD10CM | E08.630 | Diabetes mellitus due to underlying condition with periodontal disease |
| dm_unspec | ICD10CM | E08.638 | Diabetes mellitus due to underlying condition with other oral complications |
| dm_unspec | ICD10CM | E08.64 | Diabetes mellitus due to underlying condition with hypoglycemia |
| dm_unspec | ICD10CM | E08.640 | Diabetes mellitus due to underlying condition with hypoglycemia without coma |
| dm_unspec | ICD10CM | E08.641 | Diabetes mellitus due to underlying condition with hypoglycemia with coma |
| dm_unspec | ICD10CM | E08.649 | Diabetes mellitus due to underlying condition with hypoglycemia without coma |
| dm_unspec | ICD10CM | E08.65 | Diabetes mellitus due to underlying condition with hyperglycemia |
| dm_unspec | ICD10CM | E08.69 | Diabetes mellitus due to underlying condition with other specified complication |
| dm_unspec | ICD10CM | E08.8 | Diabetes mellitus due to underlying condition with unspecified complications |
| dm_unspec | ICD10CM | E08.9 | Diabetes mellitus due to underlying condition without complications |
| Codes for Common Diseases Known to Affect Ligaments | | | |
| ligamentous_disease_var | ICD9CM | 759.82 | Marfan's Syndrome |
| ligamentous_disease_var | ICD10CM | Q87.40 | Marfan's Syndrome, unspecified |
| ligamentous_disease_var | ICD10CM | Q87.410 | Marfan's Syndrome with aortic dilation |
| ligamentous_disease_var | ICD10CM | Q87.418 | Marfan's Syndrome with other cardiovascular manifestations |
| ligamentous_disease_var | ICD10CM | Q87.42 | Marfan's Syndrome with ocular manifestations |
| ligamentous_disease_var | ICD10CM | Q87.43 | Marfan's Syndrome with skeletal manifestations |
| ligamentous_disease_var | ICD9CM | 756.83 | Ehlers-Danlos syndrome |
| ligamentous_disease_var | ICD10CM | Q79.60 | Ehlers-Danlos syndrome, unspecified |
| ligamentous_disease_var | ICD10CM | Q79.61 | Classical Ehlers-Danlos syndrome |
| ligamentous_disease_var | ICD10CM | Q79.62 | Hypermobile Ehlers-Danlos syndrome |
| ligamentous_disease_var | ICD10CM | Q79.63 | Vascular Ehlers-Danlos syndrome |
| ligamentous_disease_var | ICD10CM | Q79.69 | Other Ehlers-Danlos syndromes |
| ligamentous_disease_var | ICD10CM | M35.0B | sjogren syndrome with vasculitis |
| ligamentous_disease_var | ICD10CM | M05.2 | Rheumatoid vasculitis with rheumatoid arthritis |
| ligamentous_disease_var | ICD10CM | M05.20 | Rheumatoid vasculitis with rheumatoid arthritis of unspecified site |
| ligamentous_disease_var | ICD10CM | M05.21 | Rheumatoid vasculitis with rheumatoid arthritis of shoulder |
| ligamentous_disease_var | ICD10CM | M05.211 | Rheumatoid vasculitis with rheumatoid arthritis of right shoulder |
| ligamentous_disease_var | ICD10CM | M05.212 | Rheumatoid vasculitis with rheumatoid arthritis of left shoulder |
| ligamentous_disease_var | ICD10CM | M05.219 | Rheumatoid vasculitis with rheumatoid arthritis of unspecified shoulder |
| ligamentous_disease_var | ICD10CM | M05.22 | Rheumatoid vasculitis with rheumatoid arthritis of elbow |
| ligamentous_disease_var | ICD10CM | M05.221 | Rheumatoid vasculitis with rheumatoid arthritis of right elbow |
| ligamentous_disease_var | ICD10CM | M05.222 | Rheumatoid vasculitis with rheumatoid arthritis of left elbow |
| ligamentous_disease_var | ICD10CM | M05.229 | Rheumatoid vasculitis with rheumatoid arthritis of unspecified elbow |
| ligamentous_disease_var | ICD10CM | M05.23 | Rheumatoid vasculitis with rheumatoid arthritis of wrist |
| ligamentous_disease_var | ICD10CM | M05.231 | Rheumatoid vasculitis with rheumatoid arthritis of right wrist |
| ligamentous_disease_var | ICD10CM | M05.232 | Rheumatoid vasculitis with rheumatoid arthritis of left wrist |
| ligamentous_disease_var | ICD10CM | M05.239 | Rheumatoid vasculitis with rheumatoid arthritis of unspecified wrist |
| ligamentous_disease_var | ICD10CM | M05.24 | Rheumatoid vasculitis with rheumatoid arthritis of hand |
| ligamentous_disease_var | ICD10CM | M05.241 | Rheumatoid vasculitis with rheumatoid arthritis of right hand |
| ligamentous_disease_var | ICD10CM | M05.242 | Rheumatoid vasculitis with rheumatoid arthritis of left hand |
| ligamentous_disease_var | ICD10CM | M05.249 | Rheumatoid vasculitis with rheumatoid arthritis of unspecified hand |
| ligamentous_disease_var | ICD10CM | M05.25 | Rheumatoid vasculitis with rheumatoid arthritis of hip |
| ligamentous_disease_var | ICD10CM | M05.251 | Rheumatoid vasculitis with rheumatoid arthritis of right hip |
| ligamentous_disease_var | ICD10CM | M05.252 | Rheumatoid vasculitis with rheumatoid arthritis of left hip |
| ligamentous_disease_var | ICD10CM | I73 | Other peripheral vascular diseases |
| ligamentous_disease_var | ICD10CM | I73.0 | Raynaud's syndrome |
| ligamentous_disease_var | ICD10CM | I73.00 | Raynaud's syndrome without gangrene |
| ligamentous_disease_var | ICD10CM | I73.01 | Raynaud's syndrome with gangrene |
| ligamentous_disease_var | ICD10CM | I73.1 | Thromboangiitis obliterans [Buerger's disease] |
| ligamentous_disease_var | ICD10CM | I73.8 | Other specified peripheral vascular diseases |
| ligamentous_disease_var | ICD10CM | I73.81 | Erythromelalgia |
| ligamentous_disease_var | ICD10CM | I73.89 | Other specified peripheral vascular diseases |
| ligamentous_disease_var | ICD10CM | I73.9 | Peripheral vascular disease, unspecified |
| ligamentous_disease_var | ICD9CM | 710.4 | polymyositis |
| ligamentous_disease_var | ICD10CM | M33.20 | polymyositis, organ involvement unspecified |
| ligamentous_disease_var | ICD10CM | M33.21 | polymyositis with respiratory involvement |
| ligamentous_disease_var | ICD10CM | M33.22 | polymyositis with myopathy |
| ligamentous_disease_var | ICD10CM | M33.29 | polymyositis with other organ involvement |
| ligamentous_disease_var | ICD10CM | M33.90 | polymyositis, unspecified, organ involvement unspecified |
| ligamentous_disease_var | ICD10CM | M33.91 | polymyositis, unspecified, with respiratory involvement |
| ligamentous_disease_var | ICD10CM | M33.92 | polymyositis, unspecified with myelopathy |
| ligamentous_disease_var | ICD10CM | M33.93 | polymyositis, unspecified without myelopathy |
| ligamentous_disease_var | ICD10CM | M33.99 | polymyositis, unspecified with other organ involvement |
| ligamentous_disease_var | ICD10CM | L94.0 | localized scleroderma [morphea] |
| ligamentous_disease_var | ICD10CM | L94.1 | linear scleroderma |
| ligamentous_disease_var | CPT | 0489T | Autologous adipose-derived regenerative cell therapy for scleroderma in the hands; adipose tissue harvesting, isolation and preparation of harvested cells including incubation with cell dissociation enzymes, removal of non-viable cells and debris, determination of concentration and dilution of regenerative cells. |
| ligamentous_disease_var | CPT | 0490T | Autologous adipose-derived regenerative cell therapy for scleroderma in the hands; multiple injections in one or both hands. |
| ligamentous_disease_var | ICD9CM | 701 | circumscribed scleroderma |
| ligamentous_disease_var | ICD9CM | 695.4 | lupus erythematosus |
| ligamentous_disease_var | ICD9CM | 710 | systemic lupus erythematosus |
| ligamentous_disease_var | ICD10CM | M32.0 | drug enduced systemic lupus erythematosus |
| ligamentous_disease_var | ICD10CM | M32.10 | systemic lupus erythematosus, organ system involvement unspecified |
| ligamentous_disease_var | ICD10CM | M32.19 | other organ system involvement in systemic lupus erythematosus |
| ligamentous_disease_var | ICD10CM | M32.8 | other forms of systemic lupus erythemaosus |
| ligamentous_disease_var | ICD10CM | M32.9 | systemic lupus erythematosus, unspecified |
| ligamentous_disease_var | ICD9CM | 714 | Rheumatoid arthritis and other inflammatory polyarthopathies |
| ligamentous_disease_var | ICD9CM | 714 | Rheumatoid arthritis and other inflammatory polyarthopathies |
| ligamentous_disease_var | ICD9CM | 714.2 | other Rheumatoid arthritis with visceral or systemic invovelment |
| ligamentous_disease_var | ICD9CM | 714.3 | polyarticular juvenile rhuematoid arthritis, chronic or unspecific |
| ligamentous_disease_var | ICD9CM | 714.31 | polyarticular juvenile rhuematoid arthritis, acute |
| ligamentous_disease_var | ICD9CM | 714.32 | pauciarticular juvenile rhuematoid arthritis |
| ligamentous_disease_var | ICD9CM | 714.33 | monoarticular juvenile rheumatoid arthritis |
| ligamentous_disease_var | ICD10CM | M06 | Other rheumatoid arthritis |
| ligamentous_disease_var | ICD10CM | M06.0 | Rheumatoid arthritis without rheumatoid factor |
| ligamentous_disease_var | ICD10CM | M06.00 | Rheumatoid arthritis without rheumatoid factor unspecified site |
| ligamentous_disease_var | ICD10CM | M06.01 | Rheumatoid arthritis without rheumatoid factor, shoulder |
| ligamentous_disease_var | ICD10CM | M06.011 | Rheumatoid arthritis without rheumatoid factor, right shoulder |
| ligamentous_disease_var | ICD10CM | M06.012 | Rheumatoid arthritis without rheumatoid factor, left shoulder |
| ligamentous_disease_var | ICD10CM | M06.019 | Rheumatoid arthritis without rheumatoid factor, unspecified shoulder |
| ligamentous_disease_var | ICD10CM | M06.02 | Rheumatoid arthritis without rheumatoid factor, elbow |
| ligamentous_disease_var | ICD10CM | M06.021 | Rheumatoid arthritis without rheumatoid factor, right elbow |
| ligamentous_disease_var | ICD10CM | M06.022 | Rheumatoid arthritis without rheumatoid factor, left elbow |
| ligamentous_disease_var | ICD10CM | M06.029 | Rheumatoid arthritis without rheumatoid factor, unspecified elbow |
| ligamentous_disease_var | ICD10CM | M05.9 | Rheumatoid arthritis with rheumatoid factor, unspecified |
| Codes for Prior Shoulder Injury | | | |
| prior_injury | ICD10CM | S41.0% | Open wound of shoulder |
| prior_injury | ICD10CM | S41.1% | Open wound of upper arm |
| prior_injury | ICD10CM | S41.7% | Multiple open wounds of shoulder and upper arm |
| prior_injury | ICD10CM | S41.8% | Open wound of other and unspecified parts of shoulder girdle |
| prior_injury | ICD10CM | S42.0% | Fracture of clavicle |
| prior_injury | ICD10CM | S42.1% | Fracture of scapula |
| prior_injury | ICD10CM | S42.2% | fracture of upper end of humerus |
| prior_injury | ICD10CM | S42.7% | Multiple Fractures of clavicle, scapula and humerus |
| prior_injury | ICD10CM | S42.8% | Fracture of other parts of shoulder and upper arm |
| prior_injury | ICD10CM | S42.9% | Fracture of shoulder girldle, part unspecified |
| prior_injury | ICD10CM | S43.0% | Dislocation of shoulder joint |
| prior_injury | ICD10CM | S43.1% | Dislocation of Acromioclavicular joint |
| prior_injury | ICD10CM | S43.3% | Dislocation of other and unspecified parths of shoulder girdle |
| prior_injury | ICD10CM | S43.4% | Sprain and strain of shoulder joint |
| prior_injury | ICD10CM | S43.5% | Sprain and strain of acromioclavicular joint |
| prior_injury | ICD10CM | S43.8% | sprain of other specified parts of unspecified shoulder girdle |
| prior_injury | ICD10CM | S43.9% | sprain of other specified parts of shoulder girdle |
| prior_injury | ICD10CM | S46.0% | unpsecified Injury of rotator cuff |
| prior_injury | ICD10CM | S46.1% | Injury of muscle and tendon of long head of biceps |
| prior_injury | ICD10CM | S46.2% | Injury of muscle and tendon of other parts of biceps |
| prior_injury | ICD10CM | S46.3% | Injury of muscle and tendon of triceps |
| prior_injury | ICD10CM | S46.7% | Injury of multiple muscles and tendons at shoulder and upper arm |
| prior_injury | ICD10CM | S46.8% | Injury of other muscles and tendons at shoulder and upper arm level |
| prior_injury | ICD10CM | S46.9% | Injury of unspecified muscle and tendon at shoulder and upper arm level |
| prior_injury | ICD10CM | S49.7% | Multiple injuries of shoulder and upper arm |
| prior_injury | ICD10CM | S49.8% | Other specified injuries of shoulder and upper arm |
| prior_injury | ICD10CM | S49.9% | Unspecified injury of shoulder and upper arm |
| prior_injury | ICD10CM | M75.0% | Adhesive capsulitis |
| prior_injury | ICD10CM | M75.2% | Bicipital Tendinitis |
| prior_injury | ICD10CM | M75.3% | Calcific tendinitis |
| prior_injury | ICD10CM | M75.4% | impingment syndrome shoulder |
| prior_injury | ICD10CM | M75.5% | Bursitis right shoulder |
| prior_injury | ICD10CM | M75.8% | Other shoulder lesions |
| prior_injury | ICD10CM | M75.9% | shoulder lesion, unspecified |
| prior_injury | ICD10CM | M06.21% | Rheumatoid Bursitis |
| prior_injury | ICD10CM | M71.11% | Other infective bursitis |
| prior_injury | ICD10CM | M19.01% | Primary osteoarthritis, shoulder |
| prior_injury | ICD10CM | M19.21% | Secondary osteoarthritis, shoulder |
| prior_injury | ICD10CM | M19.01% | Post-traumatic osteoarthritis shoulder |
| prior_injury | ICD10CM | M24.01% | loose body in shoulder |
| prior_injury | ICD10CM | C47.1% | malignant neoplasm of peripheral nerves of upper limn |
| prior_injury | ICD10CM | C49.1% | malignant neoplasm of connective or soft tissue of upper limb |
| prior_injury | ICD10CM | D21.1% | Benign neoplasm of connective and other soft tissue of upper limb |
| prior_injury | ICD10CM | M00.01% | Staphylococcal arthritis of shoulder |
| prior_injury | ICD10CM | M00.11% | Pneumococcal arthritis of shoulder |
| prior_injury | ICD10CM | M00.21% | other streptococcal arhtritis of shoulder |
| prior_injury | ICD10CM | M00.81% | Arthritis due to other bacteria in shoulder |
| prior_injury | ICD10CM | M01.X1% | Direct infection of right shoulder… |
| prior_injury | ICD10CM | M12.51% | Traumatic Arthropathy of shoulder |
| prior_injury | ICD10CM | M12.81% | other specific arhtropathies not elsewhere classified |
| prior_injury | ICD10CM | M13.11% | mono arhtritis of shoulder |
| prior_injury | ICD10CM | M13.81% | Other specified arthritis shoulder |
| prior_injury | ICD10CM | M24.21% | Disorder of ligament shoulder |
| prior_injury | ICD10CM | M24.31% | pathological dislocation of shoulder |
| prior_injury | ICD10CM | M24.41% | recurrent dislocation of shoulder |
| prior_injury | ICD10CM | M24.51% | contracture of shouler |
| prior_injury | ICD10CM | M25.51% | pain in shoulder |
| prior_injury | ICD10CM | M25.61% | stiffness of shoulder |
| prior_injury | ICD10CM | M25.71% | osteophyte, right shoulder |
| prior_injury | ICD10CM | M60.01% | infective myositis shoulder |
| prior_injury | ICD10CM | M60.11% | interstitial myositis |
| prior_injury | ICD10CM | M60.21% | foreign body granuloma |
| prior_injury | ICD10CM | M60.81% | other myositis shoulder |
| prior_injury | ICD10CM | M61.01% | myositis ossificans traumatica shoulder |
| prior_injury | ICD10CM | M61.11% | myositis ossificans progressiva shoulder |
| prior_injury | ICD10CM | M61.21% | Paralytic calcification and ossification of muscle, shoulder |
| prior_injury | ICD10CM | M61.42% | other calcification of muscle shoulder |
| prior_injury | ICD10CM | M61.51% | other ossification of muscle shoulder |
| prior_injury | ICD10CM | M62.01% | separation of muscle (nontraumatic), right shoulder |
| prior_injury | ICD10CM | M62.21% | nontraumatic ischemic infraction of muscle shoulder |
| prior_injury | ICD10CM | M62.41% | contracture of muscle shoulder |
| prior_injury | ICD10CM | M62.51% | Muscle wasting and atrophy not otherwise classified shoulder |
| prior_injury | ICD10CM | M63.81% | disorder of muscle in disease classified elsewhere |
| prior_injury | ICD10CM | M65.01% | abscess of tendon sheath shoulder |
| prior_injury | ICD10CM | M65.11% | other infective (teno)synovitis, shoulder |
| prior_injury | ICD10CM | M65.81% | other synovitis and tenosynovitis, shoulder |
| prior_injury | ICD10CM | M66.11% | rupture of synovium, shoulder |
| prior_injury | ICD10CM | M66.21% | Spontaneous rupture of extensor tendon shoulder |
| prior_injury | ICD10CM | M66.31% | Spontaneous rupture of flexor tendon shoulder |
| prior_injury | ICD10CM | M66.81% | Spontaneous rupture of extensor tendon shoulder |
| prior_injury | ICD10CM | M67.31% | Transient synovitis shoulder |
| prior_injury | ICD10CM | M67.81% | other specified disorders of synovium and tendons |
| prior_injury | ICD10CM | M67.91% | unpspecified specified disorders of synovium and tendons |
| prior_injury | ICD10CM | M70.81% | other soft tissue disorder related to use overuse and pressure, shoulderr |
| prior_injury | ICD10CM | M70.91% | unpsecified soft tissue disorder related to use overuse and prssure shoulder |
| prior_injury | ICD10CM | M71.01% | Abscess of bursa shoulder |
| prior_injury | ICD10CM | M71.31% | other bursal cyst shoulder |
| prior_injury | ICD10CM | M71.81% | Other specified bursopathies shoulder |
| prior_injury | ICD10CM | M80.01% | Age related ostroporosis with fracture shoulder |
| prior_injury | ICD10CM | M80.81% | other osteoporosis with current pathological fracture shoulder |
| prior_injury | ICD10CM | M84.31% | stress fracture shoulder |
| prior_injury | ICD10CM | M84.41% | pathological fracture shoulder |
| prior_injury | ICD10CM | M84.51% | Pathological fracture in setting of neoplasm shoulder |
| prior_injury | ICD10CM | M84.61% | pathological fracture in other disease shoulder |
| prior_injury | ICD10CM | M84.81% | other disorder of continuity of bone shoulder |
| prior_injury | ICD10CM | M85.41% | solitary bone cyst shoulder |
| prior_injury | ICD10CM | M85.51% | Aneurysmal bone cyst shoulder |
| prior_injury | ICD10CM | M85.61% | other cyst of bone shoulder |
| prior_injury | ICD10CM | M85.81% | other specified disorder of bone shoulder |
| prior_injury | ICD10CM | M86.01% | Acute hematogenous osteomyelitis shoulder |
| prior_injury | ICD10CM | M86.11% | Other acute osteomyelitis shoulder |
| prior_injury | ICD10CM | M86.21% | subacute osteomyelitis shoulder |
| prior_injury | ICD10CM | M86.31% | Chronic multifocal osteomyelitis, shoulder |
| prior_injury | ICD10CM | M86.41% | Chronic osteomyelitis with draining sinus shoulder |
| prior_injury | ICD10CM | M86.51% | other chronic hematogenous osteomyelistis shoulder |
| prior_injury | ICD10CM | M86.61% | other chronic osteomyelitis shoulder |
| prior_injury | ICD10CM | M86.8X1 | Other osteomyelitis shoulder |
| prior_injury | ICD10CM | M87.01% | idiopathic aseptic necrosis of shoulder |
| prior_injury | ICD10CM | M87.11% | osteonecrosis due to drugs shoulder |
| prior_injury | ICD10CM | M87.21% | osteonecrosis due to previous trauma shoulder |
| prior_injury | ICD10CM | M87.31% | Other secondary osteonecrosis shoulder |
| prior_injury | ICD10CM | M87.81% | Other osteonecrosis shoulder |
| prior_injury | ICD10CM | M88.81% | osteitis deformans shoulder |
| prior_injury | ICD10CM | M89.01% | algoneurodystrophy shoulder |
| prior_injury | ICD10CM | M89.21% | other disorder of bone development and growth shoulder |
| prior_injury | ICD10CM | M89.31% | hypertrophy of bone shoulder |
| prior_injury | ICD10CM | M89.41% | other hypertrophic osteoarthopathy shoulder |
| prior_injury | ICD10CM | M89.51% | Osteolysis shoulder |
| prior_injury | ICD10CM | M89.61% | osteopathy after poliomyelitis shoulder |
| prior_injury | ICD10CM | M89.71% | Major osseous defect shoulder |
| prior_injury | ICD10CM | M89.8X1 | Other specified disorder of none shoulder |
| prior_injury | ICD10CM | M90.51% | Osteonecrosis in disease classified else where shoulder |
| prior_injury | ICD10CM | M90.61% | osteitis deformans in neoplastic disease shoulder |
| prior_injury | ICD10CM | M90.81% | osteopathy in disease classified elsewhere shoulder |
| prior_injury | ICD10CM | M93.21% | osteochondritis dissecans shoulder |
| prior_injury | ICD10CM | M93.81% | other specified osteochondropathies shoulder |
| prior_injury | ICD10CM | M93.91% | osteochronropathy shoudler |
| prior_injury | ICD10CM | M94.21% | Chondromalacia shoulder |
| prior_injury | ICD10CM | M94.8X1 | other specfied disorder of cartilage shoulder |
| prior_injury | ICD10CM | M97.31X% | periprosthetic fracture around internal prosthetic right shoulder joint |
| prior_injury | ICD10CM | M97.32X% | periprosthetic fracture around internal prosthetic left shoulder joint |
|  |  |  |  |
| prior_injury | ICD9 | 171.2 | malignant neoplasm of connective and other soft tissue of upper limb, including shoulder |
| prior_injury | ICD9 | 215.2 | Other benign neoplasm of connective and other soft tissue of upper limb, including shoulder |
| prior_injury | ICD9 | 711.01 | pyogenic arthritis, shoulder region |
| prior_injury | ICD9 | 711.11 | arthropathy associated with Reiter's disease and nonspecific urethritis, shoulder region |
| prior_injury | ICD9 | 711.21 | Arthropathy in behcet's syndrome |
| prior_injury | ICD9 | 711.31 | postdysenteric arthropathy |
| prior_injury | ICD9 | 711.41 | arthropathy associated with other bacterial disease |
| prior_injury | ICD9 | 711.51 | arthropathy associated with other viral disease |
| prior_injury | ICD9 | 711.61 | arthropathy associated with mycoses |
| prior_injury | ICD9 | 711..71 | arthropathy associated with helminthese |
| prior_injury | ICD9 | 711.81 | arthropathy associated with other infrectious and parasitic diseases |
| prior_injury | ICD9 | 711.91 | unspecified infective arthritis |
| prior_injury | ICD9 | 712.11 | chrondocalcinosis due to dicalcium phosphate crystals |
| prior_injury | ICD9 | 712.21 | chrondocalcinosis due to pyrophosphate phosphate crystals |
| prior_injury | ICD9 | 712.31 | chrondocalcinosis, unpsecified, shoulder region |
| prior_injury | ICD9 | 712.81 | unspecified crystal arthropathy |
| prior_injury | ICD9 | 712.91 | other specified crystal arthropathy |
| prior_injury | ICD9 | 715.11 | osteoarthrosis, localized, primary |
| prior_injury | ICD9 | 715.21 | osteoarthrosis, localized, secondary |
| prior_injury | ICD9 | 715.31 | osteoarthrosis, localized, not specified whether primary or secondary |
| prior_injury | ICD9 | 715.91 | osteoarthrosis, unspecified whether generalized or localized |
| prior_injury | ICD9 | 716.01 | Kaschin-Beck disease, shoulder region |
| prior_injury | ICD9 | 716.11 | traumatic arthropathy |
| prior_injury | ICD9 | 716.21 | Allergic arthritis, shoulder region |
| prior_injury | ICD9 | 716.31 | Climacteric arthritis, shoulder region |
| prior_injury | ICD9 | 716.41 | Transient arthropathy, shoulder region. |
| prior_injury | ICD9 | 716.51 | Unspecified polyarthropathy or polyarthritis, shoulder region |
| prior_injury | ICD9 | 716.61 | Unspecified monoarthritis, shoulder region |
| prior_injury | ICD9 | 716.81 | Other specified arthropathy, shoulder region |
| prior_injury | ICD9 | 716.91 | Arthropathy, unspecified, shoulder region |
| prior_injury | ICD9 | 718.01 | Articular cartilage disorder, shoulder region |
| prior_injury | ICD9 | 718.11 | Loose body in joint, shoulder region |
| prior_injury | ICD9 | 718.21 | Pathological dislocation of joint, shoulder region |
| prior_injury | ICD9 | 718.31 | Recurrent dislocation of joint, shoulder region |
| prior_injury | ICD9 | 718.41 | Contracture of joint, shoulder region |
| prior_injury | ICD9 | 718.51 | Ankylosis of joint, shoulder region |
| prior_injury | ICD9 | 718.71 | Developmental dislocation of joint, shoulder region |
| prior_injury | ICD9 | 718.81 | Other joint derangement, not elsewhere classified, shoulder region |
| prior_injury | ICD9 | 718.91 | Unspecified derangement of joint, shoulder region |
| prior_injury | ICD9 | 719.01 | Effusion of joint, shoulder region |
| prior_injury | ICD9 | 719.11 | Hemarthrosis, shoulder region |
| prior_injury | ICD9 | 719.21 | Villonodular synovitis, shoulder region |
| prior_injury | ICD9 | 719.31 | Palindromic rheumatism, shoulder region |
| prior_injury | ICD9 | 719.41 | Pain in joint, shoulder region |
| prior_injury | ICD9 | 719.51 | Stiffness of joint, not elsewhere classified, shoulder region |
| prior_injury | ICD9 | 719.61 | Other symptoms referable to joint, shoulder region |
| prior_injury | ICD9 | 719.81 | Other specified disorders of joint, shoulder region |
| prior_injury | ICD9 | 719.91 | Unspecified disorder of joint, shoulder region |
| prior_injury | ICD9 | 726.0 | Adhesive capsulitis of shoulder |
| prior_injury | ICD9 | 726.10 | Disorders of bursae and tendons in shoulder region, unspecified |
| prior_injury | ICD9 | 726.11 | Calcifying tendinitis of shoulder |
| prior_injury | ICD9 | 726.19 | Other specified disorders of bursae and tendons in shoulder region |
| prior_injury | ICD9 | 726.1 | Rotator cuff syndrome of shoulder and allied disorders |
| prior_injury | ICD9 | 726.2 | Other affections of shoulder region, not elsewhere classified |
| prior_injury | ICD9 | 730.01 | Acute osteomyelitis, shoulder region |
| prior_injury | ICD9 | 730.11 | Chronic osteomyelitis, shoulder region |
| prior_injury | ICD9 | 730.21 | Unspecified osteomyelitis, shoulder region |
| prior_injury | ICD9 | 730.31 | Periostitis, without mention of osteomyelitis, shoulder region |
| prior_injury | ICD9 | 730.71 | Osteopathy resulting from poliomyelitis, shoulder region |
| prior_injury | ICD9 | 730.81 | Other infections involving bone in diseases classified elsewhere, shoulder region |
| prior_injury | ICD9 | 730.91 | Unspecified infection of bone, shoulder region |
| prior_injury | ICD9 | 755.59 | Other anomalies of upper limb, including shoulder girdle |
| prior_injury | ICD9 | 755.5 | Other congenital anomalies of upper limb, including shoulder girdle |
| prior_injury | ICD9 | 79.71 | Closed reduction of dislocation of shoulder |
| prior_injury | ICD9 | 79.81 | Open reduction of dislocation of shoulder |
| prior_injury | ICD9 | 80.01 | Arthrotomy for removal of prosthesis without replacement, shoulder |
| prior_injury | ICD9 | 80.11 | Other arthrotomy, shoulder |
| prior_injury | ICD9 | 80.21 | Arthroscopy, shoulder |
| prior_injury | ICD9 | 80.31 | Biopsy of joint structure, shoulder |
| prior_injury | ICD9 | 80.41 | Division of joint capsule, ligament, or cartilage, shoulder |
| prior_injury | ICD9 | 80.71 | Synovectomy, shoulder |
| prior_injury | ICD9 | 80.81 | Other local excision or destruction of lesion of joint, shoulder |
| prior_injury | ICD9 | 80.91 | Other excision of joint, shoulder |
| prior_injury | ICD9 | 81.23 | Arthrodesis of shoulder |
| prior_injury | ICD9 | 81.80 | Other total shoulder replacement |
| prior_injury | ICD9 | 81.81 | Partial shoulder replacement |
| prior_injury | ICD9 | 81.82 | Repair of recurrent dislocation of shoulder |
| prior_injury | ICD9 | 81.83 | Other repair of shoulder |
| prior_injury | ICD9 | 81.88 | Reverse total shoulder replacement |
| prior_injury | ICD9 | 81.8 | Arthroplasty and repair of shoulder and elbow |
| prior_injury | ICD9 | 831.00 | Closed dislocation of shoulder, unspecified |
| prior_injury | ICD9 | 831.09 | Closed dislocation of shoulder, other |
| prior_injury | ICD9 | 831.0 | Closed dislocation of shoulder |
| prior_injury | ICD9 | 831.10 | Open dislocation of shoulder, unspecified |
| prior_injury | ICD9 | 831.19 | Open dislocation of shoulder, other |
| prior_injury | ICD9 | 831.1 | Open dislocation of shoulder |
| prior_injury | ICD9 | 831 | Dislocation of shoulder |
| prior_injury | ICD9 | 84.08 | Disarticulation of shoulder |
| prior_injury | ICD9 | 840.8 | Sprains and strains of other specified sites of shoulder and upper arm |
| prior_injury | ICD9 | 840.9 | Sprains and strains of unspecified site of shoulder and upper arm |
| prior_injury | ICD9 | 840 | Sprains and strains of shoulder and upper arm |
| prior_injury | ICD9 | 84.41 | Fitting of prosthesis of upper arm and shoulder |
| prior_injury | ICD9 | 880.00 | Open wound of shoulder region, without mention of complication |
| prior_injury | ICD9 | 880.09 | Open wound of multiple sites of shoulder and upper arm, without mention of complication |
| prior_injury | ICD9 | 880.0 | Open wound of shoulder and upper arm, without mention of complication |
| prior_injury | ICD9 | 880.10 | Open wound of shoulder region, complicated |
| prior_injury | ICD9 | 880.19 | Open wound of multiple sites of shoulder and upper arm, complicated |
| prior_injury | ICD9 | 880.1 | Open wound of shoulder and upper arm, complicated |
| prior_injury | ICD9 | 880.20 | Open wound of shoulder region, with tendon involvement |
| prior_injury | ICD9 | 880.29 | Open wound of multiple sites of shoulder and upper arm, with tendon involvement |
| prior_injury | ICD9 | 880.2 | Open wound of shoulder and upper arm, with tendon involvement |
| prior_injury | ICD9 | 880 | Open wound of shoulder and upper arm |
| prior_injury | ICD9 | 88.21 | Skeletal x-ray of shoulder and upper arm |
| prior_injury | ICD9 | 907.4 | Late effect of injury to peripheral nerve of shoulder girdle and upper limb |
| prior_injury | ICD9 | 923.00 | contusion of shoulder region |
| prior_injury | ICD9 | 923.09 | contusion of multiple sites of shoulder and upper arm |
| prior_injury | ICD9 | 923.0 | contusion of shoulder and upper arm |
| prior_injury | ICD9 | 927.00 | crushing injury of shoulder region |
| prior_injury | ICD9 | 927.0 | crushing injury of shoulder |
| prior_injury | ICD9 | 943.15 | erythema of shoulder |
| prior_injury | ICD9 | 943.35 | full thickness skin loss of shoulder |
| prior_injury | ICD9 | 943.45 | deep necrosis of underlying tissue, without mention of loss of body part, shoulder |
| prior_injury | ICD9 | 943.55 | deep necrosis of underlying tissue, with loss of body part, shoulder |
| prior_injury | ICD9 | 954 | injury to other nerves of trunkm excluding shoulder and pelvic girdles |
| prior_injury | ICD9 | 955.7 | injury to other specified nerve of shoulder girdle and upper limb |
| prior_injury | ICD9 | 955.8 | injury to multiole nerves of shoulder girdle and upper limb |
| prior_injury | ICD9 | 955.9 | injury to unspecified nerve of shoulder gerdle and upper limb |
| prior_injury | ICD9 | 955 | injury to peripheral nerve of shoulder gerdle and upper limb |
| prior_injury | ICD9 | 959.2 | shoulder and upper arm injury |
| prior_injury | ICD9 | V43.61 | Shoulder joint replacement |
| prior_injury | ICD9 | V49.67 | shoulder amputation status |

Table S2: Associations between diabetes exposures and rotator cuff tears across case-control study designs (with and without imaging requirement), with and without adjustment for BMI in full-sample and complete-case analysis scenarios, and with multiple imputation.

|  | **Code Non-Image** | | | |  | **NLP Non-Image** | | | |  | **Code Image** | | | |
| --- | --- | --- | --- | --- | --- | --- | --- | --- | --- | --- | --- | --- | --- | --- |
| **T1D** | DCT Cases N-T1D/N-Total (T1D%) | Controls N-T1D/N-Total (T1D%) | OR | 95% CI |  | DCT Cases N-T1D/N-Total (T1D%) | Controls N-T1D/N-Total (T1D%) | OR | 95% CI |  | DCT Cases N-T1D/N-Total (T1D%) | Controls N-T1D/N-Total (T1D%) | OR | 95% CI |
| **Full sample** |  |  |  |  |  |  |  |  |  |  |  |  |  |  |
| Unadjusted1 | 232/7285 (3.2%) | 393/31,382 (1.3%) | 2.54 | (2.16, 2.99) |  | 1387/47369 (2.9%) | 2465/203356 (1.2%) | 2.42 | (2.26, 2.58) |  | 83/2156 (3.8%) | 382/8939 (4.2%) | 0.90 | (0.70, 1.14) |
| Adjusted1 |  |  | 2.19 | (1.85, 2.59) |  |  |  | 2.09 | (1.96, 2.24) |  |  |  | 0.92 | (0.71, 1.17) |
| **Complete-case analysis** |  |  |  |  |  |  |  |  |  |  |  |  |  |  |
| Unadjusted2 | 213/4,526 (4.7%) | 266/10,387 (2.6%) | 1.84 | (1.53, 2.21) |  | 1146/22098 (5.2%) | 1667/66356 (2.5%) | 2.06 | (1.91, 2.23) |  | 77/1605 (4.8%) | 332/5293 (6.3%) | 0.76 | (0.59, 0.98) |
| Adjusted1 |  |  | 1.67 | (1.38, 2.01) |  |  |  | 1.87 | (1.73, 2.02) |  |  |  | 0.79 | (0.60, 1.02) |
| Adjusted2 |  |  | 1.57 | (1.30, 1.89) |  |  |  | 1.78 | (1.64, 1.92) |  |  |  | 0.75 | (0.58, 0.97) |
| **BMI Imputed - Full sample** |  |  |  |  |  |  |  |  |  |  |  |  |  |  |
| Adjusted2 |  |  | 2.05 | (1.73, 2.43) |  |  |  | 2.01 | (1.88, 2.15) |  |  |  | 0.87 | (0.68, 1.12) |
| **T2D** | DCT Cases N-T2D/N-Total (T2D%) | Controls N-T2D/N-Total (T2D%) | OR | 95% CI |  | DCT Cases N-T2D/N-Total (T2D%) | Controls N-T2D/N-Total (T2D%) | OR | 95% CI |  | DCT Cases N-T2D/N-Total (T2D%) | Controls N-T2D/N-Total (T2D%) | OR | 95% CI |
| **Full sample** |  |  |  |  |  |  |  |  |  |  |  |  |  |  |
| Unadjusted1 | 560/7285 (7.7%) | 1132/31382 (3.6%) | 2.13 | (1.92, 2.36) |  | 3202/47369 (6.8%) | 7179/203356 (3.5%) | 1.91 | (1.83, 2.00) |  | 246/2156 (11.4%) | 651/8939 (7.3%) | 1.57 | (1.34, 1.83) |
| Adjusted1 |  |  | 2.10 | (1.89, 2.34) |  |  |  | 1.77 | (1.69, 1.85) |  |  |  | 1.31 | (1.12, 1.53) |
| **Complete-case analysis** |  |  |  |  |  |  |  |  |  |  |  |  |  |  |
| Unadjusted2 | 546/4526 (12.0%) | 993/10387 (9.6%) | 1.26 | (1.13, 1.41) |  | 2953/22098 (13.4%) | 6344/66356 (9.6%) | 1.40 | (1.33, 1.46) |  | 234/1605 (14.6%) | 607/5293 (11.5%) | 1.27 | (1.08, 1.49) |
| Adjusted1 |  |  | 1.20 | (1.07, 1.34) |  |  |  | 1.26 | (1.20, 1.32) |  |  |  | 1.06 | (0.89, 1.25) |
| Adjusted2 |  |  | 1.12 | (1.00, 1.26) |  |  |  | 1.18 | (1.13, 1.24) |  |  |  | 1.00 | (0.84, 1.78) |
| **BMI Imputed - Full sample** |  |  |  |  |  |  |  |  |  |  |  |  |  |  |
| Adjusted2 |  |  | 1.95 | (1.75, 2.18) |  |  |  | 1.69 | (1.61, 1.76) |  |  |  | 1.23 | (1.05, 1.45) |
| **Any Diab** | DCT Cases N-AD/N-Total (Any Diab %) | Controls N-AD/N-Total (Any Diab%) | OR | 95% CI |  | DCT Cases N-AD/N-Total (Any Diab %) | Controls N-AD/N-Total (Any Diab%) | OR | 95% CI |  | DCT Cases N-AD/N-Total (Any Diab %) | Controls N-AD/N-Total (Any Diab%) | OR | 95% CI |
| **Full sample** |  |  |  |  |  |  |  |  |  |  |  |  |  |  |
| Unadjusted1 | 1197/7285 (16.4%) | 2546/31382 (8.1%) | 2.03 | (1.88, 2.18) |  | 7477/47369 (15.8%) | 16028/203356 (7.9%) | 2.00 | (1.94, 2.06) |  | 476/2156 (22.1%) | 1589/8939 (17.8%) | 1.24 | (1.11, 1.39) |
| Adjusted1 |  |  | 1.82 | (1.69, 1.96) |  |  |  | 1.75 | (1.69, 1.80) |  |  |  | 1.12 | (0.99, 1.26) |
| **Complete-case analysis** |  |  |  |  |  |  |  |  |  |  |  |  |  |  |
| Unadjusted2 | 1077/4526 (23.8%) | 1869/10387 (18.0%) | 1.32 | (1.22, 1.44) |  | 6103/22093 (27.6%) | 11804/66356 (17.8%) | 1.55 | (1.50, 1.61) |  | 440/1605 (27.4%) | 1347/5293 (25.4%) | 1.08 | (0.95, 1.22) |
| Adjusted1 |  |  | 1.21 | (1.12, 1.32) |  |  |  | 1.38 | (1.33, 1.43) |  |  |  | 0.97 | (0.86, 1.10) |
| Adjusted2 |  |  | 1.14 | (1.04, 1.24) |  |  |  | 1.30 | (1.25, 1.34) |  |  |  | 0.92 | (0.81, 1.05) |
| **BMI Imputed - Full sample** |  |  |  |  |  |  |  |  |  |  |  |  |  |  |
| Adjusted2 |  |  | 1.70 | (1.57, 1.83) |  |  |  | 1.67 | (1.62, 1.72) |  |  |  | 1.06 | (0.94, 1.20) |

Complete-case analysis: Effective sample size limited to individuals with information available on BMI

Unadjusted1: Unadjusted in the full SD sample size

Unadjsuted1: Unadjusted in effective sample size with BMI availability

Adjusted1: Adjusted for Age, sex, race/ethnicity

Adjusted2: Adjusted for Age, sex, race/ethnicity, and BM

| Table S3: Demographics and characteristics of rotator cuff tear cases and controls across sets with and without imaging restricted to individuals for whom BMI is not missing across the entire VUMC Synthetic Derivative. | | | | | | | |
| --- | --- | --- | --- | --- | --- | --- | --- |
|  | | Non-Image Required | | | | Image Required | |
|  | | Cases  (Code) | Controls ^**^  (Code + NLP) | Cases  (NLP) | Controls^**^  (Code + NLP) | Cases  (Code) | Controls (Code) |
| Total Count (N) | |  |  |  |  |  |  |
| All SD | | 8,482 | 33,928 | 54,846 | 219,384 | 2,632 | 10,528 |
| Participants with BMI data available | | 5,603 | 12,256 | 28,201 | 78,160 | 2,045 | 6,640 |
| Sex (N) | |  |  |  |  |  |  |
| Male | | 2,927 (52%) | 5,422 (44%) | 14,020 (50%) | 34,367 (44%) | 1,016 (50%) | 3,255 (49%) |
| Female | | 2,676 (48%) | 6,833 (56%) | 14,181 (50%) | 43,790 (56%) | 1,029 (50%) | 3,385 (51%) |
| Unknown | | - | 1(<0.1%) | - | 3 (<0.1%) | - | - |
| Age  Mean (SD) | | 61.08 (10.28) | 58.77 (12.28) | 62.88 (11.07) | 58.95 (12.33) | 61.71 (10.52) | 57.19 (11.61) |
| Race/Eth (N) | |  |  |  |  |  |  |
| Non-Hispanic | White | 3,744 (67%) | 7,122 (58%) | 18,538 (66%) | 46,098 (59%) | 1,343 (66%) | 4,482 (68%) |
|  | Black | 396 (7%) | 879 (7%) | 2,092 (7%) | 5,340 (7%) | 139 (7%) | 649 (10%) |
|  | Asian | 28 (0%) | 100 (1%) | 133 (1%) | 718 (1%) | 7 (0%) | 62 (1%) |
| Hispanic | | 124 (2%) | 309 (2%) | 589 (2%) | 2,014 (2%) | 50 (2%) | 182 (3%) |
| Other | | 1,311 (23%) | 3,846 (31%) | 6,849 (24%) | 23,990 (31%) | 506 (25%) | 1,265 (19%) |
| BMI Continuous (kg/m2)  Mean (SD) | | 30.33 (6.43) | 29.65 (7.24) | 30.27 (6.81) | 29.60 (8.08) | 29.85 (6.20) | 29.42 (7.01) |
| BMI categorical (N) | |  |  |  |  |  |  |
| Underweight  <18.5 (kg/m2) | | 29 (1%) | 222 (2%) | 284 (1%) | 1,357 (2%) | 14 (1%) | 115 (2%) |
| Normal  18.5-24.9 (kg/m2) | | 1,029 (18%) | 3,078 (25%) | 5,705 (20%) | 19,774 (25%) | 408 (20%) | 1,697 (26%) |
| Overweight  25-29.9 (kg/m2) | | 2,036 (36%) | 3,996 (33%) | 9,573 (34%) | 25,617 (33%) | 755 (37%) | 2,219 (33%) |
| Obese  >30 (kg/m2) | | 2,509 (45%) | 4,960 (40%) | 12,639 (45%) | 31,412 (40%) | 868 (42%) | 2,609 (39%) |
| Diabetes (N) | |  |  |  |  |  |  |
| Type I | | 213 (5%) | 266 (2%) | 1,146 (5%) | 1,667 (2%) | 77 (5%) | 332 (6%) |
| Type II | | 546 (11%) | 993 (9%) | 2,953 (12%) | 6,344 (9%) | 234 (13%) | 607 (10%) |
| Any | | 1,077 (19%) | 1,869 (15%) | 6,103 (22%) | 11,804 (15%) | 440 (22%) | 1,347 (20%) |
| None | | 4,526 (81%) | 10,387 (85%) | 22,098 (78%) | 66,356 (85%) | 1,605 (78%) | 5,293 (80%) |
| Ligamentous Disease: N (%) | | 519 (9%) | 619 (5%) | 2,810 (10%) | 3,718 (5%) | 214 (10%) | 593 (9%) |
| Prior Shoulder Injury N (%) | | 5,547 (99%) | 1,569 (13%) | 18,239 (65%) | 9,978 (13%) | 2,029 (99%) | 3,882 (58%) |
| Arthritis N (%) | | 3,354 (60%) | 260 (2%) | 7,619 (27%) | 1,669 (2%) | 1,315 (64%) | 801 (12%) |
| VUMC Utilization Average Days: Mean (SD) | | 56.68 (75.39) | 23.39 (37.30) | 46.16 (67.56) | 22.89 (37.02) | 58.22 (78.68) | 54.77 (77.28) |
| *This table only includes information on participants for whom BMI was available. **The non-image controls were sampled from the same, code+ NLP non-image set, but randomly selected to match either code-based or NLP-based cases at a 4:1 ratio and thus were ultimately composed of different participants | | | | | | | |

| Table S4: Demographics and characteristics of rotator cuff tear cases and controls across sets with and without imaging restricted to individuals for whom genetic data is available. | | | | | | | |
| --- | --- | --- | --- | --- | --- | --- | --- |
|  | | Not Image Required | | | | Image Required | |
|  |  | Cases (Code) | Cases (NLP) | Controls ^**^  (Code + NLP) | Cases (Code) | | Controls (Code) |
| Total Count (N) | |  |  |  |  | |  |
| All BioVU | | 1,045 | 6,430 | 68,369 | 306 | | 1,300 |
| Participants with BMI data available | | 878 | 4,694 | 49,971 | 283 | | 1,076 |
| Sex (N) | |  |  |  |  | |  |
| Male | | 404 (46%) | 2,063 (44%) | 21,369 (43%) | 126 (45%) | | 461 (43%) |
| Female | | 474 (54%) | 2,631 (56%) | 28,602 (57%) | 157 (55%) | | 615 (57%) |
| Age  Mean (SD) | | 62.76 (10.26) | 63.71 (11.07) | 59.21 (12.49) | 62.59 (10.25) | | 56.97 (11.69) |
| Race/Eth (N) | |  |  |  |  | |  |
| Non-Hispanic | White | 676 (77%) | 3,679 (78%) | 37,510 (75%) | 216 (76%) | | 804 (75%) |
|  | Black | 116 (13%) | 541 (12%) | 6,070 (12%) | 40 (14%) | | 161 (15%) |
|  | Asian | 3 (0%) | 25 (1%) | 503 (1%) | 2 (1%) | | 13 (1%) |
| Hispanic | | 20 (2%) | 81 (2%) | 913 (2%) | 7 (2%) | | 31 (3%) |
| Other | | 63 (7%) | 368 (8%) | 4,975 (10%) | 18 (6%) | | 67 (6%) |
| BMI Continuous (kg/m2)  Mean (SD) | | 30.49 (6.70) | 30.19 (6.86) | 29.50 (13.22) | 30.17 (6.16) | | 29.37 (7.43) |
| BMI categorical (N) | |  |  |  |  | |  |
| Underweight  <18.5 (kg/m2) | | 5 (1%) | 45 (1%) | 1,082 (2%) | 1 (0%) | | 24 (2%) |
| Normal  18.5-24.9 (kg/m2) | | 159 (18%) | 997 (21%) | 13,291 (27%) | 55 (19%) | | 293 (27%) |
| Overweight  25-29.9 (kg/m2) | | 317 (36%) | 1,565 (33%) | 15,967 (32%) | 96 (34%) | | 344 (32%) |
| Obese  >30 (kg/m2) | | 397 (45%) | 2,087 (44%) | 19,631 (39%) | 131 (46%) | | 415 (39%) |
| Diabetes (N) | |  |  |  |  | |  |
| Type I | | 61 (7%) | 356 (8%) | 2,734 (5%) | 18 (6%) | | 113 (11%) |
| Type II | | 97 (11%) | 376 (8%) | 2,670 (5%) | 22 (8%) | | 54 (5%) |
| Any | | 237 (30%) | 1,311 (28%) | 10,791 (22%) | 70 (25%) | | 270 (25%) |
| None | | 641 (73%) | 3,383 (72%) | 39,180 (78%) | 213 (75%) | | 806 (75%) |
| Ligamentous Disease: N (%) | | 164 (19%) | 836 (18%) | 4,597 (9%) | 57 (20%) | | 172 (16%) |
| Prior Shoulder Injury: N (%) | | 867 (99%) | 3,445 (73%) | 10,998 (22%) | 283 (100%) | | 697 (65%) |
| Arthritis: N (%) | | 465 (53%) | 1,253 (27%) | 1,658 (3%) | 167 (59%) | | 145 (13%) |
| VUMC Utilization  Average Days: Mean (SD) | | 111.06 (110.97) | 85.23 (93.68) | 50.58 (59.79) | 114,77 (108.97) | | 98.79 (99.14) |
| *This table only includes information on participants for whom BMI was available.  **In BioVU, all cases and controls were retained for analysis, and therefore there is only one set of non-image-based controls. | | | | | | | |

| Table S5: Associations between diabetes exposures and rotator cuff tears across case-control study designs (with and without imaging requirement) with adjustment for BMI in complete-case analysis scenarios in individuals for whom genetic data is available. | | | | | | | | | | | | |
| --- | --- | --- | --- | --- | --- | --- | --- | --- | --- | --- | --- | --- |
|  | Code Non-Image | | | | NLP Non-Image | | | | Code Image | | | |
|  | Cases | Controls^a^ | OR | 95% CI | Cases | Controls^a^ | OR | 95% CI | Cases | Controls | OR | 95% CI |
| Diabetes^**^ | | | | | | | | | | | | |
| TIDM v none | 61 v 641 | 2,734 v 39,180 | 1.36 | 1.04-1.76 | 356 v 3,383 | 2,734 v 39,180 | 1.51 | 1.34-1.69 | 18 v 213 | 113 v 806 | 0.60 | 0.35-0.99 |
| TIIDM v none | 97 v 641 | 2,670 v 39,180 | 2.22 | 1.78-2.74 | 376 v 3,383 | 2,670 v 39,180 | 1.63 | 1.45-1.82 | 22 v 213 | 54 v 806 | 1.54 | 0.90-2.55 |
| Any v none | 237 v 641 | 10,791 v 39,180 | 1.34 | 1.15-1.56 | 1,311 v 3,383 | 10,791 v 39,180 | 1.41 | 1.32-1.50 | 70 v 213 | 270 v 806 | 0.98 | 0.72-1.32 |
| ^**^Adjusted for Age, Sex, Race, and BMI (continuous)  ^a^Note: Though the algorithm for control classification is identical for code non-image and NLP, and thus the overall set of controls in the SD from which these two control groups were selected is identical, the control groups represented in this table are different as they were randomly selected to meet a 1:4 case-control ratio. | | | | | | | | | | | | |
